# Tumor Cell‐Intrinsic SETD2 Deficiency Reprograms Neutrophils to Foster Immune Escape in Pancreatic Tumorigenesis

**DOI:** 10.1002/advs.202202937

**Published:** 2022-12-01

**Authors:** Ningning Niu, Xuqing Shen, Li Zhang, Yueyue Chen, Ping Lu, Wenjuan Yang, Mingzhu Liu, Juanjuan Shi, Dapeng Xu, Yingying Tang, Xiaotong Yang, Yawen Weng, Xinxin Zhao, Lian‐Ming Wu, Yongwei Sun, Jing Xue

**Affiliations:** ^1^ State Key Laboratory of Oncogenes and Related Genes Stem Cell Research Center Shanghai Cancer Institute Ren Ji Hospital Shanghai Jiao Tong University School of Medicine 160 Pujian Rd Shanghai 200127 P. R. China; ^2^ Department of Biliary‐Pancreatic Surgery Ren Ji Hospital Shanghai Jiao Tong University School of Medicine 160 Pujian Rd Shanghai 200127 P. R. China; ^3^ Department of Radiology Ren Ji Hospital Shanghai Jiao Tong University School of Medicine 160 Pujian Rd Shanghai 200127 P. R. China

**Keywords:** immune evasion, neutrophil, pancreatic tumorigenesis, SETD2

## Abstract

Genetic and epigenetic alterations play central roles in shaping the immunosuppressive tumor microenvironment (TME) to evade immune surveillance. The previous study shows that SETD2‐H3K36me3 loss promotes KRAS‐induced pancreatic tumorigenesis. However, little is known about its role in remodeling the TME and immune evasion. Here, it is shown that *SETD2* deficiency can reprogram neutrophils to an immunosuppressive phenotype, thereby promoting immune escape during pancreatic tumor progression. By comprehensive profiling of the intratumoral immune cells, neutrophils are identified as the subset with the most significant changes upon *Setd2* loss. *Setd2*‐deficient pancreatic tumor cells directly enhance neutrophil recruitment and reprogramming, thereby inhibiting the cytotoxicity of CD8^+^ T cells to foster tumor progression. Mechanistically, it is revealed that *Setd2*‐H3K36me3 loss leads to ectopic gain of H3K27me3 to downregulate *Cxadr* expression, which boosts the PI3K‐AKT pathway and excessive expression of CXCL1 and GM‐CSF, thereby promoting neutrophil recruitment and reprogramming toward an immunosuppressive phenotype. The study provides mechanistic insights into how tumor cell‐intrinsic *Setd2* deficiency strengthens the immune escape during pancreatic tumorigenesis, which may offer potential therapeutic implications for pancreatic cancer patients with SETD2 deficiency.

## Graphical abstract

The authors demonstrate that the tumor‐intrinsic *Setd2* deficiency enhances recruitment and reprogramming of neutrophils in pancreatic tumor, thereby escaping from immune surveillance via inhibiting the cytotoxicity of CD8^+^ T cells. This work may provide an anti‐neutrophils strategy to potentiate the efficacy of immunotherapy in pancreatic cancer patients with *SETD2* deficiency.

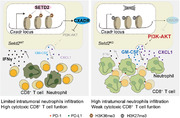

## 1 Introduction

The TME is highly heterogeneous and plays a vital role in tumor initiation and progression, immune evasion and therapy response. Tumor cells have the ability to sculpt their microenvironment by reprogramming surrounding cells.^[^ ^1^ ^]^ Accumulating evidence has shown that tumor cell–intrinsic factors play a central role in shaping the immunosuppressive TME to foster immune escape and tumor progression.^[^ ^1a,b^ ^]^ For example, oncogenic mutation of *KRAS* reprograms myeloid cells to favor the initiation and progression of pancreatic tumor.^[^ ^2^ ^]^ Recently, benefiting from CRISPR–Cas9 screening, several epigenetic regulators (e.g., *ARID1A*, *ASF1A*, *KDM3A*, and *SETDB1*) have been identified as cell‐intrinsic factors that trigger immune TME remodeling and thus have become promising targets to improve immunotherapy.^[^ ^3^ ^]^

Neutrophils are defined as a type of myeloid cell stored in the bone marrow and rapidly mobilized in physiological and pathological conditions.^[^ ^4^ ^]^ Similar to macrophages, the high plasticity of neutrophils enables them to be reprogrammed by tumor‐derived signals.^[^ ^4^, ^5^ ^]^ Whether tumor‐infiltrating neutrophils (TANs) exert pro‐ or antitumoral effects depends, in part, on tumor type and stage.^[^ ^6^ ^]^ In patients with pancreatic cancer, the circulating neutrophil‐to‐lymphocyte ratio is strongly associated with poor prognosis, and intratumoral neutrophils have the ability to favor tumor growth and metastasis by enhancing the immunosuppressive TME.^[^ ^7^ ^]^ However, little is known about whether tumor cell‐intrinsic genetic or epigenetic alterations are able to mobilize and reprogram neutrophils.

SETD2 is a histone H3K36 trimethyltransferase, and its mutation is widespread in diverse tumor types and accounts for 5% of all cancers in the TCGA cohort (521/10953). It functions as a tumor suppressor involved in multiple solid tumors with distinct mechanisms, including colorectal adenocarcinoma, kidney renal clear cell carcinoma, prostate cancer, and pancreatic ductal adenocarcinoma (PDAC).^[^ ^8^ ^]^ Therefore, it is of great significance to develop patient‐tailored therapies based on *SETD2‐*H3K36me3 mutations or deficiency. However, SETD2‐targeting molecules with therapeutic potential have not yet been developed. Whether tumor cell‐intrinsic *SETD2* alteration triggers TME reshaping, which might provide an alternative therapeutic strategy to treat patients with *SETD2* deficiency, remains to be explored.

Previously, with genetically engineered mouse models (GEMMs) and established cell lines of pancreatic tumor, we determined that *Setd2* deficiency facilitated *Kras*‐induced pancreatic tumorigenesis.^[^ ^8a^ ^]^ Strikingly, we observed a significant difference in proliferative capacity between *Setd2*‐proficient (*Setd2* ^WT^) and *Setd2*‐deficient (*Setd2* ^KO^) pancreatic tumors under in vivo condition that was not observed under in vitro culture conditions. The above results drove us to further investigate whether *Setd2* loss could reshape the TME to favor the pancreatic tumorigenesis. By comprehensively comparing the intratumoral immune cell profiles between *Setd2*‐proficient and *Setd2*‐deficient groups, neutrophils were the subset with the most significant change in pancreatic tumors. Future studies delineated that *Setd2* loss enhanced recruitment and neutrophil reprogramming, thereby facilitating immune escape by inhibiting the cytotoxicity of CD8^+^ T cells. Mechanistically, we revealed that *Setd2*‐H3K36me3 loss led to ectopic gain of H3K27me3 to downregulate *Cxadr* in pancreatic tumor cells, which boosted the PI3K‐AKT pathway and excessive expression of CXCL1 and GM‐CSF, thereby contributing to neutrophil recruitment and reprogramming toward an immunosuppressive phenotype. Herein, we highlight that tumor‐intrinsic *SETD2* deficiency reshapes the immunosuppressive TME via neutrophils in pancreatic tumorigenesis, potentially providing a therapeutic strategy for pancreatic cancer patients with *SETD2* deficiency.

## 2 Results

### 2.1 SETD2 Loss Reshapes the Immune TME, Particularly the Increase in Infiltrating Neutrophils, to Foster Tumor Progression

Our previous study determined the role of SETD2 in pancreatic tumorigenesis, in which *SETD2* deficiency promotes acinar‐to‐ductal metaplasia and EMT‐related metastasis.^[^ ^8a^ ^]^ Interestingly, we found that pancreatic tumor cells lacking *Setd2* (KPC1199‐*Setd2* ^KO^, hereafter termed *Setd2* ^KO^) grew slightly slower than control cells (*Setd2* ^WT^) in vitro (Figure S1A, Supporting Information). However, *Setd2* ^KO^ cells displayed remarkably high tumorigenicity in C57BL/6J wild‐type immunocompetent mice (Figure S1A,B, Supporting Information). According to transcriptome databases of human and murine pancreatic tumors, gene set enrichment analysis (GSEA) revealed that human pancreatic tumor with low level of SETD2 (TCGA‐PAAD) was coupled with enhanced inflammatory response (Figure S1C, Supporting Information). Similar results were obtained in murine *Setd2* ^KO^ cells (*versus* *Setd2* ^WT^, GES126302). Thus, we speculated that the TME, especially the immune TME, might contribute to the *SETD2*‐deficient tumor progression in vivo.

**Figure 1:**
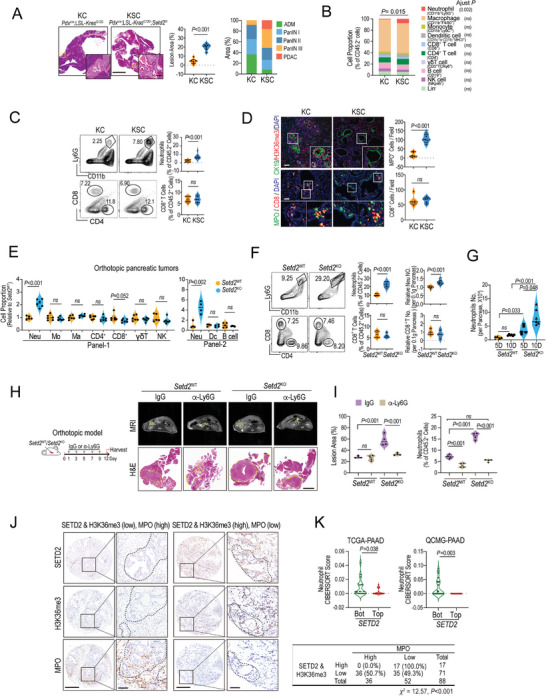
*Setd2* loss reshapes the immune TME, particularly the increase in infiltrating neutrophils, to foster tumor progression. A) Pancreatic tissues from the indicated mice at 13 weeks of age were used for H&E staining and lesion area quantification. Scale bars = 1 mm and 100 µm (for insets). Ratio of ADM, PanINs, and PDAC lesions in affected pancreatic areas from each group (*n* = 6 per group). B) Polychromatic flow cytometry analysis of leukocyte populations in pancreatic tissues of the indicated mice. The results are shown as the percentage of total CD45.2^+^ cells for the indicated subpopulations (*n* = 6 per group). C) Representative flow cytometric plots (left) and percentage of neutrophils and CD8^+^ T cells (right) in pancreatic tissues of the indicated mice at 13 weeks of age (*n* = 6–9 per group). D) Representative images (left) and quantification analysis (right) of H3K36me3‐, CK19‐, MPO‐, and CD8‐positive signals in pancreatic tissues of the indicated mice (shown in A) by immunofluorescence staining. Scale bars = 100 µm. E) Relative proportion of leukocyte subpopulations of total CD45.2^+^ cells in orthotopic *Setd2* ^WT^ and *Setd2* ^KO^ pancreatic tumors (*n* = 4–6 per group). F) Representative plots (left), cell proportion (middle), and absolute numbers (right) of neutrophils and CD8^+^ T cells in orthotopic *Setd2* ^WT^ and *Setd2* ^KO^ pancreatic tumors (*n* = 6–9 per group). G) The absolute numbers of neutrophils from the indicated orthotopic pancreatic tumors on the 5th and 10th days after inoculation (*n* = 6 per group). H,I) Mice bearing *Setd2* ^WT^ and *Setd2* ^KO^ pancreatic tumors (orthotopic model) were administered IgG and Ly6G neutralizing antibodies (1 mg kg^−1^ every other day) for 12 days. H) Representative MRI and H&E staining are shown (The yellow dashed line in the MRI image identifies the tumor growth on the pancreas, Scale bar = 2 mm). I) Quantification of lesion area and the percentage of neutrophils in tumor tissues from the indicated groups (right, *n* = 4 per groups). J) Left panel, representative IHC staining of SETD2, H3K36me3, and MPO in pancreatic tumors. Scale bar = 500 and 100 µm (for insets). Right, statistical analysis of the correlation between MPO and SETD2&H3K36me3 levels in the pancreatic tumor tissue array from the Renji cohort (*χ* ^2^ = 12.57, *P* value < 0.001). K) CIBERSORTx analyses to estimate the abundance of tumor‐infiltrating neutrophils in the database as indicated. Data are represented as the mean ± SEM. Statistical differences were determined with unpaired Student's t test in (A, C–F, and K); Two‐way ANOVA and Bonferroni's multiple comparisons test in (B); Two‐way ANOVA and Tukey's multiple comparisons test in (I); One way ANOVA and Tukey's multiple comparisons test in (G); and Chi‐square with Yates' correction test in (J).

By comparing our previously generated spontaneous pancreatic tumor models KC (*Pdx* ^cre^; LSL‐*Kras* ^G12D^) and KSC (*Pdx* ^cre^; LSL‐*Kras* ^G12D^; *Setd2* ^f/f^), we found a remarkable difference in the relative proportion of immune cells in the pancreas between KC and KSC mice (interaction *P =* 0.015, Two‐way ANOVA). Of note, the proportion of neutrophils (CD11b^+^Ly6G^+^) in the pancreas of KSC mice was significantly higher than that of KC mice, and no difference was found in other immune cell types between the two groups (**Figure** **1**A–C; Figure S1D, Supporting Information). In addition, neutrophils in the peripheral blood and spleen of KSC mice were also increased, as observed (Figure S1E, Supporting Information). Myeloperoxidase (MPO) staining further confirmed the elevated neutrophils in KSC tumors (Figure 1D). Next, orthotopic *Setd2* ^WT^ and *Setd2* ^KO^ tumors were further employed to decipher the proportion of tumor‐infiltrating immune cells. Consistent with the genetically engineered murine models (GEMMs), we found that the proportion and number of neutrophils in *Setd2* ^KO^ tumors were greatly increased (Figure 1E,F). Elevated neutrophils were also seen in the peripheral blood and spleen of *Setd2* ^KO^ tumor‐bearing mice (Figure S1F, Supporting Information). In addition, similar results were observed in *Setd2* ^KO^‐derived subcutaneous tumors and lung metastasis sites (Figure S1G,H, Supporting Information). Herein, above data imply that *Setd2* deficiency enhances intratumoral neutrophil infiltration during pancreatic tumor progression. Notably, although macrophages are the predominant subtype in the immune microenvironment of pancreatic tumors, we did not find any changes in macrophage numbers or polarization upon *Setd2* depletion (Figure S1I,J, Supporting Information).

The infiltrating neutrophils in the pancreas inoculated with *Setd2* ^KO^ pancreatic tumor cells increased significantly as early as 5 days and were maintained at higher levels as tumor progressed, suggesting their early role in the tumor progression of *Setd2* ^KO^ (Figure 1G). To assess the potential role of accumulated neutrophils, we used a Ly6G neutralizing antibody (*α*‐Ly6G) to deplete neutrophils in orthotopic tumor‐bearing mice. Interestingly, depletion of neutrophils significantly suppressed *Setd2* ^KO^ tumor growth but failed to shrink *Setd2* ^WT^ tumors (Figure 1H,I; Figure S2A, Supporting Information). In addition, *Setd2* ^KO^ tumor‐bearing mice injected with *α*‐Ly6G showed a reduction in tumor‐infiltrating neutrophils, as well as peripheral neutrophils, but had less effect on tumor‐infiltrating monocytes and macrophages (Figure 1I; Figure S2B,C, Supporting Information). Similar responses were observed in mice bearing subcutaneous *Setd2* ^WT^ or *Setd2* ^KO^ tumors upon depletion of neutrophils (Figure S2D,E, Supporting Information). Taken together, these data indicate that tumor‐infiltrating neutrophils are essential for *Setd2*‐deficient pancreatic tumor progression.

Furthermore, we assessed the clinical relevance between SETD2/H3K36me3 levels and neutrophil infiltration. Our previous study showed that the SETD2 expression level was closely related to H3K36me3 levels in pancreatic tumors.^[^ ^8a^ ^]^ Here, we observed that the expression of SETD2/H3K36me3 was negatively correlated with MPO levels in pancreatic tumors (Figure 1J, *χ* ^2^ = 12.57, *P* < 0.001). To further confirm our finding, we used CIBERSORTx, a digital cytometry tool, to evaluate the correlation between neutrophil infiltration and SETD2 levels.^[^ ^9^ ^]^ Consistently, tumor‐infiltrating neutrophils exhibited relatively higher proportions in tumors with low expression of SETD2 in TCGA‐PAAD and QCMG‐PAAD datasets (Figure 1K; Figure S3A,B, Supporting Information). Of note, similar results were observed in TCGA‐LUAD (lung adenocarcinoma) and TCGA‐COAD (colorectal adenocarcinoma) datasets as well, further indicating the role of *SETD2* deficiency in reshaping the immune TME, especially for neutrophils (Figure S3C–E, Supporting Information).

### 2.2 SETD2 Loss in Pancreatic Tumor Enhances the Recruitment and Immunosuppressive Phenotype of Neutrophils

**Figure 2:**
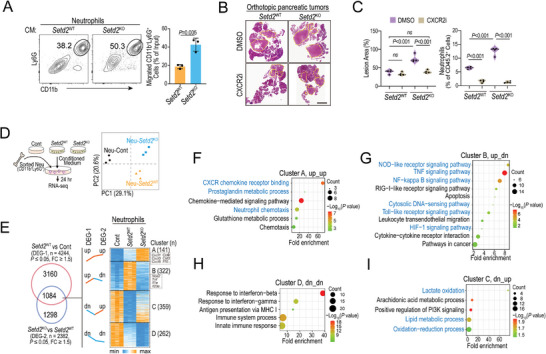
*Setd2* loss in pancreatic tumor promotes the recruitment and immunosuppressive phenotype of neutrophils. A) Neutrophil chemotaxis assay using 20% conditioned medium from *Setd2* ^WT^ or *Setd2* ^KO^ pancreatic tumor cells. Representative flow cytometry plots and statistical analysis are shown (*n* = 3). B,C) Orthotopic *Setd2* ^WT^ and *Setd2* ^KO^ tumor‐bearing mice were administered CXCR2 antagonist (CXCR2i, SB225002, 1 mg kg^−1^ every other day) and DMSO as a control for 12 days. B) Pancreatic tissues were collected for H&E staining. C) Quantification of lesion area and percentage of infiltrating neutrophils from the indicated mice (*n* = 4 per group). Scale bar = 2 mm. D) Neutrophils sorted from bone marrow (BM) were treated with conditioned medium from *Setd2* ^WT^ or *Setd2* ^KO^ cells, as well as control medium for 24 h. The above‐primed neutrophils were collected for RNA‐seq. Principal component analysis (PCA) scores plot indicating discrimination among Neu‐Cont, Neu‐*Setd2* ^WT^, and Neu‐*Setd2* ^KO^ (*n* = 3 per groups). E) Heatmap of RNA‐Seq data to compare the gene expression among the Neu‐Cont, Neu‐*Setd2* ^WT^, and Neu‐*Setd2* ^KO^ groups. F–I) Pathway enrichment of the indicated gene cluster based on RNA‐Seq data. The experiments had three replicates and were repeated three times (A). Data are represented as the mean ± SD (A) or mean ± SEM (C). Statistical differences were determined with unpaired Student's t test in (A), and one‐way ANOVA and Tukey's multiple comparisons test in (C).

To explore how *Setd2* loss leads to excessive neutrophil accumulation in the TME, we first compared the recruitment potential between *Setd2* ^KO^ and *Setd2* ^WT^ pancreatic tumor cells. Through in vitro chemotaxis analysis, we found that the supernatants of *Setd2* ^KO^ cells significantly accelerated neutrophil trafficking compared with those of *Setd2* ^WT^ cells (**Figure** **2**A). Neutrophil CXCR2 is essential for its emigration from bone marrow and trafficking toward sites of inflammation and tumors.^[^ ^10^ ^]^ As expected, intraperitoneal injection of the CXCR2 antagonist SB225002 in *Setd2* ^KO^ tumor‐bearing mice significantly decreased tumor burden in the pancreas, further reinforcing the protumoral function of neutrophils in *Setd2* ^KO^ tumors (Figure 2B,C; Figure S4A, Supporting Information). Of note, CXCR2 antagonist largely reduced tumor‐infiltrating and peripheral neutrophils, but had less effect on tumor‐infiltrating monocytes and macrophages (Figure 2C; Figure S4B–D, Supporting Information).

Neutrophils are extremely short‐lived cells and die upon spontaneous apoptosis, but their life span is prolonged once they arrive at inflammation or tumor sites.^[^ ^4^, ^6^ ^]^ As shown, conditioned medium from pancreatic tumor cells could largely enhance the longevity of neutrophils; however, there was no significant difference between the *Setd2* ^WT^ and *Setd2* ^KO^ groups (Figure S4E, Supporting Information). The above results indicate that the increase in neutrophils in *Setd2* ^KO^ pancreatic tumors mainly occurs through enhancing their chemotactic migration ability rather than anti‐apoptotic survival.

Neutrophil diversity and plasticity underlie its dual potential in the TME.^[^ ^4b^ ^]^ Therefore, the role of neutrophils in tumor progression has two sides, including antitumoral function via directly killing tumor cells and protumoral function by impairing T‐cell mediated cytotoxicity or enhancing metastasis through NETosis.^[^ ^6^, ^7^ ^]^ Next, we asked whether *Setd2* loss was able to reprogram neutrophils into protumoral function directly. To this end, BM‐derived neutrophils were freshly isolated and cultured in control medium (Cont), as well as conditioned medium from *Setd2* ^WT^ and *Setd2* ^KO^ pancreatic tumor cells. We performed RNA sequencing (RNA‐seq) on the above neutrophils, and principal component analysis (PCA) demonstrated distinct transcriptome profiles among the three groups of neutrophils (Figure 2D), implying that *Setd2‐*deficient pancreatic tumor cells may significantly rewire neutrophil characteristics. There were 4244 differentially expressed genes (DEG‐1, fold change ≥ 1.5, *P* value ≤ 0.05) between the Neu‐*Setd2* ^WT^ and Neu‐Cont groups and 2382 differentially expressed genes (DEG‐2, fold change ≥ 1.5, *P* value ≤ 0.05) between the Neu‐*Setd2* ^WT^ and Neu‐*Setd2* ^KO^ groups (Figure 2E). We focused on both altered genes from DEG‐1 and DEG‐2, and overlapped 1084 genes were divided into 4 clusters based on expression profiles, including cluster A (up_up, 141 genes), cluster B (up_dn, 322 genes), cluster C (dn_up, 359 genes) and cluster D (dn_dn, 262 genes) (Figure 2E; Tables S1–S4, Supporting Information). Next, gene ontology (GO) analyses were performed with the above gene clusters (Figure 2F–I). In line with our previous findings (Figure 2A), genes in cluster A (up_up) were closely related to neutrophil chemotaxis and CXCR chemokine receptor binding (Figure 2F). The prostaglandin metabolic process, which is associated with the immunosuppressive phenotype of neutrophils,^[^ ^11^ ^]^ was also upregulated in the Neu‐*Setd2* ^KO^ group (Figure 2F). Moreover, genes in clusters B (up_dn) and D (dn‐dn) were linked to the NOD‐like receptor signaling pathway, TNF signaling pathway, NF‐kappa B signaling pathway, cytosolic DNA‐sensing pathway, Toll‐like receptor signaling pathway, and HIF‐1 signaling pathway, indicating that the proinflammatory features of neutrophils were suppressed in the Neu‐*Setd2* ^KO^ group (Figure 2G,H). Moreover, genes in cluster C (dn_up) were associated with mitochondrial‐related pathways, such as lactate oxidation, oxidation–reduction and lipid metabolic processes (Figure 2I). Accumulating evidence has demonstrated that mitochondrial oxidative metabolism is critical for neutrophil chemotaxis, which is consistent with our finding that Neu‐*Setd2* ^KO^ have higher chemotactic ability. Some researchers raised the N1 and N2 terms to define neutrophils with pro‐inflammatory and anti‐inflammatory features, respectively.^[^ ^12^ ^]^ By applying marker genes of N1 and N2, we found that most of N1‐associated genes were significantly down‐regulated in Neu‐*Setd2* ^KO^ compared to Neu‐*Setd2* ^WT^, such as *Tnfa*, *Ifng, Nos2*, *Icam1*, *Cxcl9*, and *Cxcl10*. Moreover, some N2‐associated genes like *Mrc1*, *Il10*, and *Ccl7* were further upregulated in Neu‐*Setd2* ^KO^ compared to Neu‐*Setd2* ^WT^ (Figure S4F, Supporting Information). Collectively, *Setd2* deficiency in pancreatic tumor cells not only enhances neutrophil chemotaxis but also rewires them into a pro‐tumoral phenotype with immunosuppressive cues.

### 2.3 Neutrophils from *Setd2*‐Deficient Tumors Favor Immune Escape by Impeding CD8^+^ T Cells

**Figure 3:**
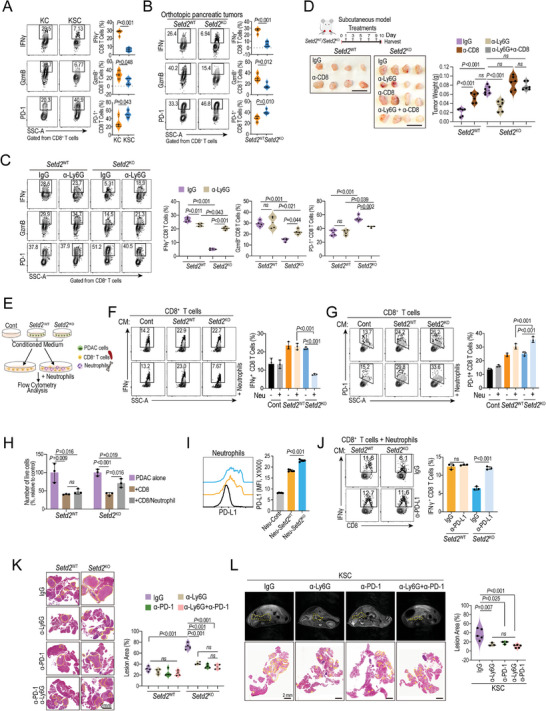
Neutrophils primed by *Setd2*‐deficient tumor cells favor immune escape via CD8^+^ T cells. A,B) Representative plots and statistical analysis of IFN*γ* and GzmB production, and surface PD‐1 of tumor‐infiltrating CD8^+^ T cells from the indicated mice (*n* = 4 per group). C) *Setd2* ^WT^ and *Setd2* ^KO^ tumor‐bearing mice (orthotopic model) were administered with IgG and Ly6G neutralizing antibodies (1 mg kg^−1^, every other day) for 12 days. Representative plots and statistical analysis of IFN*γ* and GzmB production, and surface PD‐1 of tumor‐infiltrating CD8^+^ T cells from the indicated mice (*n* = 4 per group). D) Mice bearing *Setd2* ^WT^ and *Setd2* ^KO^ tumors (subcutaneous model) were administered Ly6G neutralizing antibody, CD8 neutralizing antibody, the combination and IgG as a control every other day for 10 days. Tumors were collected for weight quantification (*n* = 6 per group). Scale bar = 1 cm. E) Schematic diagram of the coculture of CD8^+^ T cells (sorted from spleen) with and without neutrophils (sorted from bone marrow) upon treatment with different pancreatic tumor cell‐derived conditioned medium (CM). After 24 h, immune cells were used for flow cytometry analysis. F) Representative plots and statistical analysis of IFN*γ* production by CD8^+^ T cells from the indicated coculture groups as described in (E). G) Representative plots (left) and statistical analysis (right) of surface PD‐1 on CD8^+^ T cells from the indicated groups as described in (E). H) CTL‐mediated cytotoxicity analysis of CD8^+^ T cells on *Setd2* ^WT^ and *Setd2* ^KO^ cells with or without neutrophils. I) PD‐L1 levels on neutrophils after priming with the indicated conditioned medium for 24 h. Representative data (left) and statistical analysis (right) are shown. J) Conditioned medium (CM) from *Setd2* ^WT^ or *Setd2* ^KO^ pancreatic tumor cells was neutralized with PD‐L1 or control IgG for 6 h and then added to the CD8^+^ T‐cell and neutrophil coculture system for 24 h before analysis. Representative plots and bar graphs of IFN*γ* produced by CD8^+^ T cells are shown. K) Orthotopic *Setd2* ^WT^ and *Setd2* ^KO^ tumor‐bearing mice were administered as indicated (*α*‐Ly6G, 1 mg kg^−1^, and *α*‐PD‐1, 1 mg kg^−1^). Pancreatic tissues were collected for H&E staining (left) and lesion area quantification (right, *n* = 4 per group). Scale bar = 2 mm. L) Seven‐week‐old KSC mice were administered with Ly6G‐ and PD‐1‐ neutralizing antibodies, and IgG as a control for 6 weeks (twice every week). Representative MRI and H&E staining images are shown, the yellow dashed line in the MRI image identifies the pancreas. Scale bar = 2 mm (left). Quantification of lesion area from the indicated groups (right, *n* = 3–5 per groups). The experiments had three replicates and were repeated three times (F–J). Data are represented as the mean ± SEM (A–D and K–L) or mean ± SD (F–J). Statistical differences were determined with unpaired Student's t test in (A and B) and one‐way ANOVA and Tukey's multiple comparisons test in (C,D and F–L).

We speculated that loss of *Setd2* reprogrammed neutrophils to form an immunosuppressive TME, thereby allowing tumor cells to evade immune surveillance.^[^ ^13^ ^]^ Given that the proportion of CD8^+^ T cells did not differ significantly upon *Setd2* loss (Figure 1C–F), we speculated that the cytotoxicity of CD8^+^ T cells might be altered in *Setd2‐*deficient pancreatic tumors. To this end, we compared the proliferation, cytotoxicity, and immune checkpoint PD‐1 expression of infiltrating CD8^+^ T cells by using GEMM and orthotopic models of pancreatic tumor. As expected, in the tumor‐infiltrating CD8^+^ T cells, Ki67 expression did not differ between the two groups (Figure S5A, Supporting Information). However, in *Setd2*‐deficient GEMM and orthotopic models, we both observed increased PD‐1 expression and decreased IFN*γ* and granzyme B (GzmB) production in intratumoral CD8^+^ T cells, indicating their impaired cytotoxicity (**Figure** **3**A,B). More importantly, the impaired cytotoxicity of CD8^+^ T cells in *Setd2* ^KO^ tumors could be partially rescued by neutrophil depletion (Figure 3C). Next, to explore whether the protumoral function of neutrophils in *Setd2*‐deficient tumors was dependent on CD8^+^ T cells, we injected a neutralizing antibody against Ly6G and/or CD8 into tumor‐bearing mice. As shown, the CD8 neutralizing antibody (*α*‐CD8) had fewer effects on *Setd2* ^KO^ tumors than on *Setd2* ^WT^ tumors, consistent with the above finding that the cytotoxic CD8^+^ T cells were largely suppressed in *Setd2* ^KO^ tumors (Figure 3D). For *Setd2* ^KO^ tumors, depletion of neutrophils greatly reduced tumor size, which could be rescued by blockade of CD8^+^ T cells (Figure 3D; Figure S5B, Supporting Information). These data suggest that the tumor‐promoting effects of neutrophils in *Setd2* ^KO^ tumors are largely mediated through the inhibition of CD8^+^ T cells.

To further examine whether Neu‐*Setd2* ^KO^ (*Setd2* ^KO^ tumor‐primed neutrophils) had direct effects on CD8^+^ T cells, we set up a coculture system in which CD8^+^ T cells from the spleen were cocultured with or without neutrophils (sorted from bone marrow) upon treatment with conditioned medium from *Setd2* ^WT^ and *Setd2* ^KO^ cells, respectively (Figure 3E). Upon treatment with conditioned medium from tumor cells, the IFN*γ* levels of CD8^+^ T cells were greatly increased in both groups, while only the neutrophils primed by *Setd2* ^KO^ cells were able to reduce IFN*γ*, and surface PD‐1 levels in CD8^+^ T cells (Figure 3F–G). Meanwhile, we observed that Neu‐*Setd2* ^KO^ also decreased GzmB and perforin (Prf1) production in CD8^+^ T cells compared with Neu‐*Setd2* ^WT^, but did not affect CD8^+^ T cell proliferation (Figure S5C, Supporting Information). Consistently, only neutrophils cocultured with *Setd2* ^KO^ cells were able to impair the cytotoxicity of CD8^+^ T cells (Figure 3H). In contrast, the above findings could not be repeated in CD4^+^ T cells (Figure S5D,E, Supporting Information).

In addition to tumor cell‐derived PD‐L1, immunosuppressive neutrophils with elevated PD‐L1 levels participate in the inhibition of cytotoxic CD8^+^ T cells.^[^ ^7^, ^14^ ^]^ As shown in Figure 3I, neutrophils treated with *Setd2* ^KO^ cell‐conditioned medium displayed higher PD‐L1 levels than those treated with *Setd2* ^WT^ cell‐conditioned medium (Figure 3I). In the neutrophil and CD8^+^ T‐cell coculture system, blockade of PD‐L1 significantly rescued the IFN*γ* expression in CD8^+^ T cells that was suppressed by *Setd2* ^KO^‐primed neutrophils (Figure 3J). Notably, we observed a higher level of PD‐L1 on tumor‐infiltrating neutrophils (TANs) from *Setd2* ^KO^ orthotopic tumors than on TANs from *Setd2* ^WT^ orthotopic tumors, and the expression of PD‐L1 on TANs was much higher than that on epithelial tumor cells (Figure S5F, Supporting Information). These data suggest that PD‐L1/PD‐1 axis between neutrophils and CD8^+^ T cells plays a critical role in CD8^+^ T cell dysregulation in *Setd2*‐deficient pancreatic tumors. Next, we divided orthotopic *Setd2* ^WT^ and *Setd2* ^KO^ tumor‐bearing mice into 4 groups treated with Ly6G antibody (*α*‐Ly6G), PD‐1 antibody (a‐PD‐1), Ly6G plus PD‐1 antibodies, and control IgG (Figure 3K). As shown, the *Setd2* ^KO^ tumor burden on the pancreas was reduced in both a‐Ly6G and a‐PD‐1 treatment groups in *Setd2* ^WT^ and *Setd2* ^KO^ tumor‐bearing mice, accompanied by a marked increase in IFN*γ* production in CD8^+^ T cells (Figure 3K; Figure S5G, Supporting Information). As shown, in *Setd2* ^KO^ tumor‐bearing mice, blockade of Ly6G had a similar effect as a‐PD‐1 on reducing pancreatic tumor burden and increasing IFN*γ* production in tumor‐infiltrating CD8^+^ T cells (Figure 3K). Of note, no significant synergistic effects were observed with the combination of the two antibodies, and all treatments failed to reduce pancreatic tumor burden in *Setd2* ^WT^ tumor‐bearing mice (Figure 3K; Figure S5G, Supporting Information). Consistently, blockade of PD‐1 or Ly6G had similar inhibitory effects on tumor lesions and liver metastases in KSC mice (Figure 3L; Figure S5H, Supporting Information). Taken together, we propose that tumor‐infiltrating neutrophils in *Setd2*‐deficient pancreatic tumors impair the cytotoxicity of CD8^+^ T cells, at least partially, in a PD‐L1/PD‐1 axis‐dependent manner.

### 2.4 Tumor Cell‐Intrinsic SETD2 Loss Reprograms Neutrophils via AKT Activation

**Figure 4:**
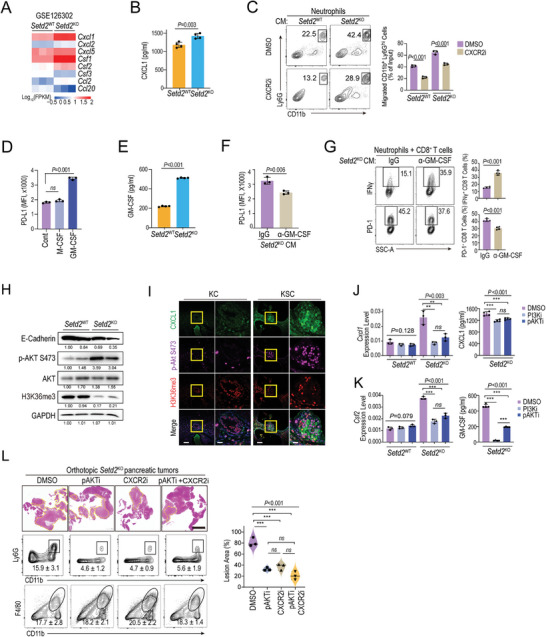
Tumor cell‐intrinsic SETD2 loss reprograms neutrophils via AKT activation‐mediated CXCL1 and GM‐CSF. A) Heatmap of RNA‐Seq data comparing the expression of cytokine‐ and chemokine‐encoding genes between *Setd2* ^WT^ and *Setd2* ^KO^ pancreatic tumor cells. B) ELISA analysis of CXCL1 levels in the supernatant of *Setd2* ^WT^ and *Setd2* ^KO^ cells. C) Neutrophil chemotaxis assay using 20% conditioned medium from *Setd2* ^KO^ cells with or without CXCR2 inhibitor. D) PD‐L1 levels on neutrophils upon treatment with M‐CSF and GM‐CSF for 24 h. E) ELISA analysis of GM‐CSF levels in the supernatant of *Setd2* ^WT^ and *Setd2* ^KO^ cells. F) Neutrophils were treated with conditioned medium (CM) from *Setd2* ^KO^ cells neutralized with GM‐CSF antibody or control IgG for 6 h. PD‐L1 levels on neutrophils were determined after 24 h. G) Representative plot (left) and statistical analysis (right) of IFN*γ* production and surface PD‐1 of CD8^+^ T cells from the indicated coculture groups. H) E‐cadherin, phosphorylation and total AKT, H3K36me3, and GAPDH levels in orthotopic *Setd2* ^WT^ and *Setd2* ^KO^ pancreatic tumors were analyzed by western blot. I) IHC staining of CXCL1 (green), phosphorylated AKT (purple), and H3K36me3 (red) in pancreatic tissue from KC and KSC mice. Scale bars = 100 µm and 20 µm (for inset). J,K) *Setd2* ^WT^ and *Setd2* ^KO^ cells were treated with LY294002 (PI3Ki, 500 nM), MK2206 (pAKTi, 500 nM), or DMSO as a control for 24 h. Left, the mRNA levels of J) *Cxcl1* and K) *Csf2* were determined with qPCR assay. Right panel, ELISA analyses of J) CXCL1 and K) GM‐CSF levels in the supernatant of *Setd2* ^WT^ and *Setd2* ^KO^ cells. L) Orthotopic *Setd2* ^KO^ tumor‐bearing mice were treated with pAKTi (200 mM kg^−1^, every other day), CXCR2i (1 mg kg^−1^, every other day), the combination and DMSO as a control for 10 days. Pancreatic tissues were collected for H&E staining (left) and lesion area quantification (right). Representative flow cytometry plots and proportion of neutrophils and macrophages from the indicated groups (*n* = 3 per group). The experiments had three replicates and were repeated three times (B–G, J–K). Data are represented as the mean ± SD (B–G, and J–K) or mean ± SEM (L). Statistical differences were determined with unpaired Student's t test in (B,C and E–G) and one‐way ANOVA and Tukey's multiple comparisons test in (D, and J–L). *ns*, *P* value > 0.05; *, 0.01 < *P* value ≤ 0.05; **, 0.001 < *P* value < 0.01; ***, *P* value ≤ 0.001.

Next, we aimed to elucidate mechanism by which *SETD2* deficiency in pancreatic tumor reprogrammed neutrophils. According to our previous RNA‐seq data, we found that the neutrophil chemoattractant‐related gene *Cxcl1*, granulocyte (macrophage and neutrophil) migration‐ and differentiation‐related genes *Csf1* (encoding M‐CSF) and *Csf2* (encoding GM‐CSF) were predominantly expressed and significantly elevated in *Setd2* ^KO^ pancreatic tumor cells (**Figure** **4**A). The elevated mRNA and protein levels of CXCL1 in *Setd2* ^KO^ were further validated (Figure 4B; Figure S6A, Supporting Information). As a chemokine receptor of CXCL1, CXCR2 expression was much higher on TANs than CXCR1 (Figure S6B, Supporting Information). As expected, blockade of the CXCL1‐CXCR2 axis almost completely eliminated the increase in neutrophil trafficking upon *Setd2* depletion (Figure 4C). GM‐CSF has been demonstrated to increase neutrophil PD‐L1 levels in gastric cancer.^[^ ^7a^ ^]^ Consistently, GM‐CSF, rather than M‐CSF, was able to upregulate neutrophil surface PD‐L1 expression (Figure 4D). First, elevated mRNA and protein levels of GM‐CSF were confirmed in *Setd2* ^KO^ (Figure 4E; Figure S6A, Supporting Information), and neutralizing GM‐CSF secreted by *Setd2* ^KO^ greatly downregulated PD‐L1 levels in neutrophils (Figure 4F). Next, we applied neutralized conditioned medium to the neutrophil/CD8^+^ T‐cell coculture system. As expected, blockade of GM‐CSF secreted by *Setd2* ^KO^ effectively rescued the decreased IFN*γ* expression and increased PD‐1 levels in CD8^+^ T cells (Figure 4G). In addition, we confirmed that the serum levels of CXCL1 and GM‐CSF in *Setd2* ^KO^ tumor‐bearing mice were also greatly increased (Figure S6C, Supporting Information). Moreover, in the GEMM model, elevated levels of CXCL1 and GM‐CSF were detected in H3K36me3‐negative ductal lesions from KSC mice compared with H3K36me3‐positive lesions from KC mice (Figure S6D,E, Supporting Information).

Previously, we reported that PI3K‐AKT signaling was boosted in *Setd2* ^KO^ pancreatic tumor cells.^[^ ^8a^ ^]^ Here, in an orthotopic model, we further confirmed the high level of AKT phosphorylation in *Setd2* ^KO^ tumors compared with *Setd2* ^WT^ tumors (Figure 4H). Consistently, activation of the AKT signal (p‐AKT) was detected in malignant lesions with low H3K36me3 levels in KSC mice, while the p‐AKT signal was barely found in H3K36me3‐positive lesions in KC mice (Figure 4I). It is worth noting that lesions with high levels of p‐AKT usually came with high levels of CXCL1 (Figure 4I). Previous studies have shown that PI3K‐AKT can upregulate various cytokines and chemokines, including CXCL1 and GM‐CSF.^[^ ^15^ ^]^ As expected, the upregulated mRNA and protein levels of *Cxcl1*/CXCL1 and *Csf2*/GM‐CSF were almost abolished by PI3K/AKT inhibitors in *Setd2* ^KO^ (Figure 4J,K; Figure S6F, Supporting Information). Furthermore, in the established orthotopic tumor model of *Setd2* ^KO^, we found that inhibition of the PI3K‐AKT pathway by MK2206 (pAKTi) partially reduced tumor burden and neutrophil recruitment in the pancreas but had no effect on macrophage abundance (Figure 4L). Of note, AKT and CXCR2 inhibitors had comparable effects on antitumor growth and neutrophil depletion. However, in combination with CXCR2 and AKT antagonists, no further beneficial effect was observed (Figure 4L).

### 2.5 SETD2‐H3K36me3 Epigenetically Regulates CXADR to Restrain AKT Activation

**Figure 5:**
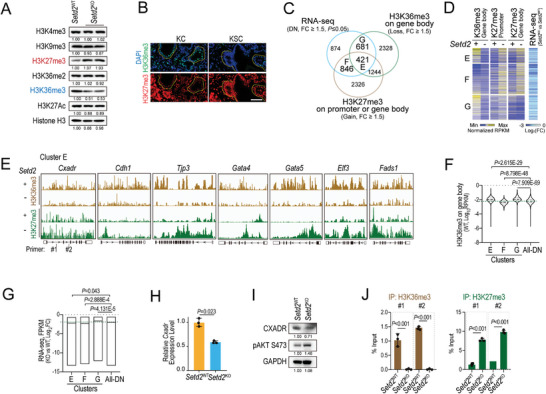
*Setd2* loss triggers hyperactivation of PI3K‐AKT via downregulation of *Cxadr*. A) The levels of the indicated histone modification marks were determined in *Setd2* ^WT^ and *Setd2* ^KO^ pancreatic tumor cells. B) Representative images of H3K36me3 and H3K27me3 in pancreatic tissues of the indicated mice by immunofluorescence staining. Scale bars = 100 µm. C) Venn diagram illustration of downregulated genes (RNA‐seq, FC ≥ 1.5, *P* ≤ 0.05) overlapping with H3K36me3‐loss‐ and H3K27me3‐gain‐related genes (CUT&Tag, FC ≥ 1.5) upon *Setd2* loss in pancreatic tumor cells. D) Heatmap illustrating the occupation of H3K36me3 and H3K27me3, as well as the mRNA expression of genes from clusters E, F, and G. E) Representative IGV screenshot of H3K36me3 and H3k27me3 CUT&Tag signals of the indicated genes from *Setd2* ^WT^ and *Setd2* ^KO^ cells. F) The violin plot represents the H3K36me3 distribution on genes from the indicated clusters. G) Box plot showing normalized transcriptional changes (*Setd2* ^KO^ versus *Setd2* ^WT^, Log_2_FC) of genes from the indicated clusters. The H) mRNA and I) protein levels of *Cxadr* were validated in *Setd2* ^WT^ and *Setd2* ^KO^ cells. J) Quantitative PCR analysis of H3K36me3 and H3K27me3 occupancies in the zones indicated in (E) of the *Cxadr* gene. IgG was used as the control. Data are represented as the mean with range (F and G) and mean ± SD (H and J). The experiments had three replicates and were repeated three times (H and J). Statistical differences were determined using unpaired Student's t test in (F–H and J).

We further investigated how *SETD2* loss augments AKT activation. Previous studies have shown that H3K36me3 plays an important role in cross‐talk with other histone marks, such as excluding H3K27me3, in embryonic development and prostate cancer progression.^[^ ^8^, ^16^ ^]^ Consistently, we found that the H3K27me3 levels were significantly increased along with the reduction in H3K36me3 levels in *Setd2* ^KO^ cells (**Figure** **5**A), as well as in pancreatic tumor lesions of KSC mice (Figure 5B). H3K27me3 modifications, regulated by the enhancer of zeste (EZH2)‐SET domain of polycomb repressive complex2 (PRC2) lead to the global gene silencing.^[^ ^8^, ^16^ ^]^ CUT&Tag analysis further confirmed that the global H3K27me3 occupancy was significantly elevated (Figure S7A, Supporting Information). Consistently, the transcript levels in *Setd2* ^WT^ cells were positively correlated with H3K36me3 levels and negatively correlated with H3K27me3 levels (Figure S7B, Supporting Information). Intriguingly, in *Setd2* ^KO^ cells, loss of H3K36me3 in gene bodies correlated with gene repression, and genes that acquired ectopic H3K27me3 either at promoters or in gene bodies were preferentially downregulated (Figure S7C–E, Supporting Information). Herein, our results demonstrate that transcriptional repression in *Setd2*‐deficient pancreatic tumor cells is associated with both H3K36me3 loss and ectopic H3K27me3 deposition.

To further define the direct targets of SETD2‐H3K36me3, we intersected the RNA‐seq (KO vs WT, DN) and CUT&Tag of H3K36me3 (KO vs WT, loss) and H3K27me3 (KO vs WT, gain) datasets (Figure 5C). We divided overlapping genes into three clusters, in which there were 421 downregulated genes (Cluster E) linked to both H3K36me3 loss and H3K27me3 gain, while 846 genes (Cluster F) and 681 genes (Cluster G) were related to H3K27me3 gain and H3K36me3 loss, respectively (Figure 5C–E; Figure S7F and Tables S5–S7, Supporting Information). Genes in clusters E and G were both marked by H3K36me3 in *Setd2* ^WT^ cells, but only genes in cluster E acquired ectopic H3K27me3 upon *Setd2* loss, while genes in cluster F with ectopic deposition of H3K27me3 were independent of H3K36me3 (Figure 5F). Notably, for H3K36me3 direct target genes, the expression of genes in cluster E was lower than that in cluster G, indicating that H3K27me3 plays an indispensable role in repressing gene expression upon *Setd2* loss (Figure 5G). The genes in cluster E were involved in cell–cell adhesion (e.g., *Cxadr*, *Cdh1*, and *Tjp3*), transcription (e.g., *Gata4*, *Gata5*, and *Elf3*), and lipid metabolic processes (*Fads1*), of which *Cxadr* (coxsackie and adenovirus receptor), the negative regulator of the PI3K‐AKT pathway, attracted our attention (Figure 5H; Figure S7G, Supporting Information). The mRNA and protein levels of *Cxadr* were both downregulated in *Setd2* ^KO^ cells, which could be partially rescued by EZH2 antagonist treatment (Figure 5H,I; Figure S7I–K, Supporting Information). Furthermore, using ChIP–qPCR, we confirmed the enrichment of H3K36me3 on *Cxadr* gene bodies (Figure 5J). With the deletion of *Setd2*, the H3K36me3 signals decreased, while the H3K27me3 marks increased in the indicated regions of *Cxadr* (Figure 5J). These results indicate that *Cxadr* is a direct target gene of Setd2/H3K36me3.

**Figure 6:**
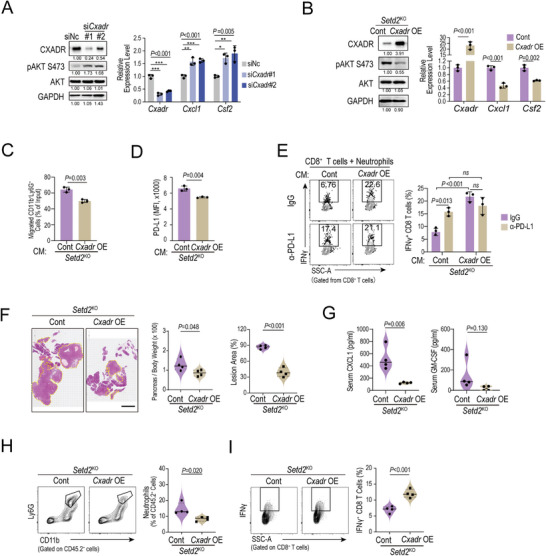
Overexpression of *Cxadr* rescues AKT activation, neutrophil infiltration and tumor progression upon *Setd2* deficiency. A) Western blot analysis of CXADR, phosphorylation and total AKT and GAPDH levels in cells as indicated (left). The mRNA expression of *Cxadr*, *Cxcl1* and *Csf2* was determined by qPCR assays (right). B) Left, western blot analysis of CXADR, phosphorylation and total AKT and GAPDH levels in *Setd2* ^KO^ cells overexpressing the *Cxadr* gene (*Cxadr* OE) and control cells (Cont). Right, the mRNA expression of *Cxadr*, *Cxcl1* and *Csf2* was determined with qPCR assays in the indicated cells. C) Neutrophil chemotaxis assay using to 20% conditioned medium from the indicated cells. D) Flow cytometry analysis of surface PD‐L1 levels on neutrophils after treatment with cell supernatants from the indicated pancreatic tumor cells. E) Representative plot (left) and statistical analysis (right) of IFN*γ* production in CD8^+^ T cells from the indicated coculture groups. F) Orthotopic tumors were inoculated with Cont and *Cxadr* OE pancreatic tumor cells for 14 days (*n* = 4 per group). Pancreatic tissues were collected for quantification of relative pancreas weight (left), H&E staining (middle) and lesion area (right). Scale bar = 1 mm. G) ELISA analyses of serum CXCL1 (left) and GM‐CSF (right) levels from (F). H) Representative plot (left) and statistical analysis (right) of tumor‐infiltrating neutrophils in pancreas tissues from (F). I) Representative plot (left) and statistical analysis (right) of IFN*γ*‐producing CD8^+^ T cells in pancreas tissues from (F). Data are represented as the mean ± SD (A–E) or mean ± SEM (F–I). The experiments had three replicates and were repeated three times (A–E). Statistical differences were determined with one‐way ANOVA and Tukey's multiple comparisons test in (A), unpaired Student's t test in (B–D and F–I), and two‐way ANOVA and Tukey's multiple comparisons test in (E). *ns*, *P* value > 0.05; *, 0.01 < *P* value ≤ 0.05; **, 0.001 < *P* value < 0.01.

Consistent with previous reports, we demonstrated that knockdown of *Cxadr* could facilitate AKT activation and E‐cadherin downregulation in pancreatic tumor cells (**Figure** **6**A).^[^ ^8a^ ^]^ In addition, the mRNA levels of both *Cxcl1* and *Csf2* were also upregulated in step with AKT phosphorylation (Figure 6A). More importantly, rescue of *Cxadr* expression in *Setd2* ^KO^ cells largely impaired AKT activation and reduced the mRNA levels of *Cxcl1* and *Csf2* (Figure 6B). Furthermore, the supernatants of *Cxadr*‐overexpressing *Setd2* ^KO^ cells decreased neutrophil trafficking and surface PD‐L1 levels (Figure 6C,D). In the neutrophil and CD8^+^ T‐cell coculture system, overexpression of *Cxadr* significantly rescued the IFN*γ* level of CD8^+^ T cells that was suppressed in *Setd2* ^KO^ cells (Figure 6E). Unlike parental *Setd2* ^KO^ cells, neutralizing PD‐L1 could not further enhance IFN*γ* expression when *Cxadr* was overexpressed (Figure 6E). We further examined the in vivo tumorigenicity and immune TME reshaping capability of *Cxadr*‐overexpressing *Setd2* ^KO^ cells. As shown, compared to paternal *Setd*2^KO^ cells, *Cxadr* overexpression retarded tumor growth (Figure 6F) and reduced serum CXCL1 and GM‐CSF levels in the corresponding tumor‐bearing mice (Figure 6G). In addition, *Cxadr* overexpression largely rescued the excessive neutrophil infiltration and cytotoxic CD8^+^ T‐cell suppression in *Setd2*‐deficient pancreatic tumors (Figure 6H,I). Taken together, *Setd2* deficiency leads to H3K36me3 loss and ectopic gain of H3K27me3 to downregulate *Cxadr* expression, thereby remodeling the TME by boosting AKT activation and related cytokines/chemokines.

## 3 Discussion

The immunosuppressive microenvironmen in vivo ork, we found that neutrophils were major executors of immune escape in *Setd2*‐deficient pancreatic tumors. A recent study demonstrated that the gain‐of‐function *Trp53* ^R172H^ mutation promoted the accumulation of neutrophils in pancreatic tumors.^[^ ^18^ ^]^ We observed that loss of *Setd2* could enhance neutrophil accumulation in pancreatic tumorigenesis, from *Kras* ^G12D^‐induced precancerous pancreatic intraepithelial neoplasia (PanIN) to malignant pancreatic tumors with *Kras* ^G12D/+^ and *Trp53* ^R172H/+^ mutations. More importantly, we further demonstrated the pro‐tumor function of infiltrating neutrophils in *Setd2*‐deficient pancreatic tumor progression. In addition, our data indicates that the role of SETD2 in reprogramming neutrophils is not affected by *Trp53* status. It is worth noting that the correlation between *SETD2* loss and elevated neutrophils might be not only in pancreatic cancer but also in lung and colon cancers, which still needs further investigation.

Genetic and epigenetic alterations confer inter‐ and intra‐heterogeneity of pancreatic tumors. Along with the increasing incidence of pancreatic cancer in recent years, targeted therapy for patients harboring specific mutations will be of great significance to improve their therapeutic efficacy. *SETD2* mutations are widespread in many tumor types, and the role of *SETD2* in multiple tumors, including pancreatic tumor, has been explored.^[^ ^8^, ^19^ ^]^ However, no small molecules or antibodies have shown specific targeted therapeutic potential for SETD2 and/or H3K36me3 loss.^[^ ^20^ ^]^ Understanding how SETD2‐H3K36me3 loss reprograms the TME might provide an alternative strategy for targeting *SETD2*‐deficient/low tumors. Here, our study reveals an anti‐neutrophil immunotherapy targeting pancreatic tumors with *SETD2/*H3K36me3 deficiency or low expression.

Of note, recent studies have demonstrated that the heterogeneity of the immune TME is a tumor cell‐intrinsic trait.^[^ ^21^ ^]^ As immunotherapy has achieved promising clinical benefits, a large portion of in vivo CRISPR–Cas9 screens have been used to identify the tumor‐intrinsic factors that might modulate immune escape or sensitivity to immunotherapy. Intriguingly, several epigenetic regulators (e.g., *Setdb1*, *Asf1a*, *Ezh2*, *Kdm3a*) were screened out with cell‐intrinsic effects on tumor immunogenicity or sensitivity to immunotherapy, raising the possibility that epigenetic therapy could enhance the efficacy of immunotherapy.^[^ ^3^, ^22^ ^]^ However, little is known about how epigenetic regulators contribute to immune escape in pancreatic cancer. From a different perspective, our study identified that SETD2 contributed to immune surveillance in pancreatic tumorigenesis. Oncogenic *Kras* activation drives immune evasion,^[^ ^23^ ^]^ and loss of *Setd2* further enhanced immune evasion by reprogramming neutrophils into an immunosuppressive phenotype to inhibit the cytotoxicity of CD8^+^ T cells rather than directly affecting CD8^+^ T cells (Figure 3A,B,E,G). Our findings provide more evidence for how epigenetic dysregulation in tumor cells contributes to immune evasion.

More interestingly, a recent study utilized an autochthonous pancreatic tumor model, KPC mice, to uncover the diversity of epigenetic landscapes in tumor cells with or without T‐cell infiltration.^[^ ^21^ ^]^ The key tumor cell‐intrinsic immune regulator *Cxcl1* increased in a non‐T‐cell‐inflamed microenvironment due to enhanced chromatin accessibility.^[^ ^21^ ^]^ Recently, a study proved that suppression of neutrophils with lorlatinib attenuated pancreatic cancer growth and improved immune checkpoint blockade treatment.^[^ ^24^ ^]^ In line with our study, these findings indicate the importance of neutrophils in modulating the immune TME of pancreatic tumors. In addition, we also noticed elevated levels of several CXCR2 ligand‐encoding genes, including *Cxcl1*, *Cxcl3* and *Cxcl5*, in Neu‐*Setd2* ^KO^ cells compared with Neu‐*Setd2* ^WT^ cells, indicating that neutrophils reprogrammed by *Setd2*‐deficient pancreatic tumor cells could further strengthen the recruitment of neutrophils (Table S1, Supporting Information). In addition to enhancing the PD‐L1/PD‐1 axis, we also found elevated arachidonic acid and prostaglandin metabolic processes in *Setd2* ^KO^‐primed neutrophils, which contributed to the immunosuppressive function of neutrophils (Figure 2F–I).^[^ ^7^, ^11^ ^]^

SETD2 is the primary methyltransferase catalyzing H3K36 trimethylation (H3K36me3), which is associated with transcription elongation, RNA splicing and DNA repair. Recent studies have shown that H3K36me3 also participates in cross‐talk with other chromatin marks, including antagonizing H3K4me3 and H3K27me3.^[^ ^8^, ^16^ ^]^ Similarly, loss of *Setd2* in pancreatic tumor cells triggered a profound decrease in H3K36me3 levels, accompanied by an increase in H3K27me3 levels. Downregulated genes in *Setd2* ^KO^ cells were more related to ectopic spreading of H3K27me3 both on transcribed regions (gene body) and promoters. Of note, only approximately 30% of genes with H3K27me3 gain were guided by H3K36me3 loss. In addition, the redistribution of H3K27me3 also led to H3K27me3 loss at the promoter or gene body of other gene loci, accounting for 39.2% of the upregulated genes in *Setd2* ^KO^ cells (Figure S7H, Supporting Information). Yuan et al recently showed that SETD2 was able to methylate EZH2 at K735 to promote EZH2 degradation, which provides a mechanism for how H3K36m3 excludes H3K27me3 signals during prostate cancer development.^[^ ^8b^ ^]^ However, in different contexts, whether and how H3K36me3 antagonizes H3K27me3 remains to be explored in depth.

## 4 Conclusion

In summary, our study revealed that *Setd2* deficiency directly enhanced the recruitment and reprogramming of neutrophils, thereby facilitating immune escape and fostering tumor progression by inhibiting cytotoxicity of CD8^+^ T cells. Mechanistically, we revealed that SETD2‐H3K36me3 loss directly downregulated *Cxadr* expression in pancreatic tumor cells, allowing for PI3K‐AKT activation and the release of excessive CXCL1 and GM‐CSF, which attracted neutrophils and reprogrammed them toward an immunosuppressive phenotype. Our work provides mechanistic insights into how tumor‐intrinsic SETD2 deficiency reshapes the immunosuppressive TME via neutrophils, thereby providing the potential use of anti‐neutrophil immunotherapy in pancreatic cancer patients with SETD2 deficiency.

## 5 Experimental Section

### Mouse Models and Treatments

*Pdx* ^cre^; LSL‐*Kras* ^G12D^; *Setd*2^f/f^ (KSC), *Pdx* ^cre^; LSL‐*Kras* ^G12D^ (KC) were generated as described.^[^ ^8a^ ^]^ All genetically modified mice were on a C57BL/6J background. C57BL/6J wild‐type mice were purchased from Shanghai SLAC Laboratory Animal Co., Ltd. (Shanghai, China). Age‐ and sex‐matched mice were used in the following experiments. *Setd2* ^WT^ and *Setd2* ^KO^ pancreatic tumor cells (1 × 10^6^) were injected into the pancreas of C57BL/6J mice at 8 weeks of age to establish orthotopic tumor models. Mice were sacrificed at the indicated times after injection. Subcutaneous tumor models were established by subcutaneous injection of *Setd2* ^WT^ and *Setd2* ^KO^ pancreatic tumor cells (1 × 10^6^) into 8‐week‐old C57BL/6J mice. For the lung metastasis model, *Setd2* ^WT^ and *Setd2* ^KO^ pancreatic tumor cells (5 × 10^5^) were injected into 8‐week‐old C57BL/6J mice via the tail vein, and mice were harvested 2 weeks after injection for lung tumor burden analysis. In the treatment groups, mice (1) received i.p. injection of *α*‐Ly6G (1 mg/kg/time, BE0075‐1, clone‐1A8, BioXcell), or (2) *α*‐PD‐1 (1 mg/kg/time, BE0146, clone‐RMP1‐14, BioXcell), or (3) combined (1) and (2), or (4) *α*‐CD8 (1 mg/kg/time, BE0004‐1, clone‐53‐6.7, BioXcell), or (5) combined (1) and (4), or (6) IgG (BE0089, clone‐2A3, BioXcell); (7) CXCR2 inhibitor, SB225002 (1 mg/kg/time, S7651, Selleck); or (8) inhibitor of phosphorylation AKT, MK2206 (200 mM/kg/time, S1078, Selleck); (9) combined (7) and (8), or (10) dimethyl sulfoxide as indicated.

### Magnetic Resonance Imaging (MRI)

The mice underwent repetitive MRI examinations (at time points) using a 7‐Tesla dedicated animal scanner (Bruker, Biospin, Ettlingen, Germany) and a one‐channel circular polarized volume coil (Bruker, Ettlingen, Germany). To minimize motion effects in the imaging process, animals were anesthetized by 2.0% isoflurane inhalation, and their respiration was monitored and kept constant between 30–50 breaths per min during the entire examination. The imaging protocol consisted of a T1‐weighted spin echo and a T2‐weighted turbo spin echo sequence. T2 maps were generated using a multislice multiecho sequence with the following parameters: TE1 = 6 ms, ΔTE = 6 ms, Echoes = 12, TR = 3000 ms FOV = 30 mm×30 mm, slice thickness = 1 mm, slice gap = 0.2 mm, slices = 10, matrix128×128, Average = 2, BW = 125000 Hz. To avoid respiratory artifacts, all sequences were triggered by respiratory gating. The MRI data analysis was accomplished by postprocessing software of the Biospin system (Paravision 6.0.1, Bruker, Ettlingen, Germany).

### Deciphering the Proportion of Tumor‐Infiltrating Immune Cells

CIBERSORTx was employed to estimate the abundance of tumor‐infiltrating immune cells.^[^ ^9^ ^]^ For a specific sample, the most abundant types of immune cells were identified within the TME by using the R package CIBERSORTx (R/supportFunc_cibersort. R). Statistical analyses were performed using GraphPad Software (Prism 9.0). All data are presented as the mean ± SEM or mean with range.

### CUT&Tag Analysis

The CUT&Tag assay was performed following the manual of the hyperactive pG‐Tn5/pA‐Tn5 transposase for CUT&Tag kit (Vazyme, TD901). DNA libraries were prepared according to the manufacturer's instructions of the Trueprep index kit v2 (Vazyme, TD202). The DNA library was sequenced to an average of 20 million reads per sample. All raw sequence data were quality trimmed for adaptor sequences, and reads shorter than 50 bp were discarded using Fastp v 0.20.1. Then, all reads were aligned to the mm10 mouse genome using Bowtie2 version 2.4.2 with the following parameters: –very‐sensitive‐local–no‐unal–no‐mixed–no‐discordant–phred33 ‐I 10 ‐X 700. Reads were then normalized by calculating the reads per kilobase of transcript per million mapped reads (RPKM). Peak calling used macs2 v 2.2.7.1 with the following parameters: ‐B ‐t –broad ‐g mm –broad‐cutoff 0.1. Scatterplots, correlation plots, and heatmaps are displayed using deepTools v3.5.0. Annotation of peaks was performed using the R/Bioconductor package ChIPseeker.

### Data Availability 

Datasets for this study have been deposited into the GEO database (http://www.ncbi.nlm.nih.gov/geo) under accession identification numbers GSE173430, GSE176013, and GSE126302.

### Statistical Analysis

Statistical analyses were performed using GraphPad Software (Prism 9.0). All data are presented as the mean ± SD (in vitro), ± SEM (in vivo) or mean with range. The values were defined as outliers (Z‐score>3) and removed from the data. Unless otherwise stated, one‐way or two‐way ANOVA together with the indicated post‐hoc test was used to determine statistical significance among multiple groups. Unpaired Student's t test was used to determine statistical significance between two groups. Unless indicated, the results are from at least two or three independent experiments. In all analyses, a *P* value of <0.05 was considered to be statistically significant.

### Study Approval

All animal experiments were approved by the Animal Care and Use Committees at Ren Ji Hospital (RJ2020‐0505), Shanghai Jiao Tong University School of Medicine. The human pancreatic tumor microarray from Ren Ji Hospital was approved with Local Ethics Committee approval and patient consent (KY2022‐036‐B).

## Conflict of Interest

The authors declare no conflict of interest.

## Author Contributions

N.N., X.S., and L. Z. share the first author credit. J.X. and N.N. designed the experiment and interpreted the data. N.N., X.S, and L.Z. performed most of the experiments. Y.C., W.Y., P.L., J.S., D.X., Y.T., X.Y., Y.W., and M.L. assisted in some experiments. X.Z., and L.‐M.W. assisted in MRI. Y.S. provided the key materials and assisted in some discussion. J.X. and N.N. wrote the manuscript. J.X. and N.N. provided the overall guidance.

## Supporting information

Supporting Information

## Data Availability

The data that support the findings of this study are available from the corresponding author upon reasonable request.

## References

[1] a) H. Garner , K. E. de Visser , Nat. Rev. Immunol. 2020, 20, 483; 32024984 10.1038/s41577-019-0271-z

[2] G. Y. Liou , H. Doppler , B. Necela , B. Edenfield , L. Zhang , D. W. Dawson , P. Storz , Cancer Discov 2015, 5, 52. 25361845 10.1158/2159-8290.CD-14-0474 PMC4293204

[3] a) F. Li , Q. Huang , T. A. Luster , H. Hu , H. Zhang , W. L. Ng , A. Khodadadi‐Jamayran , W. Wang , T. Chen , J. Deng , M. Ranieri , Z. Fang , V. Pyon , C. M. Dowling , E. Bagdatlioglu , C. Almonte , K. Labbe , H. Silver , A. R. Rabin , K. Jani , A. Tsirigos , T. Papagiannakopoulos , P. S. Hammerman , V. Velcheti , G. J. Freeman , J. Qi , G. Miller , K. K. Wong , Cancer Discov 2020, 10, 270; 31744829 10.1158/2159-8290.CD-19-0780 PMC7007372

[4] a) L. G. Ng , R. Ostuni , A. Hidalgo , Nat. Rev. Immunol. 2019, 19, 255; 30816340 10.1038/s41577-019-0141-8

[5] V. Cortez‐Retamozo , M. Etzrodt , A. Newton , P. J. Rauch , A. Chudnovskiy , C. Berger , R. J. Ryan , Y. Iwamoto , B. Marinelli , R. Gorbatov , R. Forghani , T. I. Novobrantseva , V. Koteliansky , J. L. Figueiredo , J. W. Chen , D. G. Anderson , M. Nahrendorf , F. K. Swirski , R. Weissleder , M. J. Pittet , Proc. Natl. Acad. Sci. USA 2012, 109, 2491. 22308361 10.1073/pnas.1113744109 PMC3289379

[6] a) A. Takeda , M. Hollmen , D. Dermadi , J. Pan , K. F. Brulois , R. Kaukonen , T. Lonnberg , P. Bostrom , I. Koskivuo , H. Irjala , M. Miyasaka , M. Salmi , E. C. Butcher , S. Jalkanen , Immunity 2019, 51, 561; 31402260 10.1016/j.immuni.2019.06.027

[7] a) T. T. Wang , Y. L. Zhao , L. S. Peng , N. Chen , W. Chen , Y. P. Lv , F. Y. Mao , J. Y. Zhang , P. Cheng , Y. S. Teng , X. L. Fu , P. W. Yu , G. Guo , P. Luo , Y. Zhuang , Q. M. Zou , Gut 2017, 66, 1900; 28274999 10.1136/gutjnl-2016-313075 PMC5739867

[8] a) N. Niu , P. Lu , Y. Yang , R. He , L. Zhang , J. Shi , J. Wu , M. Yang , Z. G. Zhang , L. W. Wang , W. Q. Gao , A. Habtezion , G. G. Xiao , Y. Sun , L. Li , J. Xue , Gut 2020, 69, 715; 31300513 10.1136/gutjnl-2019-318362

[9] A. M. Newman , C. B. Steen , C. L. Liu , A. J. Gentles , A. A. Chaudhuri , F. Scherer , M. S. Khodadoust , M. S. Esfahani , B. A. Luca , D. Steiner , M. Diehn , A. A. Alizadeh , Nat. Biotechnol. 2019, 37, 773. 31061481 10.1038/s41587-019-0114-2 PMC6610714

[10] S. L. Highfill , Y. Cui , A. J. Giles , J. P. Smith , H. Zhang , E. Morse , R. N. Kaplan , C. L. Mackall , Sci. Transl. Med. 2014, 6, 237ra67. 10.1126/scitranslmed.3007974 PMC6980372 24848257

[11] F. Veglia , V. A. Tyurin , M. Blasi , A. De Leo , A. V. Kossenkov , L. Donthireddy , T. K. J. To , Z. Schug , S. Basu , F. Wang , E. Ricciotti , C. DiRusso , M. E. Murphy , R. H. Vonderheide , P. M. Lieberman , C. Mulligan , B. Nam , N. Hockstein , G. Masters , M. Guarino , C. Lin , Y. Nefedova , P. Black , V. E. Kagan , D. I. Gabrilovich , Nature 2019, 569, 73. 30996346 10.1038/s41586-019-1118-2 PMC6557120

[12] a) A. C. Mihaila , L. Ciortan , R. D. Macarie , M. Vadana , S. Cecoltan , M. B. Preda , A. Hudita , A. M. Gan , G. Jakobsson , M. M. Tucureanu , E. Barbu , S. Balanescu , M. Simionescu , A. Schiopu , E. Butoi , Front Immunol 2021, 12, 708770; 34447377 10.3389/fimmu.2021.708770 PMC8384118

[13] X. G. Zhu , A. Chudnovskiy , L. Baudrier , B. Prizer , Y. Liu , B. N. Ostendorf , N. Yamaguchi , A. Arab , B. Tavora , R. Timson , S. Heissel , E. de Stanchina , H. Molina , G. D. Victora , H. Goodarzi , K. Birsoy , Cell Metab. 2021, 33, 211. 33152324 10.1016/j.cmet.2020.10.017 PMC7790894

[14] S. H. Gohil , J. B. Iorgulescu , D. A. Braun , D. B. Keskin , K. J. Livak , Nat. Rev. Clin. Oncol. 2021, 18, 244. 33277626 10.1038/s41571-020-00449-x PMC8415132

[15] a) H. M. Lo , T. H. Lai , C. H. Li , W. B. Wu , Acta Pharmacol. Sin. 2014, 35, 339; 24487964 10.1038/aps.2013.182 PMC4647895

[16] Q. Xu , Y. Xiang , Q. Wang , L. Wang , J. Brind'Amour , A. B. Bogutz , Y. Zhang , B. Zhang , G. Yu , W. Xia , Z. Du , C. Huang , J. Ma , H. Zheng , Y. Li , C. Liu , C. L. Walker , E. Jonasch , L. Lefebvre , M. Wu , M. C. Lorincz , W. Li , L. Li , W. Xie , Nat. Genet. 2019, 51, 844. 31040401 10.1038/s41588-019-0398-7

[17] a) V. P. Balachandran , G. L. Beatty , S. K. Dougan , Gastroenterology 2019, 156, 2056; 30660727 10.1053/j.gastro.2018.12.038 PMC6486864

[18] D. Siolas , E. Vucic , E. Kurz , C. Hajdu , D. Bar‐Sagi , Cell Rep. 2021, 36, 109578. 34433022 10.1016/j.celrep.2021.109578 PMC8687588

[19] Z. Ji , Y. Sheng , J. Miao , X. Li , H. Zhao , J. Wang , C. Cheng , X. Wang , K. Liu , K. Zhang , L. Xu , J. Yao , L. Shen , J. Hou , W. Zhou , J. Sun , L. Li , W. Q. Gao , H. H. Zhu , Nat. Commun. 2019, 10, 3353. 31350389 10.1038/s41467-019-11282-x PMC6659703

[20] S. X. Pfister , E. Markkanen , Y. Jiang , S. Sarkar , M. Woodcock , G. Orlando , I. Mavrommati , C. C. Pai , L. P. Zalmas , N. Drobnitzky , G. L. Dianov , C. Verrill , V. M. Macaulay , S. Ying , N. B. La Thangue , V. D'Angiolella , A. J. Ryan , T. C. Humphrey , Cancer Cell 2015, 28, 557. 26602815 10.1016/j.ccell.2015.09.015 PMC4643307

[21] J. Li , K. T. Byrne , F. Yan , T. Yamazoe , Z. Chen , T. Baslan , L. P. Richman , J. H. Lin , Y. H. Sun , A. J. Rech , D. Balli , C. A. Hay , Y. Sela , A. J. Merrell , S. M. Liudahl , N. Gordon , R. J. Norgard , S. Yuan , S. Yu , T. Chao , S. Ye , T. S. K. Eisinger‐Mathason , R. B. Faryabi , J. W. Tobias , S. W. Lowe , L. M. Coussens , E. J. Wherry , R. H. Vonderheide , B. Z. Stanger , Immunity 2018, 49, 178. 29958801 10.1016/j.immuni.2018.06.006 PMC6707727

[22] J. Mallen‐St Clair , R. Soydaner‐Azeloglu , K. E. Lee , L. Taylor , A. Livanos , Y. Pylayeva‐Gupta , G. Miller , R. Margueron , D. Reinberg , D. Bar‐Sagi , Genes Dev. 2012, 26, 439. 22391448 10.1101/gad.181800.111 PMC3305982

[23] I. Ischenko , S. D'Amico , M. Rao , J. Li , M. J. Hayman , S. Powers , O. Petrenko , N. C. Reich , Nat. Commun. 2021, 12, 1482. 33674596 10.1038/s41467-021-21736-w PMC7935870

[24] S. R. Nielsen , J. E. Strobech , E. R. Horton , R. Jackstadt , A. Laitala , M. C. Bravo , G. Maltese , A. R. D. Jensen , R. Reuten , M. Rafaeva , S. A. Karim , C. I. Hwang , L. Arnes , D. A. Tuveson , O. J. Sansom , J. P. Morton , J. T. Erler , Nat. Commun. 2021, 12, 3414. 34099731 10.1038/s41467-021-23731-7 PMC8184753

