# Supplementary material for: Tumor Cell‐Intrinsic SETD2 Deficiency Reprograms Neutrophils to Foster Immune Escape in Pancreatic Tumorigenesis
Source: Adv Sci (Weinh). 2022 Dec 1;10(2):2202937. doi: 10.1002/advs.202202937 (PMC9839845; doi:10.1002/advs.202202937)
Supplement: Supplementary file 1 — Supporting Information [file ADVS-10-2202937-s001.pdf]

ADVANCED SCIENCE
Open Access

Supporting Information

for *Adv. Sci.*, DOI 10.1002/advs.202202937

Tumor Cell-Intrinsic SETD2 Deficiency Reprograms Neutrophils to Foster Immune Escape in Pancreatic Tumorigenesis

Ningning Niu, Xuqing Shen, Li Zhang, Yueyue Chen, Ping Lu, Wenjuan Yang, Mingzhu Liu, Juanjuan Shi, Dapeng Xu, Yingying Tang, Xiaotong Yang, Yawen Weng, Xinxin Zhao, Lian-Ming Wu, Yongwei Sun and Jing Xue**

Supporting Information

Figures S1-7

Supporting Experimental Section

Tables S1-8

Figures S1-7

Figure S1.

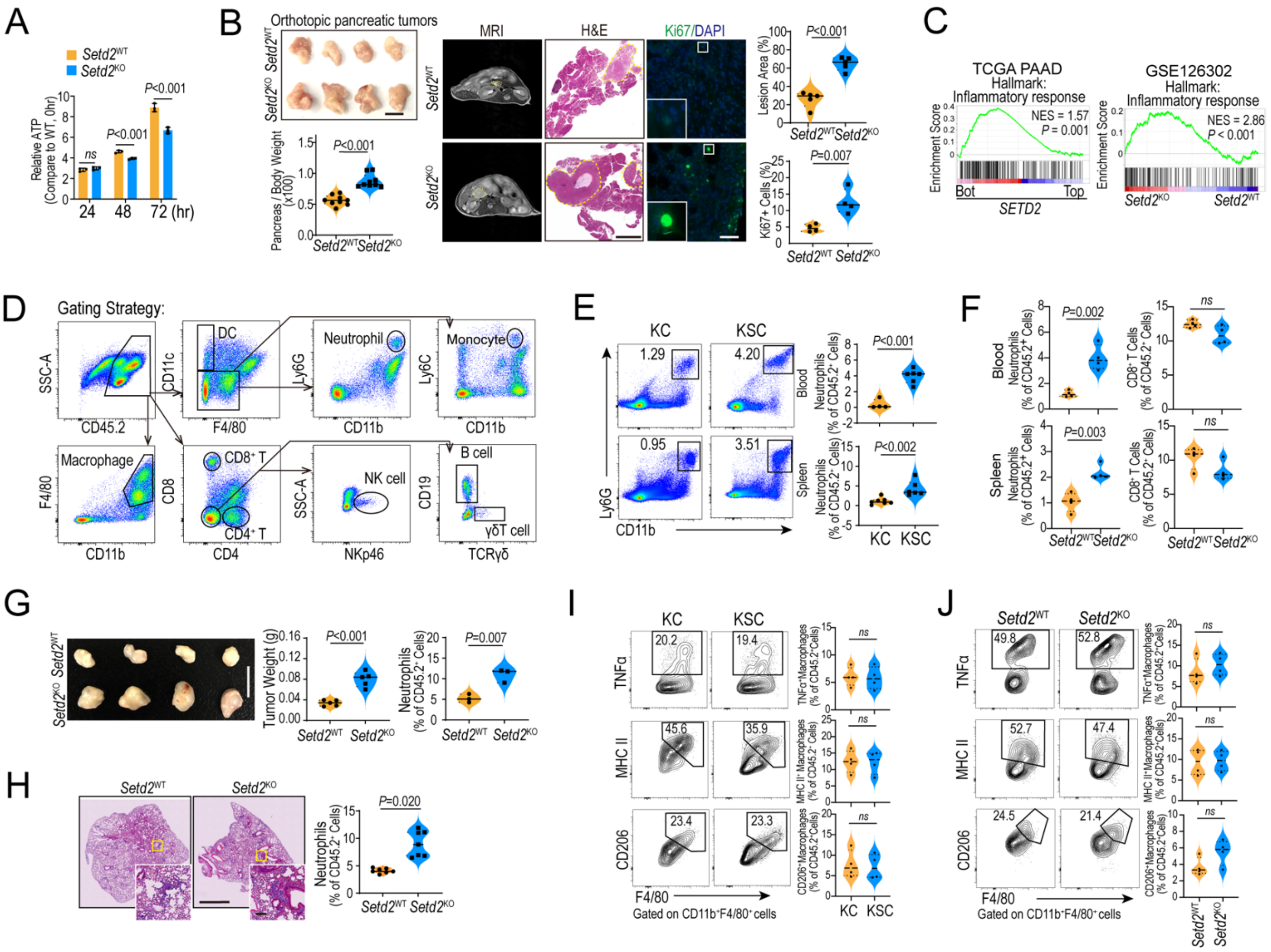

Figure S1. *Setd2* deficiency leads to neutrophil accumulation in GEMM, pancreatic orthotopic, subcutaneous, and lung metastasis models.

(A) Cell proliferation assay of *Setd2*^{WT} and *Setd2*^{KO} cells at the indicated days by ATP levels.

(B) Orthotopic pancreatic tumors from *Setd2*^{WT} and *Setd2*^{KO} cells (n = 9, scale bar = 1 cm). Pancreatic tissues were collected, and the relative pancreas weight is shown. Representative MRI and H&E staining of pancreatic tissues and the percentage of lesion area from the indicated group are shown (The yellow dashed line identifies the tumor growth on the pancreas, scale bar = 2 mm). Representative image and quantification of Ki67^{+} (green) cells in orthotopic pancreatic tumor models of *Setd2*^{WT}

and *Setd2*^{KO} cells. Scale bar = 100 μm.

(C) Gene set enrichment analysis (GSEA) of gene expression between low- versus high-*SETD2*-expressing cases (18 cases, 10%, respectively) from the TCGA-PAAD database (left) and *in vitro* cultured *Setd2*^{WT} versus *Setd2*^{KO} cells (GSE126302, right).

(D) Representative gating strategy of polychromatic flow cytometry analyses in figure 1B.

(E-F) Representative FACS plots and percent of neutrophils among total CD45.2^{+} cells in the blood and spleen of the indicated mice (n =4-6 per group).

(G) Subcutaneous tumors were collected and weighed at the indicated times. The percent of neutrophils among total CD45.2^{+} cells in tumors from the indicated mice (n =6 per group, scale bar = 1 cm).

(H) Representative images of lung metastases and percentage of neutrophils of total CD45.2^{+} cells from lung tissues (n = 7 per group, scale bars = 1 mm and 100 μm for insets).

(I-J) Representative FACS plots and the percent of tumor-infiltrating TNFα^{+}, MHCII^{+}, and CD206^{+} macrophages (% of total CD45.2^{+} cells) in the indicated pancreatic tissues (n =4 per group).

Data are represented as the mean ± SD (A) or mean ± SEM (B, E-J). All significant differences were determined with an unpaired Student's t test.

Figure S2.

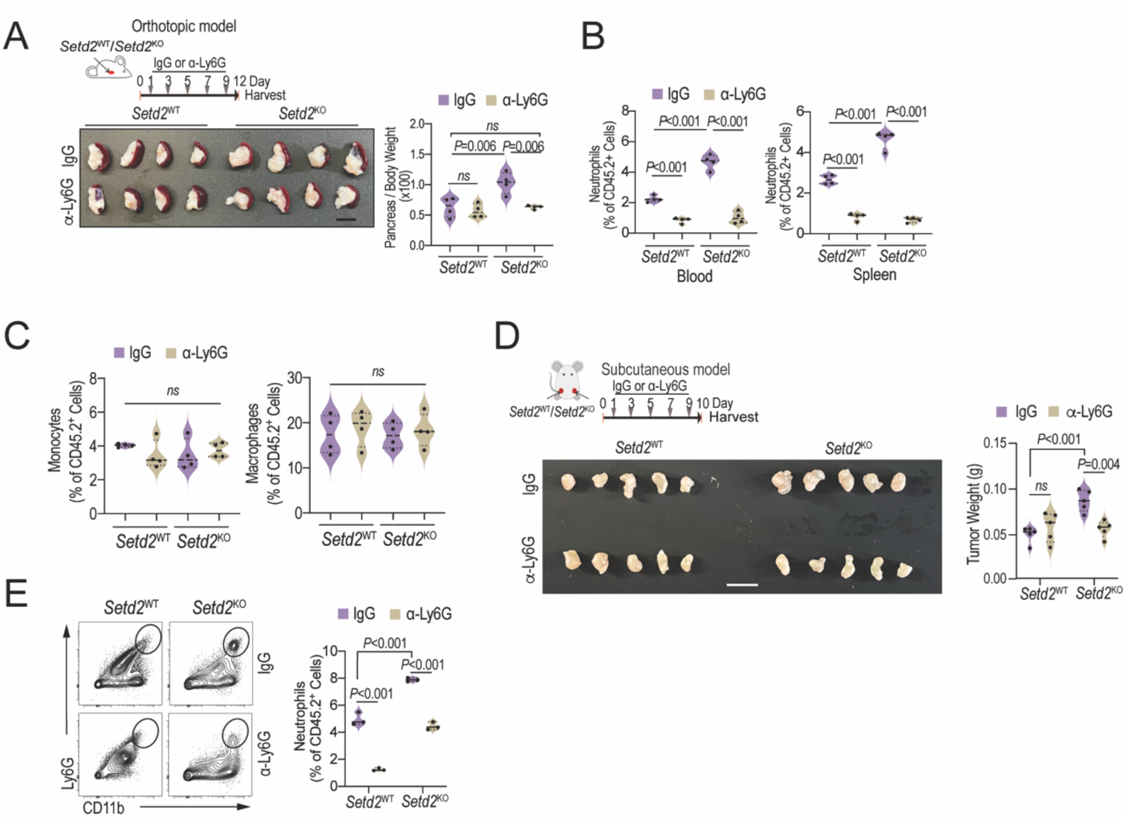

Figure S2. Neutrophil depletion impaired the progression of *Setd2*-deficient pancreatic orthotopic tumors.

(A) Mice bearing *Setd2*^{WT} and *Setd2*^{KO} pancreatic tumors (orthotopic model) were administered IgG and Ly6G neutralizing antibodies (1 mg/kg every other day) for 12 days. Pancreatic tissues were collected for quantification (n = 4, scale bar = 1 cm).

(B) The percent of neutrophils among total CD45.2^{+} cells in the blood and spleen of the indicated mice (n =4 per group).

(C) The percent of tumor-infiltrating monocytes and macrophages (% of total CD45.2^{+} cells) in the indicated pancreas tissues (n =4 per group).

(D) Mice bearing *Setd2*^{WT} and *Setd2*^{KO} tumors (subcutaneous model) were administered IgG and Ly6G neutralizing antibodies (1 mg/kg, every other day) for 10 days. Tumors were collected for quantification (n = 5, scale bar = 1 cm).

(E) Representative plots and the percentage of tumor-infiltrating neutrophils in tumors from the indicated groups (D).

Data are represented as the mean ± SEM. All statistical differences were determined with one-way ANOVA and Tukey's multiple comparisons test.

Figure S3.

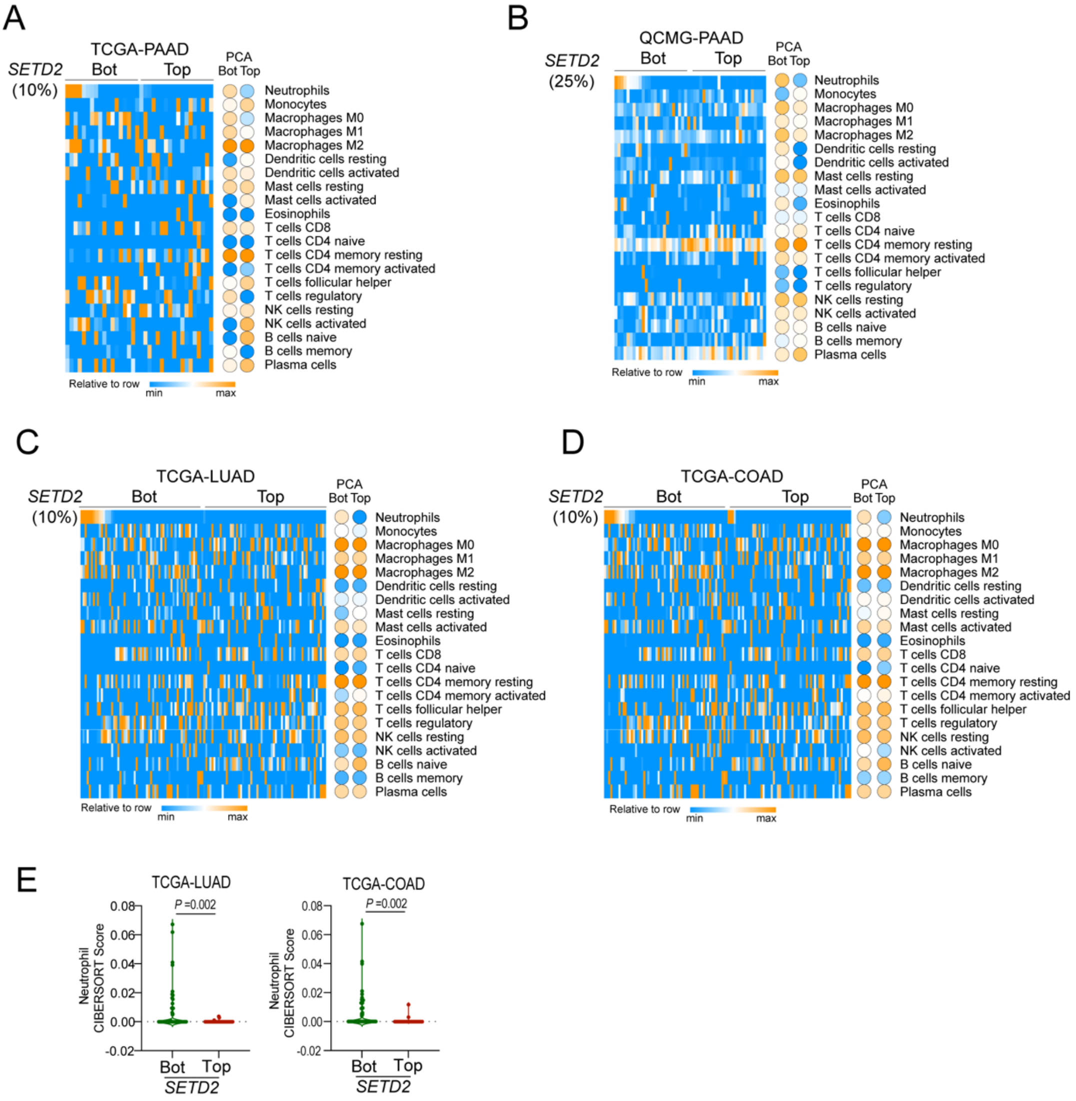

Figure S3. CIBERSORTx analysis to estimate the abundance of tumor-infiltrating immune cells in the TCGA database.

(A-D) Heatmap of CIBERSORTx analysis of tumor-infiltrating immune cells in the TCGA-PAAD (A), QCMG-PAAD (B), TCGA-LUAD (C) and TCGA-COAD (D) databases.

(E) CIBERSORTx analyses to estimate the abundance of tumor-infiltrating neutrophils in the database as indicated.

Figure S4.

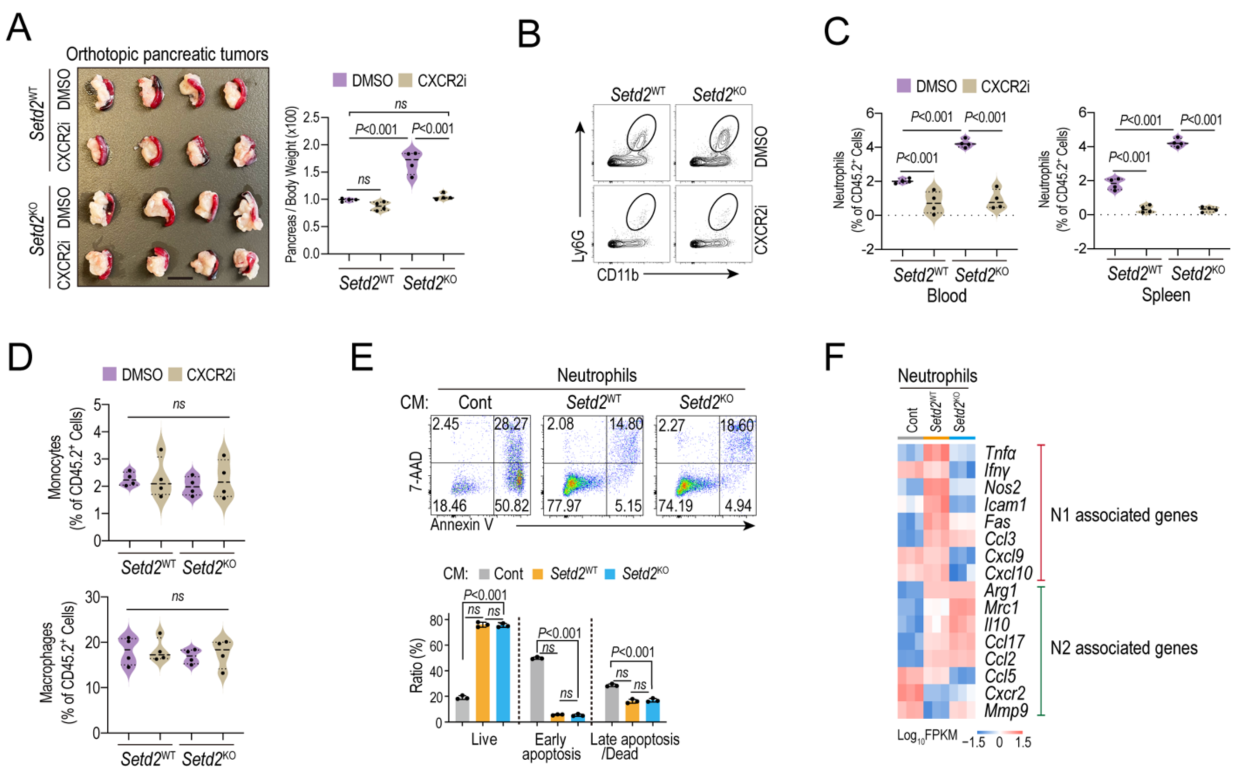

Figure S4. Neutrophils are recruited and reprogrammed by *Setd2*^{KO} pancreatic tumors.

(A) Mice bearing orthotopic *Setd2*^{WT} and *Setd2*^{KO} tumors were administered DMSO and CXCR2i. Pancreatic tissues from the indicated groups were collected for quantification (n =4 per group, scale bar =1 cm).

(B) Representative FACS plots of neutrophils from total CD45.2^{+} cells in pancreatic tissue.

(C) The percent of neutrophils among total CD45.2^{+} cells in the blood and spleen of the indicated mice (n =4 per group).

(D) The percent of tumor-infiltrating monocytes and macrophages (% of total CD45.2^{+} cells) in the indicated pancreas tissues (n =4 per group).

(E) Cell viability was determined by annexin V and 7-AAD staining. The frequency of live, early apoptotic and late apoptotic/dead cells from the indicated groups.

(F) Heatmap of RNA-Seq data comparing the expression of N1- and N2-associated genes [1] among the Neu-Cont, Neu-*Setd2*^{WT} and Neu-*Setd2*^{KO} groups. Data are shown as heat map with a color scale. Each square represents an individual

independent data point.

Data are represented as the mean ± SEM (A, C-D) or mean ± SD (E). All statistical differences were determined with one-way ANOVA and Tukey's multiple comparisons test.

Figure S5.

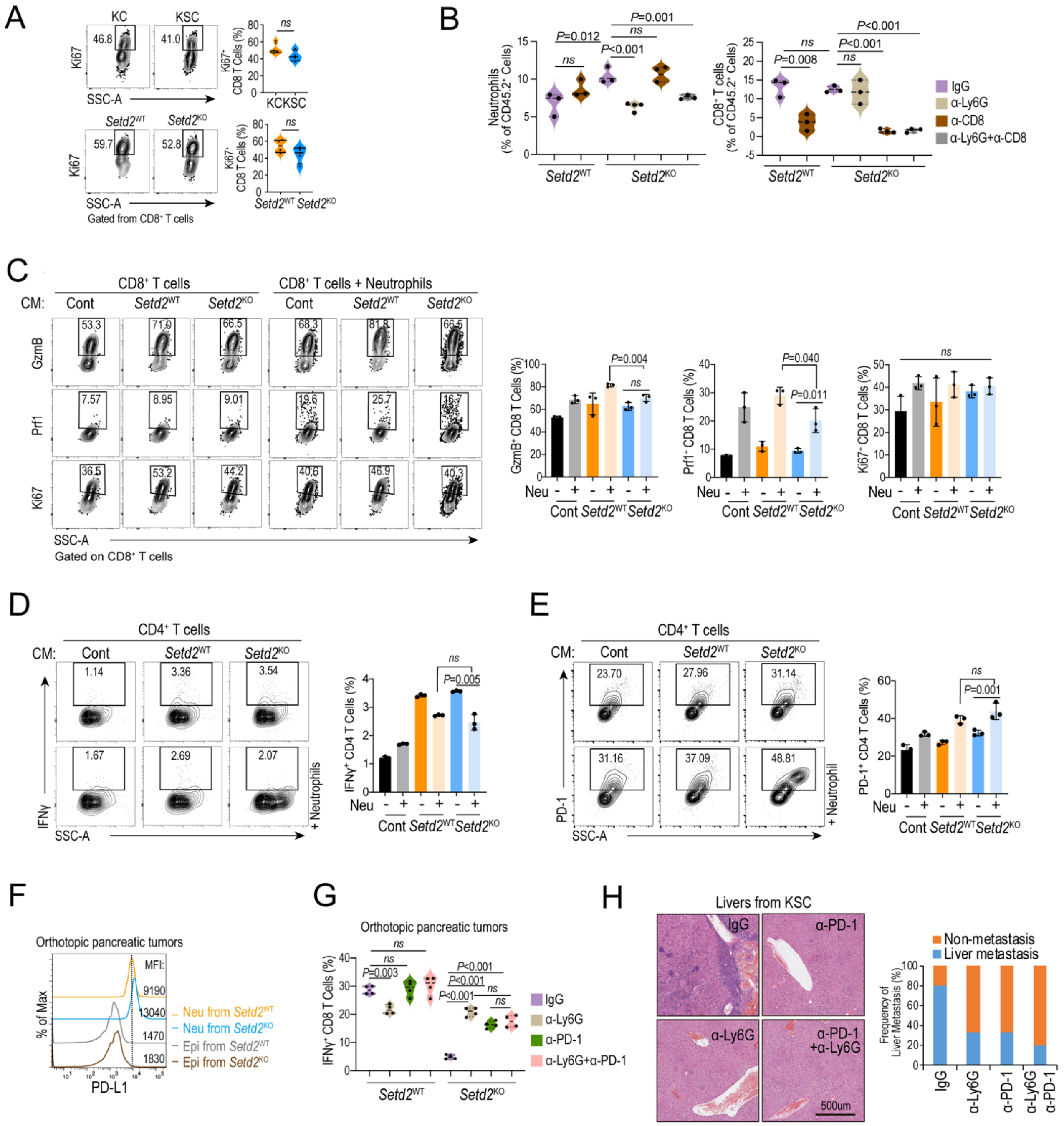

Figure S5. Functional analysis of neutrophils primed by conditioned medium.

(A) Representative plots and statistical analysis of Ki67 levels in tumor-infiltrating CD8$^+$ T cells from the indicated mice (n =4 per group).

(B) The percentage of tumor-infiltrating neutrophils and CD8$^+$ T cells in tumors from the indicated groups.

(C) Representative plots and statistical analysis of GzmB, Prf1 and Ki67 production by $CD8^+$ T cells from the indicated coculture group.

(D-E) Representative plots and statistical analysis of IFNγ expression (D) and surface PD-1 (E) by $CD4^+$ T cells from the indicated groups.

(F) PD-L1 levels on tumor cells and tumor-infiltrating neutrophils from the indicated mice.

(G) Representative flow cytometry plots of IFNγ production in tumor-infiltrating $CD8^+$ T cells from the indicated mice.

(H) Representative images and statistics of metastases and H&E staining of liver tissues of KSCs from the indicated groups (n = 5). Scale bars = 500 μm.

Data are represented as the mean ± SEM (A-B and G) or mean ± SD (C-E). Statistical differences were determined with unpaired Student’s t test in (A) and one-way ANOVA and Tukey's multiple comparisons test in (B-E and G).

Figure S6.

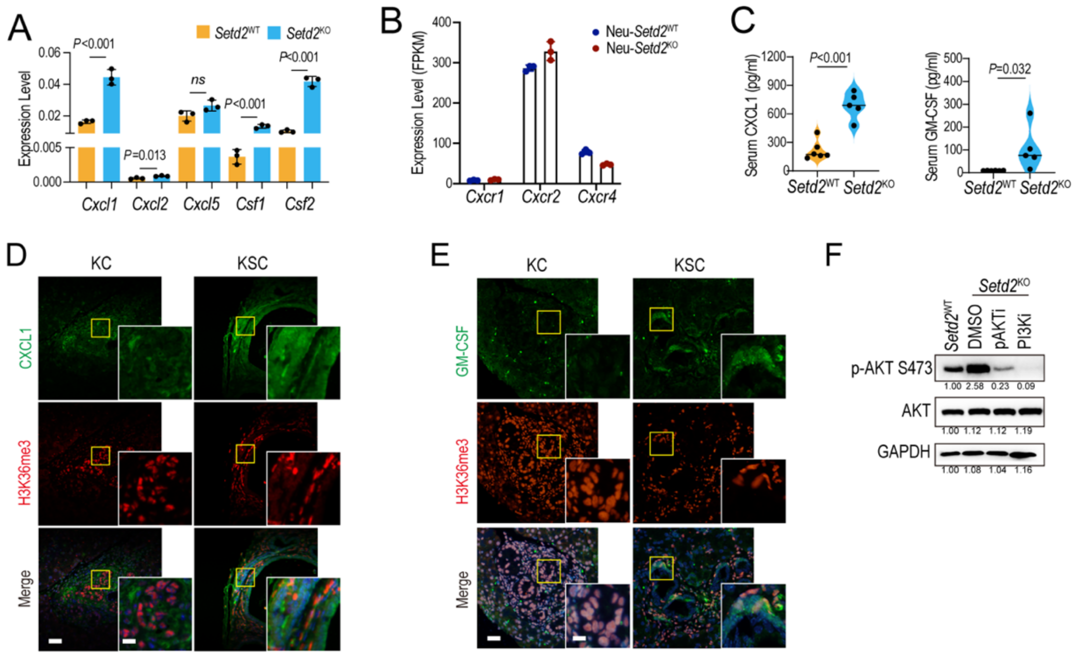

Figure S6. *Setd2*^{KO}-derived CXCL1 and GM-CSF can recruit and activate neutrophils.

(A) Expression levels of the indicated genes in *Setd2*^{WT} and *Setd2*^{KO} cells.

(B) The mRNA levels of *Cxcr1*, *Cxcr2* and *Cxcr4* in Neu-*Setd2*^{WT} and Neu-*Setd2*^{KO} cells measured by RNA-seq.

(C) ELISA analyses of serum CXCL1 (left) and GM-CSF (right) levels from the indicated orthotopic pancreatic tumor-bearing mice.

(D-E) Immunofluorescence staining of CXCL1, GM-CSF (green), H3K36me3 (red), and merged images (bottom) on pancreatic tissues from the indicated mice. Scale bars = 100 μm and 20 μm (for insets).

(F) *Setd2*^{KO} cells were treated with MK2206 (pAKTi), Ly294002 (PI3Ki) or DMSO as a control for 20 hr. Phosphorylation and total AKT levels were analyzed in the indicated cells by western blotting.

Data are represented as the mean ± SD (A-B) or mean ± SEM (C). Statistical differences were determined with unpaired Student's t test in (A and C).

Figure S7.

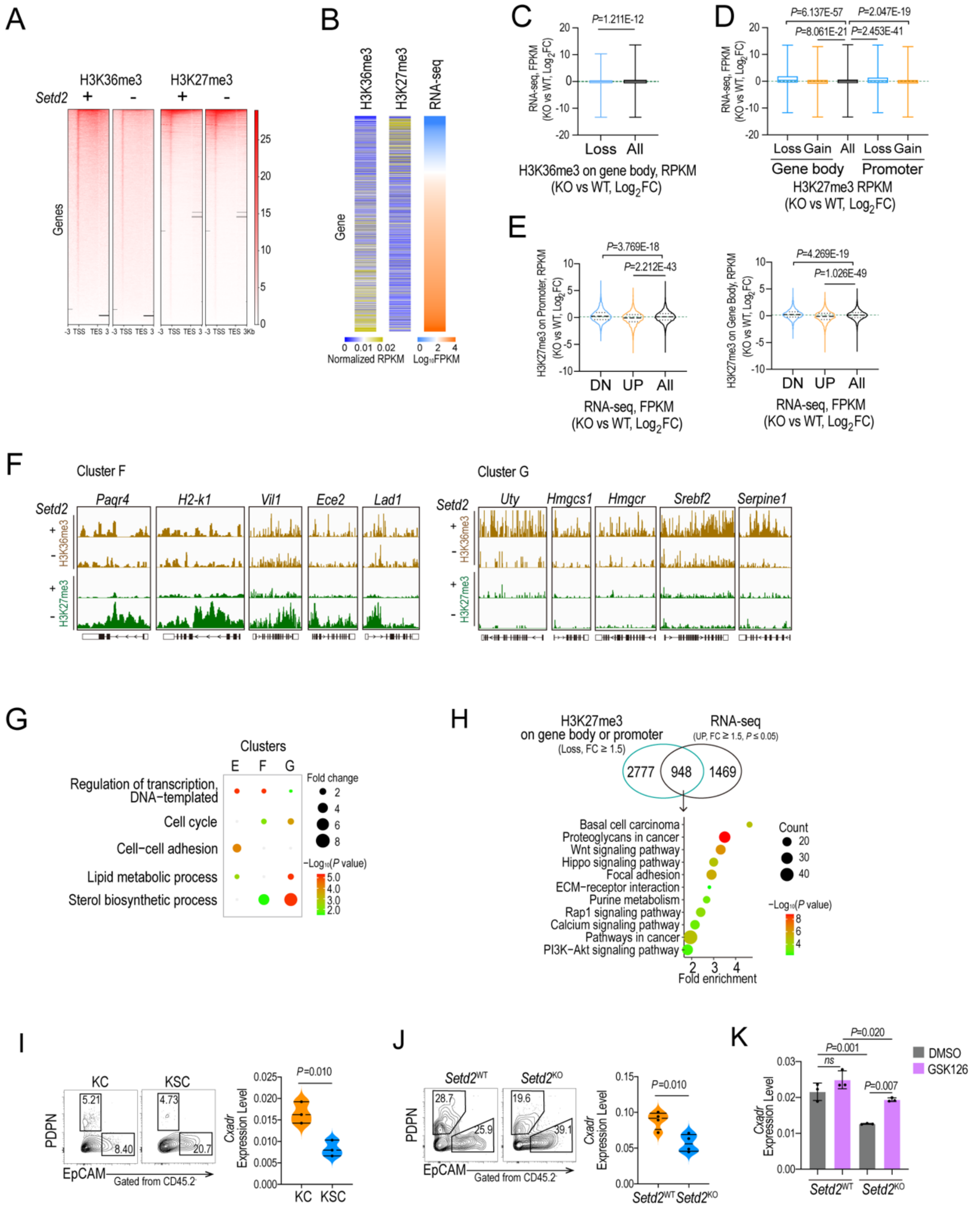

A
H3K36me3
H3K27me3
Setd2
Genes
B
H3K36me3
H3K27me3
RNA-seq
Gene
Normalized RPKM
Log10FPKM
C
P=1.211E-12
RNA-seq, FPKM (KO vs WT, Log2FC)
Loss
All
H3K36me3 on gene body, RPKM (KO vs WT, Log2FC)
D
P=6.137E-57
P=2.047E-19
P=8.061E-21
P=2.453E-41
Loss Gain All Loss Gain
Gene body
Promoter
H3K27me3 RPKM (KO vs WT, Log2FC)
E
P=3.769E-18
P=2.212E-43
H3K27me3 on Promoter, RPKM (KO vs WT, Log2FC)
DN UP All
RNA-seq, FPKM (KO vs WT, Log2FC)
P=4.269E-19
P=1.026E-49
H3K27me3 on Gene Body, RPKM (KO vs WT, Log2FC)
F
Cluster F
Paqr4
H2-k1
Vil1
Ece2
Lad1
Cluster G
Uty
Hmgcs1
Hmgcr
Srebf2
Serpine1
G
Clusters
E F G
Fold change
Regulation of transcription, DNA−templated
Cell cycle
Cell−cell adhesion
Lipid metabolic process
Sterol biosynthetic process
−Log10(P value)
H
H3K27me3 on gene body or promoter (Loss, FC ≥ 1.5)
RNA-seq (UP, FC ≥ 1.5, P ≤ 0.05)
2777
948
1469
Basal cell carcinoma
Proteoglycans in cancer
Wnt signaling pathway
Hippo signaling pathway
Focal adhesion
ECM−receptor interaction
Purine metabolism
Rap1 signaling pathway
Calcium signaling pathway
Pathways in cancer
PI3K−Akt signaling pathway
Count
Fold enrichment
I
KC
KSC
PDPN
EpCAM
Gated from CD45.2
P=0.010
Cxadr Expression Level
KC KSC
J
Setd2WT
Setd2KO
PDPN
EpCAM
Gated from CD45.2
P=0.010
Cxadr Expression Level
K
P=0.020
P=0.001
ns
P=0.007
DMSO
GSK126
Cxadr Expression Level
Setd2WT
Setd2KO

Figure S7. CUT&Tag analysis of H3K36me3 and H3K27me3 redistribution affected by *Setd2* loss.

(A) Heatmap of CUT&Tag signals upstream and downstream of the gene body. Scale regions were from 3,000 bp upstream of transcription start sites (TSS) to downstream of the transcription end site (TES). Length was plotted by using plotHeatmap tools in deepTools.

(B) Heatmap illustrating the occupation of H3K36me3 and H3K27me3, as well as the expression of genes in *Setd2*^{WT} pancreatic tumor cells.

(C-D) Box plot showing normalized transcriptional alternation of the indicated genes.

(E) Violin plot showing alterations in the normalized H3K27me3 distribution of the indicated genes.

(F) Representative IGV screenshot of CUT&Tag signals for the indicated genes from clusters F (left) and G (right).

(G) GO enrichment analysis of genes from the indicated clusters.

(H) Venn diagram illustration of H3K27me3-gain genes in *Setd2*^{KO} pancreatic tumor cells and the overlap with upregulated genes determined by RNA sequencing (upper). KEGG pathway enrichment of the 948 overlapping genes (lower).

(I-J) Expression level of *Cxadr* in tumor cells isolated from the pancreas of KC and KSC mice (GEMM model, I) and *Setd2*^{WT} and *Setd2*^{KO} pancreatic tumor-bearing mice (orthotopic model, J).

(K) *Setd2*^{WT} and *Setd2*^{KO} pancreatic tumor cells were treated with GSK126 (inhibitor of EZH2, 5 μM) or DMSO as a control for 24 hrs. The mRNA levels of *Cxadr* were determined with qPCR assay.

Data in (C-E) are represented as the mean with range, mean ± SEM (I and J), and mean ± SD (K). Statistical differences were determined with unpaired Student’s t test in (C-E and I-J) and one-way ANOVA and Tukey's multiple comparisons test in (K).

Supporting Experimental Section

Cell preparation and culture conditions:

Setd2^{KO} cells were constructed using the CRISPR/Cas9 gene-editing system in the murine PDAC cell line KPC1199 which was generated from KPC (*Pdx*^{cre}; LSL- *Kras*^{G12D}; LSL-*TP53*^{R172H}) mice as described previously [2]. Leukocytes of pancreatic tissues and subcutaneous tumors were isolated using the collagenase digestion method described for flow cytometry analysis [3]. In brief, pancreatic tumor tissue was cut into pieces and digested in FACS buffer (HBSS +10% FCS) containing 2 mg/ml collagenase type IV (40510ES60, Yeasen Biotech). The tissues were incubated in a 37°C water bath for 20 min, vortexed every 10 min, and then passed through a 70 μm filter. Bone marrow (BM) was flushed with a syringe filled with FACS buffer (HBSS + 10% FCS) to obtain BM cells. Primary neutrophils were sorted from BM cells with CD45.2$^+$, CD11b$^+$, and Ly6G$^+$ staining. Splenocytes were acquired by simply smashing the spleen and then washed with FACS buffer (HBSS + 2% NCS). Primary CD8$^+$ T cells were sorted from splenocytes with CD45.2$^+$, CD4$^-$, and CD8$^+$ antibodies. *Setd2*^{WT} and *Setd2*^{KO} cell lines were established as described (*Setd2*^{KO} for *Setd2*^{KO2})[2b]. Primary neutrophils were treated with or without 20% CM, M-CSF (Peprotech, 315-02, 50 ng/ml), or GM-CSF (Peprotech, 315-03, 50 ng/ml) for 24 hours. Primary CD8$^+$ T cells were cultured with 20% CM from *Setd2*^{WT} and *Setd2*^{KO} cell lines with or without primary neutrophils for 24 hours. Twenty percent CM from *Setd2*^{KO} cells was neutralized with α-GM-CSF (10 ng/ml, 505401, clone-MP1-22F9, Biolegend) or control IgG for 6 hours and used to culture primary neutrophils and CD8$^+$ T cells. CXCR2i, SB225002 (200 nM, S7651, Selleck), α-PD-L1 (10 ng/ml, 124318, clone-10F.9G2, Biolegend), α-PD-1 (10 ng/ml, 135247, clone-29F.1A12, Biolegend) and control IgG were administered as indicated. LY294002 (PI3Ki, 500 nM, S1105, Selleck), MK2206 (pAKTi, 500 nM, S1078, Selleck), and GSK126 (EZH2 inhibitor, 5 μM, S7061, Selleck) were used to treat tumor cells for 24 hr.

Histology:

Pancreatic tissues and subcutaneous tumors were fixed in 10% formalin overnight and then sectioned and stained with H&E and immunofluorescent staining (performed by Ruiyu Inc.; Shanghai, China). The lesion area of pancreatic tissues was calculated by ImageJ software as follows: pancreatic paraffin blocks were successively sectioned at 4 μm intervals. The 1st, 11th and 21st sections were stained with H&E, and the lesion areas were measured by ImageJ. The mean lesion area was calculated to represent the lesion area for each sample.

Western Blot and Antibodies:

Pancreatic tissues were fixed in 10% formalin and then sectioned for immunocytochemistry staining. Tissues and cells were lysed in lysis buffer (60 mM pH 6.8 Tris-HCl, 25% glycerol, 2% SDS, 5% β-mercaptoethanol, and 19% (vol/vol) in ddH_2O) supplemented with PMSF (1 mM) and protease inhibitor cocktail (HY-K0010, MedChem Express; Shanghai, China). p-Akt S473 (4060S), AKT (2920S) and E-cadherin (3195P) were purchased from CST. MPO (ab208670), CD8 (Ab25478), H3K36me3 (Ab9050), Histone H3 (Ab1791), H3K27me3 (Ab6002), H3K27Ac (Ab4729), H3K4me3 (Ab3099), H3K9me3 (Ab8898), and H3K36me2 (Ab9049) were purchased from Abcam. CK19 (10712-1-AP), CXCL1 (12335-1-AP) and GAPDH (HRP-60004) were purchased from Proteintech. CXADR (AF2654) was purchased from R&D Systems.

ELISA:

Serum collected from mice carrying orthotopic pancreatic tumors and cultured medium from *Setd2*^{WT} and *Setd2*^{KO} cell lines were used to determine the levels of CXCL1 (KE10019, Proteintech) and GM-CSF (MGM00, R&D Systems) according to the manufacturer’s instructions.

RNA-seq and analysis:

For RNA preparation, neutrophils were sorted from BM cells, freshly treated with 20%

CM from *Setd2*^{WT} or *Setd2*^{KO} cells, and harvested after 24 hours. The RNA-seq transcriptome library was prepared following the TruSeq™ RNA sample preparation kit (Illumina) using 5 μg of total RNA, according to the manufacturer's instructions. The RNA-seq library was sequenced to an average of 20 million reads per sample. The raw paired-end reads were trimmed and quality controlled, and then clean reads were separately aligned to the reference genome with orientation mode using TopHat (version 2.0.0). To identify DEGs between two different groups, the expression level of each transcript was calculated according to the FPKM (fragments per kilobase per million). RSEM was used to quantify gene abundances. The R statistical package software EdgeR (Empirical analysis of Digital Gene Expression in R) was utilized for DEG analysis. In addition, functional enrichment analyses, including GO and KEGG, were performed to identify which DEGs were significantly enriched in GO terms and metabolic pathways at a Bonferroni corrected P value ≤0.05 compared with the whole transcriptome background.

Transfection of siCxadr and overexpression of Cxadr:

Setd2^{KO} cells were seeded into 6-well plates at 5 x 10^5 per well in complete cell culture medium. After 24 hr, cells were transfected with si*Cxadr* or plasmids expressing *Cxadr*. Forty-eight hours later, the cells were collected and analyzed by western blotting. The si*Cxadr sequence* was ordered from GenePharma: *Cxadr* (si*Cxadr* -1: 5'- GAGCAUCACUACACCCGAATT -3'; si*Cxadr* -2: 5'- GGAGCUAUUGGCAGCAATT -3).

Transwell-based chemotaxis assay and apoptosis assay:

Neutrophil chemotaxis assays were performed in 24-well plates containing transwell inserts with 3.0 μm pores according to the manufacturer's instructions. BM cells (1 x 10^6 in 100 μL) in serum-free RPMI 1640 were seeded into the upper chambers, while boiled or untreated cultured medium from *Setd2*^{WT} and *Setd2*^{KO} cells was added to the lower chambers. After 1.5 hr of incubation, cells that migrated through the membrane were collected for phenotypic analysis by flow cytometry. Apoptosis assays were

performed with an Assay Kit (KGA1025, KeyGEN BioTECH Corp., Ltd.). Briefly, neutrophils were cultured with 20% CM from *Setd2*^{WT} or *Setd2*^{KO} cells for 24 hr. Then, harvested cells were stained with Annexin V and 7-AAD and analyzed by flow cytometry according to the manufacturer's instructions.

Cytotoxicity of CD8^{+} T cells:

Setd2^{WT} and *Setd2*^{KO} cells were cultured with primary CD8^{+} T cells with or without primary neutrophils for 24 hours and alone as a control. After removal of immune cells, pancreatic tumor cells were washed with 1x PBS and starved for 4 hrs [4]. Next, *Setd2*^{WT} and *Setd2*^{KO} cells were collected and stained with DAPI. Live cells were quantified by using flow cytometry.

Flow cytometry:

For cell surface staining, single-cell suspensions were incubated with the antibody for 30 min at 4°C. For intracellular cytokine staining, immediately after isolation, cells were cultured in RPMI complete medium and stimulated with PMA (10 ng/ml; Yeasen), ionomycin (1 μg/ml; Yeasen) and brefeldin A (10 μg/ml; BioLegend) for 4 hr. The cells were washed and stained with surface markers and then fixed and permeabilized using a Foxp3 Transcription Factor Staining Buffer Set (2271994, Invitrogen) followed by cytokine antibody incubation for 30 min at 4°C. Antibodies were from BioLegend unless indicated: AF488-, AF700-, APC-, APC/Cyanine7-, and Brilliant Violet 510™-conjugated CD45.2 (104); PerCP/Cyanine5.5-, APC/Cyanine7-, PE/Cyanine7-, and Brilliant Violet 510™-conjugated CD4 (RM4-5); PE/Cyanine7-conjugated CD8 (5.3-6.7); Brilliant Violet 421™-conjugated TCR γ/δ (GL3); APC/Cyanine7-conjugated NK1.1; Brilliant Violet 421™-conjugated NKp64 (29A1.4); FITC- and Brilliant Violet 421™-conjugated PD-1 (29F.1A12); PE/Cyanine7- and Brilliant Violet 650™-conjugated PD-L1 (10F.9G2); FITC-conjugated CD19 (6D5); PE/Cyanine7- and Brilliant Violet 510™-conjugated CD3 (17A2); Percp5.5-conjugated CD11b (M1/70); PE- and Brilliant Violet 421™-conjugated F4/80 (BM8); AF647-conjugated CD206 (C068C2), AF700-conjugated MHC II (M5/114.15.2); APC/Cyanine7-conjugated

CD11c (N418); AF488- and PB-conjugated Ly6C (HK1.4); AF488-, PE-, APC-, and PE/Cyanine7-conjugated Ly6G (1A8); APC-conjugated Pdpn (8.1.1); PE/Cyanine7-conjugated Epcam (G8.8); FITC-conjugated Ki67 (11F6); PE-conjugated GzmB (QA16A02); PE-conjugated TNF-α (MP6-XT22); AF700-conjugated INF-γ (XMG1.2; BD Biosciences), and APC-cy7-conjugated Prf1 (17-9392-80, BD Biosciences). Data were obtained on a Fortessa LSRII (BD Biosciences) and analyzed using FlowJo software (Tree Star).

Quantitative RT–PCR:

RNA was extracted using TRIzol Reagent (Invitrogen) followed by reverse transcription using a HighCapacity cDNA RT Kit (Invitrogen) according to the manufacturer's instructions. cDNA was amplified by RT–PCR with SYBR Green (Roche), and the results were normalized to the housekeeping gene *Gapdh* (ΔCt). The sequences of the primers are listed in **Table S8**.

References

[1] a) H. Peng, J. Shen, X. Long, X. Zhou, J. Zhang, X. Xu, T. Huang, H. Xu, S. Sun, C. Li, P. Lei, H. Wu, J. Zhao, *Adv Sci (Weinh)* **2022**, *9* (10), e2105240, https://doi.org/10.1002/advs.202105240; b) Z. G. Fridlender, J. Sun, S. Kim, V. Kapoor, G. Cheng, L. Ling, G. S. Worthen, S. M. Albelda, *Cancer Cell* **2009**, *16* (3), 183, https://doi.org/10.1016/j.ccr.2009.06.017; c) A. C. Mihaila, L. Ciortan, R. D. Macarie, M. Vadana, S. Cecoltan, M. B. Preda, A. Hudita, A. M. Gan, G. Jakobsson, M. M. Tucureanu, E. Barbu, S. Balanescu, M. Simionescu, A. Schiopu, E. Butoi, *Front Immunol* **2021**, *12*, 708770, https://doi.org/10.3389/fimmu.2021.708770.

[2] a) W. He, J. Wu, J. Shi, Y. M. Huo, W. Dai, J. Geng, P. Lu, M. W. Yang, Y. Fang, W. Wang, Z. G. Zhang, A. Habtezion, Y. W. Sun, J. Xue, *Cancer Res* **2018**, *78* (12), 3293, https://doi.org/10.1158/0008-5472.CAN-17-3131; b) N. Niu, P. Lu, Y. Yang, R. He, L. Zhang, J. Shi, J. Wu, M. Yang, Z. G. Zhang, L. W. Wang, W. Q. Gao, A. Habtezion, G. G. Xiao, Y. Sun, L. Li, J. Xue, *Gut* **2020**, *69* (4), 715, https://doi.org/10.1136/gutjnl-2019-318362.

[3] J. Wu, L. Zhang, J. Shi, R. He, W. Yang, A. Habtezion, N. Niu, P. Lu, J. Xue, *EBioMedicine* **2020**, *58*, 102920, https://doi.org/10.1016/j.ebiom.2020.102920.

[4] Y. Wei, Q. Zhao, Z. Gao, X. M. Lao, W. M. Lin, D. P. Chen, M. Mu, C. X. Huang, Z. Y. Liu, B. Li, L. Zheng, D. M. Kuang, *J Clin Invest* **2019**, *129* (8), 3347, https://doi.org/10.1172/JCI127726.

Supplemental tables

Supplemental table S1

gene_name	WTvsCon_log2FC	KOvsCon_log2FC	KOvsWT_log2FC	N_CON_1_fpkm	N_CON_2_fpkm	N_CON_3_fpkm	N_WT_1_fpkm
Csf3	6.161925	7.494047	1.320433	0.419104	0.247421	0.141785	17.76486
Cxcl1	5.169733	6.167974	0.986575	0.315837	0.093228	0.106849	6.973322
Cxcl3	7.188463	7.813023	0.612136	0.474026	0.333148	0.515461	64.86816
Cxcl5	3.881433	4.955089	1.057889	0.095361	0.093829	0.143383	2.161736
Ccl9	1.309423	2.488302	1.166177	7.409716	9.223322	9.825224	23.78867
Il10	2.255842	3.289282	1.022037	0.72329	0.53375	0.356844	2.568924
Ccl17	4.037083	4.956738	0.902207	0.115213	0	0.259848	1.824645
Cd81	1.224044	2.155236	0.918714	3.085414	4.290637	2.876284	7.109268
Lrg1	1.006834	2.214459	1.194484	86.38964	102.0098	76.97817	149.3238
Lipg	5.834575	6.557764	0.715907	1.548117	1.261671	1.781057	80.13809
Spp1	1.070176	2.336809	1.253609	7.294621	7.177389	7.699245	21.3243
Dcstamp	2.191954	3.165723	0.958949	0.022229	0.109361	0.075204	0.331343
Ifitm2	1.255847	2.323244	1.054908	335.1546	280.0406	256.4179	617.047
Qsox1	2.541791	3.716023	1.161808	19.26362	16.43105	16.21394	101.727
H2-Q10	1.208956	2.230132	1.008359	15.85358	18.2656	15.94261	38.44552
Atp1a1	1.041603	1.681829	0.627644	24.45003	22.89634	22.01662	46.79918
Pf4	2.334544	3.608155	1.261233	5.585185	5.73787	5.279482	26.93395
Dap	0.965069	1.805247	0.827565	19.99616	20.73421	18.8027	38.37448
Pfkfb3	1.390731	2.442669	1.039146	13.54973	12.76171	11.33181	34.99392
Olfml2b	1.353474	2.551603	1.185756	9.930488	8.678964	9.731227	26.54158
Stfa3	1.106414	2.061331	0.941452	8.911655	8.344156	10.53581	26.78092
Id1	0.629985	1.739215	1.096738	13.17121	13.81346	11.45636	18.69077
Isyna1	0.812981	1.639448	0.813773	13.05452	15.06922	10.99307	19.20486
Cib2	1.440299	2.118674	0.665708	7.975814	8.294909	7.409735	21.3668
Slc44a1	1.898386	2.677291	0.766495	6.233171	6.367913	6.90405	28.19203
Ppbp	3.474751	5.274941	1.781335	0.546809	0.807031	1.479907	8.710845
1110008P14Rik	1.005657	1.980585	0.962249	7.188099	8.110755	7.139152	13.02423
Ctsl	1.634659	2.400597	0.753309	5.776833	5.782767	5.320648	17.03776
Camkk1	3.967539	4.638135	0.657941	1.270802	1.18786	1.200192	18.11332
Soat2	3.005247	3.817876	0.800363	2.488659	2.027388	1.750243	14.63354
Romo1	1.265861	1.994274	0.717409	8.641115	6.474185	4.380535	12.77668
Stfa2	1.23536	1.851578	0.603195	3.897667	5.566975	9.216036	13.82145
Atpif1	0.632125	1.314669	0.669917	7.808246	8.451036	7.739345	11.79178
Serpinb2	5.743045	6.843139	1.058768	0.126052	0.099221	0.426442	13.99767
Pou2f2	1.991436	2.66843	0.664441	3.225707	3.272829	3.281115	13.27181
Ssbp4	0.735526	1.630609	0.882386	6.082901	6.740192	6.500787	9.119224
Gdf15	1.654903	2.370989	0.703505	2.941784	3.27205	3.750111	10.21753
Ptges2	2.398717	3.122061	0.710316	1.861582	2.38462	1.967248	9.871029
Gm28875	1.054203	1.99088	0.923882	3.237393	3.332381	4.26858	7.377478
Dok2	1.225787	1.822498	0.584607	4.917943	3.413337	4.14216	8.858928
Tgm1	2.724796	4.021468	1.285282	1.423363	0.958229	0.781433	6.542776
Ddt	1.821326	2.894861	1.060423	1.378977	2.336737	1.98701	6.066415
Aldoc	0.773321	1.462219	0.676705	7.640625	6.071409	4.686732	8.80873
Eva1b	1.926615	3.265363	1.329282	1.890629	1.350178	0.825304	4.147594
1700020L24Rik	1.326084	2.268117	0.93288	3.440364	2.497186	1.65362	5.096536
Pdpn	2.792471	4.553956	1.746545	0.391102	0.448953	0.5513	3.248789
Tnfrsf9	0.692015	1.95006	1.24529	3.524445	3.788897	3.28752	5.857366
Sema6b	2.085881	3.201127	1.102424	1.074545	1.497808	1.363217	4.546412
Ggt1	2.317467	3.21849	0.88815	0.954871	1.486159	1.174685	6.307766
2310039H08Rik	0.689317	1.342194	0.63953	4.196649	4.205671	4.907777	8.108783

Igfbp6	5.12965	5.868454	0.725338	0.159765	0.157197	0.240219	5.705395
Slc32a1	2.828406	3.695253	0.854652	1.016278	0.770791	0.668524	5.483338
Gpr55	2.250206	3.589506	1.327216	0.87532	1.09614	0.861456	4.151366
Eci1	0.601657	1.212799	0.598744	4.016714	3.631715	3.366586	4.905007
Uck2	1.273625	1.895522	0.609241	2.74204	2.548677	2.407727	5.795539
Npy	3.964841	6.071299	2.092954	0	0.205893	0.117987	1.754481
Stard10	0.769504	1.457496	0.675577	3.280445	3.131375	2.40179	4.766538
Ceacam16	0.590988	1.317354	0.712935	3.513321	4.205251	2.818273	4.993863
Rab7b	0.825688	1.705486	0.867252	2.356481	2.482424	2.556273	4.354795
Gstm4	0.952602	1.825857	0.860821	2.254985	1.672593	2.386426	4.169163
Ifitm5	1.192512	2.282938	1.076958	1.315986	1.371004	1.222133	3.317333
Gm42647	6.439277	8.184562	1.733759	0	0	0	2.644251
Asnsd1	0.703294	1.6627	0.94642	1.829624	2.295693	2.082168	3.862434
Spint1	3.976403	4.598705	0.608562	0.235422	0.294813	0.386156	4.147122
Gpr35	1.505922	2.32106	0.80297	1.95807	1.348621	1.656065	5.540811
Zfp771	0.752451	1.385917	0.620607	2.417118	2.668305	1.894727	3.5699
Fam213b	1.114064	1.720061	0.593385	1.499225	2.666583	2.015779	3.652844
1110065P20Rik	0.734879	1.365044	0.61763	2.089422	2.281141	1.678397	2.639778
Epx	0.603075	1.533952	0.918346	2.682939	2.171466	2.317931	4.192578
Olfm1	1.899614	2.970712	1.059148	0.783369	0.721581	0.60146	2.670705
Gm19345	0.813862	1.659098	0.831812	1.550558	2.171101	2.55556	3.055667
Bri3bp	0.998488	1.91428	0.903358	1.514055	1.596131	1.199229	2.720234
Slc16a1	1.062304	1.720309	0.645315	1.431648	1.527124	1.629536	3.377929
Lrrc75b	1.780357	2.940444	1.148402	0.578942	0.911421	0.652865	1.965602
3110082I17Rik	0.769958	1.564756	0.782147	1.744765	1.918693	1.550889	2.849267
Dmkn	2.994242	3.648811	0.64148	0.513307	0.372148	0.456986	3.045352
Slc2a9	0.91485	1.633453	0.706003	1.46796	1.490222	1.760502	2.821928
Rnase4	0.895715	1.948925	1.040829	1.121442	1.316985	1.346223	2.426479
Dab2	4.865025	5.718399	0.840586	0.080658	0.103171	0.090957	2.547288
Ssh1	0.91357	1.893977	0.967546	0.852461	1.212272	1.194124	2.078442
Snhg15	0.757529	1.449564	0.67908	1.350847	1.329137	1.228492	2.273323
Zfp395	0.717116	1.624314	0.893772	2.139555	1.846641	1.044112	2.750858
Adora3	1.892686	2.989763	1.085805	0.571708	0.406264	0.465621	1.390683
H1fx	3.386491	4.042355	0.645578	0.389281	0.191512	0.054873	2.085259
Gucy2g	3.86472	4.486461	0.608364	0.144438	0.157907	0.16288	2.496794
Per2	0.859285	1.621443	0.750486	2.174209	1.432811	1.573728	4.173451
Car4	5.638301	6.326435	0.67334	0.060244	0.029638	0.067936	2.946446
Micall2	1.538641	2.430471	0.880486	0.800262	0.674915	0.564027	1.597549
Pla1a	2.473066	3.541499	1.057133	0.442156	0.145017	0.299167	1.78495
Gm10851	1.804105	3.102711	1.288443	0.632611	0.380382	0.396325	1.53883
P2ry6	0.78468	1.524542	0.728105	1.087159	1.231149	0.902131	1.509638
Lmna	1.044879	2.553712	1.498075	0.588339	0.608826	0.503316	0.897743
Ndufa12	1.119619	1.96494	0.834238	1.132302	1.002694	0.553315	1.758084
Gstt3	2.961917	3.839698	0.867657	0.262033	0.257822	0.104291	1.852358
Draxin	6.620994	7.558271	0.92724	0.011388	0.033616	0	1.442873
Tmem119	1.83748	2.912341	1.065932	0.511615	0.427884	0.201929	1.572844
Vcan	2.251535	3.036812	0.772641	0.434052	0.334409	0.429444	2.109548
Gstt1	3.63625	4.348645	0.701037	0.232537	0.076267	0.087409	1.371996
Cbr3	1.2762	1.950416	0.661182	0.613917	0.743447	0.745559	1.803761
Itih1	5.51875	6.734506	1.197641	0.017983	0	0.060839	1.105719
Emp1	2.164854	2.841691	0.663368	0.319296	0.3338	0.416325	1.6453
Nrg1	1.931763	2.720885	0.776384	0.274614	0.315234	0.374195	1.092608
Arhgap24	0.654479	1.293027	0.625325	1.030547	1.305588	1.010219	1.625192
Xrcc5	0.962939	1.575774	0.600838	0.579446	0.843798	0.67957	1.317139
Entpd3	3.402711	5.141942	1.733112	0.110396	0.077587	0.017785	0.675835
Hyal2	0.704905	1.443623	0.727259	0.773287	0.743165	0.466431	0.971693

Gnb5	1.589285	3.093535	1.492949	0.266155	0.224466	0.192946	0.903243
Gm26756	3.15523	4.931362	1.749498	0	0.045029	0.103216	0.468976
Oaf	1.335502	2.154276	0.805053	0.270305	0.509759	0.431827	0.881356
U90926	5.049845	6.323977	1.261447	0	0.072835	0	1.034413
Gm26535	3.128906	3.807946	0.665989	0.182213	0.022411	0.15411	0.848749
4732414G09Rik	3.690527	4.651247	0.948057	0.073352	0.024058	0.082718	0.706123
Rhbdd2	0.619411	1.30308	0.670807	0.660264	0.665121	0.771162	1.127685
Bend4	2.010551	2.679783	0.658177	0.395355	0.265228	0.236428	1.651824
Gm4211	1.59173	2.361972	0.7558	0.373456	0.131233	0.300814	1.068583
Spaca6	2.218279	3.053688	0.821646	0.134075	0.156656	0.245691	0.87433
Gm26767	0.75536	1.626711	0.860147	0.754134	0.455623	0.462512	1.121615
Cdk20	1.025477	2.091711	1.052051	0.29719	0.266612	0.48299	0.732868
Fcrlb	1.997794	2.781507	0.769837	0.276726	0.181519	0.26005	0.945258
Kcnn3	8.784956	10.53016	1.734567	0	0	0	0.427582
Zfp934	1.158013	1.83917	0.666781	0.277398	0.192069	0.451848	0.832699
Parp16	1.185873	2.001507	0.803959	0.655284	0.359862	0.24059	0.8802
Gm13571	1.538277	2.671554	1.122818	0.190907	0.187839	0.12917	0.551331
Gpc3	1.40905	3.303841	1.872693	0.132088	0.064983	0.198605	0.430685
Trio	0.732551	1.373008	0.62834	0.69104	0.630123	0.687932	1.035224
6720489N17Rik	1.106076	2.175064	1.058371	0.2631	0.237299	0.148347	0.367655
Map6	1.799153	2.847741	1.036072	0.181178	0.0764	0.145937	0.518412
Gm36660	2.237057	3.582354	1.334612	0.152982	0.090314	0.086258	0.570072
Snai3	2.855135	3.991109	1.128992	0.181878	0.035791	0	0.474424
4931406B18Rik	1.254678	2.470618	1.199638	0.126155	0.144816	0.272672	0.391754
Pax8	2.556909	3.679077	1.118957	0.087935	0.086522	0	0.436908
Gm13986	1.040566	1.860359	0.80798	0.232363	0.18508	0.199644	0.484478
Oit3	1.851593	2.817274	0.951609	0.063722	0.146296	0.095812	0.47491
Gja5	1.390592	2.197718	0.800257	0.123674	0.202811	0.058111	0.259233
Creg2	2.235061	3.690316	1.45385	0.068057	0.066963	0	0.28078
Slc28a3	4.524631	5.980991	1.437866	0	0.014748	0.016902	0.188503
Gipr	3.624151	5.133909	1.505741	0.034015	0	0	0.15844
Mrc1	2.487787	4.122933	1.619661	0.046476	0	0.031446	0.147209
Adgrl2	1.049021	1.779145	0.71607	0.108445	0.065663	0.117588	0.240911
Stxbp6	3.009203	4.21943	1.211393	0.02236	0.011	0	0.04166
Adgrv1	2.337309	3.234431	0.880737	0.007141	0.007026	0.01879	0.06431

N_WT_2_ fpkm	N_WT_3_ fpkm	N_KO_1_ fpkm	N_KO_2_ fpkm	N_KO_3_ fpkm	KOvsWT _p	WTvsCon _p	KOvsCon _p
17.00068	23.61243	53.47266	49.82547	43.94783	5.59E-22	1.65E-61	8.3E-100
6.049975	5.614571	13.09447	11.58121	12.59537	1.47E-11	5.82E-35	4.12E-55
62.53443	62.84973	95.57752	98.82317	99.14583	4.08E-17	0	0
1.432693	1.225821	3.446343	3.642441	3.055943	7.56E-05	9.08E-11	4.31E-21
20.30019	20.98129	52.35953	47.31908	47.72916	2.38E-33	3.02E-26	3.2E-102
1.85226	3.273976	6.469548	5.35272	3.983807	0.00021	1.54E-07	1.44E-19
1.888296	2.16732	3.997221	3.190502	3.897219	0.029693	0.000105	1.84E-07
8.575629	8.164449	16.8776	13.86715	14.72139	5.75E-13	9.1E-13	1.13E-42
187.5854	192.9724	455.2744	394.5571	374.1718	2.13E-26	1.14E-17	7.04E-86
94.30006	84.5871	103.1897	142.8132	183.5212	0.003233	0	3E-132
13.36173	11.52533	37.15184	35.37738	38.70459	6.87E-13	4.87E-08	6.26E-82
0.250479	0.355444	0.694112	0.594358	0.554062	0.034102	0.00671	3.21E-06
693.4861	756.9263	1622.563	1369.433	1343.468	1.8E-24	6.7E-32	7.26E-96
100.3465	98.19908	222.9895	229.0807	226.0496	4.87E-67	2.6E-202	0
37.55768	38.98239	79.77531	76.46067	77.24181	6.66E-42	3.61E-42	8.5E-132
49.4402	45.62825	74.49397	74.68466	72.04463	1.35E-16	4.62E-37	7.28E-86
27.51223	28.81573	74.01872	62.5881	64.77879	3.47E-26	2.63E-39	2.8E-111
36.99565	40.10408	72.97732	64.36293	69.50167	1.4E-19	6.76E-23	8.51E-72
33.16687	29.9238	54.63867	70.0635	78.76925	8.85E-18	7.96E-49	1.26E-87
25.98939	19.40056	47.34809	57.27974	60.51582	4.07E-20	6.96E-26	9.9E-107
15.16538	17.30489	47.12549	35.96071	31.85932	2.15E-05	2.15E-05	6.48E-22
18.35534	22.08798	46.44926	40.55921	40.67236	4.11E-19	1.6E-05	5.58E-41
22.63601	26.47813	40.39983	42.73667	38.10802	1.17E-11	7.91E-09	3.88E-42
21.50457	21.02844	37.63608	32.67445	31.96745	2.72E-09	2.35E-27	1.07E-56
24.63146	19.39278	37.2234	39.63716	47.12093	3.35E-09	3.53E-54	5.5E-135
10.08225	12.03493	37.0411	36.49832	33.43972	8.73E-36	2.13E-29	5.2E-100
14.74881	16.99536	32.96543	27.41383	27.64897	8.56E-15	3.8E-14	1.36E-59
17.16359	17.88859	30.53836	28.53153	29.5542	8.32E-21	1.27E-70	1.3E-142
19.42861	19.33368	30.38044	31.3537	28.8462	1.06E-13	4.5E-176	3.3E-245
17.40626	18.09687	29.75555	31.94131	26.44088	1.96E-11	1.21E-71	1.4E-131
14.61723	19.31236	28.26068	23.92162	25.33426	3.44E-05	2.39E-07	3.63E-22
14.8112	14.78241	24.75519	21.85117	19.92202	0.001159	1.7E-05	1.99E-12
11.11356	14.03133	22.67015	18.95428	17.70581	5.28E-06	6.19E-05	1.92E-18
10.45888	9.579756	22.21631	25.37358	24.01237	3.49E-14	1.67E-25	2.4E-101
13.15081	12.20362	21.92158	20.38175	19.47955	3.93E-14	3.66E-96	1.3E-156
10.31476	12.51668	20.90936	19.58837	18.96008	2.18E-12	1.48E-07	4.8E-46
10.38321	10.4964	18.63758	17.58756	14.88658	7.35E-08	6.17E-23	3.19E-47
11.10569	11.60694	18.14709	19.47572	16.19595	4.68E-11	2.22E-71	2.3E-124
6.938575	7.963851	16.67504	13.17413	12.81299	6.01E-08	1.47E-06	2.87E-23
8.92573	11.17073	16.61994	14.26389	12.95169	2.08E-05	3.99E-14	6.69E-32
6.925619	7.398497	15.49701	18.15216	17.70504	3.48E-28	7.23E-42	1.5E-114
6.670412	7.277764	15.34948	14.85049	11.93419	1.25E-07	8.29E-09	7.56E-27
10.16912	12.29056	15.33215	16.86242	18.27174	3.83E-07	5.28E-06	7.2E-22
5.154042	6.137912	14.17676	13.77324	11.21076	5.59E-15	2.03E-12	1.75E-50
8.030429	5.888298	13.64823	11.95605	10.94464	4.16E-06	7.65E-06	6.47E-21
2.871156	3.402704	12.26863	10.05245	9.951156	1.11E-23	3.95E-16	1.92E-58
5.415411	5.750085	12.20893	14.79163	13.76943	3.64E-22	1.45E-05	4.86E-42
6.313812	5.741267	11.66311	13.13964	11.15943	7.68E-16	2.82E-28	7.66E-92
6.064373	5.552049	11.45946	11.67119	10.35446	1.5E-13	2.22E-34	3.81E-79
6.209446	6.94424	11.32441	11.50273	10.65369	0.001656	0.007412	2.78E-09

5.837034	7.613685	11.27058	9.560426	11.14031	0.000127	1.51E-22	6.66E-32
5.552144	6.352641	10.8348	11.423	9.49888	2.85E-10	8.5E-38	1.04E-75
4.597298	4.670367	10.73943	11.89943	11.34768	1.86E-28	1.24E-30	7.8E-103
5.170963	6.534935	10.21636	7.774826	7.392665	0.005337	0.014005	9.46E-08
6.305955	6.401843	9.513299	10.15272	8.827643	2.06E-08	4.99E-23	6.18E-51
1.500462	1.771382	9.347212	5.495035	6.798933	1.35E-08	0.000169	1.69E-11
4.564033	5.618366	8.98185	7.737779	7.387512	1.44E-05	6.41E-05	1.2E-16
5.157156	5.65518	8.482177	10.41053	7.286602	8.52E-05	0.005781	1.75E-10
4.788377	3.878771	8.464444	7.863526	7.626805	4.5E-12	4.68E-08	3.85E-37
4.122282	3.817758	8.455399	7.61936	6.114571	4.65E-06	7.96E-05	2.36E-16
1.903106	3.64053	7.654287	6.726732	4.547962	0.000803	0.00591	1.46E-10
2.378883	1.334861	7.49762	7.268813	6.546309	4.6E-06	2.41E-06	3.12E-11
2.837061	3.331271	7.395992	6.363354	5.767573	5.21E-09	0.000273	9.75E-23
4.691566	5.374749	7.183947	7.745585	6.953734	4.91E-05	2.66E-40	8.8E-62
4.889025	3.568307	7.175504	8.343855	9.12899	3.7E-07	4.01E-15	2.56E-47
3.985724	4.158794	7.158786	6.051774	4.959252	0.001146	0.000769	1.51E-10
4.66627	4.989689	6.751879	6.93784	6.570129	0.007632	0.000379	3.01E-10
3.371713	4.01127	6.280892	5.028998	4.203953	0.004639	0.003087	1.85E-09
3.368853	3.256126	5.986558	8.285024	6.394368	2.07E-07	0.002671	1.41E-16
2.458558	2.696431	5.768183	5.220388	5.474765	1.46E-12	1.26E-15	3.09E-48
3.665348	4.206957	5.327817	6.776757	7.538301	0.000832	0.012713	6.05E-09
2.667962	3.178746	5.260717	5.662635	5.262677	4.61E-13	2.08E-09	1.66E-39
3.275667	2.856739	5.257123	5.059504	4.692661	4.1E-06	2.02E-09	1.57E-26
2.912491	2.456468	5.166558	6.167184	5.061924	3.75E-12	1.51E-13	6.85E-51
2.670368	3.320769	5.091919	5.429479	4.842107	2.61E-06	0.000327	4.72E-17
3.293212	4.268987	5.01458	6.372071	5.338838	0.000866	5.66E-21	6.38E-37
3.031286	2.96965	4.86056	4.850545	4.812073	4.17E-08	5.44E-08	2.16E-26
2.593956	1.973492	4.675186	4.318029	5.506578	5E-06	0.00391	6.88E-14
2.759595	2.655276	4.575354	5.245138	4.573613	6.64E-12	5.17E-60	1.38E-88
2.053432	1.960654	4.303464	3.930187	3.788225	1.57E-15	6.62E-08	1.18E-34
1.785475	2.500152	4.006339	3.70335	2.909217	0.013014	0.02673	1.76E-06
2.640243	2.847967	3.971969	5.770784	5.724809	6.32E-08	0.00106	4.29E-14
2.163578	1.777504	3.966993	3.927639	3.505853	1.36E-08	1.33E-10	1.02E-36
1.894115	2.746103	3.925127	3.810197	2.902141	0.024514	3.17E-09	5.15E-15
1.956293	2.264232	3.744443	3.386804	3.21433	0.000512	3.3E-27	3.14E-41
2.755458	2.406443	3.692686	5.407682	6.760413	0.000503	5.84E-05	2.19E-15
2.005594	2.748174	3.683807	4.861019	3.887934	0.002877	3.03E-15	4.63E-20
1.990822	2.311877	3.668878	3.86181	3.434617	7.27E-07	1.61E-09	9.22E-31
1.962667	1.136737	3.630527	2.899222	3.69976	4.74E-05	7.69E-08	3.58E-21
1.980049	1.388371	3.627508	4.731985	3.7229	5.28E-07	2.29E-05	1.43E-19
1.9331	2.08369	3.415983	2.781415	3.031068	0.000564	0.003714	7.81E-11
1.28846	1.307111	3.228965	3.206181	3.512529	1.81E-17	4.39E-05	5.9E-41
1.585164	2.485027	3.208222	3.676219	3.628567	0.001603	0.003579	5.08E-10
1.278913	1.75422	3.181859	2.825973	3.004838	8.54E-06	2.41E-16	7.48E-36
1.88984	1.263964	3.141124	2.936193	2.726988	3.85E-07	6.57E-18	3.55E-24
1.336388	1.178966	3.128452	2.638134	2.869154	1.04E-05	7.73E-06	4.47E-18
1.874962	1.671543	2.940785	3.366658	3.448237	4.79E-10	1.3E-40	5.4E-87
1.627698	1.932007	2.790076	2.960675	2.345956	0.014739	3.19E-09	7.1E-15
1.741479	1.51021	2.662423	2.886068	2.52225	0.029103	0.003043	2.7E-07
0.976332	1.319347	2.60487	2.884973	2.401231	6.89E-08	5.37E-12	6.13E-19
1.512705	1.586069	2.431907	2.563042	2.593561	2E-05	1.2E-18	1.85E-37
1.324462	1.226898	2.327297	1.999507	1.9658	1.8E-07	1.68E-19	4.02E-45
1.938795	1.678859	2.184969	2.913632	3.063851	8.39E-05	0.000132	9.14E-14
1.519506	1.242625	2.171172	1.748479	2.310795	0.017121	0.003516	1.36E-07
0.775433	0.756513	1.956084	2.545077	2.905994	8.44E-13	1.12E-09	5.36E-28
1.068438	1.184029	1.856165	1.785936	1.744894	0.002118	0.028616	2.36E-07

0.662116	0.482794	1.764359	1.960604	2.098447	1.49E-07	0.000749	1.64E-18
0.328153	0.473494	1.746545	1.354726	1.222141	0.000324	0.007137	2.43E-07
0.946034	1.207648	1.738844	1.93587	1.684314	0.003109	0.001018	3.89E-10
0.909923	0.765877	1.66934	2.19127	2.701645	0.00707	0.000307	7.47E-07
1.166568	1.071156	1.659462	1.674717	1.601726	0.015585	1.51E-08	7.68E-15
1.001842	0.574939	1.654176	1.424229	1.349435	0.003661	3.57E-07	4.34E-13
1.030618	1.035043	1.622604	1.741352	1.770228	5.64E-05	0.003042	5.16E-12
1.061546	0.884576	1.553502	1.893353	2.287552	0.002272	1.39E-13	1.03E-32
0.628475	0.702524	1.503909	1.528349	1.068548	0.03086	0.002622	1.63E-07
0.763956	0.819699	1.497247	1.208276	1.678347	4.56E-05	3.69E-12	2.87E-26
1.016446	0.671985	1.457387	1.794	1.896139	0.000409	0.019757	1.28E-09
0.814789	0.559056	1.448056	1.443979	1.509478	3.68E-07	0.001018	7.84E-17
0.897639	0.997743	1.32011	1.937813	1.642212	0.049901	0.001589	5.95E-07
0.603655	0.389064	1.292585	1.753119	1.714966	6.54E-17	6.33E-13	1.13E-18
0.663025	0.53149	1.194104	1.010503	1.042594	0.006705	0.001279	2.22E-09
0.999057	0.97468	1.163165	1.600715	2.265416	0.003272	0.001218	8.62E-09
0.508446	0.412992	1.159091	1.0391	1.036627	0.000643	0.003758	5.97E-10
0.248058	0.352008	1.145672	1.051095	1.626523	1.29E-06	0.039588	3.25E-11
1.114958	1.164238	1.115326	1.982052	2.077506	0.000715	3.21E-08	2.44E-13
0.449178	0.577417	1.083956	1.06775	0.780674	0.005059	0.045731	1.59E-06
0.477232	0.395597	0.999	0.852682	1.02527	1.56E-06	6.05E-07	1.52E-21
0.548477	0.431672	0.981906	1.343968	1.620534	1.04E-05	2.04E-05	2.34E-16
0.298091	0.821132	0.946516	1.493613	1.100928	0.016941	0.002573	1.72E-07
0.430758	0.454856	0.811543	1.104275	1.048124	8.77E-06	0.00319	1.29E-12
0.320272	0.284888	0.788134	0.653097	0.843649	0.000157	1.62E-05	6.6E-15
0.476043	0.301813	0.710194	0.739616	0.778118	0.007177	0.012413	7.35E-08
0.282855	0.339635	0.644817	0.709904	0.794129	0.022675	0.007339	8.99E-07
0.453958	0.300505	0.62574	0.570803	0.577975	0.014099	0.006301	1.49E-07
0.199184	0.164604	0.59873	0.649881	0.536584	2.38E-05	0.001383	1.72E-12
0.168888	0.352443	0.598015	0.72994	0.620412	6.32E-06	2.94E-07	1.54E-14
0.156793	0.111976	0.501547	0.341047	0.378482	0.004735	0.007969	9.18E-06
0.152344	0.131141	0.451483	0.461551	0.421985	2.07E-05	0.002762	1.09E-10
0.175174	0.180462	0.325594	0.334562	0.330443	0.007555	0.005856	4.41E-08
0.148877	0.084123	0.145453	0.213513	0.278652	0.048427	0.015425	4.24E-05
0.051206	0.047018	0.134202	0.100014	0.067806	0.025512	0.000361	2.48E-08

Supplemental table S2

gene_name	WTvsCon_log2FC	KOvsCon_log2FC	KOvsWT_log2FC	N_CON_1_fpkm	N_CON_2_fpkm	N_CON_3_fpkm	N_WT_1_fpkm
Cxcl10	0.746875	-3.15559	-3.91259	711.1585	532.0935	706.9811	961.116
Cxcl11	0.622606	-5.23617	-5.86834	9.958347	7.110683	11.07146	14.13389
Cxcl14	3.963771	3.250753	-0.72691	0.129654	0.159463	0.073104	1.540013
Il1a	5.039309	3.852295	-1.19897	3.989959	2.715616	3.755152	118.2186
Il23a	2.531441	1.312818	-1.22958	0.658872	0.337107	0.50524	2.970811
Icam1	1.145196	-1.00115	-2.15828	143.186	112.3657	137.3755	256.952
Nos2	3.403875	0.853615	-2.56223	31.97202	16.67998	16.63466	234.9546
Ptgs2	1.589739	0.390788	-1.21091	11.45598	7.137153	10.71701	33.4144
Jun	0.710735	-0.33906	-1.06217	39.79528	31.07974	30.63667	54.3729
Nfkbia	1.41472	0.729149	-0.69742	512.8546	397.1973	504.5477	1148.4
Nfkbib	1.211397	0.227177	-0.99637	45.10393	38.36156	43.99714	90.70149
Nfkbiz	2.087728	1.293853	-0.80583	38.77937	31.23556	36.22507	148.3112
Tnf	1.980894	0.34245	-1.65026	186.6549	149.953	193.3751	697.3336
Tnfaip3	1.746116	0.449014	-1.30886	48.81133	36.81039	49.84995	146.1138
Tnfaip8	1.008063	0.410558	-0.60965	10.50445	10.00841	11.05039	21.38267
Tnfrsf10b	1.730919	0.918332	-0.82717	0.152095	0.192408	0.098009	0.404834
Csf1	0.880491	-0.01817	-0.90985	43.6436	27.69397	36.84895	81.6927
Cxcl2	3.220931	2.496401	-0.73615	64.80342	47.70172	83.18539	682.6191
Clec4e	1.424438	0.271125	-1.16485	212.498	151.3434	213.5507	562.5762
Ccl4	2.029815	0.892428	-1.14904	102.6469	85.63581	123.0386	421.8733
Traf1	1.81484	1.1701	-0.6566	8.716041	6.646703	8.284183	27.31848
Hcar2	0.788699	-0.60842	-1.40918	567.7697	432.0261	469.611	850.4839
1600020E01Rik	1.051594	-0.17175	-1.23481	1.450382	1.534777	1.759014	3.212225
2010110E17Rik	1.86373	0.106048	-1.76686	1.280055	0.662886	1.367525	4.330648
9130230L23Rik	1.961937	0.889396	-1.08529	1.240355	0.848342	0.938172	4.093615
A530058O07Rik	2.355511	1.503888	-0.86255	0.316869	0.19053	0.297774	1.590774
AA467197	2.167728	1.038306	-1.14065	29.33895	25.96132	32.30793	131.2033
Acadl	0.880292	0.062338	-0.83006	30.481	31.99636	35.50541	61.33217
Acod1	0.88257	-1.19139	-2.08542	1193.644	808.7277	1101.947	1994.402
Adamts7	0.595581	-0.09901	-0.7067	0.433634	0.380289	0.446481	0.73769
Adgb	2.87331	2.13034	-0.75474	2.419655	1.590316	2.124648	15.53847
Adgre1	1.669965	1.060676	-0.62154	51.20886	42.63005	47.37354	155.4878
Agpat4	0.909811	0.305668	-0.6159	19.18436	17.47383	20.86746	35.29489
Agtrap	0.913902	0.321917	-0.60447	35.88672	32.46263	31.35671	59.2842
AI413582	0.670392	-0.14901	-0.83211	24.40632	23.00841	20.08314	29.28672
Aifm1	1.259028	0.66374	-0.60855	4.893064	4.290804	3.050646	9.956805
Ak4	2.069906	0.982984	-1.10247	1.567797	1.304313	0.776187	5.094138
Alkbh5	0.703298	-0.12797	-0.84417	32.45345	26.92945	20.46815	43.82533
Alpk1	1.676822	0.476802	-1.21172	8.21725	6.439307	9.353982	26.48386
Ankrd66	1.247353	0.430384	-0.8282	16.76448	14.13225	17.62768	36.97611
Ap1ar	0.66086	-0.01689	-0.69044	2.567998	2.400391	2.248127	3.538149
Arf4	0.673297	0.005575	-0.67992	24.30904	22.59226	24.66979	39.64245
Arf6	0.708975	0.001216	-0.72027	81.95747	75.89231	77.44659	119.0406
Arfgap3	1.136688	0.556593	-0.59323	6.382928	5.84042	4.589986	11.92138
Arhgap5	0.839125	-0.21174	-1.06297	0.446036	0.433515	0.545926	0.891861
Asph	0.69691	-0.25282	-0.96044	1.040808	0.604316	1.099051	1.788477
Ass1	0.724753	-0.03797	-0.77504	12.83005	12.69627	13.02973	20.77396
Atf3	0.830703	-0.9941	-1.83754	38.04997	38.98037	32.86335	65.13184
Atp10d	0.758706	-0.28438	-1.05525	1.848719	1.633395	1.940115	3.240668
B3gnt3	1.381653	0.129633	-1.26442	2.132286	1.640268	1.552026	4.225687
BC001981	5.895306	4.265185	-1.64689	0	0	0	0.285663
BC024978	0.994956	0.392267	-0.61439	0.39187	0.354726	0.441906	0.737432
Bcar1	3.309309	2.024592	-1.31112	0.039072	0.038444	0	0.24266

Bcl2l11	1.494661	0.388444	-1.11817	14.72925	11.44453	13.45764	37.7159
Bcor	0.656188	-0.02609	-0.69429	5.788001	5.540337	6.465394	8.925283
Bdp1	1.196388	-0.15498	-1.36362	3.93899	3.356811	3.914005	9.349351
Bhlhe40	3.067635	2.177572	-0.9022	19.15662	14.39044	15.82585	142.4185
Blm	1.331955	0.478486	-0.86581	1.052549	0.736893	0.760862	2.570809
Bnip3	0.944299	0.207819	-0.74912	69.33766	56.51835	41.60165	125.1465
C130012C08Rik	2.382047	1.624644	-0.77233	0.177739	0.174882	0.091106	0.812854
C1ra	0.649554	-0.73668	-1.39798	4.251241	4.085187	4.906071	5.996194
C1rl	1.095889	0.128305	-0.97961	24.65179	19.80326	25.55864	44.6848
C9orf72	1.001588	-0.17011	-1.18421	9.26308	8.228108	8.12454	18.40954
CAAA01147332.1	0.829976	0.21352	-0.62887	29.73154	26.37631	24.73364	46.99555
Card19	0.823135	0.173788	-0.66178	197.7454	182.338	175.9722	285.9284
Casp4	0.601413	-0.5745	-1.18774	60.225	55.03232	69.17485	99.39286
Cass4	1.398095	0.408874	-1.00062	10.56855	5.804944	9.241765	23.83411
Cbln3	0.801369	0.004066	-0.80966	1.594274	1.065726	0.974402	2.040765
Ccdc184	1.903977	0.214972	-1.69752	0.079569	0.039145	0.067297	0.148253
Ccdc34	0.649787	0.003548	-0.65992	0.460535	0.533099	0.407324	0.83283
Ccnl1	0.780384	-0.03649	-0.82869	37.13925	32.08268	39.32017	71.62638
Ccrl2	1.702656	-0.23056	-1.94499	152.5161	121.4919	152.3775	460.8433
Cd200	0.801948	0.128847	-0.68519	0.980749	0.947442	1.126084	2.109733
Cdc42ep2	0.872407	-0.10965	-0.99433	135.2792	118.6456	127.4703	213.2375
Cdc42ep4	1.012714	-0.32756	-1.35223	34.90091	26.77466	34.84774	61.01418
Cdkn1a	2.289433	1.474367	-0.82647	13.1006	8.494976	8.533209	45.37093
Cflar	1.238905	0.178931	-1.07166	25.80243	20.90351	28.61207	63.21167
Chil1	1.269917	0.629214	-0.65332	304.4603	324.9399	315.4465	694.2322
Chka	1.828763	0.91421	-0.92614	5.200904	4.04152	6.009385	18.47674
Chrna9	0.969775	-0.04118	-1.02446	0.469684	0.44288	0.286897	0.948041
Ciapin1	1.993604	0.990043	-1.01543	5.740655	4.060773	4.958541	18.66704
Ckap2l	0.644279	-0.17237	-0.82852	3.926564	2.823298	3.895725	6.032152
Cp	2.12444	0.529393	-1.60602	0.489058	0.444652	0.530561	2.151152
Cpa6	1.066878	-0.38725	-1.46835	0.112551	0.136299	0.078106	0.27423
Cped1	1.63371	0.534423	-1.10898	0.343342	0.253368	0.596905	1.292756
Crem	0.878242	0.129294	-0.76054	3.053892	2.204188	3.002021	5.287584
Cry1	1.385778	0.450193	-0.94796	30.74444	28.66281	30.17133	75.1054
Csrnp1	0.638853	-0.69962	-1.35035	37.49595	30.06498	33.11159	44.55749
Cycs	0.657186	0.004283	-0.66495	10.71655	9.876746	10.65276	16.53116
D330045A20Rik	2.161512	0.788992	-1.38424	0.237511	0.093478	0.160702	0.796553
Dcun1d3	1.399719	0.707861	-0.70407	4.578585	4.103566	4.720155	12.47947
Ddit4	1.258891	0.380928	-0.89054	48.45529	39.74154	32.95281	89.433
Dld	0.896101	0.320636	-0.58721	10.82125	10.55241	12.78319	21.69
Dleu2	0.598141	-0.02026	-0.63063	4.948011	4.976452	5.827266	9.326205
Dntt	0.65324	-0.14974	-0.81667	2.337607	1.998844	1.349424	2.87769
Dram1	0.702799	0.046802	-0.6683	45.68353	43.18887	45.57325	73.52201
Dusp10	0.760765	-1.27395	-2.04542	1.20697	0.976874	1.339129	1.831702
E230013L22Rik	0.754879	-0.23914	-1.00571	2.456839	2.166201	2.518673	3.864186
Egln3	1.320994	0.576324	-0.75827	75.52585	65.86304	22.55295	129.4039
Ehd1	0.990586	0.394332	-0.60839	273.4161	233.9356	269.16	461.7493
Eid3	1.395598	0.019947	-1.38774	1.134471	0.803692	1.125807	3.593363
Enc1	0.990704	-0.92791	-1.93091	1.662693	1.525267	1.705822	3.028047
Ereg	5.761513	3.503966	-2.28369	0	0	0	0.133645
Ero1l	1.678632	0.495281	-1.19576	62.13273	45.11472	32.75229	177.2392
Esrra	0.750535	0.093474	-0.66948	47.20758	42.8459	42.68817	64.59886
Ets2	1.637905	1.056665	-0.59297	41.49455	28.74068	36.4175	112.1604
F11r	1.456745	0.225297	-1.24559	0.600422	0.809577	0.727241	1.71949
F3	1.159583	-1.50313	-2.67446	4.82754	3.689286	3.461926	7.466439
Fam71f2	3.825709	2.824714	-1.01138	0.371281	0.182657	0.26168	4.582979

Fam78b	1.628937	0.431417	-1.19772	0.018725	0.018425	0.147815	0.148279
Fgd6	0.675631	0.069514	-0.61761	1.221611	1.336317	1.661214	2.430073
Fkbpl	0.794901	0.14835	-0.65852	19.54772	15.87532	17.84487	32.74608
Fmnl2	1.809204	0.495425	-1.32536	12.95555	10.54525	14.18322	46.8092
Fosl1	4.118602	3.07911	-1.0513	1.908065	1.433651	0.978044	24.65947
Fpr2	0.676529	0.017758	-0.67094	817.3737	729.0652	806.9349	1293.059
G0s2	1.770186	0.670605	-1.11181	91.78665	82.88592	90.93232	277.0469
Gatm	0.858967	0.025962	-0.84652	5.249036	3.786136	3.693974	7.629125
Ggh	0.641855	-0.18286	-0.83685	37.00835	36.17636	38.26728	60.39076
Ggta1	1.923478	0.267846	-1.66718	13.11655	11.54464	14.80552	52.25151
Ghitm	0.593104	-0.05903	-0.66425	31.48592	27.76867	31.43463	45.16606
Gm10053	0.734497	-0.05513	-0.80229	3.931318	3.74534	3.166635	6.336551
Gm12589	2.001171	1.068735	-0.94006	2.413329	0.6393	1.779426	6.312405
Gm12840	4.748292	2.014735	-2.76327	0.094449	0.092931	0	3.16758
Gm13477	0.75932	-0.08242	-0.85368	0.793412	0.889086	1.018985	1.888917
Gm14005	0.998527	0.3364	-0.67458	5.630934	5.597705	5.447477	10.31532
Gm14023	2.434874	1.042738	-1.40265	0.598511	0.392595	0.562444	3.314458
Gm14455	1.167023	-0.13411	-1.31334	0.248357	0.217214	0.311187	0.6684
Gm15283	1.962754	1.224745	-0.75217	1.208045	0.767657	0.624385	4.009284
Gm15568	2.141431	1.396342	-0.76314	0.976412	1.152864	0.38538	3.82042
Gm16181	1.029375	-0.09511	-1.13548	11.90654	8.323952	11.77792	21.89234
Gm16238	1.670867	0.576049	-1.1119	1.111627	0.894896	0.797723	2.165324
Gm16867	1.057239	-0.55398	-1.61766	0.334573	0.202007	0.420167	0.80047
Gm20100	1.182431	0.167771	-1.02462	1.01653	0.871963	1.234505	2.768147
Gm20186	1.355553	-0.76039	-2.11951	0.281997	0.55493	1.081213	1.31354
Gm20257	0.745599	-0.10968	-0.8672	4.808412	5.166898	5.850456	10.25572
Gm21188	0.737123	-0.48109	-1.23015	20.81486	19.46079	22.39173	38.67359
Gm21370	0.585011	-0.99344	-1.59111	10.19008	8.218289	8.665494	13.30585
Gm26809	1.528571	0.255091	-1.28502	8.065438	6.37211	7.639134	21.65292
Gm26835	2.493525	-0.16931	-2.68431	0	0.053391	0.040795	0.202207
Gm27167	0.950497	-0.03888	-0.99895	2.086006	2.622615	4.835402	6.045884
Gm32089	2.044678	1.054343	-1.00241	2.63087	2.149017	2.574952	13.08696
Gm340	0.701081	0.007521	-0.70576	3.064988	2.359458	2.865361	4.541911
Gm34643	0.624819	-0.62669	-1.26355	2.276674	2.367363	2.742419	5.061347
Gm36161	0.691047	-0.85756	-1.56055	59.88653	54.21326	68.32974	114.103
Gm37084	0.72821	-0.16506	-0.90451	0.562273	0.316135	0.520841	0.926026
Gm37305	0.759118	0.063674	-0.70783	2.158968	2.165122	2.153716	4.525405
Gm37498	1.472664	0.051947	-1.43043	0.192367	0.131037	0.250303	0.716835
Gm37691	1.720723	0.884862	-0.84756	18.89589	17.09759	21.10097	67.47604
Gm41077	1.102612	-0.26352	-1.37827	1.302367	1.582951	1.814227	2.854783
Gm42587	1.917371	0.655746	-1.27388	0.269043	0.029413	0.134843	0.556978
Gm43814	1.422061	0.089396	-1.34328	12.47479	9.31154	11.96557	28.45262
Gm44616	0.8823	0.114953	-0.77902	2.154054	0.874688	1.349497	3.44008
Gm44851	1.925879	1.303194	-0.63488	0.692474	0.580406	0.578439	2.604325
Gm45266	1.156201	-0.43471	-1.6036	0.408785	0.107258	0.368785	0.507765
Gm48420	3.713327	-0.18779	-3.91366	0	0.097113	0	0.275843
Gm4876	1.910938	0.483086	-1.44155	0.116192	0.057163	0.065514	0.288652
Gm48857	1.301943	-2.69515	-4.0006	0.660663	0.35457	0.745019	1.510704
Gm5424	0.693427	0.076353	-0.62881	9.981332	7.330448	11.36351	14.01463
Gm7099	0.810702	-0.12471	-0.94699	2.137884	2.313878	2.169774	3.485386
Gm8850	1.361284	0.047052	-1.32923	0.604279	0.792757	0.454291	1.876486
Gm9320	1.544008	0.962476	-0.59305	4.872481	5.457984	6.339951	15.0841
Gnptab	0.627902	-0.129	-0.77008	5.657198	6.517104	5.460067	8.993162
Gp6	1.665124	0.193135	-1.4832	1.856879	1.422126	1.697817	5.414012
Gpr15	1.234349	-0.40138	-1.6483	0.397688	0.355724	0.326158	0.63993
Hic1	1.590368	0.695708	-0.90803	0.300045	0.574908	0.463014	1.206355

Hilpda	3.223288	2.580683	-0.65473	6.01321	4.384602	3.935406	42.41437
Hivep2	1.664592	-0.04541	-1.72118	1.56681	1.257087	1.752266	4.877523
Hk2	0.610536	-0.23538	-0.85911	23.23535	22.28599	13.42553	29.22489
Hmgcs1	0.590158	-0.08228	-0.68465	11.07302	10.47651	11.36757	18.02529
Hmox1	1.904554	1.174241	-0.74268	125.5749	124.4885	128.5273	468.2249
Hsd11b1	0.661377	0.043942	-0.6294	21.04928	18.98394	22.51162	32.54836
Hspa13	1.028718	0.177618	-0.86294	2.625618	2.532265	3.136764	6.070655
Hspa1a	3.528064	2.88574	-0.65488	0.279431	0.078554	0.202571	2.287074
Hspa1b	3.053594	2.106024	-0.96476	0.401416	0.478115	0.238248	3.877357
Hspa4l	1.242027	-0.06182	-1.31595	2.556196	1.951809	2.350241	7.396684
Icosl	0.603836	-0.3003	-0.91615	51.41319	46.80331	58.68617	79.14677
Ighg3	1.957307	-0.19904	-2.16862	0.235746	0.231957	0.186093	0.219621
Ighv1-80	1.776608	0.738914	-1.05041	2.024594	1.494043	1.331811	5.1868
Il1bos	0.913268	0.296327	-0.62863	2.623141	1.896632	2.509883	5.128096
Il1f6	5.796966	5.030221	-0.77656	0.201198	0	0.151259	6.622742
Il1f9	1.171079	0.571154	-0.61203	414.0326	384.0787	443.1118	974.4906
Inhba	1.201241	0.358973	-0.8538	5.087863	3.76597	5.100006	11.56039
Insl3	1.264588	0.691364	-0.58503	7.984636	4.676378	5.574001	14.43418
Insl6	0.81641	-1.42895	-2.2576	6.27324	5.267134	5.187775	10.44153
Itgb1	1.240509	0.511986	-0.74087	44.57779	41.94815	43.44599	107.5391
Katna1	0.616566	-0.40185	-1.03035	28.27422	25.58999	30.24152	44.03441
Kcng3	6.116368	4.562196	-1.56258	0	0	0	0.378093
Lacc1	1.774344	1.073568	-0.71284	17.12877	15.5583	18.5095	60.1042
Lair1	1.272275	-0.75485	-2.03988	20.05933	18.82494	16.77784	47.81778
Larp4b	0.887006	0.274235	-0.62474	24.74704	20.30144	24.8929	45.40466
Layn	0.932176	-0.03573	-0.98056	0.835807	0.991687	0.942527	1.809185
Lekr1	1.916544	0.903835	-1.02655	0.16651	0.090109	0.046943	0.519654
Leng9	1.563841	0.506009	-1.07044	5.320115	5.589906	5.157868	14.6668
Lima1	1.782848	1.000824	-0.7949	2.117202	1.792144	1.808208	6.772812
Lonrf3	1.484527	0.737169	-0.75979	0.932099	1.008832	0.946003	3.198395
Lox	2.767673	2.143239	-0.63566	0.209299	0.266505	0.277675	1.686029
Lpp	1.065139	0.453337	-0.62368	2.046864	1.992825	2.55358	4.7271
Lrp11	1.542969	0.261258	-1.29812	0.381166	0.085236	0.156304	0.548782
Lrrc75a	0.809445	-0.49184	-1.31622	0.935491	1.115704	0.703292	1.901458
Lrrc8c	1.014779	-0.26857	-1.29562	10.94019	8.533846	9.98135	20.16028
Lta	0.619817	-1.17609	-1.80821	7.322136	7.836432	7.484473	11.4486
Ly6i	1.570869	-2.61114	-4.19414	402.0873	320.1697	363.2507	958.8662
Lzts3	1.255012	0.540688	-0.72657	0.334258	0.274072	0.266998	0.752536
M6pr-ps	1.653247	-0.10883	-1.7753	0.428092	0.421212	0.563212	1.661707
Maff	1.709607	0.183664	-1.53787	17.21334	12.35436	13.8762	41.49349
Malt1	0.805702	-0.02321	-0.84092	10.95067	8.803457	10.9376	18.95998
Map1b	1.371384	0.414827	-0.96729	0.134539	0.073542	0.118002	0.282006
Mapk8	1.322	0.524876	-0.80868	1.019418	0.967394	1.161253	2.940659
Mapkapk2	1.300385	0.647492	-0.66491	326.9432	265.4714	313.9643	710.6438
Metrnl	1.944988	0.671595	-1.28562	50.59411	38.17157	39.2432	141.4698
Mfsd6l	1.071015	-0.13142	-1.21449	1.437364	1.55569	1.880237	3.749328
Mfsd7a	1.855937	0.870539	-0.99734	22.78577	16.28614	19.67744	66.58517
Mipep	1.030002	0.124402	-0.91817	4.296675	3.438322	3.370641	6.469959
Mir22hg	0.663618	0.083805	-0.59177	64.05174	53.51298	65.67012	104.746
Mitf	0.662465	0.050553	-0.62468	4.711936	4.984161	4.634563	7.692987
Mllt6	1.568033	0.925167	-0.65512	25.11519	20.34794	22.12882	60.53673
Mmp2	0.690401	0.078062	-0.62443	3.144548	2.627317	3.347955	5.122456
Mrgpra2a	0.761318	-0.4434	-1.21651	30.99466	22.60222	27.62787	45.95908
Mrpl39	0.719852	0.05172	-0.68023	5.143413	5.10676	5.721057	8.886232
Ms4a6d	0.753819	0.143729	-0.62196	20.37693	18.16877	21.43914	35.8358
Msl3l2	1.325378	0.064467	-1.26891	0.719488	0.272279	0.811356	1.675684

Mtm1	0.734117	-1.0394	-1.78504	4.34743	3.21967	4.686878	7.419169
Mturn	2.255911	1.390835	-0.87838	1.091841	0.859435	0.750478	4.494868
Nab2	1.783901	1.158804	-0.63755	6.201826	5.170983	4.155352	15.83216
Nbas	0.857679	0.052827	-0.81716	0.970912	0.808338	0.898365	1.478509
Nfyb	1.467361	0.350781	-1.12875	5.176438	5.185224	6.179993	15.63185
Ninj1	0.859932	-0.0669	-0.93939	400.1646	368.6079	337.1368	570.5567
Nlrp3	0.704558	-0.19458	-0.91123	95.59471	81.53452	91.85037	146.9307
Nr4a1	3.379815	2.753995	-0.63807	8.826372	7.107028	8.012175	74.4282
Nr4a3	1.663972	0.357301	-1.31771	1.325438	0.798054	1.305054	3.842549
Nupr1	1.638279	0.182835	-1.46699	13.07349	9.386796	12.13955	34.71633
Olr1	2.471444	1.472069	-1.0102	15.26905	7.291615	13.89086	79.47874
Osgin2	1.629314	0.931639	-0.709	1.254333	1.150501	1.450454	3.921522
P2ry13	0.649057	-0.00092	-0.66206	13.79755	11.70885	13.78939	21.75759
Pcgf3	0.674247	-0.1107	-0.79719	1.80562	1.727761	1.91722	2.803522
Pde4d	2.408915	1.408583	-1.01472	0.468586	0.330018	0.26699	1.952915
Piga	0.589999	-0.11619	-0.7194	2.949376	2.327056	1.652525	3.896802
Pik3ap1	1.343643	0.725758	-0.62983	79.9858	60.42903	74.62086	178.5651
Pik3r6	0.917138	-0.21835	-1.14766	32.17663	32.19039	36.4326	61.21561
Pla2g7	0.727218	-0.53475	-1.27386	301.234	283.8167	371.8577	549.8258
Plagl2	1.855126	1.043322	-0.82401	12.17431	10.13969	11.80745	41.08552
Plcb3	1.550909	0.825656	-0.73754	15.3088	12.49683	13.63912	39.72513
Plekha1	0.786907	-0.11537	-0.91447	3.812764	3.642156	4.291766	7.259208
Plekha8	2.63118	1.705642	-0.94389	0.126808	0.141406	0.076266	0.70093
Plk2	1.291395	-0.24964	-1.55269	14.63672	11.83258	15.9703	36.55801
Pmaip1	0.848542	0.204849	-0.65539	13.87605	11.14265	18.12117	29.33573
Pnrc1	0.688325	0.083682	-0.61679	152.557	129.6908	139.2675	219.8057
Pola2	0.956474	0.229654	-0.73897	11.50572	10.28611	11.60293	20.28487
Ppa1	0.611501	-0.0219	-0.64536	3.979249	3.794827	4.556375	5.988331
Ppm1n	2.112203	1.490199	-0.63448	6.255942	5.190361	4.962227	21.01547
Ppp1cb	1.387792	-0.04772	-1.44747	24.01226	21.58675	26.73558	68.17602
Ppp1r15a	1.515927	0.583431	-0.94455	11.31157	8.729238	10.61854	27.89428
Prdx5	0.90699	-0.00413	-0.92356	1335.163	1197.279	1141.986	2013.876
Prkd3	1.056606	0.133254	-0.93521	18.06092	14.27999	18.43943	36.91099
Rab10	1.058568	0.001656	-1.06883	25.87582	21.81098	25.43488	50.92857
Rab11fip1	0.921506	0.166987	-0.76673	25.62883	21.80721	23.47222	46.74525
Rab20	1.693263	0.708046	-0.99727	41.43655	37.6312	44.95232	125.8734
Rab32	1.292839	0.384312	-0.92089	109.7546	105.2256	109.0934	263.6728
Rab33a	1.91902	1.259862	-0.67199	2.966083	2.063018	2.076088	9.14714
Rabgef1	1.30925	0.644019	-0.6774	11.66485	8.96103	9.276894	25.1224
Ralgds	1.052374	0.441554	-0.62289	36.78736	31.19151	36.44266	70.42316
Rbpj	1.536148	0.951919	-0.59634	10.10213	7.810152	8.532231	28.35454
Rel	0.655958	-0.05556	-0.72336	15.87168	13.8294	16.68179	27.01537
Rhoh	0.866654	-1.36152	-2.23986	8.750336	6.006511	5.728129	11.28341
Ripk2	1.172158	0.460313	-0.72337	4.87887	4.77977	5.644117	11.71551
Ripk3	1.001358	0.181559	-0.83244	10.67086	9.558745	7.488076	15.95848
Rnd1	1.714626	0.767614	-0.9592	40.97654	34.68173	37.25051	110.9138
Rrad	1.425381	0.637473	-0.79963	4.919302	3.214513	4.319368	10.63493
Saa3	2.048924	0.681046	-1.38105	17.08863	16.21031	14.27357	59.38149
Sash1	0.720159	-0.0406	-0.77265	1.057489	0.863678	1.161338	1.532464
Seh1l	0.681545	-0.39585	-1.09017	20.07665	16.45614	15.13774	28.55717
Serf1	3.558493	2.857527	-0.71251	1.047728	1.479102	1.438356	15.53211
Serpine1	1.46417	0.53438	-0.93764	0.106949	0.165362	0.344586	0.412769
Slc16a10	0.601164	-0.30922	-0.9231	3.703224	4.15066	4.085286	5.647366
Slc25a37	0.929904	-0.15028	-1.09271	59.10136	56.4967	55.23476	111.501
Slc30a6	1.302239	0.642055	-0.67241	3.210457	3.805857	4.492766	8.864466
Slc43a3	1.652163	0.937189	-0.72716	3.833871	3.106565	3.485647	11.29574

Slc7a11	1.360225	0.596115	-0.77588	19.42219	15.12147	21.06268	53.54398
Smpdl3b	1.480693	-0.26976	-1.76262	93.99527	70.52537	79.83071	209.0965
Sod2	2.315581	1.52875	-0.79866	46.1897	35.50322	45.755	201.1551
Spata13	1.340278	0.663179	-0.68911	51.17122	40.57995	47.78548	119.714
Spin4	2.022857	-0.00582	-2.04793	0.070955	0.041889	0.016003	0.304069
Spryd7	1.173587	0.429573	-0.75476	1.464947	0.917257	1.551878	2.963835
Sqstm1	1.293373	0.674007	-0.63172	192.0748	179.4387	188.9205	446.4455
Stxbp1	0.985736	-0.08933	-1.08781	0.805867	0.583026	0.521203	0.92739
Taf7	0.771213	-0.63507	-1.41771	6.11563	5.735007	7.240319	11.92927
Tanc1	1.61921	0.5818	-1.04829	0.264191	0.315933	0.444594	0.946614
Tank	0.77989	-0.26504	-1.05653	19.52608	16.9565	22.86279	37.99182
Tbc1d9b	0.804782	-0.02367	-0.84091	17.99886	16.45887	16.90036	28.81575
Tbk1	0.846094	-0.44091	-1.29902	44.92192	36.82338	43.36483	81.80769
Tcea1	0.604527	-0.37231	-0.9884	11.62011	8.903346	12.30697	17.60936
Tcea1-ps1	0.600274	-0.04667	-0.66004	3.406425	5.478708	4.653973	7.384304
Tcp10b	2.470394	1.593829	-0.88979	0.175777	0.207543	0.158577	0.835145
Tet1	1.169998	-0.57346	-1.75354	0.088118	0.041466	0.077768	0.153474
Tgif1	1.300311	0.340708	-0.97136	12.04391	9.498925	11.62283	27.57944
Ticam2	0.99799	0.078563	-0.9313	1.175945	1.186714	1.394101	2.485962
Tifa	1.35984	-0.46763	-1.83954	30.74817	28.05008	32.62845	76.08761
Tmbim4	0.914781	0.301982	-0.62532	44.60383	42.0358	39.58723	74.9405
Tmem263	1.713654	0.88607	-0.84049	1.97693	1.553224	1.231136	5.318954
Tmem45a2	1.080247	-0.51113	-1.60329	0.63476	0.526589	0.603526	1.171088
Trib1	0.803418	0.190643	-0.62486	21.40163	16.37612	17.27238	31.63723
Trib3	1.136293	0.45648	-0.69138	7.858761	4.222692	6.285253	12.6174
Trim13	1.859588	0.869802	-1.00359	5.024675	7.062747	4.209215	20.66318
Ttc39c	0.770973	-1.27	-2.05278	26.75527	23.0178	27.02329	43.0458
Ttyh1	1.692688	0.638516	-1.05967	0.092835	0.054806	0.177971	0.432424
Txnrd1	1.843921	1.049067	-0.80699	22.99076	18.60026	21.51575	72.60841
Tyk2	0.982456	0.010782	-0.9841	39.78438	33.76384	33.43418	62.30486
Ublcp1	0.617221	-0.38368	-1.01308	26.27831	25.69554	29.26587	40.69288
Upp1	1.004163	0.343121	-0.67327	518.298	411.5878	426.4301	821.3411
Vezt	0.946178	0.263515	-0.69583	0.249725	0.254185	0.223347	0.457266
Wfdc17	4.336019	3.686695	-0.66065	22.44337	21.86404	29.56899	463.8103
Wnk2	2.437759	1.134575	-1.31647	0.495764	0.480926	0.440953	2.478395
Xkr8	1.066722	0.057051	-1.02183	7.182685	6.107835	6.938267	13.15246
Zc3h12c	1.579336	0.4021	-1.18848	2.050401	1.7791	2.595135	7.358512
Zeb2	0.591123	-0.36427	-0.9672	10.59547	9.513565	12.81902	18.4993
Zeb2os	0.687582	0.08346	-0.61554	2.886573	2.371699	3.288704	4.047552
Zfp131	1.02245	-0.50911	-1.54333	12.41117	11.30246	14.66167	27.2376
Zfp446	0.778251	0.083149	-0.70855	0.34665	0.308595	0.214071	0.39214
Zfp503	2.283172	1.287069	-1.00853	3.91893	2.667377	3.136291	15.3101
Zfp991	1.074931	0.157002	-0.93019	2.476779	2.057446	2.083323	5.541455
Zfp992	0.923618	0.041541	-0.89366	1.015189	0.973262	1.071428	1.915748
Zfp993	1.064694	-0.16762	-1.24271	1.161164	0.411301	0.838034	1.94713
Znf41-ps	1.418853	0.331161	-1.09828	2.380555	1.883497	3.21035	7.339059

N_WT_2_fpkm	N_WT_3_fpkm	N_KO_1_fpkm	N_KO_2_fpkm	N_KO_3_fpkm	KOvsWT_p	WTvsCon_p	KOvsCon_p
801.664	1485.872	12.06559	20.33952	185.4623	0.000708	0.014739	0.003228
9.833113	19.02671	0.013538	0.104334	0.625244	1.72E-06	0.053699	1.58E-05
1.89256	2.256053	1.518156	1.021409	0.923306	0.026469	1.53E-12	1.24E-07
98.88995	123.7028	51.67654	52.53834	45.66498	5.99E-30	1.3E-301	3.4E-162
2.861647	2.77633	1.577275	1.233154	0.891487	2.85E-06	1.51E-15	0.001266
260.7193	345.1874	62.8977	68.4553	63.81419	5.77E-82	2.14E-22	1.81E-22
211.8753	239.2883	35.69423	40.83518	40.75497	7.4E-185	2.05E-23	0.013024
22.3441	31.81474	10.12706	15.33488	12.75289	1.83E-14	5.1E-09	0.020595
51.74014	58.89645	26.55996	27.25483	25.96905	9.64E-34	4.59E-11	0.002874
1178.717	1417.689	752.1519	787.0834	792.1332	8.49E-14	3.63E-39	1.7E-12
92.32073	110.0135	51.98	49.9789	46.32311	4.45E-23	8.47E-33	0.026324
142.7564	157.3656	74.15988	89.04931	95.76526	8.87E-16	9.2E-118	1.87E-30
598.6577	781.059	241.0267	225.3331	201.5971	4.62E-53	5.94E-71	0.002042
146.2223	158.8987	54.19338	65.03984	64.64994	1.98E-46	1.06E-61	0.000172
20.89204	20.75811	15.0343	13.57874	13.07667	9.32E-13	2.41E-37	2.07E-05
0.356112	0.705065	0.244996	0.311221	0.28045	0.030781	0.00014	0.057514
57.8128	58.28419	31.03644	29.14715	46.05701	0.001017	0.000462	0.948564
490.9535	637.9925	372.1676	376.3455	349.2752	4.7E-11	6.47E-34	4.34E-68
479.9774	496.4658	227.0995	226.469	239.2508	2.22E-44	1.2E-34	0.022139
355.4213	484.169	213.9401	185.2405	174.9979	6.03E-22	2.62E-55	6.33E-12
25.34728	29.91852	19.04538	17.43468	16.40209	7.46E-12	1.91E-63	3.56E-24
789.8884	880.4081	325.804	318.7541	313.5041	1.35E-77	1.04E-15	3.48E-09
3.335823	3.204558	1.519092	1.319649	1.339799	8.54E-13	1.74E-09	0.419917
4.278742	3.295115	1.168698	1.222331	1.13946	6.06E-07	1.72E-06	0.836221
3.819429	3.805819	1.922061	1.97885	1.676378	6.76E-17	1.16E-35	2.02E-06
1.244246	1.241828	0.427527	0.790071	1.049913	0.007881	8.85E-11	0.000856
114.5156	144.7833	77.02509	50.01499	51.66335	1.96E-14	6.02E-82	2.12E-11
56.90988	60.78396	35.45545	32.66016	33.52202	7.39E-21	2.56E-21	0.54895
1726.645	1962.096	473.2055	407.441	470.898	2.2E-114	6.28E-14	1.42E-21
0.646979	0.505397	0.286175	0.41408	0.469921	0.021332	0.044466	0.765457
15.21436	13.85306	8.875936	8.822067	8.981645	3.39E-16	6.1E-105	3.69E-50
149.4244	141.3661	97.08137	103.7021	92.01522	2.63E-14	5.46E-83	1.05E-27
36.47327	35.51114	26.73758	22.10872	21.78081	3.45E-09	2.42E-21	0.010581
62.26541	65.06236	43.93126	40.03545	39.9096	6.27E-13	2.27E-27	0.000624
34.61387	42.82383	22.6465	20.99881	16.87322	1.99E-08	6.53E-07	0.263519
8.903887	10.27518	7.1033	6.436919	5.75049	1.01E-06	3.06E-16	7.69E-05
5.066007	5.13122	1.813118	2.349104	3.029468	2.08E-10	1.75E-26	8.78E-05
42.18037	43.15898	21.71422	25.7195	25.20847	2.69E-21	2.13E-08	0.348654
24.03319	25.65222	10.40299	11.52715	11.27524	4.5E-43	2.22E-46	0.000233
36.24839	41.03995	27.19517	19.94571	17.78678	2.39E-08	1.54E-24	0.009033
3.648136	4.150653	2.162892	2.48446	2.448364	5.21E-08	1.53E-07	0.901727
35.47427	38.20011	22.73829	24.13883	24.53597	1.3E-14	1.4E-14	0.953328
130.616	132.3876	77.68263	81.91112	74.50206	8.85E-17	1.43E-17	0.989354
12.47659	12.38425	7.822992	8.37707	8.412993	1.12E-08	1.01E-18	7.11E-05
0.947227	0.690688	0.330256	0.457087	0.435781	1.95E-06	0.000112	0.397058
1.454189	1.169211	0.628417	0.670776	0.988693	9.76E-07	0.000612	0.258458
21.20891	21.2971	14.23476	11.73612	11.33351	4.34E-11	9.68E-13	0.767687
60.77416	68.29341	18.76681	17.72076	18.35481	9.49E-87	4.18E-18	2.39E-20
2.92177	2.938309	1.308978	1.707708	1.41057	1.3E-12	5.34E-08	0.093822
4.189715	5.378571	2.034396	1.739966	2.018873	1.45E-11	5.47E-12	0.575439
0.431894	0.57683	0.099736	0.183006	0.136479	0.035637	4.19E-05	0.019612
0.754656	0.85511	0.516634	0.486458	0.544173	0.007929	9.8E-05	0.159728
0.173433	0.367496	0.079074	0.149238	0.092747	0.024415	9.18E-05	0.048835

36.58277	36.60154	17.05533	16.88227	17.62857	2.32E-50	1.39E-55	0.000249
9.239926	9.666856	5.103237	6.042497	6.222878	4.57E-11	2.36E-11	0.824817
9.06202	7.101069	3.071123	3.632901	3.305167	1.64E-28	1.53E-23	0.202136
134.4481	134.2722	70.86198	74.6734	76.52361	1.26E-33	9.7E-211	3.88E-90
1.776234	2.030766	1.088499	1.19837	1.249256	4.57E-07	6.53E-12	0.016403
93.06789	101.8341	49.70884	65.28714	77.24554	2.12E-07	0.000103	0.451857
0.761372	0.744695	0.420444	0.432024	0.517801	0.020045	1.39E-07	0.001545
6.552502	8.053254	3.204884	2.54195	2.139724	4.98E-14	4.81E-05	0.00013
50.24225	53.62755	25.61389	27.20359	23.24014	1.16E-21	3.59E-23	0.280315
16.47336	16.0699	6.92966	7.739693	7.970813	7.46E-29	5E-22	0.151016
45.18409	50.6573	35.29974	27.92538	29.93692	0.000456	7.44E-06	0.30585
305.2379	385.7902	226.5663	204.449	192.3723	8.97E-09	1.35E-14	0.064857
83.84697	94.5851	42.17133	38.44106	42.48461	4.25E-38	1.42E-09	2.86E-08
18.43701	24.7417	10.56142	9.890763	13.35645	1.73E-13	7.68E-07	0.018739
2.169154	2.094764	1.372248	1.127259	1.126667	2.01E-05	0.000199	0.986715
0.203767	0.336783	0.103522	0.056986	0.053122	0.032332	0.024937	0.845671
0.65675	0.696464	0.446442	0.543242	0.409952	0.012681	0.016841	0.990536
54.06633	59.4246	32.67547	33.88974	38.64186	1.11E-13	8.96E-13	0.72758
421.2683	495.9626	128.213	113.359	119.674	2.6E-105	1.02E-65	0.028556
1.607411	1.559804	1.129056	1.089771	1.095253	0.004874	0.001806	0.637617
219.9438	260.158	132.2519	117.156	101.8945	1.09E-18	3.77E-20	0.310977
64.04178	68.29877	26.11218	27.18011	23.16682	1.12E-47	9.04E-22	0.005982
40.24182	60.62267	25.47253	26.29293	31.49845	2.86E-09	7.31E-16	2.03E-22
56.91715	56.39477	25.42064	28.5445	30.8118	4.8E-31	3.77E-32	0.12628
735.8778	832.82	536.5773	481.4925	434.2597	8.55E-11	2.62E-52	1.77E-11
17.027	18.25633	9.987501	9.913929	8.658696	1.1E-24	1.29E-53	4.06E-12
0.581356	0.809916	0.492255	0.355066	0.313572	0.010252	0.017824	0.930569
18.87276	20.81801	9.662866	9.51485	9.962749	6.37E-32	9.62E-68	9.07E-16
5.33713	5.131442	3.012761	3.262783	3.108791	7.24E-08	0.000143	0.353567
2.194113	1.985547	0.730247	0.650259	0.716386	1.77E-24	3.12E-34	0.018092
0.230585	0.179152	0.082603	0.066139	0.100189	0.003513	0.031684	0.532466
1.018475	1.338848	0.558375	0.539602	0.611262	6.95E-08	1.56E-09	0.08096
5.031973	4.74325	2.648812	2.983326	3.34443	1.12E-08	1.59E-09	0.438182
75.71792	81.63235	39.79964	42.12385	39.72566	1.05E-31	1.74E-66	4.98E-07
46.14677	64.92199	18.27995	18.25261	25.0901	6.79E-19	1.17E-06	1.07E-07
16.14551	16.24859	12.20732	9.552179	9.36915	4.7E-08	5.21E-11	0.974021
0.746104	0.640402	0.247208	0.287281	0.310088	0.000212	2.1E-06	0.167814
11.78713	10.83729	7.129925	7.438367	7.182909	1.07E-13	2.54E-42	1.02E-10
89.03935	109.5594	45.65715	52.65009	58.57224	2.38E-14	8.06E-23	0.005023
21.23071	20.15972	15.52225	13.35801	13.48291	6.15E-09	8.43E-18	0.007723
7.316419	7.02763	5.65125	4.799721	4.976625	3.12E-07	6.39E-07	0.859589
3.506281	2.538962	1.375829	1.753866	1.981768	0.001082	0.012357	0.606019
70.64228	73.15099	47.99061	46.1491	43.8859	5.58E-17	2.31E-20	0.598341
1.475658	2.600071	0.287046	0.539092	0.623846	1.25E-09	0.005953	0.000378
3.889397	4.201517	2.324677	1.919494	1.760954	8.51E-06	0.00061	0.361708
125.4021	151.9512	66.41469	81.57317	94.81574	8.07E-10	0.008943	0.264184
496.0327	574.3802	335.081	346.3906	333.0935	3.35E-11	2.88E-25	7.25E-06
2.6031	1.792647	0.905271	1.21331	0.969472	0.000162	0.000236	0.963766
3.009385	3.609866	0.748182	0.931141	0.87914	7.22E-24	8.26E-09	3.24E-05
0.132256	0.175412	0.012443	0.054795	0.02554	0.012	7.28E-05	0.109481
130.8498	137.0107	52.68606	65.39771	78.06012	4.68E-18	1.75E-08	0.107357
74.08876	83.12238	45.37618	48.99865	46.4241	9.77E-11	1.57E-13	0.312963
102.201	115.2067	70.31043	72.76024	77.49472	5.73E-13	6.59E-48	4.44E-19
1.86791	2.238044	0.790813	1.082987	0.613656	2.1E-05	9.05E-07	0.544931
5.713273	13.37949	1.463486	1.297917	1.439389	3.09E-24	0.004231	2.38E-11
3.090124	3.797735	2.294486	1.373837	2.065635	0.000629	1.66E-16	1.09E-07

0.25895	0.149708	0.081208	0.062584	0.100013	0.033874	0.025908	0.615056
2.223004	2.027671	1.202925	1.434137	1.759086	0.000252	3.56E-05	0.715412
29.19562	29.80957	21.22237	18.41225	19.01844	1.58E-08	6.02E-11	0.273168
40.43742	43.82085	16.11286	17.28775	19.39868	1.11E-41	6.3E-63	4.95E-05
18.55027	31.74939	13.05923	10.73336	12.72325	9.58E-10	8.35E-75	1.88E-45
1186.41	1256.089	893.2472	772.9223	701.85	3.98E-12	7.18E-19	0.861014
257.9681	364.3849	154.6236	133.419	132.0599	1.04E-18	1.49E-50	7.26E-11
7.944011	7.387086	3.423145	5.144235	4.338757	1.15E-07	5.91E-08	0.893979
54.27122	57.99615	37.64101	32.20266	27.71227	5.83E-13	2.42E-13	0.124971
48.5524	47.77506	18.13948	15.65933	13.41104	1.59E-51	1.48E-84	0.03974
45.88375	44.80033	28.95053	28.54545	29.02414	9.91E-18	1.51E-12	0.530679
4.538427	7.043218	2.652108	3.634861	4.110549	0.003356	0.005828	0.850928
6.37041	6.460515	4.911006	2.48177	2.644013	0.003653	3.57E-07	0.028257
0.386995	1.599046	0	0.180379	0.588525	0.002796	0.000182	0.277293
1.377132	1.264498	0.630823	0.883905	1.020164	0.006163	0.012974	0.810883
9.792289	12.97388	7.559511	6.863675	6.50195	4.07E-07	2.08E-14	0.008206
2.690769	2.314323	1.182446	0.984287	1.006351	1.67E-06	8.78E-12	0.017553
0.537078	0.519106	0.215414	0.31621	0.17195	0.016667	0.034546	0.845168
3.067879	3.03001	1.52805	2.186974	2.352349	0.001353	4.34E-12	0.000144
3.700703	3.627625	1.566759	2.42419	2.650961	0.007332	5.83E-09	0.001247
22.36024	20.53144	11.77665	9.873618	8.088548	4.54E-09	1.41E-07	0.694789
3.312574	3.42185	1.314786	2.412496	0.449787	0.03779	0.001186	0.396068
0.514081	0.650836	0.230836	0.108915	0.304594	3.24E-06	0.000765	0.187053
2.135969	2.108367	0.519973	1.344032	1.624142	0.002773	3.42E-05	0.669589
1.328776	2.174957	0.097837	0.26928	0.753072	0.000581	0.012646	0.344471
6.740168	9.283352	5.597391	5.074901	3.886043	0.000269	0.001193	0.662417
32.18828	32.84504	15.5486	13.3209	15.73841	1.15E-30	6.44E-14	6.82E-06
13.77504	13.2769	2.970291	4.466486	6.0968	1.53E-10	0.002576	0.000322
20.34938	21.20752	7.081759	8.49846	10.6102	4.2E-21	4.1E-40	0.112991
0.203811	0.11909	0.015689	0.051816	0.016101	0.007434	0.015928	0.917904
7.597528	4.578092	3.216551	2.655917	3.301136	0.00407	0.014262	0.928202
7.932269	9.057688	5.511005	4.787459	4.860563	3.65E-06	9.53E-17	3.47E-05
4.701308	4.137552	2.024819	3.169407	3.095894	0.000118	1.68E-05	0.970697
3.286163	2.944392	1.391257	1.70462	1.658139	6.54E-07	0.008891	0.011301
84.9824	93.27647	33.86844	31.47851	34.68921	4.44E-41	4.93E-09	2.33E-14
0.699387	0.670517	0.252554	0.47939	0.509453	0.004158	0.016509	0.649649
3.26061	3.098067	2.232708	2.154118	2.3407	9.46E-05	3.27E-05	0.718435
0.378945	0.473214	0.141181	0.197822	0.250271	0.001781	0.001997	0.928881
59.43555	59.83487	37.09089	31.75793	35.87407	3.26E-18	7.03E-65	6.46E-15
3.766821	3.386682	0.863824	1.389947	1.636689	0.000548	0.004292	0.590518
0.704298	0.365523	0.181499	0.285456	0.212882	0.031559	0.008303	0.456819
25.85091	35.33518	14.92425	9.447637	11.23226	6.88E-10	1.75E-11	0.732626
1.926318	2.637072	1.542115	1.436577	1.734848	0.007927	0.010489	0.747644
1.970383	2.412305	1.935337	1.371476	1.232833	0.016958	5.55E-10	0.000211
0.697901	0.743352	0.09455	0.364328	0.194073	0.005228	0.039003	0.560516
0.505513	0.649835	0	0	0.087858	0.017401	0.025835	0.960418
0.277718	0.327862	0.083983	0.110953	0.137907	0.024024	0.00833	0.600353
1.476548	1.299293	0.156281	0.057352	0.053464	1.25E-06	0.015352	0.008172
14.82293	17.11431	8.325986	11.81142	9.905322	0.000149	7.28E-05	0.698059
3.832406	4.222752	2.503314	1.32696	2.18853	0.020075	0.041428	0.792407
1.547488	1.326193	0.524125	0.57703	0.806863	0.03345	0.038265	0.953904
14.58948	18.4727	12.48347	9.233935	10.47628	0.001385	6.15E-14	2.11E-05
8.754314	9.335426	4.540104	5.594333	5.905014	1.32E-10	8.65E-09	0.335407
5.325875	4.928044	2.167747	1.658129	1.822689	2.06E-25	1.16E-30	0.3013
1.111017	0.782114	0.250863	0.207139	0.354008	0.003055	0.021619	0.561185
1.310289	1.485336	0.821829	0.769065	0.562294	0.000852	5.48E-07	0.060781

37.39785	53.42767	30.73475	25.73667	28.96219	3.3E-06	2.31E-74	5.14E-50
4.978489	4.530709	1.24667	1.48379	1.6752	3.57E-44	1.15E-40	0.779176
28.83175	31.34473	12.67542	16.33277	20.75394	8.29E-10	0.013405	0.454355
15.72657	15.44666	9.425359	10.67216	10.80922	1.41E-10	4.52E-09	0.450665
445.902	493.5075	302.4656	281.485	265.1707	4.69E-19	1.3E-146	1.51E-42
31.93613	33.71357	22.6433	20.97803	20.44862	2.96E-13	2.34E-13	0.66883
5.512262	5.199981	2.833529	3.127815	3.355433	3.07E-12	1.94E-15	0.222949
1.962759	2.140146	1.263766	1.86781	0.977191	0.012378	9.94E-20	1.46E-10
2.294014	3.119842	1.411006	2.320058	1.090785	0.000589	5.44E-21	5.86E-08
5.293479	3.424574	1.812037	1.884644	2.834237	0.00045	0.000133	0.718238
76.41775	81.19919	39.40153	44.30888	42.95932	8.62E-30	3.39E-11	0.003484
0.821056	1.485632	0.286266	0.067535	0.209853	0.00083	0.00125	0.785248
7.604293	3.808555	2.634066	2.577728	2.853519	0.017088	0.000508	0.202014
3.562337	4.429841	2.912941	2.523827	3.13106	0.0008	5.95E-06	0.15431
6.869934	5.677248	4.362766	3.266131	3.641694	0.001836	1.97E-16	9.74E-12
895.8248	905.5324	640.8381	595.1017	597.1063	8.21E-16	6.17E-50	5.8E-11
10.61339	9.66689	4.638059	6.380557	6.772931	6.6E-10	6.39E-21	0.024011
12.07388	16.98719	8.98669	8.532258	11.76146	0.00674	3.42E-07	0.009594
8.910731	9.912732	2.756841	1.996783	1.414669	1.01E-14	0.000442	2.67E-05
99.53186	97.94266	61.60946	61.55114	61.05952	1.91E-22	1.85E-59	3.8E-10
41.1227	42.83453	21.41154	20.55855	21.27813	7.03E-30	1.04E-10	0.000196
0.216906	0.099583	0.107136	0.05055	0.078538	0.041418	1.96E-05	0.008437
55.85054	57.87064	35.42219	36.53022	35.10959	1.25E-19	1.38E-86	3.14E-27
43.85483	41.90735	12.29307	10.95422	9.534542	1.08E-84	8.19E-45	2.92E-11
43.31737	39.72845	25.31196	28.99601	29.78299	1.18E-11	1.34E-20	0.010023
1.586416	1.849734	0.938161	0.915487	0.831536	0.000568	0.001535	0.91657
0.298489	0.328891	0.209414	0.151051	0.20751	0.007006	5.56E-05	0.09473
16.25038	16.30247	8.393677	6.735296	7.542962	4.09E-17	3.58E-35	0.000937
6.386682	6.391456	3.139379	4.169813	4.074189	2.54E-10	3.83E-49	5.88E-11
2.562042	2.264826	1.81904	1.453776	1.507329	0.000226	3.97E-11	0.002428
1.790839	1.609629	1.142615	0.928766	1.22746	0.002157	9.12E-22	3.15E-11
4.796018	4.168792	2.523248	3.239647	3.208907	5.91E-08	2.3E-21	0.000591
0.638916	0.619252	0.180331	0.380522	0.185073	0.003603	0.002643	0.686833
1.277696	1.626475	0.713051	0.812095	0.428988	0.000265	0.016959	0.238854
19.36984	19.56854	7.744238	8.880556	7.686172	3.86E-45	1.23E-24	0.022276
9.956735	13.1296	3.713976	3.189309	3.049314	2.47E-21	0.000179	5.84E-09
1040.954	1202.467	82.73383	53.17358	40.63069	9.32E-39	3.31E-53	4.47E-16
0.656263	0.668087	0.434881	0.372382	0.458715	0.025228	0.000615	0.188779
1.388637	1.342171	0.309423	0.613181	0.381072	0.002721	0.005761	0.893033
38.77286	60.78914	16.96962	16.72051	15.36336	2.55E-25	3.6E-28	0.167707
17.75131	16.56052	9.147965	10.63514	10.24203	1.79E-19	9.87E-16	0.834296
0.229691	0.323388	0.113451	0.128472	0.189622	0.0015	3.13E-05	0.286348
2.380026	2.482267	1.256729	1.487349	1.755018	8.92E-08	9.28E-20	0.002024
719.8393	786.3772	469.7105	472.3256	469.4081	3.34E-19	7.65E-48	4.51E-12
156.1288	191.895	75.49191	66.461	60.72531	4.14E-26	4.28E-54	9.36E-08
2.296892	4.082872	1.520981	1.537252	1.35626	6.44E-06	8.56E-05	0.650646
67.60324	76.9541	36.45472	35.39943	34.91214	6.64E-28	1.12E-57	8.26E-13
7.192932	8.868194	4.479721	4.281305	3.276959	2.44E-09	2.07E-12	0.436307
88.65914	94.75388	66.07413	63.69099	63.2361	4.74E-11	4.43E-11	0.413495
7.783393	7.065896	4.70038	5.0288	5.028175	4.86E-09	7.47E-10	0.66792
66.23023	72.25839	41.52022	44.74733	41.32444	1.83E-13	1.04E-58	4.65E-21
4.660733	4.808286	3.016947	3.656974	2.892993	7.73E-05	1.83E-05	0.670945
44.90909	45.81784	20.3386	19.38168	19.6406	3.03E-32	2.67E-10	0.001296
8.884888	8.334127	5.150624	5.402619	5.889597	5.93E-07	2.18E-07	0.7412
34.36903	30.20969	25.48972	19.75137	20.56949	6.41E-07	1.99E-11	0.277483
1.190553	1.587714	0.792066	0.554919	0.517297	0.000367	0.00103	0.894909

6.381757	6.427397	1.666068	2.03714	2.224358	2.7E-48	8.74E-09	5.16E-12
4.122214	4.234969	2.209702	2.293731	2.554758	9.86E-11	2.07E-39	2.33E-12
16.19153	21.15506	10.68852	10.90215	12.92333	3.12E-06	1.79E-30	8.08E-14
1.60659	1.732762	0.874516	0.939015	0.947374	3.58E-07	3.36E-07	0.777587
13.08268	16.65069	6.831005	6.951453	7.166663	3.12E-23	1.49E-33	0.004472
633.4299	789.2387	383.8254	348.1179	317.2974	1.74E-15	7.26E-15	0.49913
138.7574	149.6091	72.10245	79.72879	81.83366	6.35E-29	9.63E-18	0.039481
65.67486	107.3366	54.6139	50.15109	55.76465	9.4E-06	6.31E-45	6.6E-145
3.515875	3.405085	1.372689	1.464019	1.52325	8.22E-19	3.07E-19	0.105671
34.26386	37.90887	15.26218	13.0013	10.75688	2.61E-31	8.63E-42	0.243024
64.19388	57.05675	27.59465	32.02644	40.96777	1.59E-12	6.34E-13	3.9E-05
4.203078	3.699343	2.793642	2.304182	2.195276	1.6E-06	4.66E-24	6.44E-07
21.11635	18.30965	11.54071	13.37668	14.11711	3.18E-08	2.34E-08	0.994293
2.923753	2.906397	1.388515	1.818998	1.811664	1.52E-08	9.27E-08	0.479449
1.702726	1.985645	0.808583	1.007951	1.005476	2.03E-11	2.17E-35	6.25E-10
3.410726	3.070703	1.713484	2.116709	2.534614	1.72E-05	0.00134	0.57123
190.2893	173.1283	93.18249	128.1487	132.2814	1.19E-07	4.79E-41	4.86E-08
63.57568	64.2043	28.85873	28.57109	28.67161	7.27E-50	3.49E-28	0.020203
498.2179	525.1725	268.1552	207.7807	180.6283	2.23E-25	1.58E-13	6.66E-05
42.75394	38.74242	21.38761	25.39031	23.12659	1.59E-19	1.26E-89	6.33E-22
39.47836	41.3939	21.61677	25.80439	25.61089	5.27E-15	4.31E-61	3.62E-13
6.70855	6.146805	3.511831	3.813254	3.449628	1.16E-16	2.51E-12	0.346583
0.640821	0.795148	0.322631	0.524722	0.278438	0.001357	4.26E-13	0.000118
31.68825	34.83893	12.8325	12.03758	10.59935	2.12E-51	4.24E-31	0.051579
24.7598	22.9663	15.782	16.7766	16.83454	7.88E-09	1.53E-08	0.173826
209.608	245.0805	152.3521	144.2469	147.4007	4.64E-13	1.77E-14	0.353077
19.49533	24.53365	14.3076	12.97553	11.6186	3.14E-09	7.25E-16	0.066524
5.915647	6.775376	4.070912	3.897236	4.087139	1.1E-05	6.86E-05	0.894921
22.75486	26.75301	16.60028	14.83333	14.41758	2.32E-07	3.23E-52	2.23E-25
61.27297	58.52263	24.60196	22.55116	22.40376	4.68E-62	2.16E-49	0.632138
24.57855	34.53521	16.12458	15.61497	13.91016	2.97E-13	5.92E-29	6.52E-06
2207.75	2620.535	1420.599	1140.231	1080.202	1.03E-14	1.11E-19	0.969848
35.44875	32.52923	17.29704	18.66827	19.40269	8.43E-28	1.8E-25	0.222945
50.52536	49.77018	25.82105	23.27632	23.64898	8.34E-37	2.4E-32	0.987424
45.36133	41.29545	24.68815	26.48897	27.96336	2.81E-19	7.52E-27	0.076381
123.6763	148.4936	71.88049	64.5925	64.79554	3.65E-23	1.3E-60	3.11E-11
259.9964	264.9268	143.0909	139.3777	137.9515	1.42E-39	4.2E-79	1.47E-06
7.805325	9.764355	5.721929	5.664657	5.553741	0.000318	7.46E-16	1.04E-06
21.74383	26.7275	15.6385	15.08694	15.73356	2.98E-11	7.54E-28	7.56E-08
67.92041	76.69053	46.69405	47.45921	46.80929	3.82E-15	3.61E-32	1.83E-06
24.57862	23.24115	14.82917	17.80736	18.22493	1.73E-08	5.53E-47	1.81E-16
22.61197	22.93359	12.95459	14.89843	16.51492	5.66E-11	8.18E-11	0.624411
10.48275	15.31874	2.597769	2.324077	3.002528	9.24E-54	0.00122	2.03E-18
11.61099	10.87893	7.715588	6.325605	6.851455	1.04E-08	2.88E-19	0.002508
17.33564	21.87177	11.54128	10.09097	9.63682	4.21E-09	5.37E-11	0.234409
108.8309	148.1957	65.80239	65.33523	59.92846	2.85E-16	4.13E-49	2.61E-15
10.42439	12.11486	6.546749	6.275243	6.418062	1.48E-07	1.37E-14	0.001795
63.43155	72.82052	32.50181	23.60906	19.68216	3.43E-20	6.01E-83	1.11E-05
1.840802	1.664242	0.89324	1.056002	1.027475	4.08E-06	4.32E-05	0.835507
25.67245	28.10353	11.32177	14.20156	13.53858	1.32E-23	1.01E-09	0.00238
15.39869	15.3818	12.95961	8.438836	7.096252	0.000251	1.93E-58	3.89E-24
0.657323	0.603562	0.291541	0.248021	0.340009	0.020058	0.00267	0.333012
6.077697	6.262098	3.170118	3.405542	3.002663	5.68E-16	6.38E-08	0.014555
109.3338	102.4638	51.94439	51.71205	49.34427	7.32E-48	3.64E-37	0.063325
9.410261	9.859393	5.904713	6.500723	5.423001	1.98E-07	2.05E-18	0.000119
11.30609	9.944666	7.110917	6.561902	6.164256	1.59E-10	1.46E-39	2.14E-11

47.385	40.84488	26.72626	28.49785	28.3454	1.06E-14	4.51E-30	1.66E-07
222.7784	245.3193	81.96266	67.45839	52.00549	3.54E-39	3.61E-45	0.061383
200.811	228.0043	131.3054	120.3077	113.9472	1.47E-19	1.5E-113	2.38E-44
119.5702	111.6083	59.50741	78.15081	82.0494	5.19E-10	8.62E-50	5.41E-08
0.08722	0.133477	0.012309	0.040653	0.075794	0.018495	0.021792	0.99617
2.75872	3.061975	1.745513	1.624968	1.88362	1.79E-05	4.35E-08	0.072444
451.762	466.0486	313.9252	298.7015	276.1098	5.27E-16	2.71E-79	1.95E-15
1.311075	1.527099	0.555067	0.611095	0.622411	4.11E-05	0.000419	0.767814
9.442768	10.93085	4.448876	3.630638	4.11886	9.74E-24	1.59E-08	4.55E-05
1.119969	1.047483	0.528802	0.520079	0.470347	3.79E-10	2.66E-15	0.01374
32.14185	31.02979	14.75375	15.55651	18.78232	1.57E-20	6.55E-12	0.034625
29.31643	30.97339	17.23328	17.39831	15.58738	1.74E-22	4.75E-23	0.803077
70.43562	71.08531	28.86122	31.25375	31.50767	2.21E-46	2.94E-18	1.48E-05
16.226	15.69277	8.444378	8.153388	8.596549	9.19E-20	2.21E-06	0.007895
7.851103	5.175674	5.681891	4.003462	3.323845	0.014849	0.023755	0.873751
1.152371	1.008521	0.716569	0.520337	0.391177	0.008551	5.16E-09	0.001351
0.160906	0.147746	0.053169	0.04756	0.037515	3.01E-05	0.00497	0.297453
25.41852	28.08276	14.78378	13.10929	13.84179	1.5E-26	1.07E-36	0.003
2.470937	2.481548	1.477639	1.468429	0.993104	9.16E-06	7.71E-07	0.753486
70.08595	86.76263	21.74031	21.35388	22.60863	1.57E-77	9.76E-43	2.64E-06
73.84676	87.57499	56.06091	50.94002	47.67022	5.91E-10	2.98E-22	0.002321
4.62442	5.60609	3.224664	2.648529	2.889214	3.07E-08	1.19E-20	1.66E-05
1.568173	0.97165	0.518177	0.33278	0.376694	8.35E-08	9.67E-05	0.133837
28.87442	34.8928	19.87818	20.41843	22.16427	8.57E-11	3.08E-13	0.089488
13.95929	13.49909	8.460001	8.675267	7.913454	3.13E-08	2.16E-10	0.017724
17.20581	21.03101	10.5219	9.356273	9.77624	6.39E-14	9.94E-27	4.4E-06
41.44873	45.61472	11.83049	9.716792	10.09264	3.9E-95	6.2E-18	4.81E-30
0.275777	0.323076	0.225459	0.124108	0.148749	0.011463	0.000603	0.275036
73.2287	79.1168	43.36173	45.32052	41.11712	5.13E-24	4.06E-92	5.79E-26
69.5067	78.11809	33.5196	38.39394	35.23805	5.36E-23	1.34E-22	0.913365
39.9001	43.10076	20.93368	21.55621	19.38967	1.14E-27	2.04E-11	0.000291
845.3326	1034.698	612.8355	580.4841	516.7201	1.39E-10	6.18E-21	0.001151
0.452808	0.483269	0.311208	0.306988	0.250403	0.000303	2.57E-06	0.249895
458.0912	555.1201	364.0794	299.0832	279.894	1.07E-08	3.3E-275	1.7E-159
2.36037	2.797813	1.090151	1.086838	0.919918	2.31E-17	7.16E-42	2.15E-07
14.13841	14.77758	7.242461	7.055498	6.613827	3.97E-22	2.67E-22	0.646567
6.168066	5.500844	2.288694	2.899732	3.242224	5.07E-15	1.16E-24	0.023691
16.70914	14.05491	7.07798	8.753709	9.603533	5.8E-15	4.58E-07	0.004359
4.755375	4.842271	3.484512	2.926903	2.569526	0.002556	0.001072	0.721865
25.91327	24.21639	7.692398	9.388049	9.722687	2.26E-45	8.53E-22	9.45E-05
0.456547	0.636573	0.221922	0.307374	0.389393	0.033304	0.021233	0.822688
15.09344	16.59377	7.261176	8.342841	7.989776	8.18E-22	1.8E-65	4.02E-17
4.491905	3.819	2.535639	2.26816	2.530032	8.91E-07	5.2E-08	0.450184
1.959834	1.885235	1.072441	0.882411	1.170158	1.53E-05	8.55E-06	0.865504
2.188572	0.873727	0.564	0.532225	1.033628	0.009354	0.029835	0.763887
6.636817	5.79392	3.278119	3.023128	3.014783	8.17E-11	2.47E-12	0.148228

Supplemental table S3

gene_name	WTvsCon_log2FC	KOvsCon_log2FC	KOvsWT_log2FC	N_CON_1_fpkm	N_CON_2_fpkm	N_CON_3_fpkm	N_WT_1_fpkm
Lhx1os	-5.19808	-0.11855	5.066204698	0.678732	0.41739	0.574047	0
Apoc1	-4.41372	0.510012	4.907504929	0.159192	0.391584	0.359037	0
0610012D04Rik	-4.91662	-0.36369	4.540820915	0.557621	0.987588	0.251528	0
Grm1	-7.95044	-3.61255	4.308971435	0.34844	0.388553	0.288149	0
Gm46545	-3.67466	0.477142	4.143913577	0.197794	0.194615	0.133829	0.036853
Ctgf	-4.05791	-0.32901	3.717912754	0.041398	0.224028	0.093367	0
Ighv1-41	-5.40726	-1.7012	3.694973313	3.599963	5.313161	5.886455	0.167686
Gm47283	-2.28652	1.370688	3.63146624	0.42149	1.762545	0.059414	0.19633
Lrrd1	-2.43397	0.644332	3.071257025	0.120405	0.296176	0.113149	0.018695
Gm42809	-3.18861	-0.18363	2.993555749	0.312229	0.307211	0.293413	0.048479
Gm20427	-2.32965	0.520954	2.838904374	0.127938	0.110147	0.072137	0.029797
Gm20763	-3.83378	-1.38921	2.437862301	0.509962	0.501767	0.754789	0.029693
Gm7269	-3.87457	-1.40698	2.437445242	0.733233	0.933641	0.826856	0
Gm16193	-4.01583	-1.64651	2.355020761	7.687864	8.088571	6.952759	0.496376
Calr4	-3.63442	-1.31806	2.302974251	0.774877	1.316914	1.297486	0.153125
Gm9733	-2.34463	-0.04424	2.287255748	17.91913	27.62529	25.71818	4.999104
She	-2.21023	-0.00212	2.196578879	0.090198	0.104885	0.064728	0.007639
Gm19688	-1.79834	0.334067	2.120656981	1.536352	1.923932	1.260015	0.390344
Serpinb10	-1.76043	0.34169	2.089616605	1.069003	1.803126	1.549927	0.490037
Mir7240	-2.9437	-0.90091	2.036870738	22.46253	27.1246	18.42229	4.755921
Lhx1	-2.63138	-0.59616	2.022485841	2.675925	3.248222	2.033601	0.487739
Phldb2	-2.18838	-0.16912	2.006068543	0.109419	0.153801	0.220341	0.036405
Gm47719	-3.87732	-1.86571	2.004814152	1.012709	2.040317	1.740217	0.179703
Prps1l1	-2.65111	-0.66513	1.973164507	1.201348	2.120721	1.394587	0.263335
Armc12	-2.65673	-0.69499	1.949677163	1.226767	1.792289	1.760699	0.103896
Slc24a1	-2.20528	-0.2452	1.948096493	0.193119	0.04471	0.166536	0.042332
Gm5150	-1.30687	0.594572	1.88891349	12.49807	17.19689	15.13992	6.003518
9530077C05Rik	-2.2413	-0.34656	1.882380477	2.853652	2.507813	2.420392	0.522603
Gm44284	-1.98871	-0.1775	1.798281948	0.343899	0.101512	0.349029	0.096113
Gm10392	-4.24233	-2.43831	1.787659529	2.085671	2.859556	2.583323	0.095558
Crtac1	-3.90231	-2.14926	1.734474528	1.47143	1.87098	0.94453	0.021089
Gm14703	-3.00685	-1.34537	1.648078225	1.431677	2.177034	1.72456	0.12125
2810454H06Rik	-1.32867	0.332253	1.647036367	0.282176	0.481245	0.424275	0.157725
Gm33424	-1.19456	0.454624	1.636867028	10.83	7.935285	11.17344	6.22526
Gm45694	-2.29456	-0.68276	1.598501802	0.236805	0.465998	0.246499	0.084849
Pi16	-2.19452	-0.59259	1.588812184	54.43668	73.2036	58.94198	12.28738
Gm9465	-2.27202	-0.68382	1.575737447	2.181767	2.760047	1.874549	0.387149
Mir6924	-2.29821	-0.70935	1.573673434	26.31973	33.29581	34.98043	3.502774
Ldhc	-2.10859	-0.52425	1.572406329	1.086938	1.253861	0.633995	0.314252
Dmpk	-2.06097	-0.48116	1.566255169	0.492355	0.551726	0.570644	0.063705
Rab11fip4	-2.61904	-1.05741	1.548691935	4.879976	6.87388	5.870546	0.925525
Bnipl	-1.90115	-0.35588	1.533214016	0.618748	0.48197	0.494242	0.096071
Prkcg	-0.95072	0.591394	1.530251901	1.606727	2.080988	1.257225	0.809507
Bmx	-2.95809	-1.42446	1.520036682	28.36478	31.94169	29.13561	4.139436
Gsdme	-1.50117	0.005079	1.493621463	6.650678	6.937063	6.069077	2.51554
Il1rl1	-0.97379	0.515193	1.476538116	0.627069	0.388789	0.503714	0.344104
Krt83	-3.35021	-1.86451	1.471305778	6.687097	7.433714	5.583195	0.68886
Mgll	-1.48207	-0.00877	1.460551104	9.214756	12.1654	11.69355	3.422914
C130060C02Rik	-1.60338	-0.15128	1.442025784	1.607531	1.530674	0.935633	0.869559
Sptbn4	-1.35527	0.082911	1.425401903	0.167199	0.255907	0.174581	0.074997
Cbx8	-1.19873	0.233636	1.420245558	0.982961	1.030585	0.817724	0.420333
Fscn2	-3.92719	-2.51209	1.391751022	3.087266	3.065266	3.038369	0.052293
1500011B03Rik	-1.05612	0.335871	1.37875659	1.594107	2.436162	2.256626	1.074304

Fbxo31	-3.02177	-1.63609	1.371886618	16.30099	19.32335	13.44875	1.73094
Gm38171	-0.85478	0.523235	1.365827409	10.40675	10.36141	14.11059	7.617434
Apln	-2.30184	-0.9336	1.355353704	0.789843	0.869667	0.954315	0.192714
2900005J15Rik	-1.61893	-0.25435	1.352366485	1.031485	1.134309	0.889499	0.395677
5430425K12Rik	-2.41374	-1.06883	1.336173371	5.31655	5.72749	4.901344	1.626862
Plppr2	-1.23195	0.106611	1.325673342	0.818304	0.402577	0.67995	0.240736
Ccl6	-0.81218	0.523269	1.322628856	382.2042	536.3222	511.329	278.1485
Rgs2	-2.07969	-0.77115	1.295793111	62.95835	70.99113	68.67727	17.02061
Myl2	-4.03378	-2.74097	1.287442578	2.399144	4.004035	3.219178	0.254625
Lins1	-2.01315	-0.73124	1.269301824	4.95004	5.523846	5.695544	1.284083
Itih5	-3.26911	-1.98817	1.268172532	1.29683	2.581479	2.079504	0.279335
Tgfbr3l	-1.24406	0.026807	1.258735919	0.949936	1.39206	0.843307	0.659012
Slc22a20	-1.83556	-0.56861	1.254393487	7.398521	8.354668	9.46971	2.471976
Syn1	-1.00927	0.253649	1.251827049	1.759169	1.377653	0.748982	0.652191
Daglb	-1.33461	-0.07163	1.250373962	1.114194	1.352088	1.074971	0.530524
Cfap45	-1.32171	-0.06471	1.244093356	0.631867	0.591136	0.817677	0.231598
Gm38379	-1.48157	-0.22957	1.239324617	3.339585	3.897247	4.335279	1.193814
Tmem38a	-2.12201	-0.87887	1.230092502	12.91104	17.24636	14.90254	3.373995
Carns1	-3.19787	-1.96098	1.223984829	24.35636	28.20872	21.74416	2.752873
Sirpb1b	-1.22333	0.009034	1.219670623	11.87681	16.06057	15.45917	6.308828
Gm11827	-1.64921	-0.42555	1.21111008	8.051758	9.668684	8.152337	3.387551
Gm17619	-1.23971	-0.02085	1.206281927	3.596484	4.584206	3.318301	1.561019
Sirpb1c	-0.90662	0.299345	1.193331995	1.737016	2.009776	1.849985	1.183684
Vipr1	-1.52001	-0.32303	1.185620779	0.265466	0.2612	0.26482	0.123654
Tmem42	-1.25235	-0.05834	1.180963219	1.323773	1.762204	1.624517	0.616613
E230001N04Rik	-1.06637	0.126308	1.179226591	1.193672	2.087979	1.645216	0.823721
Gm2822	-2.1809	-1.00448	1.160834682	0.875063	0.640744	0.734359	0.132708
Fmo5	-0.80035	0.371071	1.158682563	7.01241	7.762177	5.954378	3.849656
Card10	-1.70589	-0.54023	1.152266557	16.20401	20.2266	14.14642	5.057872
2610507I01Rik	-2.27137	-1.10667	1.15189373	28.34693	34.31794	31.42627	6.287608
Panct2	-1.54944	-0.38535	1.150904653	1.308001	2.135538	2.042326	0.749866
Rflnb	-1.49075	-0.32855	1.149292672	42.6103	51.69762	49.03979	15.88142
Gm6566	-1.46768	-0.32214	1.132002176	1.466577	1.542526	2.05304	0.471125
Ovgp1	-1.57667	-0.43624	1.127682159	2.960138	3.681613	3.769431	1.084473
Gm17552	-3.42015	-2.2812	1.126067209	9.057801	9.813976	8.267937	0.896473
Tmem156	-0.73833	0.395768	1.121394668	6.01321	6.075051	5.993926	3.634564
Sirpb1a	-1.34027	-0.21226	1.114791089	2.326384	2.492462	3.118964	0.963225
Ighv8-10	-2.43697	-1.30806	1.114274171	8.105446	8.46346	8.767314	1.386922
Ceacam10	-2.53351	-1.41169	1.109126877	32.10356	43.89549	41.1577	6.711699
Ctnnbip1	-0.6088	0.511953	1.108005951	1.845303	1.982603	1.91349	0.88918
Alox5	-1.80903	-0.68923	1.107060022	84.44459	89.70042	81.58124	23.33853
Gpc2	-2.18479	-1.06311	1.10582114	0.678931	0.681653	0.79687	0.103264
Baiap3	-1.73155	-0.62009	1.09851948	0.47496	0.775079	0.431097	0.140296
Trip6	-1.04243	0.060127	1.089777993	2.584296	3.334379	2.474382	1.567163
Sgsh	-1.13654	-0.03491	1.088365928	1.32821	2.112104	1.346509	0.69992
Gm43672	-0.64894	0.452099	1.088051835	2.736887	3.399789	3.549298	2.071614
Il18r1	-1.18539	-0.08627	1.087199068	8.126222	8.443187	10.56404	4.123049
Oscp1	-1.36325	-0.28457	1.065842593	1.465268	0.987024	1.131232	0.441013
AC140186.1	-0.62394	0.451254	1.061968213	20.67499	24.88692	19.61565	12.98796
Lpcat4	-1.31889	-0.24415	1.06187998	8.223166	10.17569	8.705399	3.400408
Ccdc189	-2.27124	-1.19693	1.060775139	2.022378	2.449079	1.832282	0.402574
Gm38192	-2.61887	-1.54829	1.059425464	4.264309	6.40408	5.929645	1.194774
Aqp9	-1.25179	-0.18203	1.057825414	15.03712	12.57333	16.47357	6.551531
S100a11	-1.59318	-0.52305	1.057386508	2536.004	2752.492	2401.757	801.1875
C1qa	-1.38875	-0.3262	1.048966049	3.017911	4.594181	3.531678	1.060936
B230208H11Rik	-3.21958	-2.15778	1.048762885	19.58436	22.22634	18.52101	2.558128

E230016K23Rik	-1.39197	-0.33598	1.043018187	1.135599	1.945014	2.015756	0.646508
Nudt7	-0.97977	0.072503	1.039955298	1.855217	1.534997	1.783041	0.824879
Tbc1d10c	-2.02319	-0.97443	1.035963336	91.01864	97.01335	81.36775	22.4064
Rgl2	-1.08859	-0.04347	1.032244514	35.10909	42.15429	35.83783	16.50886
Ggt5	-0.58587	0.457584	1.030648177	15.39984	17.69086	15.87764	10.26611
Gm29264	-1.04289	-0.00347	1.027214667	3.701212	3.964414	3.539806	1.527614
Msrb2	-1.67252	-0.63412	1.02552414	5.074719	5.162422	4.17077	1.402254
Atp1a3	-1.88744	-0.8523	1.021944991	51.60575	64.38978	47.26665	13.05603
AU019990	-1.09229	-0.06458	1.015247582	0.744229	0.993793	0.839256	0.470469
Dync2li1	-1.6146	-0.58774	1.014683677	1.610195	2.068414	1.765354	0.45835
Man2b1	-1.49092	-0.46391	1.014121731	36.65611	43.53014	39.28136	12.8814
Styxl1	-1.23141	-0.20561	1.012639238	0.769517	1.218025	0.905503	0.436363
Rbfa	-1.88241	-0.85657	1.012547978	57.75718	73.16252	58.48777	15.50493
Fbxo10	-2.75698	-1.73339	1.012324649	4.266926	4.633093	4.398907	0.776196
Gm11361	-1.451	-0.43606	1.000733984	10.34144	3.647729	1.430232	1.181537
Cebpd	-0.92755	0.080462	0.995635967	43.9798	45.2951	49.02489	21.19267
Polg2	-1.74695	-0.74057	0.994635427	3.493889	4.053054	4.016661	1.177844
Krt86	-1.99515	-0.99154	0.99185595	11.70511	11.90287	10.64819	3.372513
Epha1	-1.63808	-0.63583	0.991053075	1.839299	2.017414	1.904137	0.716294
Mcmdc2	-1.81149	-0.81092	0.986068807	0.420422	0.610648	0.541832	0.177181
Tmem40	-1.65658	-0.66324	0.980396943	22.5255	24.53232	20.22148	6.599619
Gm7665	-1.50142	-0.51064	0.978087759	137.7798	155.1843	136.4847	47.73856
Mmp8	-1.31339	-0.32425	0.976772971	857.9118	902.5121	968.5559	418.2355
Nlrp12	-1.77773	-0.79075	0.974410372	20.03805	21.8552	20.81756	6.390647
Cyb5d2	-1.33712	-0.35706	0.967145873	0.748446	0.872372	1.038784	0.407624
Adam8	-0.75034	0.228287	0.966234246	194.6655	166.1847	165.181	106.0671
Rgs18	-2.23784	-1.26322	0.962671246	12.68362	15.75034	17.92001	4.210313
Ifitm10	-0.91357	0.056311	0.957392757	0.729577	1.082861	0.585674	0.276477
Ccno	-1.48266	-0.52454	0.945825468	10.40685	9.578247	6.743425	2.634278
Mettl7a1	-1.33108	-0.37737	0.940679249	4.609756	6.062532	4.966743	2.019663
Kiss1r	-2.40919	-1.45775	0.938181056	2.334374	2.886184	2.441933	0.51506
Arl11	-1.65063	-0.70482	0.933150922	17.81462	23.95804	23.91145	7.376033
Zfand4	-1.70654	-0.76157	0.932351535	9.093242	10.68914	9.71125	3.325164
Gm12854	-1.31294	-0.3697	0.930364265	90.52391	102.8022	72.17725	36.59337
Tiam2	-1.81852	-0.87759	0.928737546	1.491988	1.65094	1.651044	0.497951
Gm26642	-1.16155	-0.22405	0.9247393	2.022229	2.115397	2.064396	0.793223
Gab3	-1.28441	-0.34995	0.921808326	0.806638	1.153074	0.875308	0.510427
5830432E09Rik	-0.91035	0.019211	0.91683136	2.964845	3.346196	2.993774	1.67889
Mvb12b	-1.43349	-0.50632	0.914228806	6.090633	8.145874	7.044035	2.03861
Krt7	-2.90544	-1.97729	0.9134548	1.428188	1.892935	1.383175	0.195661
Tlr5	-3.902	-2.97654	0.910703294	5.105972	6.228193	6.09253	0.437664
Tacc2	-1.0948	-0.17243	0.90993428	0.122989	0.124794	0.134359	0.060869
Mkl1	-1.92963	-1.01127	0.905447007	26.57802	30.45853	27.5905	6.695317
Sfn	-0.6173	0.296333	0.900748573	9.729161	12.53495	10.35041	6.498481
Hhex	-1.64948	-0.73771	0.899099092	33.7779	38.79448	38.36937	12.79983
Pdzd3	-0.77502	0.136087	0.898295139	1.214618	1.16854	1.400145	0.678922
Aacs	-0.70088	0.197646	0.885682065	14.10571	15.51184	13.98342	8.402704
Gm48529	-1.06716	-0.17181	0.882608772	1.214278	1.033929	1.421989	0.522101
Cd300lb	-1.72299	-0.83007	0.88023483	132.731	142.0836	140.3382	43.00775
R3hdm4	-1.96232	-1.0704	0.879014296	53.39713	63.25548	54.08002	13.55506
Mfsd6	-1.39198	-0.50272	0.876817065	12.49715	13.76875	14.89607	5.301717
Cd300ld	-0.76281	0.123303	0.873639686	51.78618	49.05939	50.01618	31.09887
Gm11346	-0.83503	0.048785	0.871467412	1.502883	1.364982	1.417748	0.794278
Tmc6	-0.77572	0.105949	0.86870409	16.06784	18.8291	16.51972	9.938725
Gm38158	-1.47734	-0.5974	0.867259466	18.69166	19.9382	18.91138	7.811496
Tsc22d3	-1.78954	-0.91108	0.865892234	17.57074	19.46282	20.99881	6.481463

Rab23	-2.00761	-1.12858	0.864933389	2.208665	2.55998	2.32947	0.579321
L1cam	-1.7606	-0.88343	0.864341028	4.15108	4.052644	3.926679	1.201441
Mib2	-1.55709	-0.68165	0.862669399	10.83098	11.43398	8.736356	3.153164
Pithd1	-1.15631	-0.28172	0.861949853	13.22096	17.21919	16.85023	6.793076
Fam193b	-0.67922	0.195046	0.861341621	13.67075	15.46062	12.20457	7.696845
Evi2	-2.23668	-1.36458	0.858888144	5.480496	7.002757	7.314734	1.622608
1600010M07Rik	-1.65875	-0.78828	0.85697446	7.877651	8.762055	8.458646	2.871707
Col11a2	-1.41613	-0.5469	0.856440355	1.820534	2.183651	2.013885	0.775318
Atp2a3	-0.89666	-0.028	0.855684154	37.26302	42.99737	36.22932	19.97397
Stfa1	-0.85014	0.016332	0.853700346	11.92728	15.83896	17.21252	9.7905
Ear2	-2.15205	-1.28679	0.852253611	8.401081	8.914386	10.03105	2.762271
C5ar2	-2.05019	-1.18917	0.848031895	17.14552	18.04809	18.20954	4.182967
Ccdc125	-1.73146	-0.8714	0.847199311	24.89661	33.70038	30.15821	8.848441
Cnnm3	-0.98694	-0.12698	0.847007144	3.735802	4.58336	3.900749	2.1655
D330041H03Rik	-0.75198	0.108445	0.847003155	1.010947	1.740725	1.14003	0.912366
Smpd2	-0.95133	-0.0916	0.846952217	3.538849	3.573263	3.333062	1.666914
Dnmbp	-1.34397	-0.48448	0.846773021	3.267672	3.96254	3.337425	1.381888
Tmem220	-0.75465	0.104112	0.846548871	2.093435	1.675763	1.160362	1.090813
Gm33326	-1.49657	-0.63838	0.8452388	1.226299	0.931672	0.997774	0.375986
Dym	-1.11531	-0.25789	0.844448125	9.514405	16.50642	12.91834	5.581616
Dnah14	-1.84581	-0.98805	0.844220236	0.383002	0.577831	0.518287	0.139323
Rin3	-1.35407	-0.49743	0.843827205	86.93596	98.16418	86.7027	33.27071
Rassf5	-1.55033	-0.69442	0.843287295	47.47033	55.33752	54.80137	19.00608
Paqr7	-0.88973	-0.03573	0.840939073	6.229229	6.977344	6.083812	3.523335
Scrg1	-2.5286	-1.67453	0.840302534	37.64661	50.77611	43.97994	7.408367
Phospho1	-0.66498	0.186219	0.838516264	6.187536	7.007053	6.154762	4.078513
Slco4c1	-1.91306	-1.06268	0.838187216	6.768964	7.788017	8.436044	2.236872
Frat1	-1.57049	-0.72073	0.836467615	11.62186	14.39974	11.03646	3.608969
Nacad	-2.9599	-2.11232	0.834845308	4.103715	6.324638	4.723255	0.608976
S1pr4	-1.925	-1.08008	0.831932914	48.74555	56.60706	45.95729	10.52043
F2rl2	-0.728	0.115833	0.831454691	2.556232	2.23569	2.562333	1.256841
Aicda	-1.98301	-1.14021	0.831288289	5.825955	5.666437	5.940548	1.80915
E230014E18Rik	-2.95516	-2.11189	0.830725655	52.60115	61.38794	59.23979	8.670283
Ankrd13d	-1.20773	-0.36531	0.829840589	9.89397	10.63503	10.75008	4.036733
Myadm	-0.73245	0.108807	0.828819957	54.72506	58.57981	58.79097	35.40401
Grk4	-1.27822	-0.43701	0.828483186	0.723534	0.675707	0.539336	0.20564
Dhrs7	-2.09569	-1.25561	0.827333899	53.41619	59.28789	53.39071	12.96254
St8sia4	-1.19289	-0.35838	0.821911017	2.632169	3.346816	3.224199	1.534981
4732465J04Rik	-2.08826	-1.25322	0.821831201	2.600488	2.382233	2.568493	0.651599
Mtus1	-1.43952	-0.60585	0.820986858	25.19674	28.7985	27.69833	10.5542
Pqlc1	-1.57765	-0.74459	0.820309961	10.78569	12.72337	12.27234	3.816588
Rgs14	-1.57801	-0.74608	0.819154149	36.48998	42.37773	39.65259	12.92345
Retnlg	-1.85518	-1.02373	0.818758946	4930.688	7696.441	7867.523	2026.728
Pip5k1b	-0.6547	0.175438	0.817563329	2.773845	3.35021	2.763915	2.201278
Gm31583	-0.71005	0.118512	0.815840329	7.793777	9.110468	9.634015	5.895417
Trmt12	-0.6669	0.1585	0.812676397	2.14062	1.896904	2.144063	1.176701
Lrrc56	-0.95918	-0.13498	0.811996863	0.702669	0.681774	0.440216	0.336394
Slc9a9	-1.72683	-0.90184	0.809864792	0.65695	1.138882	1.011296	0.184576
Rnf125	-1.1161	-0.29487	0.809459811	3.640172	2.533377	2.052484	1.364743
Paxx	-0.81083	0.010942	0.80888258	14.21724	17.96539	15.96792	9.452912
Trp53i13	-2.1754	-1.35551	0.806537413	13.67235	16.09992	10.68122	2.250739
Fcer2a	-0.71236	0.104898	0.804738431	2.547276	2.242514	2.138196	1.33818
Cep19	-1.43283	-0.61666	0.803911214	15.79409	17.30393	19.33631	6.710983
Mical1	-1.0603	-0.24378	0.803761498	25.98132	28.97484	26.7808	13.29267
Mpzl1	-1.1201	-0.30524	0.802288186	0.900802	0.755018	0.677214	0.31081
Ndufb2	-0.88532	-0.0717	0.801198276	0.515592	0.496952	0.522096	0.362694

Tbx6	-1.3735	-0.55931	0.801129765	14.04983	16.95705	15.10342	5.688124
Il6ra	-1.35867	-0.54558	0.80039763	21.59226	24.48833	23.69243	8.637264
Padi3	-1.13489	-0.32358	0.79796398	0.962079	1.064944	0.871812	0.384116
Rdh14	-0.67146	0.134949	0.793843879	2.920956	3.346454	2.932941	1.789255
Rara	-1.01696	-0.21189	0.792171208	57.59136	62.77957	52.72089	25.80858
Cyb5r1	-0.80074	0.003414	0.791521735	19.01907	22.18032	21.07125	11.74992
Dusp7	-1.45971	-0.65601	0.790841458	5.964288	7.396313	6.785536	2.268557
Emb	-0.77716	0.025881	0.790552891	52.98531	56.71521	56.2889	33.86332
Gm40787	-1.8841	-1.08454	0.784716126	19.24163	24.6305	26.12246	4.872944
Ipcef1	-1.99692	-1.20179	0.782700653	15.68791	18.16698	20.99928	5.006422
2610035D17Rik	-1.60867	-0.81487	0.780471929	3.462142	5.466886	3.967177	1.144471
Galm	-1.35748	-0.56727	0.778500453	2.774414	3.035755	2.58924	1.515134
Aldh4a1	-0.75165	0.035542	0.7742698	1.520612	1.994899	1.469804	1.0231
Ppm1m	-1.24373	-0.45689	0.773839976	39.85836	47.53868	37.30854	16.81525
Crot	-1.07666	-0.29117	0.773176315	2.843746	2.65921	3.280289	1.42573
1700047M11Rik	-2.49752	-1.71333	0.771409114	43.82151	48.68605	44.63005	8.316011
Frat2	-1.90552	-1.12174	0.770832358	8.166301	11.27065	8.80728	1.914693
Fut7	-0.9273	-0.14405	0.770344328	6.739191	7.546343	7.17433	3.654486
Dok3	-0.77254	0.007672	0.767432762	174.8164	193.8306	176.1178	99.11967
Mast3	-1.38134	-0.60143	0.766801617	26.36282	31.46748	23.59477	8.254459
Ppcdc	-0.93696	-0.16235	0.761675861	4.316698	5.522526	4.706764	2.538349
Cyp4f18	-2.06353	-1.29104	0.759700535	102.6044	132.6807	118.3334	28.58065
Vamp5	-0.59223	0.18016	0.759484398	9.756869	11.14985	9.300468	6.317803
Prkab1	-1.02299	-0.25235	0.7578986	36.52441	37.9329	36.35264	16.44624
Eepd1	-0.7078	0.062406	0.757528537	7.808484	8.75504	7.05351	4.774394
Arhgap45	-1.49805	-0.72848	0.756688739	262.9298	313.5368	273.3868	90.89651
Dtd1	-1.53894	-0.77197	0.754185904	3.624136	3.887813	4.058503	1.359874
Sytl1	-1.49219	-0.73083	0.748705654	3.363134	3.910737	3.129522	1.029757
Svil	-0.88299	-0.12183	0.748502766	24.55991	25.10721	25.14344	13.01241
Lamtor4	-0.84138	-0.08015	0.748380775	60.44744	70.03756	62.36979	32.03343
44083	-0.6893	0.070725	0.747080387	23.69558	28.48299	24.60253	14.65817
Tcp11l2	-1.54114	-0.78232	0.746176448	41.71883	47.74288	43.91839	15.03579
Prickle3	-1.19686	-0.43962	0.744543827	14.41222	15.65453	14.27709	6.111097
Dynlt1f	-1.50184	-0.74491	0.744030599	7.592229	8.932126	7.751509	2.798742
Gapt	-1.02409	-0.26805	0.743663654	19.06631	22.45587	22.39933	11.26703
Agap2	-0.7233	0.032748	0.743394158	5.093605	4.668475	4.485027	2.71154
Arhgef4	-2.70194	-1.95181	0.738792205	1.195077	1.737217	1.340899	0.223785
Cnn2	-0.79571	-0.04549	0.737448969	246.8678	283.5219	261.4496	138.6169
Khk	-0.8429	-0.09414	0.735907316	7.876145	11.12176	8.212888	4.083158
AC133488.1	-1.92908	-1.18369	0.73398366	2.02002	1.970568	1.810675	0.482525
Cnr2	-1.27935	-0.53715	0.729589286	26.54611	28.82655	28.37598	11.42998
Izumo4	-0.67737	0.063385	0.728280606	4.041051	3.913655	4.055998	2.404637
Ptov1	-0.5986	0.141824	0.727512757	9.263132	10.69093	9.431423	6.377202
Igf1r	-1.15492	-0.41561	0.726779261	9.568352	9.879581	9.851473	4.685281
Gcnt1	-0.99945	-0.26167	0.725285069	14.98821	18.96236	20.43821	9.516623
Jaml	-3.04898	-2.31128	0.725122515	90.79162	97.65646	90.02948	12.60289
Lgals4	-1.32678	-0.589	0.724528598	5.499856	6.846136	6.230953	1.858815
Chac1	-0.66219	0.072731	0.722629636	3.147273	2.420404	3.100392	1.280641
Tecr	-0.79455	-0.05865	0.722486198	42.56985	51.32661	35.53922	22.86315
Gm44103	-0.9394	-0.20479	0.722180434	2.150786	2.259423	2.297756	1.129888
Acadm	-0.6411	0.093917	0.722093019	4.192121	6.105224	5.290583	3.186334
Satb1	-1.83153	-1.09774	0.720722601	3.775325	4.792605	3.773027	1.171396
Gm35339	-0.91956	-0.18729	0.720115026	1.511755	1.708807	1.948323	0.99758
Tnfsf14	-2.35111	-1.61941	0.718394696	65.696	85.05123	62.87593	14.09833
Rmnd5b	-1.52288	-0.79569	0.71414525	30.88396	37.04756	29.54302	10.08405
Slc12a9	-0.65989	0.067143	0.7140218	6.464586	7.463368	5.893208	4.088198

Fam117b	-1.59885	-0.87883	0.707128747	12.14444	14.42704	12.10116	4.268195
Smarca2	-1.37831	-0.66956	0.695859366	5.635989	5.851676	4.783096	2.072354
S100a6	-0.89532	-0.1878	0.695158991	832.2338	917.8756	966.0502	462.9811
Osbpl2	-1.10735	-0.39976	0.694525511	18.42915	23.96392	19.71366	9.50003
Gm34589	-2.04964	-1.34655	0.692542951	2.700354	2.142042	3.564476	0.819047
Avpr2	-1.0829	-0.38011	0.689797213	2.257149	2.180495	2.19826	1.013146
Mroh2a	-1.15792	-0.45494	0.689788384	3.368225	4.103164	2.909668	1.585158
Hgf	-1.59529	-0.89386	0.689389071	1.087804	1.248709	1.756412	0.483666
Mogat2	-0.81523	-0.11366	0.688179719	2.355867	4.508997	3.74846	2.31498
Agpat2	-1.04927	-0.34958	0.68694887	27.4411	32.17032	30.85086	13.31301
Tpgs1	-0.58519	0.113543	0.686137193	14.23462	15.08642	11.66997	6.453399
D2hgdh	-1.59945	-0.90121	0.685678772	2.628284	3.429912	2.605091	1.06961
Tpcn1	-0.71888	-0.02355	0.682386751	4.058998	4.902692	3.961708	2.496999
Ap1m1	-0.75996	-0.06677	0.680407142	35.71018	39.14401	35.17319	20.06449
Riox1	-1.2822	-0.58916	0.680342105	8.135086	10.78847	11.28208	4.424792
Fam160a2	-0.77117	-0.07988	0.678449692	8.32194	9.384669	8.00196	4.755118
Cyp4f13	-0.61781	0.073303	0.678145301	8.393859	11.18109	9.024435	6.063585
6430548M08Rik	-1.33112	-0.64227	0.675999126	74.48451	83.73449	77.52461	30.83152
Gm16556	-1.57181	-0.88642	0.672748204	51.49018	57.37296	53.57847	22.13101
Gm20605	-1.33053	-0.64544	0.672588812	2.971582	3.276251	3.395888	1.186423
Atxn10	-0.59624	0.085523	0.669259817	96.18182	103.4198	101.7744	67.786
Tmc8	-0.82716	-0.14576	0.668371463	14.99863	16.72145	13.26438	7.816768
Ypel3	-0.72414	-0.04296	0.668222218	244.8568	286.9872	228.1678	147.2658
Celsr3	-1.25778	-0.57915	0.665516458	6.053013	6.491896	4.777571	2.323277
Fbxo9	-0.832	-0.1551	0.664160509	8.988827	11.15404	10.22265	5.511535
Ankrd9	-1.29936	-0.62407	0.662473937	2.661124	3.41124	2.831845	1.169716
Mpzl3	-2.30356	-1.62876	0.662181409	13.10336	14.21041	14.49928	2.921485
Bbs9	-0.623	0.047051	0.657034029	1.024458	1.13866	0.641815	0.715788
Mon1a	-0.73662	-0.06743	0.656259201	7.774378	8.164896	6.593011	4.158149
Purg	-0.97444	-0.30889	0.652458775	3.538171	4.838429	3.212242	2.095013
Dnajc28	-1.25151	-0.58722	0.652439393	1.910303	1.405616	1.742108	0.82018
Abtb1	-1.05631	-0.39377	0.649639593	108.6338	121.2691	99.38832	49.59735
Ccdc180	-1.20313	-0.54102	0.649288918	1.264511	1.761683	1.564781	0.646345
Slc12a5	-1.01044	-0.34919	0.649234694	0.699786	0.625945	0.744301	0.311143
Tatdn3	-0.67284	-0.01113	0.649124085	1.254108	1.254867	1.246447	0.65347
Rnpepl1	-0.78484	-0.12387	0.648305569	11.15954	12.05729	11.54045	6.229569
Ttll3	-1.11025	-0.44938	0.647933194	6.2262	7.097073	6.160103	2.801663
Pigb	-1.78823	-1.12809	0.647592117	4.381576	4.964535	5.587469	1.432884
44079	-1.3785	-0.71902	0.646345292	5.218566	6.608134	4.980843	2.226476
Plekhg3	-1.12025	-0.46536	0.642190757	16.80584	18.33938	17.65902	7.832853
Il17ra	-1.27168	-0.6224	0.636585105	81.92402	84.81168	83.08668	31.97262
Ager	-1.39765	-0.75001	0.635324787	12.5575	11.72974	11.35367	4.328977
D1Ertd622e	-1.54261	-0.89634	0.634152505	2.937754	3.369948	4.053003	1.172154
Cep164	-0.77585	-0.12965	0.633585909	1.729898	1.731242	1.496489	0.899609
Acrbp	-0.83013	-0.18455	0.63269709	4.409377	4.783034	3.831185	2.441092
Mcu	-0.7263	-0.08383	0.63034203	4.157993	4.233983	5.102917	2.680486
Zdhhc3	-0.93107	-0.28807	0.630143979	5.193924	6.273079	5.80342	3.064479
Slc2a4rg-ps	-0.70318	-0.06046	0.629912799	4.735959	5.266802	3.994283	3.04021
D930048N14Rik	-1.36202	-0.72769	0.622152315	2.055149	2.073099	2.23966	0.788354
Hgsnat	-1.17366	-0.53995	0.621116095	37.86099	37.41119	38.29036	15.75771
Thap6	-2.04156	-1.40889	0.620986926	6.210919	5.516968	6.144678	1.794757
Nqo2	-1.00745	-0.37617	0.618407924	1.802074	2.5948	3.139128	1.351237
Wdr45	-1.04186	-0.41355	0.615824777	6.566016	7.09422	6.294748	3.466794
Acvrl1	-0.85423	-0.22728	0.614702203	23.28509	19.90527	22.1872	12.98695
Coa4	-0.67176	-0.04546	0.614065421	5.04954	5.07929	3.940246	2.079064
Cited2	-0.59694	0.02851	0.612513611	37.40679	38.77397	32.04501	22.70067

Rxrb	-0.65006	-0.02493	0.612410619	16.99074	19.12843	17.08346	9.965571
Calcoco1	-1.00356	-0.38015	0.610371272	8.059871	9.679082	7.411028	3.629139
Ramp1	-0.75261	-0.12936	0.610314747	7.838436	8.657241	6.673662	4.326598
Ikbkap	-1.29547	-0.67396	0.608827601	7.595512	9.072084	8.520218	3.392587
Rab3d	-1.74868	-1.12929	0.606358552	62.97352	79.79171	67.06729	20.03744
Runx2	-1.12723	-0.50817	0.60603637	7.056468	7.655173	6.408298	3.321234
Nipal3	-1.30824	-0.68907	0.605876719	3.293434	4.446096	3.458732	1.337775
Cdk5	-0.61486	0.001845	0.60388958	9.177912	11.69116	9.025267	5.598299
Abcd2	-2.03712	-1.42292	0.601504009	15.97509	19.33436	20.60928	4.286568
Crispld2	-1.78541	-1.17134	0.601406473	49.97139	48.11931	48.99869	13.09636
Eif4ebp2	-0.84269	-0.22887	0.600836848	34.9337	43.10639	36.23067	20.30673
Timm10b	-0.72039	-0.11063	0.597257585	1.967366	2.08215	1.67791	0.985705
Gba2	-1.10218	-0.49321	0.596025308	6.303871	8.114042	6.974653	2.930182
Capn1	-0.75723	-0.15026	0.594055651	116.8794	136.8651	115.558	68.93492
Cd177	-1.57228	-0.96606	0.593438589	356.0461	429.5189	396.8285	119.9142
Tcea2	-0.84357	-0.23721	0.59342842	4.204927	4.654518	3.655163	1.999457
Atrn	-0.83101	-0.22504	0.593324506	5.075435	5.280618	4.954967	3.003202
Hectd3	-1.18673	-0.58085	0.592917844	9.618166	10.94971	9.292903	4.131179
Gsn	-0.59022	0.015329	0.592772719	279.9649	269.1165	214.6632	169.368
Padi4	-1.96764	-1.36416	0.590567351	196.3977	229.1902	189.652	50.12535
Dhrs9	-2.69345	-2.09182	0.589442193	33.64203	34.85716	39.76274	6.881588
Preb	-0.73694	-0.13665	0.587483734	36.48124	36.30717	29.34688	19.98899
Maml3	-0.60754	-0.00769	0.587240594	0.547805	0.739043	0.566803	0.315671
Milr1	-1.16732	-0.56768	0.587151819	34.13373	37.98939	36.46762	16.90294
CT010583.2	-0.65793	-0.05842	0.586688269	0.625328	0.679203	0.723489	0.332888
Adpgk	-2.82584	-2.22788	0.585367379	115.0492	114.9587	114.6234	16.08728

N_WT_2_fpkm	N_WT_3_fpkm	N_KO_1_fpkm	N_KO_2_fpkm	N_KO_3_fpkm	KOvsWT_p	WTvsCon_p	KOvsCon_p
0	0	0.66229	0.486094	0.377615	0.001592	0.001115	0.883871
0	0	0.759418	0.380033	0.141707	0.003876	0.016967	0.611461
0	0	0.483656	0.21299	0.694925	0.010815	0.003901	0.708059
0	0	0.040296	0.007394	0.034463	0.022841	2.02E-10	4.06E-10
0	0	0.068623	0.491073	0.176069	0.010762	0.030256	0.616558
0	0.019469	0.089766	0.177889	0.018425	0.031503	0.013785	0.742704
0	0.169301	1.561225	0.687525	2.243192	0.006439	2.42E-06	0.001835
0.215877	0.049555	5.209546	0.352173	0.187598	0.014519	0.022122	0.404972
0.061669	0.018875	0.348114	0.191626	0.285815	0.000923	0.02265	0.32201
0	0.048946	0.315949	0.298149	0.18529	0.040907	0.027472	0.830922
0.016382	0.015042	0.208065	0.106898	0.128122	0.007731	0.047649	0.483099
0.032649	0.059957	0.138225	0.365224	0.170231	0.033378	4.55E-05	0.025193
0.176727	0	0.336692	0.329491	0.268758	0.04382	5.46E-05	0.015532
0.623769	0.286376	2.11267	2.471289	2.642527	1.11E-05	5.94E-21	2.83E-09
0.072159	0.044171	0.529529	0.313911	0.50165	0.000999	4.45E-10	0.000876
4.417101	4.506482	25.84837	21.96074	20.81305	6.17E-28	7.41E-25	0.807917
0.033598	0.015425	0.106683	0.086131	0.065693	0.015087	0.016762	0.997333
0.572278	0.394105	2.543986	2.267291	1.118951	0.006224	0.028469	0.575947
0.538827	0.271319	1.633649	1.911969	2.024015	3.16E-12	2.27E-07	0.159579
1.045888	2.88104	9.741504	18.52464	8.179916	0.016839	5.31E-05	0.087964
0.491609	0.300934	1.778577	1.555373	1.903022	3.6E-13	8.46E-22	0.004094
0.024018	0.044107	0.128801	0.186581	0.111316	0.002586	0.001064	0.730779
0.049399	0.090717	0.209138	0.368397	0.729768	0.040183	1.07E-07	0.000465
0.180971	0.299106	1.164584	0.910991	0.88068	0.000211	1.25E-07	0.072086
0.22848	0.419587	0.870581	1.206945	0.860383	0.000754	3.68E-07	0.059673
0.011637	0.032055	0.078825	0.097629	0.161796	0.030012	0.016427	0.719359
6.551244	5.464397	23.84015	20.559	22.85911	1.52E-35	1.9E-13	5.32E-05
0.587128	0.527637	1.914526	1.968032	2.203696	4.47E-17	5.3E-25	0.042181
0.052841	0.048519	0.238626	0.180615	0.275514	0.015182	0.010857	0.763036
0.105072	0.192957	0.533809	0.228546	0.608718	0.020406	6.79E-16	1.67E-10
0.162321	0.10646	0.274885	0.367481	0.322416	0.02034	1.67E-12	4.82E-08
0.233314	0.306045	0.564443	0.683558	0.839965	0.005109	4.19E-10	0.000212
0.115619	0.194632	0.668976	0.377232	0.435385	0.002118	0.020864	0.459842
2.124333	4.551371	20.18597	11.4418	9.025139	0.00016	0.005389	0.190681
0.055978	0.0514	0.205394	0.139154	0.243225	0.045021	0.001911	0.234723
13.29776	14.90645	40.42667	41.79825	40.73613	2.32E-61	1.18E-79	5.65E-08
0.532119	0.488597	1.261579	1.488129	1.47972	0.024073	0.000404	0.165518
7.70305	7.957159	24.45919	15.25923	17.57167	0.008496	1.63E-05	0.084862
0.268754	0.10576	0.780217	0.715809	0.567187	0.012173	0.000449	0.250098
0.168115	0.154365	0.39146	0.404854	0.353057	0.002237	1.78E-05	0.178271
0.960684	0.964342	2.55753	2.732198	3.127102	4.8E-22	4.17E-63	2.19E-13
0.184863	0.145494	0.402506	0.541609	0.298344	0.019991	0.002345	0.468313
0.9069	0.832726	2.346376	2.348376	2.729155	6.75E-12	0.000444	0.00708
4.375006	2.936326	11.71146	9.162678	12.22402	2.14E-19	4.6E-111	5.68E-32
2.289922	2.093891	6.199834	6.590419	6.822089	9.34E-45	2.84E-44	0.958865
0.237578	0.185829	0.759959	0.639805	0.757004	1.56E-06	0.005985	0.055773
0.658648	0.57454	2.426001	1.657785	1.287826	9.73E-05	3.57E-35	5.17E-15
4.205801	4.136093	9.590969	11.02687	12.05478	4.43E-29	5.77E-28	0.945897
0.212475	0.243871	1.43928	1.436005	0.784724	0.018571	0.007414	0.732127
0.069777	0.087368	0.230959	0.189226	0.209471	0.001148	0.003753	0.823001
0.412663	0.394069	1.090181	1.061734	1.161882	4.68E-06	0.000357	0.382291
0.402493	0.158389	0.413836	0.670011	0.524652	0.035793	1.06E-20	1.56E-16
0.903322	1.02085	2.235788	2.720566	2.928612	3.88E-06	0.001463	0.2166

2.290575	1.991467	6.258911	4.827597	4.596457	1.1E-16	1.49E-95	2.27E-29
6.852975	4.661097	10.85312	19.75662	19.18915	1.68E-06	0.002212	0.039005
0.192637	0.141506	0.440405	0.466901	0.451986	0.007879	2.52E-07	0.008435
0.227895	0.361442	0.894665	0.7339	0.918192	0.000771	3.98E-05	0.414675
0.675784	0.657013	1.649312	2.779271	3.143556	0.000858	1.18E-14	1.89E-05
0.132352	0.425346	0.877862	0.575771	0.575073	0.007937	0.019484	0.79384
260.2531	270.5257	732.5206	669.9863	639.6578	6.27E-60	1.21E-13	7.43E-06
17.37726	13.2378	41.96096	36.32725	39.6925	3.13E-29	2.92E-85	2.37E-16
0.124434	0.199949	0.500473	0.637988	0.297367	0.048252	1.23E-20	2.43E-15
1.360401	1.334304	3.263718	3.34336	3.071908	1.53E-16	1.15E-44	1.27E-08
0.215003	0.119861	0.370603	0.52254	0.600551	0.000419	1.11E-28	1.41E-15
0.372665	0.304164	0.981707	1.119398	1.133463	0.000724	0.001773	0.929965
2.430298	2.114079	6.200539	6.021549	4.668489	1.99E-09	6.86E-22	0.001104
0.698738	0.574054	1.712662	1.371298	1.533993	6.77E-06	0.003196	0.379773
0.475553	0.390081	1.02546	1.075771	1.25079	1.69E-09	2.3E-10	0.687021
0.275879	0.30203	0.655867	0.633047	0.645452	9.35E-05	6.86E-05	0.811178
1.233119	1.680135	2.997654	3.522718	3.283886	1.29E-05	1.68E-07	0.320045
3.936485	2.990436	8.177042	7.629614	8.539734	2.16E-13	6E-39	5.55E-10
2.537755	2.751316	6.679429	6.042524	6.243966	1.44E-18	4.8E-140	8.25E-61
6.009222	6.137274	13.46148	13.9161	16.01422	3.94E-23	5.02E-19	0.946186
2.394533	2.402271	6.608272	5.869842	6.666446	8.29E-08	3.21E-14	0.016485
1.590847	1.691377	3.863843	3.668459	3.747168	3.86E-06	7.95E-06	0.928111
1.12031	0.665618	2.148316	2.119432	2.577051	1.38E-06	0.000441	0.134088
0.062753	0.086431	0.177118	0.243745	0.209042	0.03123	0.004092	0.450303
0.917301	0.439449	1.587194	1.449972	1.455641	0.004303	0.003118	0.861242
0.6793	0.831655	1.936466	1.794198	1.613524	2.9E-05	0.000766	0.63452
0.229305	0.133987	0.458925	0.272057	0.380418	0.047611	1.15E-05	0.014798
4.190187	3.798404	8.462663	8.848533	9.354013	2.08E-25	5.3E-10	0.002111
5.108209	5.24157	11.48228	12.12741	10.96455	8.69E-28	7.06E-40	1.9E-05
6.735212	6.348175	13.29429	15.38463	14.76796	4.03E-14	1.16E-61	4.74E-18
0.456434	0.648933	1.458646	1.523526	1.189926	2.79E-05	3.76E-08	0.102437
19.22445	15.59032	41.63842	36.48628	35.30345	3.82E-25	1.14E-43	0.001784
0.828851	0.523229	1.272045	1.255569	1.485564	0.018645	0.001855	0.401086
1.209483	1.173128	2.567493	2.874291	2.205711	1.41E-07	1.15E-14	0.017674
0.923144	0.703974	1.603064	1.969074	1.985141	2.51E-06	6.22E-87	1.44E-51
4.491409	2.6596	6.628151	8.202923	8.825663	1.03E-09	2.7E-05	0.005369
1.429822	0.729377	1.950543	2.320208	2.531057	0.00018	4.87E-06	0.363955
2.202792	1.089108	3.156465	3.63303	3.386719	0.040154	3.27E-08	0.000245
6.568961	6.813583	17.57922	12.39842	13.74255	2.85E-11	2.47E-71	7.07E-21
1.54261	1.316693	3.145879	2.511484	2.473377	1.16E-05	0.023607	0.017943
24.74623	24.41797	50.21341	53.90463	53.52384	2.33E-42	1E-116	2.3E-16
0.241284	0.130323	0.252375	0.304311	0.468688	0.036643	3.87E-07	0.003181
0.20173	0.163439	0.311481	0.38717	0.391857	0.011793	4.38E-05	0.081724
1.148798	1.330015	3.023917	2.816969	2.864711	3.49E-06	3.78E-05	0.775275
0.604691	0.85809	1.629135	1.575785	1.445065	6.42E-06	2.83E-05	0.878526
1.99752	2.059392	4.599345	4.442895	4.111223	8.93E-07	0.009584	0.034125
3.475727	4.232145	7.272254	8.593273	9.531821	4.41E-15	5.9E-18	0.529245
0.542652	0.402856	1.319791	0.817989	0.782598	0.000426	8.34E-07	0.277413
14.98852	14.08742	31.63471	31.69541	25.28169	1.46E-15	4.86E-06	0.000921
3.56706	3.82778	8.460643	7.031054	7.24528	2.62E-12	1.27E-18	0.083365
0.407243	0.487742	1.094452	0.742761	0.892432	0.002147	2.29E-15	1.88E-06
0.821082	0.663456	1.696384	2.326863	1.626831	0.002216	6.95E-21	8.9E-11
6.398523	5.431534	12.80698	12.55732	13.24555	9.68E-21	4.69E-23	0.122312
830.9797	899.486	2011.048	1649.103	1657.583	3.06E-27	9.48E-85	1.41E-07
1.45821	1.71385	3.407823	2.06621	3.345376	0.004284	6.42E-05	0.284297
2.069815	1.803066	4.673566	3.908454	4.842587	0.000136	3.94E-58	2.7E-36

0.667794	0.613176	1.16737	1.305284	1.535025	0.000742	1.61E-05	0.23354
0.928603	0.852654	2.011418	1.529981	1.857881	3.59E-07	1.69E-06	0.6973
22.18735	21.25983	45.46237	45.00109	45.81829	6.98E-29	1.39E-97	4.19E-24
18.41487	17.92038	34.62786	35.60441	38.84384	6.22E-30	3.28E-29	0.662142
10.7351	11.40902	21.64031	22.99388	22.20938	2.69E-31	1.04E-09	1.15E-06
1.487743	2.379593	3.128996	3.400092	4.587541	0.000793	0.000459	0.989026
1.894296	1.21351	3.096032	3.080006	3.062601	0.000546	1.56E-09	0.005574
15.4861	15.30398	29.57025	30.86556	29.42768	1.08E-27	4.35E-61	1.03E-14
0.35395	0.375001	0.622458	0.812192	1.01739	0.022256	0.013605	0.865563
0.549802	0.757252	0.81469	1.238609	1.552784	0.037307	0.000199	0.119993
14.9273	14.42124	29.11241	29.71882	27.25076	1.09E-29	7.86E-58	5.38E-07
0.411265	0.377628	0.841562	0.607022	1.042387	0.03817	0.010543	0.622519
18.50242	17.07004	40.58413	31.82672	31.50651	5.89E-14	1.31E-53	4.31E-11
0.543122	0.629313	1.500087	1.350134	1.123748	7.97E-05	4.31E-47	1.05E-24
1.299176	3.11994	2.961697	3.167484	5.21071	0.02954	0.010047	0.585023
24.582	26.43748	53.42106	43.74766	48.1415	1.35E-19	4.21E-21	0.42097
0.905188	1.329847	2.216824	2.596368	2.069391	2.99E-06	3.28E-20	6.91E-06
2.132269	3.007749	6.018231	5.329367	5.773677	1.41E-06	6.55E-29	1.31E-11
0.478744	0.638112	0.980734	1.367654	1.342032	0.000615	1.27E-10	0.004389
0.184569	0.084736	0.26915	0.21029	0.409887	0.040955	1.11E-05	0.023042
7.114972	7.49609	15.04926	14.4279	12.75999	1.82E-13	1.26E-39	5.67E-08
47.38543	55.51257	106.7063	95.77137	97.08793	2.1E-13	3.98E-32	1.41E-05
371.7238	300.9184	675.1916	730.4486	761.1179	1.28E-18	1.36E-35	0.000172
6.070172	5.706586	12.49218	11.30978	12.21982	1.29E-23	1.28E-87	5.96E-18
0.377439	0.259927	0.699106	0.736686	0.625241	0.002617	2.29E-05	0.177765
109.3948	95.16337	191.3813	208.6993	212.4627	2.91E-30	3.19E-17	0.014002
2.986781	2.550522	5.361512	6.932879	6.904084	5.33E-06	4.43E-33	5.36E-15
0.48134	0.511757	0.986744	0.80295	0.693474	0.003329	0.009463	0.848347
3.75128	3.139263	6.493412	6.175007	5.818252	5.99E-08	5.47E-15	0.00156
2.010362	2.14644	4.671281	3.878884	3.412764	3.42E-06	4.55E-12	0.039092
0.424756	0.491132	1.132252	0.703928	0.929622	0.004004	9.45E-22	2.68E-10
6.999406	6.401432	14.86366	13.02385	12.14087	1.18E-11	1.39E-31	4.21E-07
2.959806	2.691084	5.4669	5.315407	6.505848	5.12E-10	2.1E-37	2.96E-09
35.74332	33.94519	71.59871	65.49772	67.26933	1.12E-10	1.64E-16	0.01223
0.390412	0.459031	0.874818	0.865476	0.852311	7.87E-07	6.24E-27	2.92E-09
1.00303	0.961037	1.624751	1.585483	2.065398	0.000769	1.01E-05	0.33485
0.374165	0.271987	0.778847	0.770263	0.663849	0.010249	0.000287	0.231685
1.935372	1.312306	3.168249	2.969928	3.234315	3.77E-08	6.95E-08	0.889552
2.886991	2.90576	4.724504	5.1561	5.011575	1.08E-10	1.51E-22	3.07E-05
0.232354	0.197546	0.393484	0.336935	0.45618	0.009045	7.96E-28	2.36E-17
0.468575	0.255825	0.471822	0.684726	1.045496	0.015118	2.65E-74	1.88E-45
0.055118	0.061455	0.100008	0.113773	0.123166	0.035406	0.011056	0.641088
7.967157	7.41575	13.24013	14.74304	13.75212	1.81E-18	1.53E-86	3.46E-26
6.468556	8.149552	15.53985	12.13014	12.12478	5.37E-08	0.000239	0.060682
12.05007	10.28042	24.36992	20.66777	21.05303	2.56E-12	1.65E-45	6.62E-11
0.884762	0.634687	1.568554	1.134071	1.417587	0.008779	0.028801	0.657177
8.955994	9.292987	15.14007	18.0035	16.57774	3.94E-16	2.43E-11	0.06755
0.645844	0.571058	1.276006	0.86964	1.080907	0.013237	0.002402	0.581263
45.43895	36.50964	76.1572	80.16102	75.79252	7.83E-20	1.68E-79	2.53E-24
14.55703	15.4113	25.9489	27.52506	27.32987	3.58E-23	2.61E-96	3.98E-30
5.446344	4.831719	8.436949	10.18856	10.25352	2.65E-15	2.92E-43	5.57E-06
30.82385	26.40754	53.28681	56.78408	53.27306	2.91E-20	1.02E-16	0.156834
0.888163	0.706785	1.341137	1.628299	1.440723	0.000356	0.000806	0.823396
9.697539	10.19785	18.21794	19.80801	16.98323	1.12E-17	4.74E-14	0.324283
6.799816	5.915056	13.78805	12.1772	11.81812	0.002285	1.57E-08	0.008584
5.701428	4.49091	10.11667	9.848185	10.69844	2.36E-10	2.09E-43	1.92E-17

0.67361	0.504226	1.264733	0.90096	1.056209	8.95E-05	2.43E-29	2.2E-11
1.321062	1.038644	1.957534	2.16282	2.417988	3.3E-07	3.35E-34	5.95E-11
3.595517	3.725918	6.262877	6.272571	6.684228	1.77E-11	6.13E-31	5.47E-08
7.542893	6.746078	14.76438	11.8029	12.06678	3.28E-10	2.22E-17	0.0427
8.75229	9.199689	15.67635	15.72981	15.62722	5.94E-21	1.34E-10	0.049837
1.491929	1.059206	2.787008	2.236721	2.606346	5.79E-05	1.06E-33	3.95E-17
2.806779	2.22285	6.357408	3.728499	4.3294	0.000546	3.38E-18	7.63E-05
0.772588	0.693092	1.428666	1.415585	1.250159	1.54E-05	4.26E-14	0.001277
20.74507	21.44259	33.77924	39.9301	39.85818	9.01E-20	5.43E-23	0.784718
8.543875	6.432971	19.09884	13.77882	12.25067	0.000195	0.000111	0.937459
2.024863	1.316971	3.786241	3.224624	4.105759	0.023419	1.83E-12	3.83E-07
4.872	3.769651	6.822609	7.860631	8.600793	9.55E-09	6.77E-66	2.73E-24
9.459167	8.256882	17.05902	16.00959	15.13769	8.31E-13	2.13E-42	2.16E-12
1.937011	2.021515	3.560298	4.10464	3.465068	1.86E-09	4.67E-12	0.354285
0.792855	0.594293	1.20567	1.77988	1.195192	0.002985	0.013133	0.691657
1.819302	1.882434	3.759204	3.126137	2.8552	1.5E-06	6.37E-09	0.564763
1.255218	1.4963	2.312139	2.545522	2.653612	9.74E-09	2.21E-20	0.000324
1.054035	0.767587	2.000407	1.185867	2.084596	0.019846	0.043331	0.759769
0.461123	0.277406	0.552015	0.592911	0.870523	0.026729	1.04E-05	0.034937
6.33374	5.950963	10.7468	10.75596	10.85874	6.11E-11	1.8E-10	0.116623
0.156932	0.113219	0.284742	0.261236	0.19482	0.005514	6.51E-12	5.29E-05
36.33478	36.01968	64.20724	63.44067	63.69763	1.17E-28	2.27E-61	3E-09
18.68259	15.77496	31.00877	32.00241	33.78931	1.85E-17	2.94E-54	2.56E-13
3.717458	3.112615	6.645157	6.731696	5.334626	9.27E-09	8.07E-11	0.793061
8.665931	6.763585	15.0424	12.35997	13.78121	3.25E-05	1.47E-49	1.6E-26
3.617567	4.419772	8.17677	7.079064	6.625101	1.1E-06	0.00014	0.246255
2.113512	1.713675	2.700079	4.113276	4.135143	1.33E-05	1.27E-38	4.59E-11
4.455628	4.34691	7.879526	8.069143	6.412921	2.24E-07	7.46E-25	8.71E-07
0.607607	0.717315	1.175961	1.340898	0.969818	0.001068	2.24E-46	7E-28
14.54249	14.54366	24.23981	24.3639	22.53534	3.16E-10	3.83E-51	2.17E-24
1.527448	1.625143	2.504574	2.395757	3.012889	0.000475	0.002788	0.591719
1.303319	1.259709	2.265218	2.536494	3.059975	0.000681	1.4E-23	1.23E-10
7.485589	6.030396	12.79623	13.10039	13.93051	1.53E-05	6.69E-88	6.57E-60
4.266033	5.139807	8.602475	7.999601	7.521533	8.57E-08	1.84E-16	0.005457
33.18781	34.27795	59.9059	60.61335	63.92872	2.28E-26	2.78E-21	0.190544
0.32661	0.265294	0.489285	0.43328	0.502152	0.029672	0.000496	0.163834
12.89978	12.74134	23.94873	22.86869	22.31568	2.89E-19	6.4E-127	6.5E-43
1.346625	1.117274	2.029926	2.355852	2.749756	1.65E-08	6.43E-19	0.0068
0.606248	0.506058	1.228885	0.90766	1.005768	0.009205	2.5E-16	4.21E-08
10.2206	9.147022	18.12887	18.08465	17.13578	1.7E-20	3.1E-58	7.97E-12
4.037471	4.054238	7.662542	7.258654	6.299877	7.71E-12	1.72E-47	1.16E-10
13.27999	13.2319	23.07092	24.34578	22.81955	1.69E-20	1.32E-68	1.86E-15
1751.205	1849.469	3842.448	3141.945	3033.114	1.79E-15	8.36E-44	1.18E-12
1.864195	1.546075	3.195152	3.419562	3.370626	5.74E-06	0.000694	0.281425
5.851209	4.370172	9.793304	8.841729	9.976717	7.02E-09	1.2E-06	0.347553
1.647967	1.050474	2.237241	2.069482	2.544618	0.000148	0.002159	0.38311
0.199939	0.394711	0.643323	0.410045	0.599429	0.020604	0.00616	0.654681
0.427269	0.235394	0.461275	0.398302	0.631206	0.037986	8.11E-07	0.002236
1.500622	0.918593	1.925198	2.6706	2.094375	0.019919	0.001617	0.339839
8.749822	9.058683	16.95569	16.25849	15.00311	3.1E-11	6.26E-10	0.931232
3.09354	3.563568	5.95321	4.771395	4.985565	0.00011	4.45E-30	7.07E-16
1.628365	1.242984	2.790815	2.139787	2.472082	0.000363	0.001859	0.609726
6.131975	6.425713	12.11505	10.33447	11.50036	4.65E-08	1.65E-24	5.35E-06
12.94086	12.70224	22.71343	23.79973	22.09899	9.55E-22	5.47E-36	0.006408
0.478457	0.282423	0.651097	0.573454	0.653369	0.039841	0.003509	0.347436
0.161678	0.296909	0.55672	0.462198	0.430862	0.033032	0.017894	0.814978

6.388962	5.619413	11.58829	9.027763	10.46118	4.51E-07	2.07E-21	8.94E-05
9.532323	8.862261	14.31805	15.77637	17.42121	9.62E-16	4.73E-54	4.79E-08
0.527951	0.403975	0.938773	0.705426	0.6576	0.026947	0.000927	0.288074
1.80345	2.145207	3.054093	3.362367	3.633062	0.002397	0.014355	0.575361
28.79511	30.37028	48.45777	51.72961	48.35531	1.61E-18	8.08E-27	0.021542
11.51161	12.23971	22.53917	20.20059	19.2825	1.44E-15	1.26E-16	0.973896
2.67518	2.340201	4.009966	4.381023	4.319616	1.75E-06	5.71E-20	6.76E-06
30.70846	31.6247	59.09943	55.42709	53.42491	5.29E-22	6.48E-24	0.757637
8.228534	5.798435	11.34227	8.740997	12.6383	0.017462	1.67E-11	1.04E-05
5.161249	3.489377	6.298587	8.083798	9.327449	1.06E-06	1.12E-47	2.79E-18
1.487222	1.575675	2.567004	2.106253	2.609654	0.003128	2.67E-10	0.000336
0.857493	0.877344	2.053742	2.009816	1.575489	0.009659	7.15E-07	0.012281
0.815908	1.101062	1.695744	1.843861	1.546966	0.000293	0.001004	0.859582
17.86269	17.6555	31.65253	29.79772	28.84737	3.14E-16	2.99E-31	2.64E-05
1.378671	1.327165	2.391226	2.466759	2.270533	3.99E-06	2.19E-10	0.063127
8.078255	7.73069	15.35875	13.41198	12.78052	2.03E-10	7.4E-144	4.73E-59
2.245683	3.324995	4.920134	4.343877	3.634677	0.001117	5.75E-20	2.45E-10
3.966825	3.595082	7.132112	6.819485	5.349847	8.55E-06	5.22E-09	0.364199
104.992	112.6461	204.5172	176.7757	162.9256	1.83E-14	8.68E-21	0.938757
10.95065	11.85089	16.94892	19.12502	17.2707	1.76E-09	1.53E-25	8.95E-08
2.289311	2.716376	4.505329	4.316933	4.096928	1.84E-08	8.45E-11	0.22474
28.28691	27.17445	51.03202	45.66591	46.88937	8.56E-16	1.41E-89	3.78E-33
6.453829	7.139985	11.72631	11.9699	10.34048	3.62E-09	9.71E-06	0.161654
19.1521	18.58487	31.49539	32.01856	28.95371	3.15E-14	3.97E-28	0.006058
5.208416	4.402871	8.575299	7.93804	8.010527	2.71E-08	1.18E-06	0.641685
103.1842	104.7953	175.508	166.0982	168.1042	1.03E-19	1.01E-61	3.23E-16
1.314805	1.278286	2.42305	1.97068	2.329946	0.002736	6.26E-12	0.00016
1.300922	1.349367	1.876694	2.335618	2.030721	0.003754	2.32E-10	0.000518
14.65377	12.63806	20.4382	23.83287	24.07423	1.68E-14	9.96E-27	0.182745
35.316	39.57189	63.87004	62.62943	54.82038	3.2E-11	1.11E-14	0.44901
16.53457	16.11732	28.17737	26.78317	25.17639	5.57E-17	4.25E-13	0.469969
14.52136	15.95829	27.95305	24.82033	24.28755	9.52E-14	1.85E-62	3.2E-15
6.286424	6.817639	10.09054	11.37438	11.04413	6.22E-12	1.34E-31	1.84E-05
2.926872	2.794988	5.041546	4.911195	4.447421	7.48E-07	1.63E-26	3.34E-08
10.14426	9.796368	18.80807	15.24456	18.66937	1.26E-08	4.4E-17	0.033291
2.930459	2.934546	4.115368	5.263683	5.119772	9.08E-08	1.81E-08	0.807556
0.18455	0.242887	0.322948	0.42436	0.352823	0.015363	1.06E-29	1.72E-19
154.5389	159.9454	265.0063	251.9414	245.5534	1.74E-18	1.48E-20	0.595602
4.793509	6.199234	8.575007	8.118874	8.653829	4.34E-05	1.86E-05	0.568303
0.495196	0.53589	0.748751	1.137573	0.660859	0.036572	1.99E-12	7.1E-06
11.88247	10.96657	18.22646	19.44135	19.69863	1.74E-14	4.97E-45	9.02E-09
2.383982	2.666601	4.184015	4.182073	4.105707	3.03E-05	0.000176	0.697212
6.50819	6.398969	11.50911	11.01985	9.697161	1.85E-10	1.31E-07	0.228355
4.606243	3.781676	6.014067	7.372137	8.451481	1.85E-08	5.03E-33	0.000313
9.244593	8.258534	13.11076	15.62175	16.36049	1.06E-10	5.08E-17	0.038357
11.12518	9.695547	18.06344	17.64879	20.04799	1.65E-09	2.4E-204	5.2E-138
2.541757	2.959444	4.504083	3.590794	4.167105	0.000902	4.92E-11	0.000679
2.297504	1.871415	2.541544	3.627144	2.898199	0.012476	0.023367	0.773119
25.16719	26.13406	46.95695	41.2696	35.27166	1.17E-09	2.15E-10	0.665894
1.109864	1.232034	1.837445	1.667745	2.274433	0.001564	1.24E-05	0.313143
3.240035	3.501719	5.513162	5.332621	5.685085	1.43E-07	8.99E-05	0.538448
1.186003	1.089002	1.73041	1.851578	2.149937	1.26E-08	3.18E-51	5.23E-18
0.857244	0.854841	1.23318	1.546695	1.7302	0.000427	2.46E-06	0.321435
15.04222	12.48503	24.973	23.45233	20.6636	8.01E-09	2.74E-78	1.07E-37
11.59334	12.037	19.46662	19.12876	17.22203	3.89E-09	7.54E-36	1.27E-11
3.951457	4.42716	7.039568	7.175628	6.437113	2.96E-09	4.65E-07	0.593466

4.52673	3.896716	7.003581	6.958075	6.941895	3.27E-11	4.59E-48	6.59E-17
2.142645	2.006438	3.135349	3.408231	3.627875	2.22E-10	2.09E-36	2.74E-09
447.8391	539.3988	886.1562	736.2526	747.5738	1.08E-11	1.43E-22	0.056818
9.591097	9.551632	16.9047	15.59923	14.27725	2.72E-11	8.29E-23	0.000749
0.621839	0.57098	0.753488	1.239318	1.295049	0.011249	2.11E-19	3.53E-09
1.177077	0.926405	1.97555	1.645914	1.442996	0.009963	1.25E-05	0.096173
1.471535	1.567632	2.473857	2.570405	2.489251	3.8E-07	2.35E-14	0.001509
0.464289	0.395311	0.586122	0.794801	0.806937	0.008208	8.41E-11	0.000117
1.851251	1.821257	3.750853	3.174135	2.815298	0.005209	0.003163	0.653094
14.86634	15.24562	26.38147	22.64611	21.50674	1.07E-08	1.88E-21	0.002642
8.740104	11.95844	17.2924	13.71371	13.08475	0.000704	0.003082	0.471332
0.751007	1.014858	1.631755	1.439811	1.539214	0.003379	1.83E-13	2.49E-06
2.595068	2.711941	3.921216	4.236824	4.485324	8.75E-09	1.54E-08	0.850349
22.86238	21.63977	34.13532	35.53843	34.75151	5.89E-14	2.63E-16	0.462159
3.881923	4.015921	6.990277	6.561989	6.386973	1.32E-05	6.96E-15	0.000143
4.982507	5.22924	7.70855	8.133026	8.338897	1.59E-13	2.51E-15	0.416107
6.612652	5.854366	11.30631	9.221037	9.371559	2.51E-07	8.17E-06	0.601041
33.59912	28.66547	47.68988	53.10954	49.32585	2.55E-14	6.57E-55	1.4E-13
17.81423	14.32594	28.19614	25.5067	33.59191	0.000415	2.18E-22	6.65E-10
1.195836	1.422449	2.335787	2.026842	1.759529	0.001107	3.13E-13	0.00021
65.98545	64.24061	108.6155	102.2084	106.9813	2.37E-17	2.64E-14	0.310544
8.138489	9.240824	13.44615	14.72936	12.26452	6.15E-08	3.93E-11	0.237269
150.9316	158.8418	263.242	228.5212	241.2682	2.67E-15	7.55E-15	0.671942
2.519391	2.359509	3.402145	3.858057	4.269466	2.59E-08	2.97E-24	8.71E-06
6.314428	5.133816	8.773147	9.020853	9.309645	3.7E-07	1.45E-09	0.218529
1.228588	1.19861	1.787998	2.075846	1.885062	0.004103	3.25E-09	0.00197
2.943217	2.551029	4.300574	4.329993	4.805992	1.43E-07	3.8E-105	1.7E-54
0.53442	0.562087	0.740475	0.914855	1.232804	0.013194	0.023144	0.858238
4.336032	4.947179	8.342608	6.803477	6.230296	4.37E-05	1.17E-06	0.659376
2.02717	1.748561	3.615006	2.777338	2.909335	0.006335	6.26E-05	0.172681
0.629587	0.656215	1.022963	1.110357	1.212519	0.02205	3.93E-06	0.015829
52.46435	55.23041	87.61088	80.55485	80.89402	2.55E-15	6.02E-32	2.72E-05
0.584606	0.747299	1.36855	0.854859	0.906475	0.016669	4.85E-07	0.022674
0.342122	0.366497	0.413839	0.615075	0.587531	0.026831	0.0002	0.173756
0.849174	0.8397	1.308997	1.278744	1.116363	0.032664	0.029863	0.967735
6.875442	6.936773	11.014	10.42932	10.25679	2.85E-12	4.3E-18	0.173932
3.188911	2.983343	4.901056	4.93583	4.350218	1.86E-06	2.15E-17	0.000396
1.598804	1.265184	2.10695	2.390077	2.293715	4.81E-06	1.29E-40	5.45E-19
2.122766	2.077195	3.870362	3.221049	3.056528	0.000188	5.71E-17	9.09E-06
8.478232	7.82281	12.00997	13.01488	12.99108	9.4E-14	3.9E-42	9.79E-08
36.87873	33.93964	49.69208	56.97978	54.63812	7.62E-13	9.29E-61	1.21E-13
4.023323	5.073107	7.677055	6.708744	6.672475	0.000131	5.95E-21	6.07E-08
1.215462	1.144357	1.804785	1.821377	1.905823	6.8E-12	2.24E-41	1.99E-14
0.964904	1.014148	1.366837	1.544403	1.597904	7.49E-05	1.17E-06	0.391625
2.517536	2.328628	4.106636	3.882668	3.410309	0.000238	1.47E-06	0.261773
2.650006	2.76252	4.147059	4.043866	4.45372	2.43E-07	2.76E-08	0.514034
3.072535	2.861949	4.46744	4.988636	4.604184	3.1E-10	3.99E-19	0.006988
3.159454	2.358271	4.729105	4.73141	3.896943	0.000511	0.00014	0.719806
0.72532	0.942142	1.228309	1.269835	1.322103	0.020923	2.68E-08	0.000896
17.95067	16.31398	23.68508	26.38378	27.57769	1.26E-09	1.12E-39	1.43E-08
1.237088	1.271136	2.169795	2.04559	2.470017	0.003457	3.06E-32	1.96E-20
1.080561	1.281571	2.109469	1.902684	1.747603	0.003598	1.61E-05	0.081649
2.803991	3.348738	5.369198	4.920267	4.60261	0.000111	4.8E-12	0.003214
12.30298	10.63069	17.89621	19.29159	18.32975	3.63E-09	7.05E-16	0.023569
3.579191	3.138035	4.770798	4.541994	4.254124	0.002863	0.001529	0.789657
24.26954	24.14352	35.05817	38.50481	36.19464	2.98E-11	3.16E-09	0.782727

11.36788	12.35103	17.30663	17.53964	17.13452	1.47E-08	4.6E-09	0.803176
4.212752	4.626288	6.847377	6.532084	5.831673	2.98E-06	8.8E-14	0.002797
4.536566	4.810632	7.954497	6.474494	6.628626	3.2E-05	3.3E-07	0.379377
3.451615	3.349987	5.86604	4.814949	5.001479	5.17E-07	9.43E-32	1.62E-08
21.62765	20.37647	31.80277	32.89701	30.62898	1.11E-12	3.68E-70	1.6E-28
3.397924	2.893103	4.231437	4.824926	5.708755	5.92E-06	4.97E-23	5.71E-05
1.542919	1.614922	2.450431	2.496558	1.959098	0.000196	9.38E-17	9.27E-06
6.827219	6.988206	10.4008	9.807374	9.555813	2.93E-05	0.000104	0.989781
5.17245	4.0843	6.081477	7.123298	7.526917	5.45E-06	3.62E-62	2.1E-31
15.26379	14.03871	20.68765	23.43873	20.8013	1.73E-09	3.02E-97	1.59E-38
21.95099	21.05125	32.0938	32.99664	31.81676	4.86E-14	1.23E-18	0.019498
1.117716	1.35285	1.849803	1.720779	1.707131	0.010083	0.002401	0.598004
3.533286	3.443189	5.009193	5.312896	4.787996	1.99E-05	1.36E-14	0.000233
72.94595	75.16896	111.1014	110.7578	108.8507	5.33E-15	9.42E-18	0.097063
135.2068	139.8387	200.791	198.18	202.563	9.9E-13	2.28E-66	1.66E-27
2.490173	2.451299	3.780138	3.743437	3.041251	0.0029	1.57E-05	0.193821
2.994496	2.555593	3.989988	4.448388	4.585823	1.14E-07	1.21E-15	0.028289
4.777667	4.136878	6.81224	6.819098	6.216952	1.57E-06	7.71E-23	5.54E-07
164.8256	169.7116	243.7079	256.0911	267.3321	7.32E-16	9.92E-10	0.882122
54.03705	52.10581	78.64527	77.22697	81.62923	6.53E-15	3E-112	4.66E-50
5.512107	4.228577	7.642853	8.044448	9.555443	0.000325	2.71E-86	1.84E-75
20.17002	20.72239	30.39535	30.3414	31.60415	1.43E-14	1.23E-14	0.17056
0.50908	0.387765	0.548617	0.668707	0.618343	0.016238	0.017026	0.972147
15.34928	15.75994	24.85168	22.74299	25.2069	1.01E-08	2.15E-33	1.21E-08
0.557367	0.389565	0.598734	0.752228	0.585562	0.037545	0.018506	0.810141
17.7395	14.46847	23.46646	24.20498	25.45506	4.86E-10	3.3E-249	2.5E-178

Supplemental table S4

gene_name	WTvsCon _log2FC	KOvsCon _log2FC	KOvsWT_ log2FC	N_CON_1 _fpkm	N_CON_2 _fpkm	N_CON_3 _fpkm	N_WT_1_ fpkm
Clic5	-1.03103	-8.47767	-7.45092	12.21206	9.255682	13.26187	5.660099
Tgtp1	-0.72764	-7.94396	-7.22817	165.0382	120.2441	148.543	85.9461
F830016B08Rik	-1.8184	-8.89021	-7.08827	1.616544	1.060376	1.600147	0.284461
BC023105	-2.09802	-8.52957	-6.44118	6.708634	4.08396	6.639983	1.528717
Gm5970	-0.97649	-7.1925	-6.22486	2.435008	1.677112	2.855759	1.315701
Gbp4	-1.03419	-6.55787	-5.53482	98.84912	71.78881	82.36657	40.39373
Gm4951	-1.28799	-6.49325	-5.21631	6.157025	4.207608	4.292151	2.148346
Iigp1	-1.10186	-6.28829	-5.19818	39.39104	31.9436	38.96439	18.56044
Tgtp2	-0.7597	-5.75262	-5.00482	149.4594	122.1876	152.7912	85.58845
Ifng	-0.83099	-5.73372	-4.91318	12.94138	14.57817	21.2461	8.562862
Gm18853	-1.23845	-5.88394	-4.66376	1.202173	1.247667	1.07711	0.552301
Gbp10	-1.60127	-6.14823	-4.55612	3.165095	2.632802	3.086436	0.854666
2210412B16Rik	-1.9679	-6.10899	-4.1623	1.545888	0.70202	1.877372	0.11078
Gbp3	-0.99137	-5.11642	-4.13673	178.3111	166.7187	177.3527	93.15313
Gm12188	-0.97407	-5.08974	-4.12504	19.48509	15.78866	17.6646	8.364285
Gm12185	-1.79673	-5.8933	-4.10784	14.30932	10.29357	13.72192	3.910292
C4b	-2.23859	-6.30264	-4.0754	8.547556	6.803863	7.035731	1.298087
Gbp2b	-1.53986	-5.53817	-4.01616	0.293685	0.350886	0.307528	0.07817
Gm38048	-2.55741	-6.4565	-3.9107	0.680996	0.616447	1.04441	0.152259
Gm12250	-0.7677	-4.59895	-3.84269	315.5733	296.8724	319.6463	190.2757
Gbp8	-1.86749	-5.64531	-3.7899	31.16629	22.54212	24.34212	6.922124
Gbp9	-0.70197	-4.46483	-3.77453	121.8731	97.29675	114.9203	72.7655
Fgf16	-2.18598	-5.86799	-3.68678	0.575994	0.708421	0.771329	0.167686
Gbp7	-1.19569	-4.70812	-3.5241	266.1497	240.1803	280.8149	126.6993
Irgm1	-0.67489	-4.08767	-3.42435	255.2549	222.4	259.4222	162.3251
Igtp	-0.6161	-3.93253	-3.32841	766.2203	690.394	728.2147	487.1324
Gm18852	-1.28997	-4.51497	-3.23898	0.477974	0.421642	0.464659	0.153545
Olfr1396	-0.79271	-3.9777	-3.19612	1.296775	1.00154	1.289387	0.675482
Slamf8	-1.42221	-4.59994	-3.19112	15.77132	13.94715	16.37529	5.016964
9530082P21Rik	-0.71188	-3.74672	-3.04727	5.454739	5.042163	5.26854	3.304196
Pla2g16	-1.11632	-4.14916	-3.04532	10.90688	8.545196	10.19044	3.881474
Trim30c	-1.76789	-4.78742	-3.03078	32.40501	23.73491	27.70859	9.162632
Cyp2b9	-1.45183	-4.46434	-3.02774	0.378802	0.279536	0.605157	0.176446
AW112010	-1.66048	-4.64783	-3.0002	201.269	180.9325	195.3989	58.46448
Gm12216	-0.81751	-3.7399	-2.9358	6.957774	7.462312	6.45859	3.584818
Gbp11	-1.46335	-4.37493	-2.92987	1.517882	0.952369	1.240357	0.57382
Gm4070	-1.43129	-4.24421	-2.82373	11.19262	9.354238	10.87213	4.55625
Olfr164	-1.9449	-4.64083	-2.71567	0.4519	0.222319	0.718073	0.133951
Clec9a	-1.9277	-4.57192	-2.65721	1.276822	1.592496	1.804887	0.35182
Gm2389	-1.18513	-3.82226	-2.6471	6.266602	9.248836	8.833441	3.210876
9930111J21Rik1	-1.69699	-4.31642	-2.63158	20.79635	17.35531	20.83234	6.649546
Gm4759	-0.76573	-3.37552	-2.62267	1.672308	1.073491	1.966515	0.783125
9130208D14Rik	-1.70735	-4.26135	-2.56127	0.576522	0.814932	0.604351	0.136163
4933412E12Rik	-0.66937	-3.21397	-2.55733	9.245426	9.192598	11.44108	5.281144
Gm22079	-1.57886	-4.13539	-2.55683	18.9949	19.23932	24.5703	9.888727
Wars	-1.23908	-3.72856	-2.50177	146.8716	136.2114	149.7192	54.04835
Cfb	-0.77456	-3.25969	-2.49648	1.949523	1.072538	1.53655	0.917851
Casp12	-0.85384	-3.32543	-2.484	0.755664	0.713172	0.643461	0.387905
Gvin1	-1.51488	-3.98311	-2.48019	12.32395	9.680003	12.12511	4.07409
Slc6a19	-0.84723	-3.29053	-2.4568	0.402209	0.419024	0.333504	0.231431
Apol9b	-1.97183	-4.38992	-2.42902	0.813006	0.639952	1.026833	0.242367
Zbp1	-0.76332	-3.17976	-2.42847	321.6037	309.8968	341.1518	198.7886
Gm1966	-1.32458	-3.72589	-2.41303	222.164	187.9777	234.3052	94.41388

Gm8989	-1.3837	-3.74029	-2.37022	0.748891	0.632735	0.495693	0.20475
Heatr9	-1.34086	-3.66853	-2.34617	2.274024	1.615957	1.531507	0.853276
9930111J21Rik2	-1.43025	-3.72824	-2.30952	32.81245	31.52117	36.03138	14.89091
Gm6545	-1.03121	-3.3114	-2.29034	3.348913	1.663858	3.365215	1.482697
Gm18301	-1.47828	-3.73852	-2.26827	2.20072	2.034119	2.256109	0.80765
Ccl5	-0.76333	-3.01504	-2.26374	299.5514	227.7902	229.2112	139.6457
Tmem140	-0.62592	-2.86896	-2.25511	18.22213	17.103	18.22125	13.48819
Irgm2	-0.88538	-3.10594	-2.23267	207.0592	200.5734	202.3513	115.3609
Oasl2	-0.71248	-2.90167	-2.20136	471.6739	490.8324	480.7402	301.714
Gm5431	-2.98355	-5.14657	-2.18055	12.38976	11.11944	13.40112	1.671346
H2-Q5	-0.68507	-2.83348	-2.16073	153.5014	136.8246	137.2973	84.74687
Adgrb1	-3.31182	-5.45223	-2.15373	3.575139	3.638704	3.217907	0.313198
9330175E14Rik	-0.8877	-2.98938	-2.11436	21.00304	22.84391	19.91728	11.71568
Gm8995	-0.94756	-3.03426	-2.09835	339.8761	290.7582	345.1795	191.8249
Ifi213	-1.24218	-3.25421	-2.02341	10.61447	9.430162	11.47185	5.016802
Gm45418	-1.12663	-3.09602	-1.98171	12.13587	12.65908	16.87528	6.715449
Mertk	-1.98773	-3.92776	-1.95739	1.2565	1.303497	1.401535	0.216298
Gm7582	-0.64863	-2.59413	-1.9569	10.99929	10.72589	15.28317	7.776681
Cysltr2	-0.99486	-2.9404	-1.95618	1.670185	1.521614	1.999704	0.941249
Il12rb1	-0.84672	-2.78292	-1.9478	5.150139	4.897039	7.174261	2.835711
Trim5	-0.60455	-2.5233	-1.93091	19.37068	19.61204	22.35076	15.24988
Nlrp1b	-1.54545	-3.44486	-1.91186	1.155271	1.127463	1.345149	0.37625
Ifit1bl2	-2.72887	-4.61407	-1.89807	29.10843	31.53065	35.58206	4.751684
Samhd1	-0.65283	-2.53025	-1.88973	838.2747	813.1428	844.3723	532.616
Gm9574	-1.06313	-2.92133	-1.87059	5.746733	4.068393	5.136986	2.285094
Gm5960	-2.50365	-4.33473	-1.84977	50.46775	44.77712	48.52301	8.153008
H2-Q7	-0.62225	-2.45106	-1.84133	112.9681	100.6193	88.40434	58.23433
Ifi206	-1.23377	-3.06068	-1.83853	19.21624	19.70874	20.59841	9.267039
H2-Q6	-0.66324	-2.48544	-1.83458	243.3533	210.7976	205.4991	130.1558
Phf11d	-1.04388	-2.84617	-1.8165	2.080183	1.774716	2.598186	0.748175
Mir1932	-1.33135	-3.11037	-1.79587	15.13384	22.01223	14.09817	5.516869
Fbxw17	-0.78248	-2.56301	-1.79298	13.80441	12.22431	11.78897	6.935822
Obscn	-1.77997	-3.54156	-1.7704	0.21927	0.177436	0.166386	0.070636
4930599N23Rik	-0.94117	-2.69406	-1.76698	5.652759	5.561914	5.767434	3.197276
Ifi208	-1.18251	-2.93559	-1.76432	12.85076	13.28698	14.94884	7.166264
Phf11b	-0.87451	-2.62595	-1.76268	40.06311	34.1043	39.19989	24.88182
Gm43197	-0.60327	-2.33711	-1.74572	15.91105	13.12884	13.72846	11.19179
Stx2	-0.69219	-2.42499	-1.7451	46.90031	45.04119	46.10294	27.77682
Ifi44	-0.82405	-2.54879	-1.73647	1.159459	0.770057	0.937045	0.729102
Fbn1	-1.14321	-2.86044	-1.72912	0.13672	0.134523	0.214747	0.036391
Il15	-1.7101	-3.42318	-1.72581	44.91873	46.92854	38.45583	14.03007
Gm48277	-1.7951	-3.50626	-1.72249	7.206952	8.730115	9.6606	2.723598
AC154218.2	-1.58294	-3.27797	-1.70854	3.450041	4.849422	5.044902	1.342132
Gm43714	-1.9006	-3.58461	-1.69772	2.557648	1.790618	2.606892	0.733139
H2-M3	-0.60625	-2.28996	-1.69619	49.04749	43.19425	43.09638	27.35415
Ctrl	-0.94525	-2.61853	-1.68317	2.202797	2.016182	2.368524	1.193094
Gm371	-2.23542	-3.90853	-1.67845	3.805262	4.446127	4.130219	0.79762
Rnf213	-0.918	-2.55968	-1.65332	86.5568	78.53093	93.34209	46.08062
Gm45014	-1.70486	-3.32478	-1.63391	19.30113	14.09934	16.98376	4.359007
Trim12c	-0.65881	-2.27701	-1.6303	117.4412	116.5977	125.2765	85.76223
Scnn1a	-3.12367	-4.69634	-1.58442	56.52499	64.68388	56.77662	6.515566
Gm46224	-0.6517	-2.19407	-1.55474	33.0427	37.39077	38.3909	27.53992
Kcnab1	-1.82182	-3.36388	-1.55128	0.427278	0.535686	0.520693	0.186185
Gm16675	-1.44458	-2.97648	-1.54453	9.496166	9.176405	9.597587	3.671582
Nrp1	-2.32383	-3.85083	-1.54318	2.081908	2.417647	2.074744	0.389028
Sema4f	-4.43913	-5.96193	-1.52997	4.321112	5.606473	4.645622	0.260722

Rasgef1b	-1.16353	-2.67128	-1.51906	1.698723	1.468826	2.022049	0.831227
Tnfsf10	-1.22377	-2.7323	-1.51767	2.901367	2.987518	3.208421	1.372408
Gm11131	-0.62098	-2.11841	-1.51054	7.720144	7.222802	8.385035	4.506091
Gm15753	-1.09722	-2.58011	-1.49726	7.081791	7.845835	5.91092	3.116875
Gpr141b	-2.12956	-3.60013	-1.4802	1.71402	1.939445	2.246967	0.558872
Siglech	-0.91409	-2.37925	-1.47735	6.581877	7.475072	9.001493	4.272597
H2-T23	-0.65646	-2.11826	-1.47436	577.5581	605.1447	552.7159	354.5004
Itgb7	-2.89275	-4.33389	-1.45432	239.1223	267.3161	240.652	29.03825
Ifi207	-0.87066	-2.30615	-1.44724	32.1709	33.42473	33.8978	21.08727
Ifi205	-1.2496	-2.68828	-1.44571	2.509416	2.608584	3.021684	1.215111
Parp11	-0.88058	-2.3123	-1.44414	6.692313	7.513593	7.518307	4.200861
Fgl2	-2.47453	-3.8998	-1.43681	190.29	187.4318	242.3008	39.92011
A930037H05Rik	-0.90471	-2.33227	-1.43466	0.802024	0.891787	1.213261	0.510259
Cpt1a	-1.11974	-2.53451	-1.42735	34.77417	39.2426	38.26696	15.71666
Irf7	-0.61558	-2.01865	-1.41492	342.0641	324.5923	337.848	213.5011
H2-T24	-1.02809	-2.4279	-1.4118	309.7212	327.2816	323.5289	167.0231
Tmem67	-1.26239	-2.65507	-1.40653	2.653555	2.422803	2.552573	1.139849
Phf11c	-1.7751	-3.16978	-1.40579	4.793706	4.885118	5.695386	1.738476
Ly75	-0.824	-2.21675	-1.40508	21.46649	18.96125	20.30021	11.59301
Klf4	-1.22675	-2.60771	-1.39275	5.263514	3.114998	4.621434	1.646556
4931422A03Rik	-1.24288	-2.6209	-1.38803	2.772443	3.018862	6.00277	1.652994
Tesc	-0.76589	-2.13841	-1.38676	4.354914	3.296097	2.728317	1.282993
Tmem229b	-1.2758	-2.64925	-1.38556	29.97262	33.03723	36.63631	13.33748
Sp110	-0.87072	-2.22304	-1.36433	51.85735	52.60502	58.91208	30.37085
Ccr9	-0.60771	-1.94833	-1.35417	0.784974	1.08471	0.924257	0.49469
Cers6	-0.72209	-2.02508	-1.31529	92.52236	92.8803	98.94095	58.50057
Oas3	-1.05163	-2.35454	-1.31504	201.944	204.8889	201.1643	92.30161
Ddx60	-0.98544	-2.28238	-1.30867	53.96165	56.20106	61.46696	32.24738
Ly6c2	-1.44294	-2.73523	-1.30495	579.5719	585.7176	515.7965	216.4868
Tnfrsf14	-0.76304	-2.0536	-1.30324	122.1863	110.2547	119.0259	62.036
Ms4a4c	-0.81222	-2.092	-1.2919	23.3022	22.26218	25.70547	16.19458
Ifi203	-0.69009	-1.96671	-1.28763	9.466624	9.505641	11.14341	6.935163
Trim34a	-0.60406	-1.87616	-1.28401	40.53055	43.88059	43.82491	30.18102
Axl	-1.76997	-3.0401	-1.28167	5.44776	4.897124	5.65242	1.600363
4930568A12Rik	-1.6129	-2.87851	-1.27818	5.38358	2.334298	3.807227	1.275085
Itga4	-1.28219	-2.52516	-1.25466	9.180742	8.641356	12.59844	4.369133
Setdb2	-0.91324	-2.14308	-1.24265	3.57043	3.412265	3.943814	1.826687
Trim12a	-0.67938	-1.90872	-1.24139	63.9008	61.59379	68.37496	44.75716
Crybg1	-1.37201	-2.59657	-1.23711	13.9923	15.5716	15.5393	5.799127
Gm43196	-0.78293	-2.00539	-1.23537	3.977839	4.713231	4.264622	2.71407
Fam174a	-0.84711	-2.06836	-1.23398	16.09035	16.21908	15.48139	8.171017
Kat2b	-1.16434	-2.38128	-1.22879	25.34497	23.95595	29.36325	12.44611
Phf11a	-0.7336	-1.94983	-1.22707	4.679818	3.012361	5.080077	3.463322
Slc46a3	-1.05389	-2.26816	-1.22696	2.385158	2.536469	2.445189	0.875337
Siglec1	-1.36721	-2.57919	-1.22399	2.711009	2.7898	3.459177	1.143072
Rnase6	-1.27999	-2.48253	-1.21493	9.059433	11.57925	11.56833	4.426734
Cyld	-0.58717	-1.76502	-1.18991	17.4748	16.14222	18.82459	11.89268
Gem	-0.68578	-1.85838	-1.18576	4.053038	3.849432	3.491392	1.940344
Rbl1	-0.75925	-1.91787	-1.16984	9.120556	8.351731	9.716815	5.624573
Usp53	-0.96557	-2.1188	-1.1654	1.111659	1.107697	1.088933	0.653845
Cd1d1	-0.72378	-1.87638	-1.16502	9.908302	8.38873	8.678908	4.744078
Gm26797	-1.84626	-2.97974	-1.15225	0.89327	1.037407	1.420166	0.313769
Clec7a	-1.76253	-2.87713	-1.12667	521.7502	472.3259	541.2335	167.932
Kynu	-0.86802	-1.98213	-1.12495	0.683782	0.663317	0.901416	0.376823
Gm38253	-0.98591	-2.09136	-1.11925	1.44314	0.734885	1.184867	0.542488
Frmd4b	-2.00962	-3.10563	-1.10954	0.467892	0.554077	0.494949	0.138867

4930512H18Rik	-0.83988	-1.93613	-1.10754	8.381142	9.584688	10.44616	5.718914
Gm43068	-1.21942	-2.29727	-1.09259	7.117491	6.820018	4.301671	2.513577
BC147527	-0.88349	-1.95938	-1.08923	4.938043	5.370124	6.154721	3.078378
Pacsin1	-0.74239	-1.80727	-1.07711	0.853122	0.589374	0.869942	0.363564
Pdlim4	-0.81939	-1.88373	-1.07685	1.087231	1.251844	1.591262	0.711159
Nectin4	-1.30689	-2.36078	-1.06648	4.426436	3.997541	4.688562	1.62001
Gzmb	-1.82121	-2.86732	-1.05878	63.17834	65.89602	60.96772	17.01931
Rpgrip1	-0.83198	-1.86721	-1.04763	0.487322	0.388387	0.351709	0.272393
Mndal	-0.65039	-1.68237	-1.04245	18.39588	18.10024	21.49945	14.55249
Srgap3	-0.73774	-1.7645	-1.03809	0.360654	0.331201	0.589721	0.201591
Hectd4	-1.00161	-2.02216	-1.03272	7.523523	5.903663	6.299157	2.918978
Gm45884	-0.88265	-1.89536	-1.02346	1.196176	0.869921	1.094767	0.678304
Prr5l	-1.02637	-2.03209	-1.01827	23.47553	23.6848	21.10849	11.21811
Mfsd4a	-0.74293	-1.74748	-1.01738	5.306426	5.561656	5.101248	3.262375
Hbb-bs	-0.78157	-1.785	-1.01644	15.2675	15.70496	16.95607	7.758097
Tbkbp1	-0.72463	-1.72814	-1.01588	54.36265	50.00025	51.17343	27.66068
Samd9l	-0.74085	-1.74339	-1.0147	232.9056	214.754	201.2038	150.2882
Twistnb	-0.78349	-1.77096	-0.99986	6.043732	7.190189	7.502152	4.228101
Zap70	-0.67933	-1.65401	-0.98866	0.851081	1.110046	0.959751	0.590039
Flt3l	-0.95091	-1.91566	-0.97749	6.428922	7.167013	7.628649	3.741456
Fzd5	-0.64549	-1.61033	-0.97746	1.198946	1.415614	1.25546	0.925462
Ptgs2os2	-0.67253	-1.63352	-0.97255	9.264695	5.409377	6.812026	5.058447
Xk	-0.85433	-1.81229	-0.9703	1.808315	1.492277	1.920804	0.902088
Cd300c	-0.97464	-1.927	-0.96433	2.780253	2.68086	2.821725	1.243238
Epsti1	-0.67434	-1.62237	-0.95953	37.99124	41.67356	45.37413	25.74222
Cd180	-1.47641	-2.41894	-0.95489	7.85389	8.141191	9.286221	3.193397
Asb13	-1.25852	-2.19549	-0.95024	2.590505	2.408469	2.104307	0.930556
Dnah8	-0.80931	-1.74115	-0.94399	0.62131	0.563378	0.696063	0.351827
Mgam	-1.72442	-2.64895	-0.93701	35.07306	36.50432	37.87878	10.84486
Plekhg6	-1.20849	-2.12328	-0.92805	5.474966	6.04048	4.149763	2.291031
Lrrk2	-0.97649	-1.88985	-0.92562	49.17101	45.36252	45.50621	26.07389
Gm37893	-0.79473	-1.69541	-0.91301	3.037515	2.91697	2.932108	1.811036
Apba1	-0.80053	-1.70168	-0.91146	0.81257	0.799511	1.339241	0.524069
Pgap1	-1.77574	-2.6546	-0.89292	0.684494	0.648939	0.743751	0.239127
Zbtb10	-0.73571	-1.61413	-0.88928	0.431325	0.353661	0.504414	0.275323
Mycbp2	-1.06074	-1.93368	-0.88512	13.05423	12.60529	14.52677	6.213929
Ly6g5b	-1.5362	-2.40473	-0.88304	9.567758	6.14387	8.688321	1.595013
Tlr12	-0.85383	-1.70874	-0.86806	1.528483	1.522259	1.261205	0.764061
Zbtb37	-1.24479	-2.09928	-0.86604	2.470884	2.254643	2.513066	1.123079
Atxn7	-0.6768	-1.51844	-0.85393	9.89464	8.749645	9.542508	5.926243
Fam126a	-1.16799	-2.00102	-0.84489	7.398765	6.773033	8.541502	3.380902
Klrk1	-0.91598	-1.74643	-0.84374	0.964593	1.159999	1.23651	0.499229
Gls	-0.79006	-1.61934	-0.84143	10.7096	10.39817	11.68675	7.064171
Il18	-0.87845	-1.70438	-0.83604	19.12711	15.20272	20.40345	12.09323
Gm26789	-1.35423	-2.16524	-0.82557	3.076946	3.72615	3.202917	1.521439
Etnk1	-0.75903	-1.56515	-0.81814	17.81478	16.58967	18.26314	10.42088
Ptgfrn	-1.82784	-2.6326	-0.8181	1.097913	1.759612	1.193426	0.384873
5830408C22Rik	-0.69399	-1.4983	-0.81707	1.093762	1.187995	1.233419	0.635188
Sfmbt2	-1.34723	-2.13272	-0.79676	2.750769	2.546976	2.95568	1.142296
Prf1	-1.04001	-1.81699	-0.78874	6.714892	7.148162	6.099177	2.775514
Vcpip1	-0.69331	-1.47003	-0.78871	13.38192	12.22252	13.73428	8.890143
Ifi27l2a	-1.07573	-1.84727	-0.78394	822.8041	834.247	880.5548	371.1673
Smchd1	-0.75446	-1.51379	-0.77116	6.484437	6.695154	7.950734	4.442319
Itk	-0.72167	-1.47819	-0.76955	1.294111	1.534505	1.609026	0.700686
Gm28068	-0.88803	-1.63165	-0.7565	2.113167	2.062572	2.15422	1.24417
Crebrf	-0.92226	-1.66135	-0.75147	20.88306	20.91485	21.70922	11.89824

Armc8	-1.02279	-1.76204	-0.75145	16.80195	17.90587	18.26222	8.692173
Zfp871	-0.91629	-1.65387	-0.74995	6.972818	5.538463	6.608518	3.417257
Got1	-0.77305	-1.50845	-0.74762	19.91861	19.43785	20.41036	10.90771
Mbnl3	-0.88216	-1.61698	-0.747	0.704428	0.733094	0.802011	0.429083
Phlpp2	-0.9887	-1.7229	-0.74615	0.981266	0.855153	1.074943	0.470132
Vamp1	-0.64567	-1.37475	-0.74158	4.403448	4.693737	4.781788	2.968159
Dennd2d	-1.08931	-1.81645	-0.73942	10.1142	11.43634	12.30019	5.510443
Cxcr4	-1.97642	-2.70254	-0.73865	319.2868	319.312	290.2084	84.64488
Mov10	-1.84689	-2.57045	-0.73614	53.19922	60.44998	56.38316	15.54602
Cmah	-0.73421	-1.45568	-0.73358	8.627364	8.734684	10.63105	5.79116
Gfpt1	-1.12073	-1.8366	-0.72807	13.42314	13.16692	15.66087	6.666034
1600014C10Rik	-0.61202	-1.32653	-0.72694	131.1854	141.5304	133.0374	82.24312
Ciita	-1.29787	-2.00291	-0.71734	4.403413	3.538225	3.515886	1.371262
Fryl	-0.62354	-1.32486	-0.71372	7.507671	7.153054	7.7234	4.608285
Rag1	-0.61068	-1.30734	-0.70857	3.880447	3.588823	4.284968	2.638212
Trpm2	-1.75396	-2.44924	-0.70801	144.5478	143.2723	125.1356	40.99017
Nhsl2	-2.12599	-2.81791	-0.70434	19.80035	21.077	23.45916	5.125689
Kmt2a	-0.59351	-1.27613	-0.69508	7.080038	6.966254	7.751702	4.735836
Trim30b	-0.85975	-1.53751	-0.68969	83.73481	82.19557	90.85101	51.56993
Utrn	-1.05957	-1.7349	-0.68775	6.376088	5.50389	5.788868	2.824864
Fcgr4	-0.96782	-1.6427	-0.68706	475.5467	483.8165	548.6023	251.6413
Rcbtb1	-0.76839	-1.43985	-0.68381	1.468168	1.570609	1.51118	0.954666
Kmo	-0.78876	-1.45893	-0.68227	2.005737	2.516216	2.671798	1.436441
Hist3h2a	-0.84333	-1.50418	-0.67359	2.483572	3.529728	3.391943	2.082322
Ikzf2	-0.71041	-1.36702	-0.66938	0.326362	0.406414	0.402537	0.232779
Khdc4	-0.89234	-1.54634	-0.66614	26.61347	25.76957	28.91848	16.18117
Entpd1	-0.69273	-1.33667	-0.65567	106.4251	83.32903	112.0673	65.85639
Mafk	-0.81181	-1.45047	-0.65146	45.42025	48.79786	39.64736	25.83778
Fyn	-0.77784	-1.41526	-0.65027	4.411254	4.153391	4.484708	2.263396
Rbm41	-0.70556	-1.34084	-0.64692	1.133001	1.04457	1.096582	0.772927
Pdpr	-0.65863	-1.28887	-0.64292	5.429621	4.542746	4.46268	3.203909
Atp11c	-1.14759	-1.77476	-0.63874	5.968912	5.968611	8.155808	3.229239
Sp100	-0.65461	-1.27501	-0.63239	74.23584	75.78807	80.1087	52.40879
Zcchc24	-0.60537	-1.21466	-0.62045	1.182568	1.13799	1.260292	0.859552
Stx17	-1.63217	-2.23859	-0.61881	23.08907	24.54409	24.57444	8.468184
Gm28043	-0.76696	-1.37307	-0.61816	2.433798	2.394684	2.88793	1.649731
Afdn	-0.81638	-1.41731	-0.6126	1.540874	1.453414	1.646167	0.987564
Tmem71	-1.7524	-2.34651	-0.60668	65.02637	71.39154	71.07195	23.30083
H2-DMb1	-1.02605	-1.61543	-0.60234	17.50661	16.51898	16.81887	7.578729
Lrch3	-0.7468	-1.33346	-0.59865	3.52091	3.323284	3.647182	2.320256
Zbtb20	-0.7312	-1.31382	-0.59451	0.68797	0.485406	0.680195	0.373416

N_WT_2_fpkm	N_WT_3_fpkm	N_KO_1_fpkm	N_KO_2_fpkm	N_KO_3_fpkm	KOvsWT_p	WTvsCon_p	KOvsCon_p
5.456912	5.752676	0.064337	0.025757	0.006003	2.21E-85	3.99E-17	8.43E-50
81.08391	93.1	0.592973	0.599895	0.559223	0	1.27E-11	0
0.570369	0.354778	0	0	0	7.24E-08	2.16E-07	3.53E-13
1.186533	1.316466	0.041862	0	0	4.66E-07	2.41E-12	1.94E-12
0.947836	1.236762	0	0.046504	0	1.51E-06	0.009509	7.94E-09
39.3968	42.88626	0.897881	0.754589	1.012939	0	4.76E-23	0
1.788884	2.02163	0.019419	0.064139	0.07972	4.04E-19	8.29E-10	1.53E-31
15.27714	17.1687	0.444356	0.344258	0.608055	4.5E-142	2.09E-23	2.6E-218
78.35618	84.97206	2.377653	2.598401	2.852855	0	1.2E-15	0
7.02643	11.57014	0.317308	0.480338	0.12212	3.29E-36	5.71E-05	3.92E-54
0.691636	0.247831	0.042851	0.015726	0	2.25E-07	0.000412	9.91E-14
1.033736	1.021097	0.039786	0.087605	0	1.37E-14	2.02E-14	1.48E-31
0.609051	0.335543	0	0	0	0.036655	0.025621	2.1E-05
79.3709	88.40367	3.382335	4.277972	7.313906	1.41E-32	1.66E-33	1.47E-50
10.17548	8.265178	0	0.182416	1.360388	1.79E-10	0.000242	3.82E-07
2.931557	4.097503	0.229838	0.212552	0.198141	3.46E-61	3.23E-34	4.9E-152
1.757533	1.66269	0.099211	0.139014	0.046282	2.67E-30	6.78E-50	5.22E-98
0.150418	0.098654	0	0.020031	0	0.012805	0.024536	4.47E-05
0.139516	0.102484	0.023627	0	0	0.01674	2.07E-05	5.03E-07
165.6526	187.6928	10.72441	8.477897	19.03368	1.11E-25	1.12E-20	5.59E-37
6.695143	7.619737	0.537065	0.410608	0.59712	5.11E-75	4.33E-48	7.6E-180
66.31372	64.88476	4.547622	4.241441	6.246724	6.1E-198	5.99E-14	4.6E-253
0.073753	0.203162	0	0	0.032046	0.031203	0.001431	9.45E-06
109.7396	104.8817	7.301159	8.410818	14.23512	4.3E-28	1.69E-39	4.15E-50
141.3382	154.8386	11.30722	11.51417	20.28757	8.66E-30	3.95E-15	3.15E-41
427.9607	500.4746	43.66727	41.39089	57.17311	3.6E-175	1.02E-13	3E-263
0.135066	0.263541	0.014296	0	0.044015	0.002163	0.009993	2.87E-07
0.657035	0.721334	0.012094	0.039945	0.173772	1.04E-08	0.003099	1.93E-16
5.969613	6.09644	0.78406	0.514248	0.582108	3.53E-47	1.24E-29	1.9E-128
2.994238	3.2555	0.381891	0.268613	0.511691	4.06E-40	5.98E-08	3.01E-71
4.106697	5.582203	0.634425	0.495112	0.52748	5.6E-55	3.66E-15	7.8E-141
8.943685	6.362018	0.209528	0.230677	2.580455	0.00578	4.41E-30	1.17E-05
0.161678	0.118764	0.02738	0	0.0281	0.041198	0.047524	0.00036
60.34639	62.64454	9.872617	6.242411	6.709184	4.79E-79	2.64E-77	3.4E-236
3.918821	4.271675	0.65976	0.512721	0.378385	1.21E-28	1.4E-07	7.82E-56
0.473214	0.289674	0.076321	0	0.097911	0.000132	0.000135	8.85E-13
3.708364	3.302972	0.265821	0.365815	1.017357	1.59E-06	2.21E-31	1.89E-13
0.147288	0.077281	0.035633	0	0.018285	0.03662	0.002765	6.8E-06
0.534219	0.338294	0.046794	0.085862	0.064033	5.1E-05	3.63E-09	3.5E-19
4.814406	2.652386	0.271769	1.196802	0.278915	0.004722	0.020173	1.05E-06
6.71676	4.710898	0.377756	1.028162	1.539989	2.49E-06	7.61E-38	1.29E-15
1.282484	0.689733	0.108593	0.102475	0.238818	2.59E-12	0.00337	1.09E-24
0.29944	0.175662	0.014086	0.077539	0.014456	0.000209	6.64E-07	4.16E-17
5.408196	7.940813	1.202861	0.859618	1.126204	1.99E-27	9.94E-05	2.25E-58
4.005949	6.831146	0.484569	2.133921	0.994623	0.004103	0.000639	2.52E-09
55.62839	72.37621	11.5484	10.72903	10.16985	3.4E-94	3.36E-31	0
0.858924	0.867542	0.163638	0.160138	0.149281	5.87E-08	0.011916	6.17E-14
0.442324	0.33362	0	0.073631	0.137279	0.000242	0.017509	4.32E-09
4.621188	3.173144	0.433502	0.483539	1.229334	7.33E-07	1.03E-29	2.91E-16
0.21812	0.189153	0.030782	0.033889	0.052652	0.000431	0.04448	1.04E-07
0.199873	0.183526	0.028207	0.031054	0.057897	0.034309	0.00058	7.8E-07
183.7722	186.5614	36.85304	30.73414	39.09187	1.2E-134	3.95E-25	2.6E-220
91.52877	69.66142	9.937614	14.9727	23.53491	1.62E-10	7.45E-32	2.37E-24

0.275166	0.237348	0.021181	0.046638	0.072461	2.6E-05	1.2E-05	4.64E-16
0.679409	0.594135	0.19176	0	0.224917	0.000595	0.000205	7.8E-12
12.91553	9.192067	1.427396	1.732651	4.372259	1.67E-05	5.17E-27	1.12E-13
1.460496	1.122735	0.086278	0.221636	0.531282	1.15E-05	0.001364	7.85E-13
0.751438	0.752704	0	0.191044	0.296819	0.010843	0.00137	6.57E-08
156.0138	147.0831	36.32954	27.03744	29.59729	1.32E-84	1.58E-12	9.1E-116
10.72692	10.23976	2.650268	2.637596	1.999442	2.58E-54	1.15E-08	1.7E-120
107.1358	105.483	18.72222	20.41277	31.30043	2.21E-51	3.19E-35	8.3E-106
281.5041	291.6085	57.1744	51.32341	83.48888	3.22E-56	4.84E-26	1.8E-101
1.743508	1.225922	0.305898	0.190351	0.532337	1.65E-10	4.32E-85	2.1E-119
91.73577	87.73381	20.72651	17.13172	21.73542	5.17E-91	1.53E-16	3.4E-149
0.36958	0.36249	0.06401	0.078301	0.09489	8.69E-07	6.27E-68	2.76E-75
11.10459	11.4158	2.404728	2.314143	3.258069	6.6E-57	6.23E-23	5.3E-119
165.4093	145.3389	29.02328	33.23094	56.20429	1.76E-10	2.83E-20	1.17E-21
4.806804	3.412039	0.698374	0.636589	1.949839	0.000203	1.07E-19	4.8E-10
6.636314	5.578594	1.187156	0.871324	2.76165	4.38E-07	7.39E-07	4.69E-21
0.419706	0.359687	0.023692	0.052167	0.182363	0.002893	1.69E-10	5.79E-19
8.047962	7.574476	2.470256	1.219131	2.360374	3.04E-09	0.004872	7.89E-17
0.86599	0.775768	0.160961	0.255966	0.256967	5.72E-06	0.000376	3.25E-15
3.354486	3.310798	0.875883	0.78521	0.821866	2.22E-22	4.69E-07	1.61E-43
12.64193	12.15138	3.577405	3.18758	3.828625	5.43E-46	7.59E-08	7.45E-92
0.452195	0.406377	0.122199	0.125565	0.083608	4.52E-06	3.64E-10	2.33E-25
5.495029	4.185366	0.900057	0.688597	2.317144	0.000388	6E-112	1.58E-18
483.8004	560.0814	148.2098	143.1274	138.0903	6.3E-119	1.04E-17	1.7E-239
2.584396	2.241188	0.668648	0.669218	0.623846	5.27E-05	0.000525	2.87E-13
8.665931	8.368736	2.656774	0.974981	3.375839	4.02E-06	8.18E-42	3.07E-59
67.50283	69.13866	19.22096	16.95804	18.70054	3.84E-79	7.88E-10	4.4E-120
8.819159	7.058266	0.874572	1.388725	4.833073	0.011029	2.66E-25	1.79E-05
148.2934	135.3366	40.99289	36.39742	39.669	5.03E-91	9.28E-14	6.2E-143
1.550928	0.817298	0.17129	0.25144	0.468786	5.97E-06	9.14E-05	1.57E-20
6.066152	8.664462	2.282857	0.628321	2.928612	0.034669	0.010439	5.26E-06
7.054404	7.848785	1.923807	1.866203	2.568097	1.47E-29	9.87E-12	1.2E-66
0.035686	0.055897	0.014219	0.015655	0.018242	0.000963	2.04E-08	4E-19
3.515611	2.088754	0.350211	0.578341	1.677296	0.000474	0.000961	3.56E-11
6.356884	4.459823	0.879191	0.903406	3.559116	0.010567	1.82E-16	1.02E-05
18.08574	18.39421	4.598492	5.87459	7.791493	6.23E-19	2.48E-10	2.68E-56
9.487903	7.288671	1.312444	2.473878	4.632717	0.000897	5.92E-05	3.35E-06
26.25667	30.80146	8.635384	8.767176	8.149792	2.78E-69	6.25E-16	6.5E-156
0.514668	0.363518	0.092186	0.101491	0.292431	0.000142	0.004655	1.3E-11
0.095034	0.087261	0.012706	0.027976	0.026079	0.016464	0.016916	8.49E-07
11.61718	13.89423	4.11187	3.351417	4.593038	2.48E-28	9.52E-51	2.9E-142
2.089375	2.494035	1.061489	0.779089	0.393396	1.63E-06	5.43E-19	2.13E-38
1.436924	1.640336	0.591906	0.398232	0.371233	7.48E-05	5.25E-09	2.9E-21
0.6046	0.508888	0.255968	0.140903	0.175133	0.021838	7.51E-06	9.55E-11
29.82454	31.14248	9.929954	8.690756	8.871373	4.21E-41	6.79E-09	1.67E-76
0.892081	1.300954	0.399895	0.44026	0.228006	0.003062	0.012522	5.78E-08
0.82831	0.984259	0.082513	0.408787	0.338731	0.009616	7.53E-12	1.41E-18
49.61256	40.17216	8.665139	10.86243	24.07261	0.000655	1.06E-22	1.1E-07
4.643227	6.326433	1.775556	0.977388	2.212729	0.000454	8.22E-10	3.04E-21
71.626	68.65709	23.67702	21.3646	28.64089	6.39E-42	1.12E-12	5.3E-99
6.400876	7.36018	3.132575	2.025469	1.658507	4E-16	3.1E-221	5.72E-61
20.22051	20.98845	7.774949	6.966169	8.870723	3.36E-21	1.07E-06	1.31E-55
0.105891	0.123159	0.04782	0.052646	0.042943	0.007693	1.65E-07	3.72E-15
3.480293	3.16369	1.178756	1.086855	1.300485	2.88E-15	2.47E-28	1.33E-76
0.526952	0.392775	0.141731	0.127141	0.183169	1.64E-06	5.8E-37	2.9E-65
0.160541	0.242174	0.048548	0.064139	0.11958	0.010932	3.06E-74	1.04E-67

0.698673	0.766608	0.253008	0.262161	0.294028	1.58E-13	4.18E-13	2.06E-45
1.255622	1.237545	0.253603	0.236247	0.870909	6.78E-05	3.63E-13	5.01E-06
5.73195	4.834955	2.253974	1.467156	1.604076	4.25E-14	1.4E-05	5.24E-32
3.541444	3.042002	1.305872	1.011704	1.141662	1.07E-05	4.31E-06	1.05E-18
0.351152	0.423192	0.092917	0.122754	0.267008	0.012669	3.83E-11	2.13E-18
4.285576	3.589307	1.503123	1.320533	1.573815	2.44E-15	2.95E-09	6.44E-41
342.4257	396.5524	135.5491	126.3297	135.3548	4.49E-68	4.09E-16	3.2E-152
35.69613	35.20971	14.83445	11.79973	10.16239	9.54E-26	1.7E-206	2.6E-288
18.84371	14.12849	3.887596	3.9495	12.16974	0.011517	1.73E-12	2.66E-05
1.031115	1.146806	0.245939	0.175996	0.83294	0.000751	4.08E-10	4.18E-05
3.764364	3.749808	1.542537	1.3602	1.440547	4.24E-25	2.97E-15	1.96E-70
36.35207	34.52584	9.912169	12.19653	19.20452	1.41E-06	2.6E-124	1.09E-34
0.500948	0.52744	0.073523	0.10585	0.394695	0.001375	6.48E-05	0.001348
17.40844	18.19969	6.115934	5.536532	7.60077	6.72E-29	2.18E-33	2.7E-100
220.1711	217.5048	63.32118	57.32765	125.8543	0.000235	2.19E-20	1.49E-07
152.6824	148.1337	42.01891	46.0788	89.38843	0.000152	1.87E-41	4.65E-11
1.14367	0.877507	0.391334	0.284789	0.524155	4.53E-08	9.86E-15	2.04E-38
1.245148	1.465369	0.460333	0.441405	0.792478	5.74E-06	8.08E-22	4.2E-43
11.37678	11.09969	3.9202	4.668048	4.405006	6.63E-42	1.08E-21	2.11E-96
1.969659	1.899905	0.791775	0.686228	0.639703	2.1E-07	1.58E-08	2.72E-25
1.325314	1.94707	0.448876	0.564783	0.888455	0.000608	0.000111	2.24E-12
2.668954	2.135579	0.90396	0.675317	0.762065	0.000281	0.011132	3.55E-11
13.98599	13.54836	5.166822	4.52917	6.082502	2.35E-30	1.25E-40	5E-104
29.08282	29.27521	10.92277	10.55757	13.30119	4.2E-41	1.57E-27	3.82E-97
0.733141	0.597175	0.235295	0.253533	0.231206	3.08E-07	0.005002	5E-16
54.70814	57.9846	22.16622	23.28222	23.99647	8.69E-66	1.02E-23	2.5E-135
99.96407	99.07671	26.55772	31.0703	60.58237	0.000795	3.78E-52	1.47E-09
28.95463	24.90598	8.292577	8.550058	18.24144	0.001696	1.14E-23	1.98E-08
212.6031	185.1724	94.04914	74.174	82.63175	2.04E-36	2.13E-63	9.7E-162
74.91992	68.79235	32.32593	30.00474	21.81185	2.66E-21	2.47E-16	1.78E-57
13.58042	10.52926	4.693482	4.747395	7.165148	8.15E-11	2.36E-08	2.27E-38
6.105953	5.490274	1.784653	2.040685	3.836106	0.000295	2.5E-09	1.96E-08
26.86706	26.73262	9.729887	8.857583	16.1277	2.23E-13	4.52E-11	4.46E-30
1.470436	1.582578	0.540892	0.595488	0.796015	4.86E-08	1.01E-32	9.83E-61
1.308569	1.158631	0.63315	0.348529	0.568575	0.007522	3.47E-06	1.63E-12
4.487338	3.566433	1.488234	1.707228	2.059121	1.96E-19	1.37E-21	6.09E-65
2.248388	1.692887	0.609213	0.768517	1.081133	1.26E-06	2.28E-07	7.51E-23
38.55222	36.92144	17.51237	14.89776	18.903	2.11E-29	4.62E-14	2.85E-77
6.005692	5.507094	2.102352	2.254441	3.054319	8.09E-18	2.55E-48	1.69E-86
2.582562	2.186901	0.631727	0.829241	1.74553	0.000281	0.000148	1.18E-11
8.89635	9.325777	4.097193	3.664989	3.558866	4.27E-24	2.93E-18	4.33E-76
11.35456	11.04389	4.706898	4.763725	5.534698	1.02E-26	3.75E-30	2.66E-92
2.464094	1.686636	0.79664	1.127637	1.362648	0.001023	0.015041	1.43E-09
1.036527	1.608913	0.438832	0.414109	0.664835	0.001752	0.00014	1.12E-13
1.341806	0.966935	0.258871	0.435418	0.797035	0.000105	2.09E-15	1.35E-08
4.162376	4.5738	1.829624	1.717458	2.174223	1.73E-10	5.08E-18	7.06E-47
11.75027	11.02398	4.848077	5.031665	5.457614	4.98E-40	6.12E-12	7.52E-72
2.566005	2.54145	1.049746	1.048197	1.027241	2.08E-05	0.001692	5.56E-14
5.142007	5.176867	1.611294	2.245726	3.298537	2.39E-09	2.38E-12	7.71E-26
0.550065	0.478493	0.241051	0.256386	0.259969	1.12E-06	7.9E-08	2.86E-26
5.54687	5.938456	2.398333	2.508395	2.399867	5.78E-11	8.47E-07	5.51E-31
0.450011	0.165282	0.177819	0.097884	0.143389	0.043958	6.68E-07	1.4E-13
147.6986	133.8239	72.84072	63.75149	71.11931	4.22E-29	5.52E-80	8.6E-215
0.325554	0.516329	0.242245	0.202322	0.120021	0.005556	0.00367	1.04E-08
0.791012	0.357205	0.197639	0.145059	0.43948	0.014714	0.003083	5.44E-08
0.106037	0.128521	0.071828	0.043493	0.058974	0.012978	1.19E-13	2.64E-22

5.121022	4.909631	1.657941	1.755087	3.959353	0.000157	8.16E-07	8.47E-14
2.525578	2.756565	0.443839	0.844013	2.401784	0.017602	1.98E-06	0.000937
3.270778	2.514362	1.706776	1.028162	1.454207	0.000211	4.58E-05	1.66E-14
0.557807	0.45243	0.157438	0.216662	0.282763	0.003275	0.009457	3.47E-08
0.734573	0.761525	0.501604	0.198805	0.350061	0.016895	0.012691	2.59E-06
1.813692	1.828915	0.795283	0.724599	1.013209	1.16E-05	2.28E-14	1.94E-31
17.15082	19.23904	9.049559	8.269686	8.553342	9.72E-15	5.06E-72	6.3E-141
0.234619	0.178761	0.118351	0.125644	0.091097	0.004021	0.003477	4.23E-09
11.03545	11.08475	4.318112	4.177262	9.473592	0.011563	1.84E-07	2.42E-05
0.277078	0.282684	0.109485	0.109057	0.15517	0.00647	0.021372	8.93E-07
3.264942	3.600877	1.305739	1.671396	1.853024	1.51E-13	2.2E-18	2.22E-50
0.532742	0.48917	0.135327	0.215204	0.493817	0.036385	0.006478	5.75E-06
10.25884	11.80838	5.4084	5.646921	5.540012	3.9E-21	1.6E-26	1.34E-87
3.300216	2.921795	1.307871	1.581512	1.841028	9.82E-11	1.79E-10	1.13E-33
8.293567	11.60419	6.219895	2.945255	4.598836	0.000356	7E-05	3.7E-14
28.69023	37.10538	17.10851	15.66298	13.88494	1.06E-15	3.05E-11	5.63E-65
121.446	113.9353	49.16851	57.86181	85.63398	0.000231	4.23E-13	5.44E-12
3.860492	3.868964	2.102807	2.051735	1.881949	5.67E-12	9.69E-10	6.76E-35
0.608237	0.614339	0.480682	0.2079	0.229043	0.031583	0.044307	6.5E-05
3.816764	3.346603	2.410599	1.633181	1.536049	7.13E-07	4.31E-12	2.75E-27
0.815838	0.716892	0.319402	0.384353	0.5565	0.000394	0.001746	7.41E-11
4.206329	4.117659	2.089894	2.074	2.718826	1.78E-05	0.00147	2.94E-12
1.123361	0.844939	0.414881	0.523601	0.540028	0.000452	9.2E-05	1.07E-13
1.480938	1.464416	0.434064	0.796463	0.940456	0.047028	0.005194	2.7E-06
26.21435	25.8462	10.62252	9.769974	19.98674	0.003702	2.16E-12	1.5E-06
3.161556	2.668269	1.606202	1.630374	1.461383	3.91E-08	2.53E-28	6.94E-61
0.97823	1.042764	0.495078	0.419269	0.62535	0.000324	3.47E-11	4.18E-23
0.357738	0.355216	0.211332	0.143476	0.20243	0.000383	7.46E-05	4.58E-13
11.25555	10.80214	5.558957	5.951148	5.838382	1.19E-25	2.4E-111	1.3E-196
2.188154	2.262448	1.276712	1.131321	1.166474	1.83E-05	2.69E-11	4.79E-26
24.61523	20.01326	10.40394	11.54966	15.61316	3.2E-11	1.23E-23	1.31E-51
1.841999	1.439929	0.632306	0.997785	1.103187	0.002993	0.000457	1.97E-10
0.548807	0.604706	0.170388	0.375174	0.357686	0.006899	0.0027	1.25E-07
0.226417	0.137481	0.108226	0.091916	0.12694	0.009013	9.64E-15	2.15E-28
0.212733	0.277975	0.124704	0.129664	0.163535	0.032193	0.027964	1.59E-05
6.681339	6.239061	2.834002	3.657699	3.968415	1.22E-13	9.79E-36	6.72E-59
3.507639	3.268119	1.572376	1.202145	1.793028	0.01701	3.27E-08	6.79E-19
0.82104	0.788953	0.452691	0.444987	0.414818	0.014182	0.002292	5.28E-08
1.018871	0.891131	0.404056	0.667261	0.610815	4.14E-06	7.11E-24	2.26E-39
6.037258	5.550463	2.755567	3.508612	3.521823	5.92E-12	3.63E-12	2.08E-35
3.411723	3.242796	1.578956	1.995449	2.068584	2.04E-10	3.15E-26	9.32E-51
0.650277	0.620355	0.364691	0.283414	0.344926	0.003204	5.56E-05	2.88E-12
6.139878	5.63031	3.278282	3.52656	3.80562	3.3E-14	1.7E-15	5.34E-59
8.119582	9.292356	4.732888	3.345764	8.589838	0.003356	1.3E-06	5.65E-12
1.551693	0.823703	0.554291	0.565037	1.095596	0.030456	1.56E-07	1.72E-14
10.66967	9.820633	4.537161	5.366771	7.793076	7.04E-08	1.67E-21	1.33E-26
0.382613	0.367289	0.191438	0.226974	0.231737	0.004134	2.4E-16	2.47E-27
0.858486	0.668027	0.332654	0.515437	0.39198	0.01655	0.009932	6.61E-07
0.96362	1.110585	0.540203	0.638096	0.693011	5.54E-05	7.32E-20	6.18E-38
2.699719	4.160264	1.888393	2.057119	1.693246	0.000746	3.11E-08	9.74E-24
8.263257	7.010364	3.793445	4.506975	5.818172	6.29E-08	1.31E-11	3.76E-28
431.3125	393.5099	241.7339	198.8372	260.2733	1.53E-12	2.28E-39	1.37E-69
4.250131	3.746419	1.935608	2.295124	3.126178	2.27E-07	4.19E-13	2.83E-25
0.781779	1.185994	0.623586	0.433042	0.521833	0.009576	0.002948	1.79E-09
1.298776	0.858637	0.601181	0.661863	0.767475	0.011714	0.000171	5.7E-11
11.15622	10.23077	6.651448	6.438017	6.863293	5.95E-15	4.63E-27	2.28E-77

7.607071	9.577588	4.836785	5.255732	5.432125	1.59E-10	1.54E-23	3.04E-68
3.949317	2.704191	1.404492	2.05116	2.588684	0.000384	1.21E-09	1.23E-19
10.79914	13.00912	6.979394	7.238438	6.66464	1.58E-08	8.51E-12	2.87E-38
0.499557	0.280316	0.187997	0.316927	0.223088	0.027712	0.000669	4.13E-09
0.545659	0.441698	0.231015	0.341341	0.305721	0.009884	9.93E-06	6.41E-12
2.977289	2.865266	1.763169	1.8299	1.726576	6.53E-10	9.06E-10	2.64E-31
5.04335	5.23912	3.365453	3.104097	3.077857	1.95E-10	1.81E-24	3.72E-54
76.22951	73.56851	46.81878	45.00767	49.9743	2.34E-15	1.7E-123	4.5E-207
15.89295	15.51696	9.8478	7.523717	11.06573	9.13E-09	7.4E-113	9.9E-98
5.252399	5.658839	3.218701	3.453409	3.471477	5.56E-13	2.97E-12	2.18E-37
6.432337	6.193817	3.620795	4.289452	3.85209	3.01E-12	1.03E-31	3.81E-62
88.34246	93.10622	46.07048	45.89497	68.8539	9.88E-08	6.52E-15	6.64E-24
1.565048	1.694078	0.851134	1.031936	0.961973	2.01E-05	4.12E-20	4.33E-40
5.142972	4.682007	2.390374	3.055993	3.43869	3.31E-08	2.04E-12	1.39E-26
2.634663	2.368603	1.461333	1.420569	1.834814	1.18E-05	5.56E-06	4.94E-17
41.40505	39.22568	24.25364	25.55344	25.34416	2.27E-22	7.2E-109	3.5E-177
5.435632	4.084193	2.672446	3.112325	3.286914	1.95E-08	6.65E-87	6.2E-157
5.309247	4.306985	2.471156	3.358333	3.121159	4.62E-08	1.78E-09	6.02E-27
44.80103	44.16345	25.67191	25.84406	36.42498	7.4E-08	4.09E-24	5.5E-36
3.003291	2.594867	1.522194	1.771698	1.984362	3.37E-08	2.11E-26	1.65E-48
244.8194	269.1877	180.7565	154.8341	144.3787	3.35E-12	2.84E-31	1.75E-56
0.787288	0.908255	0.58116	0.442229	0.640299	0.007692	0.000146	2.18E-10
1.181384	1.509233	0.858971	0.778086	0.959672	0.003173	7.02E-05	5.82E-12
1.6395	1.479453	1.22068	0.711473	1.351037	0.036899	0.000866	1.34E-07
0.245509	0.21104	0.137113	0.141214	0.158876	0.046209	0.011539	7.94E-06
13.77522	13.53546	9.049899	8.903086	9.711487	7.78E-11	5.26E-21	9.54E-60
60.36799	59.21381	35.69726	39.89839	43.23009	1.75E-12	2.94E-11	1.33E-30
24.10288	25.81538	14.02281	15.40925	19.25632	4.09E-08	1.44E-17	3.26E-32
2.87805	2.42563	1.565769	1.555322	1.739849	0.000434	4.54E-07	2.8E-18
0.667112	0.553813	0.255351	0.460024	0.571781	0.040962	0.001922	1.12E-06
3.148897	2.736263	1.215662	2.159385	2.505746	0.003556	4.24E-06	3.12E-10
3.052987	2.719494	1.770214	1.763286	2.299794	3.35E-05	3.38E-16	1.13E-29
48.20969	44.59105	27.64672	25.13118	41.76179	2.23E-05	1.09E-15	3.35E-19
0.678898	0.794494	0.450861	0.670099	0.416445	0.046159	0.015473	1.62E-05
7.379547	7.282778	5.140757	4.983164	5.080537	2.59E-08	7.72E-68	5.4E-120
1.53491	1.315414	0.905826	0.945228	1.107492	0.001471	3.62E-06	1.05E-14
0.842603	0.784585	0.467268	0.553154	0.706442	0.004479	9.04E-07	7.72E-14
19.89568	17.97018	14.64604	13.11526	12.77298	4.74E-07	4.8E-66	8.3E-126
8.333299	8.889513	6.952339	4.683848	4.827773	0.002037	1.23E-13	1.52E-21
2.248422	1.643195	1.041273	1.668235	1.435507	0.003522	1.44E-06	1.19E-14
0.418491	0.317199	0.213948	0.264987	0.26246	0.00157	3.29E-05	1.16E-12

Supplemental table S5

name	PR_K27me_LogFC_KO/WT	PR_K27me_KO	PR_K27me_WT	GB_K27me_LogFC_KO/WT	GB_K36_LogFC_KO/WT	GB_K27me_KO	GB_K27me_WT	GB_K36_KO
00001L05R	0.8682	0.0049	0.0027	1.2563	-0.625	0.0073	0.0031	0.0059
00040D17R	0.6467	0.0328	0.0209	0.6253	-0.65	0.022	0.0143	0.0061
00066M21F	#DIV/0!	0.002	0	4.844	-1.282	0.0025	######	0.0022
33434E20R	0.2147	0.0029	0.0025	1.0401	-1.065	0.0046	0.0022	0.0118
ABCB10	3.5264	0.0092	0.0008	1.3232	-0.662	0.0044	0.0018	0.0057
ABHD13	1.3304	0.0056	0.0022	0.6979	-0.669	0.0026	0.0016	0.0041
ACAT2	1.2811	0.0091	0.0038	0.9236	-1.063	0.0028	0.0015	0.0052
ADAR	2.1003	0.021	0.0049	0.6981	-1.172	0.0078	0.0048	0.008
AKR1C19	0.5171	0.0008	0.0006	1.8362	-1.566	0.001	0.0003	0.0013
ALDH3A2	0.3609	0.0051	0.004	0.835	-0.73	0.0056	0.0031	0.0078
ALG11	2.7358	0.0167	0.0025	0.6909	-0.725	0.0025	0.0016	0.0059
ALG3	1.6919	0.0147	0.0046	0.9358	-0.929	0.0126	0.0066	0.0081
ALG8	0.2991	0.0031	0.0025	0.9858	-0.92	0.0027	0.0014	0.0042
AMIGO1	0.2736	0.0199	0.0164	0.6256	-1.332	0.0251	0.0163	0.0076
APOE	1.1023	0.0156	0.0073	1.014	-0.684	0.0204	0.0101	0.0101
ARHGAP5	0.2233	0.0005	0.0004	1.0546	-0.585	0.0004	0.0002	0.0016
ASNS	1.2787	0.0121	0.005	0.7251	-1.045	0.0024	0.0014	0.0034
ATP2A2	0.8031	0.0024	0.0014	0.6941	-1.049	0.0018	0.0011	0.0093
B4GALNT1	0.3014	0.0088	0.0072	0.8749	-1.816	0.0145	0.0079	0.0071
BLMH	1.7148	0.0052	0.0016	1.5648	-1.141	0.0054	0.0018	0.0062
BUB1	-0.758	0.0058	0.0097	1.0664	-0.822	0.0018	0.0009	0.003
CABLES2	1.0199	0.0153	0.0076	1.1868	-1.2	0.0146	0.0064	0.013
CAMSAP3	1.2879	0.0576	0.0236	1.0909	-0.869	0.0205	0.0096	0.0085
CBLC	1.0307	0.0035	0.0017	0.7768	-1.391	0.0033	0.0019	0.0079
CCDC58	0.8665	0.0029	0.0016	0.9114	-0.807	0.002	0.0011	0.0047
CCNG1	0.0063	0.0034	0.0034	1.1943	-0.803	0.0029	0.0013	0.0032
CDH1	3.0551	0.017	0.002	1.4589	-1.342	0.0032	0.0012	0.0053
CDH17	-0.971	0.0006	0.0013	0.6827	-0.624	0.0008	0.0005	0.0074
CEBPZ	-0.302	0.0016	0.0019	0.8081	-1.21	0.0023	0.0013	0.0045
CHORDC1	1.0314	0.003	0.0015	1.1556	-0.589	0.0008	0.0003	0.0024
CHP1	0.5854	0.0055	0.0036	1.0916	-1.179	0.003	0.0014	0.0052
CHRM4	2.5717	0.0845	0.0142	1.7695	-0.626	0.0474	0.0139	0.0096
CIDEC	0.6423	0.0064	0.0041	1.2023	-1.383	0.0073	0.0032	0.0071
CLU	0.3038	0.0034	0.0027	1.0207	-1.697	0.0038	0.0019	0.009
CMAS	0.3603	0.0108	0.0084	0.668	-0.836	0.0026	0.0016	0.0097
COL27A1	1.1752	0.0408	0.0181	0.6352	-0.82	0.0035	0.0022	0.0049
COMMD2	0.9054	0.007	0.0038	0.736	-1.142	0.0021	0.0013	0.0029
CPD	1.9284	0.0095	0.0025	1.315	-0.656	0.0034	0.0014	0.0041
CRB3	2.1361	0.0363	0.0082	2.5168	-0.9	0.041	0.0072	0.0079
CREB3L3	-0.139	0.0019	0.002	1.0768	-0.595	0.0061	0.0029	0.0073
CWC22	1.8952	0.003	0.0008	0.8586	-0.66	0.0024	0.0013	0.0044
CXADR	1.4537	0.0034	0.0013	0.9342	-0.677	0.0026	0.0014	0.0074
CYP2C65	2.7661	0.0046	0.0007	2.7153	-0.67	0.0038	0.0006	0.003

CYP2S1	2.6255	0.0175	0.0028	1.4365	-3.144	0.0066	0.0024	0.0037
CYP3A13	0.5157	0.0024	0.0017	0.7522	-1.392	0.0023	0.0014	0.0035
CYP4F16	0.9347	0.011	0.0057	1.1516	-1.1	0.0051	0.0023	0.0048
DCBLD1	0.3529	0.0017	0.0014	0.6354	-0.682	0.0009	0.0006	0.0034
DDX3X	-0.554	0.0009	0.0014	0.7306	-1.047	0.0007	0.0004	0.0078
DEGS2	2.5703	0.0236	0.004	1.1695	-0.709	0.0105	0.0047	0.0077
DEPDC7	2.1999	0.0052	0.0011	0.7063	-0.957	0.0022	0.0013	0.0052
DHX32	-0.803	0.0013	0.0023	0.6387	-1.066	0.002	0.0013	0.0044
DHX33	1.5441	0.0023	0.0008	1.2938	-0.708	0.0027	0.0011	0.0081
DIAPH1	0.3182	0.0035	0.0028	0.9605	-0.902	0.0024	0.0013	0.0043
E2F5	0.7938	0.0042	0.0024	0.6304	-0.979	0.0013	0.0008	0.0026
EFNB1	1.6567	0.0083	0.0026	2.6971	-1.268	0.0044	0.0007	0.0031
EI24	0.631	0.0055	0.0035	1.5476	-1.221	0.0016	0.0005	0.0018
EIF3J1	1.0502	0.0042	0.002	0.7338	-0.665	0.0018	0.0011	0.0041
ELF3	2.2583	0.0103	0.0022	2.164	-1.751	0.0112	0.0025	0.0134
ENTPD2	1.5922	0.0782	0.0259	1.5734	-0.604	0.0753	0.0253	0.0106
ENTPD5	1.1235	0.0037	0.0017	0.8382	-1.167	0.0018	0.001	0.003
EPM2AIP1	0.8562	0.0045	0.0025	1.3113	-1.259	0.0041	0.0016	0.0037
ERBB2	1.7598	0.0245	0.0072	0.9299	-1.58	0.013	0.0068	0.0152
ERBB3	3.6843	0.0373	0.0029	2.9554	-2.253	0.02	0.0026	0.0038
ESRP2	3.6612	0.0259	0.002	3.8541	-1.476	0.0324	0.0022	0.0091
EXOSC9	-0.101	0.0055	0.0059	1.4367	-0.831	0.0068	0.0025	0.0086
FABP5	1.1648	0.0242	0.0108	1.2554	-0.898	0.0174	0.0073	0.0027
FADS1	2.3872	0.0149	0.0028	2.0564	-1.039	0.0114	0.0027	0.0098
FADS2	2.3724	0.0297	0.0057	1.8724	-1.033	0.0088	0.0024	0.0087
FADS6	2.0988	0.0453	0.0106	1.4452	-0.843	0.0204	0.0075	0.0119
FAM83H	1.6182	0.0321	0.0105	1.5124	-1.281	0.0312	0.011	0.0131
FANCD2	1.3516	0.0029	0.0011	0.9707	-0.626	0.0028	0.0014	0.0063
FBP2	2.4886	0.0038	0.0007	2.5721	-0.743	0.0047	0.0008	0.0033
FDPS	2.096	0.0131	0.0031	1.7522	-1.962	0.0129	0.0038	0.0094
FERMT1	3.1873	0.0249	0.0027	1.522	-2.331	0.0057	0.002	0.0067
FGFR4	1.5825	0.0099	0.0033	0.9877	-0.998	0.0043	0.0022	0.0054
FLRT3	1.9854	0.0018	0.0005	1.1097	-1.073	0.001	0.0005	0.0052
FUNDC1	-0.138	0.0005	0.0005	1.6095	-1.624	0.0002	######	0.0006
FZD8	1.1577	0.011	0.0049	0.6535	-1.081	0.0101	0.0064	0.0041
G2E3	1.2317	0.0027	0.0011	0.9292	-0.626	0.0008	0.0004	0.0049
GALNT3	-0.024	0.003	0.0031	0.6157	-0.826	0.0016	0.001	0.0041
GALNT4	-0.485	0.0008	0.0011	0.9498	-1.751	0.0012	0.0006	0.0021
GATA4	4.8214	0.1254	0.0044	3.3314	-1.86	0.0178	0.0018	0.0028
GATA5	5.0708	0.1606	0.0048	4.5033	-2.154	0.088	0.0039	0.0077
GATA5OS	4.9829	0.1583	0.005	4.301	-0.588	0.123	0.0062	0.0083
GCA	0.759	0.0031	0.0018	1.0729	-0.608	0.0016	0.0008	0.0033
GCAT	2.0122	0.0223	0.0055	1.4856	-1.043	0.0574	0.0205	0.0097
GCLM	-0.513	0.0041	0.0058	0.6345	-1.725	0.0031	0.002	0.0052
GM13420	3.806	0.0461	0.0033	3.3003	-0.593	0.0239	0.0024	0.0115
GM14288	#DIV/0!	0.0001	0	2.0351	-0.977	######	######	######
GM14295	0.4446	0.0005	0.0003	1.6198	-1.02	0.0002	######	0.0016
GM3776	-0.729	0.0008	0.0013	1.0314	-1.494	0.001	0.0005	0.0021

GM4787	1.3491	0.0006	0.0002	1.6199	-0.709	0.0008	0.0003	0.0043
GMCL1	0.3142	0.0033	0.0026	0.9683	-0.786	0.0032	0.0016	0.0053
GPATCH4	1.1389	0.0073	0.0033	0.7852	-1.279	0.0074	0.0043	0.0061
GRAMD1B	1.0939	0.0068	0.0032	0.7174	-0.601	0.0051	0.0031	0.0031
GRAMD2	1.6822	0.0038	0.0012	0.8608	-0.751	0.003	0.0016	0.0038
GRB7	3.1818	0.1069	0.0118	2.6869	-2.073	0.0736	0.0114	0.0146
GUF1	0.9117	0.0041	0.0022	0.6475	-0.661	0.0009	0.0006	0.0027
HACD3	-0.084	0.0035	0.0037	0.7612	-0.941	0.0023	0.0014	0.0039
HARS	1.1599	0.002	0.0009	0.8235	-0.873	0.002	0.0011	0.0044
HAUS6	1.1599	0.002	0.0009	1.425	-0.587	0.0019	0.0007	0.0036
HDAC3	1.6946	0.0109	0.0034	0.8564	-1.252	0.0027	0.0015	0.0064
HDAC5	0.1298	0.0192	0.0176	0.5974	-1.277	0.0135	0.0089	0.0154
HELQ	1.9036	0.0026	0.0007	1.2619	-0.665	0.0021	0.0009	0.0068
HGFAC	0.3075	0.0096	0.0078	0.7946	-1.504	0.0144	0.0083	0.0115
HNF1A	2.2885	0.0128	0.0026	1.7196	-0.816	0.0136	0.0041	0.0101
HNF1B	3.4162	0.0243	0.0023	1.2124	-0.975	0.0051	0.0022	0.0099
HPDL	2.1705	0.0277	0.0061	3.4039	-1.183	0.0564	0.0053	0.0121
HS3ST1	2.6159	0.0112	0.0018	1.6723	-2.125	0.0038	0.0012	0.0048
HSD17B14	2.2076	0.006	0.0013	1.4363	-1.231	0.0057	0.0021	0.0059
HSF2	-0.798	0.001	0.0018	0.8264	-0.637	0.0008	0.0004	0.0023
HUS1	0.8949	0.0083	0.0044	1.1872	-0.849	0.0047	0.0021	0.0052
ICAM1	1.0328	0.0396	0.0193	0.8534	-0.698	0.0153	0.0085	0.0047
IDS	3.1319	0.0025	0.0003	1.1599	-0.664	0.0006	0.0002	0.0023
IFNLR1	1.5207	0.0042	0.0015	0.642	-1.507	0.0026	0.0017	0.0041
IK	1.1	0.0024	0.0011	1.3253	-1.407	0.0028	0.0011	0.0052
INPP5J	1.7276	0.0109	0.0033	1.5931	-1.327	0.0084	0.0028	0.0081
INTS3	-0.579	0.0044	0.0066	0.5865	-1.089	0.0041	0.0027	0.0093
INTS6	-0.44	0.0015	0.002	0.842	-0.764	0.0007	0.0004	0.0024
INTS8	1.2536	0.0024	0.001	0.9875	-0.833	0.0008	0.0004	0.0031
ITGA1	2.1695	0.0026	0.0006	1.5356	-0.83	0.0014	0.0005	0.0025
KBTBD7	0.5441	0.0012	0.0008	0.6146	-1.194	0.0009	0.0006	0.0029
KCNQ1OT1	0.8547	0.0027	0.0015	1.1695	-0.884	0.0017	0.0007	0.0024
KLRG2	1.6285	0.0392	0.0127	1.7526	-0.588	0.02	0.0059	0.0098
KRT7	3.5074	0.0479	0.0042	2.7972	-2.071	0.022	0.0032	0.0107
KRTCAP3	2.4943	0.0205	0.0036	2.3016	-0.635	0.0259	0.0053	0.0174
LIG3	1.3854	0.0027	0.001	0.5865	-1.15	0.0022	0.0015	0.0074
LLGL2	3.6232	0.0568	0.0046	1.9279	-1.036	0.0208	0.0055	0.0127
LRRC75A	0.9307	0.0033	0.0017	0.8584	-0.865	0.0048	0.0027	0.0106
LRRFIP2	-0.177	0.0015	0.0017	0.7461	-0.642	0.0012	0.0007	0.0033
LSM5	0.8074	0.0028	0.0016	1.0293	-0.799	0.0038	0.0019	0.004
MBIP	-0.554	0.0009	0.0014	0.9665	-0.84	0.0009	0.0004	0.0029
MFSD2A	2.9145	0.0249	0.0033	2.0227	-1.053	0.0089	0.0022	0.0052
MGST1	0.5423	0.0045	0.0031	0.824	-1.131	0.0028	0.0016	0.0045
MMGT1	1.3523	0.0012	0.0005	1.1695	-1.237	0.0006	0.0003	0.0012
MPZL2	1.7775	0.0252	0.0073	2.0309	-1.895	0.0125	0.0031	0.0015
MRPS31	1.4893	0.0045	0.0016	1.7374	-1.128	0.0042	0.0013	0.0062
MTFP1	1.1426	0.0124	0.0056	1.1462	-0.738	0.0103	0.0047	0.0088
MTMR10	0.0925	0.0014	0.0013	1.0305	-0.795	0.0015	0.0007	0.0041

MTO1	1.1686	0.0045	0.002	0.6668	-0.735	0.0019	0.0012	0.0064
MTX3	0.447	0.0014	0.001	0.9281	-0.669	0.0011	0.0006	0.0022
MYRF	2.8854	0.0521	0.0071	2.1256	-2.054	0.0229	0.0053	0.0111
MYSM1	-0.406	0.002	0.0026	1.1235	-0.642	0.0012	0.0006	0.0028
NDRG2	1.2007	0.0063	0.0027	1.0309	-0.819	0.0038	0.0019	0.0072
NDUFA2	1.0293	0.0023	0.0011	1.4229	-0.939	0.0032	0.0012	0.007
NFATC3	-0.056	0.0019	0.0019	0.772	-0.587	0.0011	0.0006	0.0033
NFE2	1.5981	0.0238	0.0079	1.9866	-0.696	0.0409	0.0103	0.0108
NKRF	0.8031	0.0024	0.0014	0.8567	-1.709	0.0008	0.0004	0.0007
NOTCH1	2.7768	0.0468	0.0068	1.2183	-1.157	0.0119	0.0051	0.0139
NOXA1	0.8624	0.0103	0.0057	1.2613	-0.716	0.0129	0.0054	0.0077
NR4A1	1.6824	0.0219	0.0068	1.0529	-2.115	0.0143	0.0069	0.0594
NRARP	0.3635	0.0059	0.0046	1.0836	-1.748	0.0037	0.0017	0.0056
NUDT5	-0.077	0.0015	0.0016	0.8405	-0.816	0.002	0.0011	0.0056
NUFIP1	1.632	0.0049	0.0016	1.1172	-0.815	0.0032	0.0015	0.0034
OARD1	0.7414	0.0063	0.0038	0.8171	-0.709	0.0033	0.0019	0.007
ONECUT3	1.6551	0.1313	0.0417	1.1713	-1.092	0.0621	0.0276	0.0043
PALM3	0.8382	0.0122	0.0068	0.7876	-0.799	0.0134	0.0077	0.0103
PAN2	0.7088	0.0037	0.0023	1.4613	-1.044	0.0048	0.0017	0.0072
PANK3	-0.101	0.003	0.0032	0.6765	-0.669	0.0016	0.001	0.0049
PAQR5	1.9978	0.015	0.0038	1.5483	-1.037	0.0054	0.0018	0.0057
PARD6B	3.1494	0.0368	0.0042	2.5689	-1.092	0.0188	0.0032	0.007
PARP6	0.838	0.0049	0.0027	0.701	-0.601	0.0011	0.0007	0.0051
PCSK9	1.4608	0.0354	0.0129	1.3807	-1.666	0.014	0.0054	0.0059
PCYT2	-0.203	0.0066	0.0076	1.0094	-1.49	0.0149	0.0074	0.0264
PDGFB	3.5362	0.0462	0.004	3.1485	-0.869	0.0219	0.0025	0.0092
PERP	2.1605	0.0155	0.0035	2.4989	-1.032	0.0045	0.0008	0.0021
PEX11B	0.9144	0.0062	0.0033	0.8908	-0.912	0.0075	0.0041	0.0079
PGLYRP1	0.9129	0.0089	0.0047	1.0939	-1.076	0.0071	0.0033	0.0063
PIGA	0.6146	0.0007	0.0005	0.9571	-0.929	0.0005	0.0002	0.0018
PIGB	0.1673	0.0013	0.0011	1.0679	-0.911	0.0023	0.0011	0.0036
PKLR	1.6675	0.0181	0.0057	1.9998	-1.577	0.017	0.0042	0.0095
PLEKHH1	0.9697	0.0027	0.0014	0.9122	-0.977	0.0029	0.0016	0.0051
PLLP	1.5964	0.0435	0.0144	1.4654	-1.387	0.0149	0.0054	0.004
PLS1	2.6285	0.0127	0.002	1.5443	-0.759	0.003	0.001	0.0027
PNISR	0.838	0.0016	0.0009	0.5898	-1.168	0.0007	0.0005	0.0023
PNPLA8	0.0299	0.0008	0.0008	0.6327	-0.763	0.0007	0.0004	0.0022
PRKX	0.8142	0.0036	0.002	0.659	-0.725	0.001	0.0006	0.0022
PROSER2	1.433	0.0086	0.0032	0.7181	-1.146	0.0038	0.0023	0.0081
PRSS8	2.2351	0.0158	0.0034	2.3407	-1.258	0.0189	0.0037	0.0067
PTPRR	1.1841	0.0202	0.0089	0.6539	-0.594	0.0018	0.0012	0.0028
PURA	1.7	0.0041	0.0013	0.9101	-1.095	0.0031	0.0017	0.0062
RAD1	1.1335	0.0042	0.0019	1.3766	-0.6	0.0056	0.0022	0.0036
RAP2C	-0.466	0.001	0.0014	0.6097	-0.971	0.0001	######	0.0007
RBM12B2	0.8777	0.0021	0.0011	1.0299	-0.599	0.0007	0.0003	0.0011
REEP6	1.7954	0.0174	0.005	1.3557	-0.992	0.0132	0.0051	0.0059
RELL2	1.4771	0.0127	0.0046	2.1178	-2.12	0.018	0.0041	0.006
RETSAT	-0.376	0.0027	0.0035	1.2283	-1.248	0.0061	0.0026	0.011

RFX5	0.8846	0.0055	0.003	1.598	-0.92	0.0115	0.0038	0.0117
RHBDL1	2.4106	0.0442	0.0083	2.1475	-1.553	0.0488	0.011	0.0155
RIPPLY3	2.5065	0.0155	0.0027	2.1652	-0.746	0.0124	0.0028	0.0085
RNF128	#NUM!	0	0.0005	1.5166	-1.377	0.0004	0.0002	0.0011
RNF141	0.4709	0.0022	0.0016	1.1482	-0.645	0.0018	0.0008	0.0042
RNFT1	0.3021	0.0041	0.0033	0.5874	-0.778	0.0012	0.0008	0.0049
RPL5	0.3008	0.0048	0.0039	0.7752	-0.908	0.0051	0.003	0.0103
RPS27A	0.5749	0.002	0.0014	0.6611	-0.95	0.0027	0.0017	0.0108
S100A6	0.2131	0.0049	0.0042	0.9111	-1.956	0.0105	0.0056	0.0428
SAMD10	0.4652	0.0071	0.0052	0.6728	-1.634	0.0111	0.007	0.0071
SCD1	1.503	0.0164	0.0058	1.0258	-0.74	0.008	0.0039	0.0081
SELP	3.5382	0.002	0.0002	1.0365	-0.603	0.0026	0.0013	0.0061
SEMA4A	0.5776	0.0206	0.0138	1.7697	-1.836	0.0198	0.0058	0.0089
SEMA4G	4.1278	0.0795	0.0046	2.8025	-1.058	0.0329	0.0047	0.0154
SEMA7A	1.0421	0.0223	0.0108	0.7294	-0.94	0.0095	0.0057	0.0077
SERINC1	#DIV/0!	0.0012	0	1.0296	-0.62	0.0005	0.0002	0.0026
SH2D5	2.7801	0.034	0.0049	1.2796	-0.682	0.0175	0.0072	0.0093
SIGIRR	2.1221	0.0208	0.0048	1.158	-0.683	0.0106	0.0047	0.0102
SKIDA1	0.2234	0.0028	0.0024	0.9697	-0.719	0.0037	0.0019	0.0046
SLC25A1	1.9766	0.0168	0.0043	2.3079	-1.196	0.0112	0.0023	0.0209
SLC35A5	0.1337	0.0034	0.0031	0.7608	-1.053	0.0013	0.0008	0.0026
SLC35D1	-0.249	0.0008	0.001	0.7707	-1.122	0.0012	0.0007	0.0049
SLC35E2	0.8191	0.0022	0.0013	1.2963	-0.597	0.0036	0.0015	0.0057
SLC37A1	1.3932	0.0105	0.004	0.5872	-1.646	0.0046	0.0031	0.0079
SLC39A9	-0.471	0.0014	0.0019	1.0896	-0.937	0.0022	0.001	0.0029
SLC5A6	0.4929	0.0072	0.0051	0.8538	-0.922	0.0075	0.0041	0.0099
SLC7A6	2.5312	0.002	0.0003	0.7739	-0.647	0.001	0.0006	0.0055
SMIM6	0.3829	0.007	0.0053	0.7805	-1.769	0.0068	0.004	0.0116
SMPD3	2.4814	0.0476	0.0085	2.1315	-0.772	0.0091	0.0021	0.0043
SMTNL2	2.9096	0.0637	0.0085	2.3738	-0.784	0.0254	0.0049	0.0093
SPRYD7	0.3405	0.0051	0.004	1.2707	-0.731	0.0015	0.0006	0.0028
SRP54B	#NUM!	0	0.0001	0.8782	-0.747	######	######	0.0003
STAT2	1.3843	0.008	0.0031	1.9656	-0.873	0.0061	0.0016	0.0037
SULT2B1	1.2348	0.0062	0.0026	0.6177	-0.901	0.004	0.0026	0.0051
SV2A	2.6935	0.0364	0.0056	1.1722	-0.906	0.0163	0.0072	0.0082
SYCE2	3.1783	0.0371	0.0041	3.004	-0.774	0.0157	0.002	0.0063
TCP1	0.1371	0.0033	0.003	0.736	-1.006	0.003	0.0018	0.0077
TDRKH	0.7134	0.0328	0.02	0.6677	-0.662	0.0218	0.0137	0.0038
THOC1	-0.574	0.0029	0.0043	0.6098	-0.671	0.0011	0.0007	0.003
TJP3	1.8032	0.0214	0.0061	1.7866	-2.001	0.0159	0.0046	0.0057
TLE6	0.3202	0.0038	0.0031	1.1394	-0.692	0.0096	0.0044	0.0071
TM2D3	2.7107	0.0045	0.0007	1.7752	-0.826	0.0051	0.0015	0.0046
TMEM102	1.1804	0.0454	0.02	1.9825	-0.599	0.0558	0.0141	0.0078
TMEM126A	0.6149	0.0017	0.0011	2.7253	-1.354	0.0014	0.0002	0.0034
TMEM151A	2.793	0.0678	0.0098	3.1925	-0.84	0.0691	0.0076	0.0151
TMEM159	0.8428	0.0084	0.0047	0.9223	-1.513	0.0031	0.0017	0.0017
TMEM41A	-0.087	0.0041	0.0044	1.1235	-1.27	0.0017	0.0008	0.0037
TMIGD1	2.3518	0.0041	0.0008	0.9654	-1.762	0.0038	0.0019	0.0056

TMPRSS2	3.4175	0.017	0.0016	1.2479	-1.307	0.0049	0.0021	0.0089
TOP1	0.2933	0.0014	0.0011	0.7929	-0.904	0.0008	0.0005	0.0031
TRIM59	0.0305	0.0028	0.0027	0.6489	-1.006	0.0016	0.001	0.008
TRPC1	1.2865	0.005	0.002	0.9367	-0.87	0.0015	0.0008	0.0016
TSPAN15	3.3488	0.0197	0.0019	1.6598	-1.178	0.0045	0.0014	0.0052
TSR1	0.6354	0.0044	0.0028	1.104	-0.697	0.0031	0.0015	0.0107
TSTD3	3.2072	0.001	0.0001	1.7853	-1.307	0.001	0.0003	0.0016
TUBGCP4	2.3104	0.004	0.0008	1.5135	-0.627	0.0028	0.001	0.0071
TUG1	-0.249	0.0016	0.0019	1.7306	-0.65	0.001	0.0003	0.0054
UBE4A	-0.173	0.0038	0.0043	0.6288	-0.777	0.0018	0.0012	0.0042
UBL4A	3.0753	0.0038	0.0005	4.7367	-1.071	0.0064	0.0002	0.0036
UCP2	0.9535	0.0053	0.0027	0.95	-1.495	0.0048	0.0025	0.0073
UEVLD	-0.422	0.0022	0.003	1.1725	-1.357	0.0027	0.0012	0.0027
UGT1A7C	0.6822	0.0026	0.0016	1.2033	-0.597	0.0033	0.0014	0.0061
UGT2B34	-0.485	0.0008	0.0011	1.8504	-1.164	0.0024	0.0007	0.003
UIMC1	-1.292	0.0005	0.0011	0.6916	-0.634	0.0011	0.0007	0.0023
UNC13A	-0.167	0.0044	0.0049	0.7622	-1.164	0.0123	0.0073	0.0119
UNG	1.62	0.0189	0.0061	1.1222	-0.913	0.0102	0.0047	0.0087
UQCRB	1.8082	0.0014	0.0004	1.5171	-2.068	0.002	0.0007	0.0027
VNN1	-1.138	0.0005	0.001	1.0304	-0.746	0.0014	0.0007	0.0047
WDR47	1.379	0.0098	0.0038	0.8695	-0.638	0.0026	0.0014	0.0049
WDR77	-0.126	0.003	0.0033	0.664	-1.491	0.0041	0.0026	0.0071
WNK4	0.8146	0.0133	0.0076	0.776	-1.273	0.0083	0.0048	0.0092
ZFP113	0.2825	0.0029	0.0024	1.2506	-0.6	0.0037	0.0016	0.0073
ZFP157	0.8976	0.0036	0.0019	0.754	-0.784	0.0019	0.0011	0.0045
ZFP160	1.5955	0.0041	0.0014	0.6148	-0.927	0.0007	0.0005	0.0027
ZFP24	-0.233	0.0029	0.0034	0.9286	-0.885	0.0026	0.0014	0.005
ZFP322A	0.9208	0.0037	0.0019	1.1882	-0.954	0.0019	0.0008	0.0028
ZFP383	0.0314	0.0009	0.0009	0.7306	-0.672	0.0011	0.0007	0.0035
ZFP456	1.5658	0.0034	0.0011	2.0807	-0.733	0.0033	0.0008	0.0021
ZFP551	0.8374	0.0037	0.002	1.1473	-0.784	0.0019	0.0008	0.004
ZFP623	0.7382	0.0046	0.0028	0.6607	-1.331	0.0061	0.0038	0.0059
ZFP715	-0.586	0.0017	0.0026	0.6399	-0.731	0.0013	0.0008	0.0028
ZFP719	0.7674	0.0029	0.0017	1.0672	-1.015	0.002	0.0009	0.0042
ZFP82	2.0004	0.0055	0.0014	1.2607	-1.01	0.0025	0.0011	0.0048
ZFP825	0.9536	0.0021	0.0011	0.8788	-0.684	0.0012	0.0007	0.0019
ZFP970	1.2513	0.0016	0.0007	0.736	-1.079	0.0011	0.0006	0.0016
ZIK1	0.1829	0.0012	0.001	0.7439	-1.334	0.0008	0.0005	0.0019
ZKSCAN1	1.3527	0.0058	0.0023	1.0689	-0.763	0.0041	0.002	0.0058
ZMAT3	0.8108	0.0092	0.0052	0.7196	-0.908	0.003	0.0018	0.0046
ZSCAN29	1.9492	0.004	0.001	2.36	-0.589	0.0056	0.0011	0.0058
ABCC4	1.0305	0.0028	0.0014	-0.071	-1.563	0.0013	0.0014	0.0044
ABCC5	0.8976	0.0036	0.0019	0.2439	-0.921	0.0016	0.0014	0.0076
ACAD9	0.6157	0.0073	0.0048	0.1756	-1.022	0.0033	0.0029	0.0094
ACAN	1.0423	0.007	0.0034	0.4687	-1.29	0.0023	0.0016	0.0053
ACLY	0.6227	0.006	0.0039	0.5766	-0.866	0.0047	0.0031	0.0154
ACOT9	0.838	0.0016	0.0009	0.1351	-0.842	0.0004	0.0004	0.0021
ACVR2B	1.516	0.0073	0.0026	0.4493	-1.367	0.0024	0.0017	0.0033

AGRN	0.8042	0.0062	0.0035	0.2928	-1.308	0.0031	0.0025	0.0099
APPBP2	2.614	0.0021	0.0003	0.3603	-0.697	0.0015	0.0012	0.0052
ARHGAP35	0.895	0.0071	0.0038	0.4421	-0.59	0.0016	0.0011	0.004
ATAD5	2.2166	0.0029	0.0006	0.4512	-0.589	0.0013	0.001	0.0048
ATP6AP2	0.8923	0.0023	0.0013	0.0303	-0.716	0.0005	0.0005	0.0026
ATRX	0.7063	0.0005	0.0003	0.241	-1.019	0.0003	0.0003	0.0013
BRIX1	1.1157	0.0041	0.0019	0.1502	-1.11	0.0012	0.0011	0.005
CAMKK2	1.7963	0.0079	0.0023	0.3854	-0.821	0.0032	0.0025	0.0068
CARD10	0.9665	0.0102	0.0052	0.1109	-1.114	0.0048	0.0045	0.0109
CASP3	0.5927	0.0036	0.0024	0.1631	-0.588	0.0014	0.0013	0.0096
CCL20	0.7903	0.0026	0.0015	0.3781	-0.591	0.0041	0.0031	0.0042
CCNL1	0.5947	0.004	0.0026	-0.084	-1.069	0.0017	0.0018	0.0127
CD2AP	1.321	0.0026	0.001	-0.186	-1.568	0.0009	0.0011	0.0047
CDHR2	1.3014	0.0048	0.0019	0.1919	-0.915	0.0033	0.0029	0.0055
CENPE	0.8781	0.0016	0.0009	-0.61	-0.714	0.0008	0.0012	0.0077
CGGBP1	1.279	0.0022	0.0009	-0.522	-0.629	0.0007	0.001	0.0056
CSE1L	0.7037	0.0034	0.0021	-0.25	-0.9	0.0013	0.0015	0.005
CSF3	0.6399	0.0105	0.0067	-0.364	-0.723	0.0053	0.0068	0.0152
CTSH	1.2689	0.0107	0.0044	0.319	-1.278	0.0044	0.0035	0.0037
CUL4B	0.838	0.0008	0.0005	0.3781	-0.603	0.0004	0.0003	0.002
CYB5B	1.0279	0.0007	0.0003	-0.196	-1.048	0.0008	0.0009	0.0042
DCP2	1.1097	0.0022	0.001	-0.427	-0.587	0.0012	0.0016	0.0053
DCTN4	1.4843	0.003	0.0011	0.2058	-1.169	0.0015	0.0013	0.0053
DENND2D	1.485	0.0123	0.0044	0.3103	-1.377	0.0036	0.0029	0.0046
DIS3L	0.9971	0.0025	0.0013	0.3524	-0.677	0.002	0.0016	0.0055
DOK7	0.666	0.0745	0.047	-0.443	-0.66	0.0152	0.0207	0.008
DUS4L	0.6769	0.0021	0.0013	0.445	-0.952	0.0011	0.0008	0.0018
EIF4G2	0.6149	0.0017	0.0011	-9E-04	-1.059	0.001	0.001	0.0074
EIF5	0.5992	0.005	0.0033	0.0314	-1.432	0.0013	0.0013	0.0124
ELF4	1.4097	0.0015	0.0006	0.0013	-0.766	0.0007	0.0007	0.004
FASTKD5	0.6484	0.0038	0.0024	0.2346	-1.041	0.002	0.0017	0.005
FBXO45	1.1363	0.0016	0.0007	0.2495	-0.602	0.0015	0.0012	0.0033
FKBP11	1.4899	0.0109	0.0039	0.4661	-1.284	0.0063	0.0045	0.0058
FKBP3	2.2672	0.0038	0.0008	0.5085	-1.088	0.0012	0.0008	0.0039
GATA6	1.1209	0.0153	0.0071	0.5509	-1.897	0.0024	0.0016	0.0055
GATB	1.2076	0.003	0.0013	0.0507	-0.702	0.0016	0.0015	0.0043
GCH1	1.2715	0.003	0.0013	0.3524	-0.98	0.0018	0.0014	0.0035
GCNT3	0.7508	0.0016	0.001	0.2379	-0.749	0.0016	0.0013	0.0046
GGT6	0.8723	0.021	0.0115	0.2081	-1.201	0.0199	0.0172	0.0116
GIPC2	0.6354	0.0044	0.0028	-0.021	-1.233	0.0028	0.0028	0.0077
GM14325	0.9486	0.002	0.001	0.4076	-0.682	0.0008	0.0006	0.0021
GMEB2	0.7456	0.0051	0.003	0.1913	-0.893	0.0022	0.0019	0.006
GMNN	1.6163	0.0014	0.0005	0.3254	-1.311	0.0009	0.0007	0.003
GNPAT	1.5831	0.0026	0.0009	0.5013	-1.291	0.0017	0.0012	0.0053
GRASP	0.6844	0.0062	0.0039	-0.052	-1.337	0.0042	0.0043	0.0116
GTF3C2	2.7098	0.0037	0.0006	0.1628	-1.067	0.0026	0.0024	0.0072
HACD1	1.2317	0.0027	0.0011	0.4781	-0.776	0.0014	0.001	0.0061
HAPLN4	1.3654	0.0233	0.009	0.4196	-0.993	0.0225	0.0168	0.0098

HCN3	1.269	0.0263	0.0109	0.4846	-0.885	0.0238	0.017	0.0081
HECTD1	2.0261	0.0009	0.0002	0.2051	-1.038	0.0006	0.0006	0.004
HERPUD1	0.6872	0.006	0.0038	-0.063	-2.684	0.0046	0.0048	0.0084
IFI27L2B	1.617	0.0017	0.0006	-0.077	-1.194	0.0014	0.0015	0.0024
IL1RN	2.0299	0.0033	0.0008	0.1715	-1.238	0.0013	0.0011	0.01
IL22RA1	0.5916	0.0034	0.0023	0.1435	-1.997	0.0024	0.0022	0.0077
KCNK6	0.605	0.0273	0.0179	-0.007	-0.903	0.0172	0.0173	0.0051
ME1	0.6475	0.0013	0.0009	0.45	-2.044	0.0014	0.001	0.0045
MIR17HG	0.7966	0.004	0.0023	-0.969	-2.322	0.0003	0.0006	0.0031
MKKS	0.7508	0.0033	0.0019	0.5108	-0.619	0.001	0.0007	0.0048
MPHOSPH1	0.6832	0.0038	0.0024	0.3045	-0.633	0.0012	0.0009	0.0034
MPHOSPH8	0.8937	0.0035	0.0019	-0.071	-0.693	0.0008	0.0009	0.0044
MST1R	0.8168	0.0509	0.0289	0.351	-0.739	0.0332	0.026	0.009
MTCH2	1.2131	0.0049	0.0021	0.5663	-0.778	0.0023	0.0015	0.0053
MTMR6	1.4446	0.0019	0.0007	0.4962	-0.619	0.0011	0.0008	0.0034
MUC1	0.641	0.0066	0.0042	0.4661	-1.954	0.0056	0.004	0.0157
MVD	0.7934	0.0055	0.0032	0.1765	-1.357	0.0026	0.0023	0.0075
MYO5B	0.9353	0.0365	0.0191	0.0299	-0.596	0.002	0.0019	0.004
NCOR1	1.0299	0.0016	0.0008	-0.022	-0.75	0.0009	0.0009	0.0057
NEURL1B	0.7899	0.0189	0.0109	0.1601	-1.23	0.0063	0.0057	0.0128
OGT	1.0314	0.0012	0.0006	-0.585	-1.112	0.0005	0.0007	0.0039
PARG	0.658	0.002	0.0013	0.0884	-0.599	0.0012	0.0011	0.003
PCDHB21	0.7037	0.0034	0.0021	-0.084	-1.971	0.0031	0.0033	0.0023
PCDHGC5	0.796	0.003	0.0017	-0.475	-1.176	0.0016	0.0022	0.0056
PGGT1B	1.3512	0.0009	0.0003	-0.328	-1.244	0.0008	0.001	0.0037
PHLDB2	2.8362	0.0024	0.0003	0.0131	-1.081	0.0012	0.0011	0.0069
PITPNM2	1.9403	0.0089	0.0023	0.4681	-0.685	0.0043	0.0031	0.0081
PKHD1	1.956	0.0044	0.0011	0.4862	-1.061	0.0017	0.0012	0.0029
PLAC8	0.8547	0.0027	0.0015	0.031	-0.994	0.0009	0.0009	0.0121
PLK4	1.0807	0.0034	0.0016	0.0604	-1.03	0.001	0.001	0.0062
POLI	0.759	0.0031	0.0018	0.3587	-0.859	0.0018	0.0014	0.0041
PTAR1	1.9036	0.0013	0.0003	0.1403	-1.369	0.0008	0.0008	0.0031
PTPN3	1.9036	0.0026	0.0007	0.2355	-1.31	0.0017	0.0014	0.0044
RAP1GAP	1.9378	0.0174	0.0046	0.2728	-0.826	0.0075	0.0062	0.0068
RBL1	0.6789	0.0034	0.0021	-0.199	-0.84	0.0014	0.0016	0.0058
RBM12	1.5734	0.0019	0.0006	0.0775	-0.753	0.0013	0.0012	0.0057
RBM38	2.4402	0.0148	0.0027	0.0588	-1.058	0.0042	0.004	0.011
RBPMS	0.658	0.002	0.0013	-0.118	-1.603	0.0016	0.0018	0.0056
RFWD3	1.7306	0.0015	0.0005	0.3176	-1.244	0.0009	0.0007	0.004
RGL1	0.9114	0.0202	0.0108	-0.275	-0.673	0.0029	0.0035	0.0056
RSAD1	0.5901	0.0065	0.0043	0.1242	-0.797	0.0041	0.0038	0.0119
SCARA3	1.8665	0.0029	0.0008	0.1226	-0.802	0.0016	0.0015	0.006
SDCBP2	0.9376	0.007	0.0036	-0.016	-1.202	0.0043	0.0043	0.0089
SENP1	0.9281	0.0024	0.0013	0.4037	-0.667	0.002	0.0015	0.0065
SH3BP5	0.7311	0.0053	0.0032	-0.094	-1.678	0.002	0.0021	0.0047
SH3TC1	1.5333	0.0079	0.0027	0.5475	-1.203	0.0067	0.0046	0.009
SLC25A36	1.2147	0.0029	0.0013	0.536	-1.013	0.0009	0.0006	0.0025
SLC25A44	1.0993	0.0088	0.0041	0.1447	-1.064	0.0041	0.0037	0.0116

SLC25A46	0.9965	0.0048	0.0024	0.5494	-1	0.0017	0.0012	0.0036
SLC2A6	1.1391	0.0353	0.016	0.3575	-1.531	0.0314	0.0245	0.0082
SLC30A7	0.6443	0.003	0.0019	-0.116	-0.688	0.0012	0.0013	0.0049
SLC39A4	0.594	0.0077	0.0051	0.5587	-1.487	0.0096	0.0065	0.0148
SLC44A3	1.9856	0.0239	0.006	0.1559	-0.734	0.004	0.0036	0.0056
SLC6A9	0.9806	0.0065	0.0033	0.5093	-1.397	0.0039	0.0027	0.0055
SORD	1.0558	0.0069	0.0033	0.5838	-2.057	0.0046	0.0031	0.0051
SPINT2	1.4127	0.0092	0.0035	0.37	-2.127	0.0023	0.0018	0.0029
STARD10	0.7	0.0041	0.0025	-0.074	-1.31	0.002	0.0021	0.0082
STT3A	0.8086	0.0042	0.0024	0.1496	-0.692	0.0007	0.0006	0.0035
STYX	1.1583	0.0014	0.0006	-0.4	-1.258	0.0006	0.0008	0.0026
TBC1D2	1.3433	0.0089	0.0035	-0.206	-1.677	0.0026	0.003	0.0068
TBC1D2B	2.2562	0.0076	0.0016	0.5763	-0.694	0.0017	0.0012	0.004
TM7SF2	0.6533	0.0113	0.0072	0.0305	-1.012	0.0051	0.005	0.0113
TMEM263	1.9581	0.0022	0.0006	-0.809	-0.68	0.0004	0.0008	0.0024
TOM1L1	0.9185	0.0043	0.0023	0.0222	-0.759	0.0014	0.0014	0.0053
TRIM56	0.6553	0.0053	0.0034	-0.429	-1.3	0.0028	0.0038	0.0108
TSPAN8	1.2837	0.0018	0.0007	0.2043	-1.233	0.0006	0.0005	0.0062
TUSC1	0.7105	0.0009	0.0006	#DIV/0!	-2.616	0.001	0	0.0021
UFSP2	0.5874	0.0029	0.0019	0.2591	-0.788	0.0013	0.0011	0.0053
UPF2	0.7674	0.0029	0.0017	0.4353	-0.698	0.0011	0.0008	0.0042
USP38	0.9379	0.0061	0.0032	0.453	-1.08	0.0014	0.001	0.0035
UVSSA	0.7923	0.0045	0.0026	-0.054	-0.654	0.0038	0.0039	0.0073
WDR33	0.7928	0.0023	0.0013	-0.059	-1.379	0.0009	0.0009	0.0039
XYLB	1.8665	0.0029	0.0008	0.4639	-0.841	0.0016	0.0011	0.0043
ZBTB39	0.8856	0.0071	0.0039	0.1131	-1.61	0.0024	0.0022	0.0045
ZDHHC20	1.3021	0.0017	0.0007	0.0417	-0.972	0.0008	0.0008	0.0033
ZFP106	1.6163	0.0014	0.0005	-0.196	-0.878	0.0011	0.0012	0.0065
ZFP317	1.6148	0.0024	0.0008	0.1674	-0.825	0.0004	0.0004	0.002
ZFP369	0.8943	0.0012	0.0006	0.0314	-0.665	0.0006	0.0006	0.0023
ZFP386	0.6337	0.0046	0.003	-0.35	-0.945	0.0012	0.0015	0.0019
ZFP445	1.6799	0.0053	0.0017	0.3851	-0.888	0.0016	0.0012	0.0037
ZFP516	1.0306	0.018	0.0088	-0.166	-1.737	0.0031	0.0035	0.0059
ZFP568	0.6981	0.0016	0.001	-0.225	-0.813	0.001	0.0012	0.0061
ZFP58	0.6166	0.0023	0.0015	0.1888	-1.312	0.001	0.0009	0.0022
ZFP654	1.279	0.0022	0.0009	0.4133	-0.634	0.0007	0.0005	0.0025
ZFP655	0.7	0.002	0.0013	0.5161	-0.97	0.0013	0.0009	0.0085
ZFP68	0.8952	0.003	0.0016	0.4903	-1.163	0.0019	0.0014	0.0039
ZFP869	0.6897	0.0087	0.0054	0.3107	-1.172	0.0025	0.002	0.0069
ZFP91	2.2226	0.0037	0.0008	-0.088	-0.841	0.0008	0.0009	0.004
ZFP952	0.9054	0.0026	0.0014	0.4456	-0.92	0.002	0.0015	0.0025
ZMAT2	1.4903	0.0038	0.0014	-0.272	-2.197	0.0018	0.0022	0.0023

GB_K36_WT	Pvalue	logFC_KO/WT	KO_1	KO_2	KO_3	WT_1	WT_2	WT_3
0.0091	0.00653	-1.525	25	20	22.5	52	88	70
0.0095	0.00864	-5.668	0	0	0	11	9	10
0.0054	0.00028	-0.997	118	118	118	262	243	253
0.0246	9.53E-11	-0.715	1063	1037	1050	1860	1840	1850
0.009	2.22E-06	-0.845	321	276	299	556	594	575
0.0065	7.04E-06	-0.655	660	500	580	945	1009	977
0.0108	2.00E-69	-1.844	816	842	829	3210	3184	3197
0.018	1.29E-13	-0.801	984	909	947	1658	1889	1774
0.0037	4.36E-15	-11.45	0	0	0	547	555	551
0.013	2.05E-11	-0.742	1120	1119	1120	1812	2228	2020
0.0097	3.27E-10	-0.928	475	521	498	986	1056	1021
0.0155	0.00518	-0.597	251	232	242	411	371	391
0.008	2.32E-07	-0.672	668	668	668	1107	1183	1145
0.0192	2.92E-05	-0.887	228	200	214	439	408	424
0.0163	0.02729	-0.949	41	63	52	113	104	109
0.0024	1.91E-54	-2.443	301	226	264	1616	1440	1528
0.0071	3.20E-55	-1.316	1560	1394	1477	3831	4059	3945
0.0192	2.32E-73	-1.08	7085	6125	6605	####	####	####
0.0251	4.25E-09	-0.796	880	636	758	1377	1434	1406
0.0136	1.03E-24	-1.331	612	689	651	1747	1778	1763
0.0053	9.92E-20	-1.074	1009	1173	1091	2396	2560	2478
0.0299	1.79E-11	-0.913	572	491	532	1024	1124	1074
0.0155	2.96E-13	-6.094	3	1	2	158	133	146
0.0207	2.71E-05	-1.194	112	87	99.5	227	261	244
0.0082	1.15E-23	-1.482	355	367	361	1047	1123	1085
0.0055	8.72E-15	-1.113	505	580	543	1200	1333	1267
0.0135	0	-4.081	503	453	478	8491	8872	8682
0.0114	#######	-1.959	4703	4499	4601	####	####	####
0.0105	2.97E-15	-0.699	1690	1550	1620	2728	2919	2824
0.0036	1.78E-05	-0.738	427	333	380	688	666	677
0.0118	8.92E-49	-1.192	2666	2807	2737	6435	7040	6738
0.0148	0.02393	-1.366	18	23	20.5	44	71	57.5
0.0184	1.13E-21	-7.37	0	4	2	362	349	356
0.0291	0	-3.698	7591	6651	7121	####	####	####
0.0173	1.46E-21	-1.09	767	729	748	1675	1744	1710
0.0087	3.57E-06	-1.946	48	24	36	142	153	148
0.0065	6.66E-09	-0.855	465	477	471	872	963	918
0.0064	2.81E-33	-0.985	2042	1833	1938	3819	4429	4124
0.0148	4.20E-17	-3.715	13	14	13.5	187	194	191
0.0111	0.01693	-3.369	1	2	1.5	11	23	17
0.0069	3.81E-09	-0.592	1288	1232	1260	1915	2173	2044
0.0119	4.72E-09	-0.592	4037	3140	3589	5201	5602	5402
0.0047	1.74E-10	-9.574	0	0	0	150	151	151

0.0324	1.63E-42	-5.445	13	7	10	441	493	467
0.0093	2.15E-11	-2.091	50	72	61	272	290	281
0.0104	0.00036	-0.601	413	360	387	569	692	631
0.0055	3.73E-44	-2.347	195	165	180	968	994	981
0.0161	4.50E-44	-0.912	4541	4136	4339	8168	9391	8780
0.0125	0.00209	-6.168	0	0	0	7	22	14.5
0.0101	2.50E-37	-2.063	227	213	220	970	1004	987
0.0093	4.77E-38	-1.218	1192	1096	1144	2662	3062	2862
0.0132	0.00017	-0.722	291	248	270	450	504	477
0.008	9.24E-17	-0.815	1539	1258	1399	2465	2812	2639
0.005	2.88E-16	-1.553	199	224	212	656	681	669
0.0075	1.15E-15	-2.135	113	65	89	390	444	417
0.0042	1.18E-14	-1.202	368	353	361	895	885	890
0.0064	2.09E-14	-1.785	215	142	179	748	555	652
0.0453	9.85E-11	-1.024	494	365	430	859	1012	936
0.0161	0.00209	-5.424	0	1	0.5	15	32	23.5
0.0068	3.16E-36	-2.235	319	190	255	1195	1362	1279
0.0089	7.70E-11	-1.215	234	241	238	569	618	594
0.0456	6.85E-30	-1.021	1690	1412	1551	3170	3582	3376
0.0183	2.68E-26	-6.19	4	4	4	315	311	313
0.0254	1.44E-30	-2.904	60	57	58.5	452	489	471
0.0152	9.53E-24	-1.426	426	462	444	1269	1298	1284
0.005	4.04E-61	-3.626	68	53	60.5	728	877	803
0.0201	2.78E-45	-1.169	1760	1669	1715	3878	4414	4146
0.0177	4.24E-60	-1.387	1462	1297	1380	3713	4030	3872
0.0214	1.43E-29	-3.991	14	29	21.5	369	370	370
0.0318	2.27E-08	-0.858	461	391	426	821	833	827
0.0097	1.73E-17	-1.166	582	486	534	1147	1429	1288
0.0055	6.35E-37	-4.201	23	18	20.5	394	414	404
0.0366	#######	-2.149	2524	2277	2401	####	####	####
0.0336	#######	-3.396	375	329	352	3640	4331	3986
0.0108	1.14E-23	-8.599	0	3	1.5	607	643	625
0.011	1.04E-26	-1.058	1193	1088	1141	2339	2772	2556
0.0019	0.00205	-0.61	312	396	354	523	648	586
0.0087	0.00385	-3.574	4	0	2	25	25	25
0.0076	3.67E-20	-1.64	222	232	227	763	758	761
0.0073	2.13E-23	-1.373	445	464	455	1192	1345	1269
0.0071	5.41E-17	-1.022	667	650	659	1358	1520	1439
0.01	1.66E-50	-7.614	5	4	4.5	908	985	947
0.0344	6.87E-60	-9.111	4	3	3.5	1997	2158	2078
0.0125	0.03287	-5.14	0	0	0	6	8	7
0.005	1.18E-15	-2.992	37	20	28.5	206	280	243
0.02	5.73E-40	-1.296	1047	977	1012	2623	2713	2668
0.0172	2.39E-56	-1.403	1276	1179	1228	3275	3705	3490
0.0174	2.17E-11	-9.954	0	0	0	190	202	196
######	0.00032	-3.326	1	7	4	52	35	43.5
0.0032	1.74E-12	-2.65	28	33	30.5	219	192	206
0.0059	3.55E-23	-3.857	28	10	19	282	304	293

0.0071	0.0437	-5.018	0	0	0	4	9	6.5
0.0092	0.00028	-0.653	469	329	399	675	663	669
0.0147	2.41E-19	-0.914	1128	1112	1120	2130	2414	2272
0.0047	2.44E-84	-3.847	65	72	68.5	1015	1107	1061
0.0065	3.32E-10	-1.852	80	62	71	242	309	276
0.0614	#######	-8.797	9	5	7	3268	3412	3340
0.0043	6.55E-16	-1.712	144	166	155	513	583	548
0.0075	9.53E-07	-0.843	408	512	460	893	888	891
0.0081	1.85E-24	-0.856	1865	1747	1806	3405	3617	3511
0.0054	1.05E-11	-1.166	369	467	418	1027	996	1012
0.0152	3.10E-19	-0.748	2147	2096	2122	3746	3914	3830
0.0373	2.23E-22	-0.934	1422	1186	1304	2553	2789	2671
0.0108	0.0003	-0.642	338	362	350	551	627	589
0.0327	6.25E-07	-6.338	1	11	6	536	540	538
0.0177	1.36E-27	-7.466	1	4	2.5	443	509	476
0.0195	6.98E-06	-0.643	698	550	624	936	1157	1047
0.0275	0.02943	-1.184	57	17	37	75	103	89
0.0209	#######	-4.847	51	62	56.5	1695	1803	1749
0.0138	1.88E-65	-3.986	44	50	47	724	883	804
0.0036	0.00635	-0.682	251	155	203	358	334	346
0.0094	2.06E-11	-1.157	324	343	334	698	910	804
0.0076	9.98E-18	-2	107	98	103	420	461	441
0.0036	8.87E-11	-0.943	577	453	515	964	1161	1063
0.0116	0.00016	-0.983	141	201	171	319	416	368
0.0139	3.84E-23	-0.903	2000	2124	2062	3901	4413	4157
0.0202	9.95E-41	-5.277	9	12	10.5	418	458	438
0.0197	1.12E-25	-0.835	2180	1961	2071	3755	4181	3968
0.0041	0.00034	-0.601	444	356	400	599	703	651
0.0054	4.22E-18	-1.624	223	178	201	646	677	662
0.0044	#######	-6.197	48	29	38.5	2723	3351	3037
0.0065	0.00117	-1.361	56	33	44.5	112	132	122
0.0044	3.37E-08	-2.324	21	48	34.5	172	205	189
0.0147	9.56E-18	-2.511	63	44	53.5	309	344	327
0.045	#######	-4.196	1411	1042	1227	####	####	####
0.027	3.79E-06	-1.403	74	101	87.5	236	265	251
0.0165	1.42E-15	-0.828	1229	1236	1233	2154	2564	2359
0.0261	9.38E-08	-1.027	258	223	241	525	525	525
0.0193	9.56E-12	-1.398	203	250	227	674	611	643
0.0051	4.08E-08	-0.714	653	651	652	1094	1208	1151
0.0069	0.00794	-0.705	138	136	137	235	245	240
0.0052	1.50E-05	-0.834	267	248	258	451	537	494
0.0107	3.44E-19	-4.872	11	2	6.5	198	207	203
0.01	1.19E-18	-0.866	1228	1202	1215	2282	2482	2382
0.0029	4.52E-12	-1.111	345	327	336	760	799	780
0.0055	3.95E-17	-12.22	0	0	0	905	985	945
0.0135	1.97E-07	-0.778	463	429	446	784	859	822
0.0147	0.00292	-1.06	73	58	65.5	136	157	147
0.0071	1.75E-18	-1.299	593	412	503	1258	1384	1321

0.0107	9.63E-12	-1.259	281	217	249	627	648	638
0.0035	0.00087	-0.671	304	230	267	421	490	456
0.0459	0	-6.354	63	46	54.5	4441	5140	4791
0.0043	1.62E-10	-1.022	394	368	381	738	929	834
0.0127	2.19E-14	-1.076	497	540	519	1129	1226	1178
0.0135	1.14E-05	-0.599	638	553	596	940	995	968
0.005	1.25E-38	-1.311	1043	881	962	2402	2722	2562
0.0175	2.28E-05	-7.249	0	0	0	31	29	30
0.0024	4.74E-08	-1.31	154	120	137	333	396	365
0.0309	5.29E-26	-1.83	213	202	208	747	840	794
0.0126	0.02355	-0.846	96	53	74.5	137	148	143
0.2574	5.63E-22	-1.667	235	267	251	822	897	860
0.0188	1.21E-06	-1.206	187	119	153	377	375	376
0.0098	1.48E-09	-0.683	1024	866	945	1600	1651	1626
0.0061	0.00107	-0.586	339	312	326	497	553	525
0.0114	4.74E-09	-0.782	606	544	575	1027	1095	1061
0.0091	2.72E-09	-9.088	0	0	0	82	135	109
0.0179	3.06E-66	-4.898	21	24	22.5	685	759	722
0.0148	1.09E-14	-1.284	344	276	310	741	880	811
0.0078	3.75E-44	-1.299	1289	1100	1195	2948	3363	3156
0.0117	1.05E-51	-3.961	61	28	44.5	756	717	737
0.0149	3.11E-18	-2.282	118	64	91	446	497	472
0.0077	0.00217	-0.713	191	172	182	291	349	320
0.0187	#######	-10.15	11	4	7.5	8839	9412	9126
0.0742	9.29E-54	-1.301	1722	1495	1609	4183	4312	4248
0.0167	3.47E-09	-1.12	263	222	243	513	620	567
0.0043	2.96E-21	-1.376	408	425	417	1166	1157	1162
0.0149	0.00353	-0.587	331	241	286	449	469	459
0.0132	0.00583	-5.8	0	0	0	11	11	11
0.0033	7.90E-05	-1.022	170	116	143	314	305	310
0.0069	1.20E-06	-1.247	118	120	119	279	330	305
0.0284	3.12E-20	-13.34	0	0	0	1988	2112	2050
0.0101	5.69E-12	-1.522	165	129	147	437	467	452
0.0106	3.18E-20	-3.636	22	14	18	217	263	240
0.0045	#######	-3.647	134	138	136	1764	1901	1833
0.0051	1.48E-17	-1.115	657	589	623	1276	1631	1454
0.0037	5.62E-06	-0.734	481	370	426	710	807	759
0.0036	8.01E-07	-0.994	229	211	220	429	514	472
0.0178	8.52E-24	-2.342	87	96	91.5	480	519	500
0.0161	8.51E-05	-5.136	2	0	1	33	42	37.5
0.0043	1.05E-39	-1.875	352	293	323	1222	1314	1268
0.0132	2.00E-30	-1.54	529	408	469	1400	1518	1459
0.0055	7.45E-05	-0.855	206	199	203	396	390	393
0.0015	4.62E-12	-1.2	279	286	283	682	714	698
0.0017	0.00018	-1.238	69	112	90.5	240	222	231
0.0118	5.04E-11	-1.732	107	85	96	297	389	343
0.0259	2.26E-07	-1.813	65	42	53.5	212	188	200
0.0262	2.27E-41	-1.589	575	513	544	1685	1829	1757

0.0221	8.57E-07	-0.914	270	280	275	540	575	558
0.0456	0.01246	-1.245	26	38	32	82	82	82
0.0142	1.13E-16	-1.807	140	121	131	485	494	490
0.003	1.73E-50	-1.826	466	449	458	1664	1823	1744
0.0066	4.15E-21	-1.535	372	270	321	923	1070	997
0.0084	0.00117	-0.626	304	305	305	527	481	504
0.0194	4.40E-28	-1.341	713	777	745	2040	2021	2031
0.0209	4.98E-09	-0.822	641	524	583	1122	1080	1101
0.1662	1.45E-24	-0.619	####	####	####	####	####	####
0.022	0.00121	-0.823	147	143	145	249	304	277
0.0136	#######	-1.471	4763	3880	4322	####	####	####
0.0093	0.02325	-0.724	149	85	117	177	236	207
0.0319	1.64E-91	-7.952	10	5	7.5	1929	2056	1993
0.032	3.32E-87	-3.355	115	128	122	1227	1454	1341
0.0147	0.01579	-1.071	38	50	44	106	93	99.5
0.004	7.41E-14	-0.659	2001	1968	1985	3116	3635	3376
0.0149	3.14E-06	-2.262	20	25	22.5	135	96	116
0.0163	2.35E-06	-0.657	835	608	722	1181	1250	1216
0.0076	0.00373	-1.838	11	20	15.5	50	71	60.5
0.048	0.01346	-2.981	20	15	17.5	37	268	153
0.0054	8.84E-06	-0.874	263	288	276	477	615	546
0.0108	1.57E-54	-2.039	447	355	401	1613	1926	1770
0.0086	7.77E-05	-0.794	245	275	260	483	488	486
0.0247	8.57E-56	-3.895	43	40	41.5	613	715	664
0.0055	3.09E-06	-0.619	703	592	648	1000	1134	1067
0.0188	8.36E-06	-0.832	304	252	278	519	541	530
0.0087	1.13E-21	-1.212	718	793	756	1699	2085	1892
0.0394	3.57E-19	-3.854	26	7	16.5	258	247	253
0.0074	1.45E-81	-4.64	45	26	35.5	933	960	947
0.016	0.00185	-1.346	37	41	39	99	115	107
0.0047	0.00017	-1.086	109	127	118	301	236	269
0.0006	0.00405	-1.567	26	20	23	82	63	72.5
0.0067	0.00154	-0.991	83	108	95.5	203	207	205
0.0094	1.71E-10	-3.158	15	12	13.5	116	143	130
0.0153	0.01562	-0.703	120	112	116	179	228	204
0.0108	2.67E-36	-2.478	127	114	121	662	782	722
0.0154	5.61E-06	-0.836	6071	7134	6603	####	####	####
0.0061	#######	-4.052	100	77	88.5	1473	1679	1576
0.0047	6.44E-81	-2.759	221	244	233	1687	1698	1693
0.0227	2.02E-38	-4.902	18	8	13	402	429	416
0.0114	2.70E-06	-1.285	109	94	102	262	268	265
0.0082	1.85E-05	-0.81	290	253	272	509	511	510
0.0118	0.00011	-1.971	22	20	21	82	95	88.5
0.0086	0.00027	-0.755	266	217	242	387	489	438
0.0271	6.24E-15	-4.618	5	6	5.5	135	156	146
0.005	0.00175	-1.219	50	52	51	129	126	128
0.009	4.52E-11	-1.384	175	159	167	451	485	468
0.019	1.59E-33	-7.555	1	5	3	623	588	606

0.022	4.93E-77	-1.916	680	623	652	2503	2780	2642
0.0059	2.88E-25	-1.044	1272	1298	1285	2610	3105	2858
0.0161	2.83E-24	-1.406	713	710	712	2211	1824	2018
0.003	0.0011	-1.692	23	25	24	93	73	83
0.0117	7.68E-63	-4.72	31	19	25	640	774	707
0.0174	1.37E-10	-0.648	1655	1795	1725	2742	3090	2916
0.004	0.00586	-0.661	180	186	183	323	298	311
0.0109	1.05E-22	-1.118	818	695	757	1718	1802	1760
0.0084	2.87E-35	-1.426	760	641	701	2022	2008	2015
0.0071	9.60E-13	-0.987	592	662	627	1228	1457	1343
0.0075	0.00041	-1.111	79	113	96	205	245	225
0.0207	#######	-3.681	188	129	159	2124	2223	2174
0.007	5.43E-08	-0.802	473	481	477	850	941	896
0.0092	#######	-6.604	18	10	14	1385	1537	1461
0.0068	2.20E-92	-8.208	8	6	7	2113	2332	2223
0.0036	5.06E-06	-0.8	340	294	317	555	630	593
0.0266	1.70E-11	-1.51	146	134	140	427	428	428
0.0163	0.00047	-0.698	279	230	255	412	474	443
0.0114	2.25E-12	-1.595	145	159	152	529	456	493
0.0078	3.02E-67	-3.022	127	119	123	1057	1087	1072
0.0077	0.00675	-0.72	138	138	138	222	268	245
0.0199	3.19E-20	-0.852	1729	1727	1728	3346	3354	3350
0.0222	9.06E-31	-1.437	629	503	566	1595	1688	1642
0.0111	2.26E-05	-0.939	206	171	189	348	429	389
0.0078	6.64E-05	-0.945	162	154	158	324	329	327
0.0051	3.83E-07	-1.682	72	58	65	251	193	222
0.0092	9.31E-64	-2.084	456	377	417	1769	2021	1895
0.0055	4.01E-05	-1.138	109	108	109	265	247	256
0.0055	0.00293	-1.433	38	25	31.5	96	85	90.5
0.0035	0.00031	-2.775	2	15	8.5	74	53	63.5
0.0069	0.00348	-1.238	40	47	43.5	98	124	111
0.0148	0.00047	-0.747	353	221	287	467	562	515
0.0046	2.02E-08	-1.828	68	52	60	244	210	227
0.0085	7.80E-06	-1.278	91	128	110	273	302	288
0.0096	0.00131	-1.845	22	14	18	67	71	69
0.0031	8.04E-11	-3.279	25	6	15.5	156	162	159
0.0034	0.00393	-0.81	142	126	134	281	220	251
0.0047	2.10E-09	-2.202	49	30	39.5	187	201	194
0.0098	2.33E-10	-0.926	669	480	575	1117	1216	1167
0.0086	8.45E-10	-0.957	418	355	387	784	825	805
0.0088	0.00037	-0.749	222	220	221	394	404	399
0.0129	#######	-2.232	865	878	872	3931	4905	4418
0.0144	3.51E-41	-1.117	2051	1790	1921	4108	4848	4478
0.0191	9.88E-10	-0.624	1505	1213	1359	2128	2362	2245
0.013	6.20E-98	-1.58	2461	2086	2274	6769	7833	7301
0.028	1.67E-67	-0.971	8255	7606	7931	####	####	####
0.0038	1.17E-07	-0.841	599	646	623	1311	1078	1195
0.0084	5.74E-06	-1.309	153	86	120	269	364	317

0.0246	1.17E-73	-1.704	1058	916	987	3200	3712	3456
0.0084	5.47E-11	-0.843	837	693	765	1298	1653	1476
0.006	2.16E-18	-0.667	3281	3012	3147	4907	5846	5377
0.0072	2.94E-18	-1.325	353	334	344	887	963	925
0.0042	5.09E-07	-0.653	860	699	780	1339	1282	1311
0.0026	8.86E-56	-1.704	780	667	724	2301	2768	2535
0.0107	1.72E-20	-1.135	656	639	648	1515	1539	1527
0.0121	0.00014	-0.732	381	262	322	547	594	571
0.0236	2.13E-15	-1.608	167	170	169	511	596	554
0.0144	1.34E-43	-0.87	6228	5323	5776	####	####	####
0.0063	0.03271	-4.474	0	10	5	101	150	126
0.0267	4.43E-26	-1.229	705	694	700	1639	1893	1766
0.0138	#######	-1.984	3763	3331	3547	####	####	####
0.0103	6.37E-09	-4.315	50	14	32	624	716	670
0.0126	6.02E-19	-0.744	2331	2171	2251	3694	4421	4058
0.0086	1.45E-15	-0.845	1357	1341	1349	2681	2518	2600
0.0093	4.56E-81	-1.64	1544	1597	1571	4962	5582	5272
0.0251	5.41E-07	-1.855	42	43	42.5	157	174	166
0.009	2.60E-20	-3.494	43	13	28	353	313	333
0.003	2.63E-06	-0.755	637	450	544	1007	948	978
0.0087	1.92E-56	-1.067	4332	3780	4056	8466	9797	9132
0.0079	7.98E-86	-2.066	651	560	606	2545	2902	2724
0.0119	5.80E-21	-0.917	1323	1349	1336	2573	2859	2716
0.012	5.55E-32	-3.495	39	31	35	362	488	425
0.0088	6.00E-06	-0.689	492	417	455	761	810	786
0.0126	5.81E-06	-7.56	0	0	0	43	31	37
0.0034	0.04296	-0.77	65	62	63.5	109	124	117
0.0155	1.20E-31	-0.661	####	9140	9573	####	####	####
0.0335	#######	-1.85	1699	1717	1708	6627	6597	6612
0.0067	1.28E-08	-0.685	824	764	794	1259	1488	1374
0.0103	2.29E-14	-1.691	185	298	242	866	823	845
0.0051	1.61E-11	-0.957	485	493	489	975	1068	1022
0.0142	5.05E-07	-0.756	596	450	523	928	960	944
0.0083	2.31E-06	-0.695	671	817	744	1294	1304	1299
0.0204	1.34E-06	-1.123	167	143	155	329	397	363
0.007	4.17E-12	-1.082	403	345	374	845	851	848
0.0069	2.01E-05	-1.883	24	44	34	137	135	136
0.0077	#######	-7.894	9	10	9.5	2344	2510	2427
0.0267	7.22E-09	-8.884	0	0	0	100	86	93
0.0182	4.07E-27	-2.13	177	129	153	719	712	716
0.0034	2.66E-07	-1.996	34	41	37.5	164	158	161
0.0112	3.11E-07	-0.613	938	782	860	1304	1520	1412
0.0075	0.00012	-0.589	614	752	683	1094	1123	1109
0.013	3.96E-09	-0.638	1031	977	1004	1586	1774	1680
0.0294	5.99E-12	-1.363	305	198	252	627	757	692
0.0152	3.09E-15	-0.619	2434	2237	2336	3669	4041	3855
0.0105	6.11E-14	-1.429	231	289	260	687	829	758
0.0195	3.30E-05	-0.871	211	210	211	401	427	414

0.0149	0.00024	-2.647	12	5	8.5	44	70	57
0.0082	4.60E-14	-0.623	3369	2919	3144	4655	5759	5207
0.0538	3.01E-31	-1.348	719	604	662	1699	1916	1808
0.0055	6.12E-30	-2.835	59	82	70.5	508	580	544
0.0236	4.93E-40	-0.91	4089	4024	4057	7795	8613	8204
0.0308	#######	-2.922	345	312	329	2451	2906	2679
0.0096	0.00014	-5.008	0	2	1	33	37	35
0.0187	#######	-1.604	2811	2489	2650	8629	8637	8633
0.0154	5.50E-10	-3.074	11	19	15	113	162	138
0.0074	3.22E-06	-0.681	561	490	526	915	889	902
0.0053	6.66E-07	-0.684	609	625	617	981	1157	1069
0.0071	2.71E-09	-1.052	285	273	279	602	641	622
0.015	1.85E-05	-7.3	0	0	0	34	28	31
0.0091	2.12E-13	-0.693	1674	1730	1702	2837	3087	2962
0.0053	3.86E-05	-0.598	863	616	740	1078	1320	1199
0.061	#######	-2.718	506	457	482	3298	3501	3400
0.0193	2.56E-47	-1.822	450	397	424	1555	1658	1607
0.0061	1.44E-16	-12.03	0	0	0	697	962	830
0.0096	1.98E-27	-0.95	3226	2718	2972	5421	6933	6177
0.03	2.24E-39	-1.3	1012	893	953	2429	2605	2517
0.0084	1.28E-14	-1.035	644	727	686	1389	1646	1518
0.0046	2.05E-05	-0.653	488	418	453	744	783	764
0.0088	0.0437	-5.018	0	0	0	4	9	6.5
0.0127	0.04701	-2.797	3	1	2	20	9	14.5
0.0087	6.39E-18	-1.322	380	422	401	990	1175	1083
0.0147	8.92E-76	-1.406	2592	2138	2365	6444	6987	6716
0.013	8.57E-08	-0.856	383	373	378	700	772	736
0.0061	1.03E-29	-3.705	39	20	29.5	338	488	413
0.0242	5.74E-45	-0.767	####	9922	####	####	####	####
0.0126	8.40E-34	-1.228	965	889	927	2265	2396	2331
0.0075	5.00E-05	-0.931	227	158	193	393	390	392
0.0081	1.69E-75	-2.702	279	199	239	1569	1760	1665
0.011	9.99E-13	-1.666	138	111	125	380	469	425
0.0121	1.06E-46	-1.682	552	529	541	1808	1918	1863
0.0104	4.59E-26	-1.295	711	566	639	1571	1789	1680
0.0096	2.17E-08	-0.687	793	703	748	1196	1392	1294
0.0228	1.67E-07	-0.717	694	550	622	1072	1116	1094
0.0171	4.19E-74	-1.633	1157	1014	1086	3496	3725	3611
0.0094	9.87E-13	-0.756	1126	1134	1130	2017	2086	2052
0.0089	4.28E-94	-1.597	2018	1715	1867	5679	6447	6063
0.0207	0.00085	-0.663	284	234	259	426	453	440
0.0104	1.29E-97	-2.031	815	799	807	3284	3821	3553
0.0204	1.67E-34	-1.931	281	255	268	1127	1062	1095
0.0103	4.03E-18	-0.847	1462	1283	1373	2408	2903	2656
0.0151	1.13E-15	-1.544	193	216	205	598	689	644
0.0208	8.65E-05	-0.687	350	361	356	558	677	618
0.0051	2.23E-11	-1.073	431	353	392	911	851	881
0.0242	1.24E-29	-1.153	1040	896	968	2175	2446	2311

0.0072	2.80E-45	-2.033	301	289	295	1287	1304	1296
0.0238	0.04099	-2.283	11	0	5.5	34	21	27.5
0.0079	6.52E-13	-0.829	882	764	823	1461	1679	1570
0.0414	1.55E-78	-2.29	677	469	573	2859	3130	2995
0.0093	1.89E-10	-3.816	35	79	57	824	933	879
0.0144	1.55E-17	-1.506	254	218	236	715	721	718
0.0213	5.02E-14	-0.999	559	515	537	1123	1181	1152
0.0128	6.04E-48	-1.651	703	632	668	2281	2209	2245
0.0204	1.67E-51	-1.281	1595	1522	1559	3992	4142	4067
0.0057	8.23E-59	-1.333	1706	1672	1689	4321	4840	4581
0.0063	0.0438	-1.191	21	23	22	55	53	54
0.0218	0.00018	-0.763	238	225	232	396	449	423
0.0065	0.00053	-0.84	173	165	169	277	376	327
0.0228	1.09E-08	-1.29	187	140	164	433	421	427
0.0039	6.69E-07	-0.948	300	242	271	574	543	559
0.0089	7.78E-09	-0.833	502	464	483	854	997	926
0.0265	5.56E-06	-1.029	345	195	270	510	667	589
0.0147	#######	-2.119	1364	1567	1466	6586	7155	6871
0.0129	0.01629	-5.445	0	0	0	11	6	8.5
0.0092	2.80E-06	-0.609	754	813	784	1202	1376	1289
0.0068	1.59E-07	-0.761	847	621	734	1155	1516	1336
0.0074	1.15E-06	-0.741	529	444	487	890	849	870
0.0116	9.28E-07	-1.323	101	129	115	308	313	311
0.0102	3.26E-29	-0.935	1848	1677	1763	3408	3835	3622
0.0078	1.26E-10	-1.258	219	222	221	580	552	566
0.0138	1.99E-07	-0.988	248	252	250	506	562	534
0.0065	8.07E-15	-1.053	534	472	503	1095	1144	1120
0.0119	2.26E-33	-0.902	7070	5851	6461	####	####	####
0.0036	1.70E-10	-1.189	241	233	237	544	619	582
0.0036	9.41E-05	-1.674	56	28	42	113	174	144
0.0037	3.35E-08	-1.39	170	118	144	440	362	401
0.0069	2.69E-37	-1.7	405	394	400	1351	1438	1395
0.0197	2.52E-35	-1.31	861	761	811	2034	2284	2159
0.0108	7.16E-06	-0.798	320	313	317	539	647	593
0.0055	0.02412	-1.058	40	35	37.5	88	79	83.5
0.004	9.77E-09	-1.698	82	81	81.5	316	249	283
0.0166	2.35E-07	-0.888	351	409	380	712	807	760
0.0088	1.23E-23	-1.571	364	292	328	934	1160	1047
0.0155	9.07E-10	-0.919	469	403	436	878	888	883
0.0071	1.39E-14	-1.05	704	847	776	1710	1753	1732
0.0048	0.00407	-1.368	25	78	51.5	138	154	146
0.0106	1.68E-17	-0.843	1215	1191	1203	2213	2431	2322

Supplemental table S6

name	PR_K27me_LogFC_KO/	PR_K27me_KO	PR_K27me_WT	GB_K27me_LogFC_KO/	GB_K36_LogFC_KO/WT	GB_K27me_KO	GB_K27me_WT
10010F05R	0.26511	0.00232	0.00193	0.89175	0.1207	0.00125	0.00068
10032A03R	0.25267	0.00407	0.00341	1.52275	0.81493	0.0085	0.00296
00002E11R	1.29741	0.00447	0.00182	1.06012	-0.152	0.00354	0.0017
10300C02R	3.285	0.01331	0.00137	1.32525	-0.1963	0.00354	0.00141
210408I21R	-0.3854	0.00035	0.00046	0.73767	0.67785	0.00068	0.00041
10044O15R	-0.3136	0.00151	0.00188	1.07188	0.80299	0.00142	0.00067
10316D01R	4.2314	0.03632	0.00193	2.41019	0.17915	0.003	0.00057
21507P07R	1.06205	0.01342	0.00643	1.51053	0.13513	0.00476	0.00167
30089N07R	1.68222	0.00256	0.0008	0.85076	0.78795	0.00105	0.00058
30082N09R	0.40974	0.00076	0.00057	2.48505	0.65655	0.00098	0.00017
ABCC2	2.41173	0.00726	0.00137	1.01534	0.30071	0.02098	0.01038
ACACA	0.02788	0.0007	0.00068	0.83152	-0.2973	0.00187	0.00105
ACP5	1.12635	0.00906	0.00415	1.2787	-0.1298	0.01248	0.00515
ACSL3	0.57495	0.00407	0.00273	0.64219	-0.359	0.00172	0.0011
ACSS2	2.0438	0.06426	0.01559	1.91781	-0.5111	0.00978	0.00259
ADAP1	1.79453	0.0588	0.01695	1.30253	-0.4907	0.01364	0.00553
ADAP2	2.1229	0.02998	0.00688	1.09383	0.21698	0.01187	0.00556
ADCY1	0.19551	0.01668	0.01456	1.96506	-0.008	0.02408	0.00617
AI987944	1.87984	0.00209	0.00057	1.22691	-0.4183	0.00094	0.0004
ALDH6A1	0.84668	0.00511	0.00284	1.40767	-0.0972	0.00366	0.00138
ALDOB	1.03067	0.00349	0.00171	0.59923	0.36737	0.00221	0.00146
ALG10B	1.60186	0.00587	0.00193	1.05551	0.01299	0.00318	0.00153
ALG13	0.44464	0.00093	0.00068	1.18166	0.03747	0.00012	5.44E-05
AMER1	1.61672	0.00314	0.00102	1.05896	0.30525	0.00075	0.00036
ANKS4B	1.02927	0.00232	0.00114	0.87923	0.20123	0.00159	0.00087
ANO9	1.96151	0.01418	0.00364	2.44365	-0.1565	0.01477	0.00272
AOC2	0.64601	0.00837	0.00535	0.64601	-0.5251	0.01084	0.00693
AP5M1	2.61399	0.00209	0.00034	0.81338	-0.3581	0.00124	0.00071
APBB1	1.79994	0.02377	0.00683	2.66744	0.69775	0.02347	0.00369
APOL7A	0.19627	0.0075	0.00654	1.2134	-0.3646	0.00915	0.00395
AQP8	0.64967	0.005	0.00319	1.08622	2.59052	0.00812	0.00382
ARAP2	3.5854	0.01639	0.00137	1.6715	0.26433	0.00202	0.00063
ARHGAP32	0.78049	0.00215	0.00125	0.82981	0.34157	0.00184	0.00104
ARHGAP6	3.04293	0.01313	0.00159	1.24889	0.21167	0.00062	0.00026
ARHGDIG	2.7857	0.05334	0.00774	2.99422	-0.1513	0.06899	0.00866
ARHGEF9	4.82009	0.00645	0.00023	2.84699	0.01819	0.00188	0.00026
ARRDC2	0.86077	0.02882	0.01587	0.6911	-0.585	0.02092	0.01296
ATAD1	-0.0429	0.00221	0.00228	0.9799	-0.2455	0.00122	0.00062
ATM	0.9225	0.00151	0.0008	0.60872	-0.1193	0.00073	0.00048
ATP11C	1.0296	0.0014	0.00068	0.9968	0.08509	0.00032	0.00016
ATP13A3	0.03091	0.00267	0.00262	0.97794	-0.3586	0.00197	0.001
ATP2C1	2.29838	0.01063	0.00216	0.96028	0.27026	0.00178	0.00091
ATP7B	2.81884	0.01766	0.0025	1.2151	-0.3161	0.0057	0.00246
ATR	2.66386	0.0036	0.00057	0.66448	-0.4768	0.00067	0.00042

ATXN1L	1.1298	0.00174	0.0008	0.61555	-0.4641	0.00166	0.00108
ATXN3	0.3518	0.00203	0.00159	0.61113	-0.3407	0.00136	0.00089
AU041133	2.3518	0.00407	0.0008	1.9233	0.7411	0.00145	0.00038
AUTS2	2.66755	0.01156	0.00182	0.80894	0.09804	0.00191	0.00109
AVL9	1.27103	0.00796	0.0033	0.74518	-0.4498	0.00095	0.00057
AW112010	2.78996	0.00866	0.00125	2.03072	0.06623	0.00755	0.00185
AW549877	1.16804	0.00384	0.00171	0.61546	0.21102	0.00122	0.0008
30118H07F	0.13906	0.00401	0.00364	0.70267	0.6776	0.00192	0.00118
30354K17F	0.7	0.00407	0.0025	0.83739	0.48108	0.00252	0.00141
BAG4	0.32861	0.00343	0.00273	1.40475	0.09905	0.00214	0.00081
BBOF1	1.12347	0.00372	0.00171	1.66176	-0.4174	0.00315	0.00099
BC055324	1.2011	0.00314	0.00137	0.67588	0.63615	0.00105	0.00066
BCAP29	1.49869	0.00273	0.00097	0.72041	0.23385	0.00107	0.00065
BET1	2.22261	0.00372	0.0008	1.56794	1.19464	0.00101	0.00034
BICC1	4.40263	0.01685	0.0008	0.8425	-0.4304	0.00144	0.0008
BIVM	3.32684	0.00343	0.00034	2.01263	0.77249	0.00213	0.00053
BOD1L	0.84475	0.00296	0.00165	0.93465	-0.1685	0.00152	0.00079
BTBD10	-1.162	0.00081	0.00182	0.87374	-0.2525	0.00079	0.00043
BTG3	1.06989	0.0043	0.00205	0.79644	-0.0318	0.00171	0.00098
30074G19F	1.98215	0.01191	0.00302	2.54984	-0.4604	0.00467	0.0008
C3AR1	1.93791	0.00174	0.00046	1.00377	0.35026	0.00213	0.00106
CADM2	2.43566	0.01139	0.00211	1.64308	1.11063	0.0013	0.00042
CAPRIN2	0.54694	0.00715	0.00489	0.76764	0.49135	0.00253	0.00149
CARD14	0.26006	0.06403	0.05347	0.76169	-0.3983	0.02845	0.01678
CASC4	0.54085	0.06281	0.04317	1.38141	0.01028	0.00663	0.00254
CASP8AP2	-0.384	0.00174	0.00228	1.28652	-0.012	0.00079	0.00033
CBX5	1.46183	0.00877	0.00319	1.26611	-0.329	0.0035	0.00146
CCDC116	2.55829	0.02144	0.00364	0.7374	0.33159	0.01477	0.00886
CCDC117	0.09268	0.00413	0.00387	0.67318	-0.3308	0.00269	0.00169
CCDC138	0.99057	0.00203	0.00102	1.15147	0.69941	0.00122	0.00055
CCDC141	1.35259	0.00436	0.00171	1.28623	0.25292	0.00277	0.00113
CCDC43	1.34848	0.00941	0.0037	1.31471	0.10968	0.00703	0.00283
CCT4	-0.2906	0.00186	0.00228	0.6746	0.04682	0.00137	0.00086
CD36	1.83798	0.00325	0.00091	1.9965	0.19624	0.00353	0.00088
CD38	-0.4453	0.00267	0.00364	0.79026	0.3113	0.00839	0.00485
CDC14B	0.89436	0.00645	0.00347	0.9024	0.05842	0.00155	0.00083
CDH10	0.68221	0.00128	0.0008	1.76953	0.45582	0.0018	0.00053
CDH3	1.14158	0.02673	0.01212	0.90381	-0.0231	0.0042	0.00225
CDH6	2.47536	0.01708	0.00307	1.25131	0.4311	0.00203	0.00085
CDK12	0.14959	0.00442	0.00398	0.65171	-0.3008	0.00169	0.00107
CEACAM18	2.64793	0.01034	0.00165	2.14146	-0.2188	0.00972	0.0022
CENPI	0.25126	0.00081	0.00068	0.98649	-0.3077	0.00041	0.0002
CENPQ	0.47091	0.00221	0.00159	1.80764	0.87091	0.00182	0.00052
CEP83	-0.2327	0.00116	0.00137	0.96831	0.19001	0.00078	0.0004
CETN2	2.03136	0.00186	0.00046	1.34905	-0.2936	0.00073	0.00029
CFAP97	0.37444	0.00384	0.00296	1.18383	-0.0704	0.00282	0.00124
CFH	3.27375	0.00221	0.00023	2.64615	1.39145	0.00171	0.00027
CGREF1	0.34292	0.00418	0.0033	0.94204	-0.5075	0.00579	0.00302

CHD5	1.07957	0.05578	0.02639	0.61205	0.24442	0.01354	0.00886
CHML	2.53119	0.00198	0.00034	0.88099	1.13493	0.00103	0.00056
CHMP1B	1.05795	0.00936	0.00449	0.83798	-0.3872	0.00386	0.00216
CHODL	4.39834	0.00721	0.00034	3.48385	0.11579	0.00404	0.00036
CLIC3	2.3677	0.02818	0.00546	2.12467	-0.1295	0.02854	0.00654
CLRN3	0.73061	0.00151	0.00091	0.89116	-0.2652	0.0028	0.00151
CMC4	#DIV/0!	0.0007	0	1.72533	-0.2499	0.00039	0.00012
CMTR2	-0.317	0.00256	0.00319	1.12412	0.09425	0.00171	0.00078
CNTD1	1.16522	0.01174	0.00523	1.81514	-0.2841	0.0091	0.00259
COPRS	0.79622	0.01087	0.00626	1.33653	0.67839	0.01131	0.00448
CPEB2	-0.2057	0.00424	0.00489	1.17949	-0.1846	0.00126	0.00056
CPNE3	0.83798	0.00163	0.00091	0.71125	-0.5358	0.00075	0.00046
CPT1C	1.85149	0.01848	0.00512	1.28755	0.05125	0.02734	0.0112
CRBN	0.18542	0.00285	0.0025	1.01897	-0.2529	0.00196	0.00097
CREBRF	0.16824	0.01214	0.01081	1.07409	-0.3739	0.00444	0.00211
CST9	0.40763	0.00151	0.00114	1.35226	1.72135	0.00194	0.00076
CSTF2	1.61483	0.00244	0.0008	1.61537	-0.0183	0.00083	0.00027
CSTF3	0.73809	0.00285	0.00171	0.90505	0.13897	0.00118	0.00063
CTSE	-0.4875	0.01116	0.01564	0.78103	0.74342	0.00878	0.00511
CYP2C68	1.72128	0.00244	0.00074	1.01779	0.18541	0.00147	0.00072
CYP2J5	1.97443	0.00447	0.00114	2.38248	0.47135	0.00417	0.0008
CYP2J9	0.40763	0.00151	0.00114	0.91952	0.94875	0.00151	0.0008
CYP4F14	1.52611	0.02998	0.01041	1.01351	-0.4055	0.02366	0.01172
[illegible]1ERTD622	0.7408	0.00209	0.00125	0.64967	-0.2583	0.00165	0.00105
[illegible]30048N14R[illegible]	2.03107	0.0079	0.00193	1.98731	-0.3327	0.01026	0.00259
DDB2	1.02098	0.00877	0.00432	1.71523	-0.1231	0.00554	0.00169
DDIAS	2.19506	0.01563	0.00341	1.36791	-0.2541	0.0019	0.00073
DDX10	0.35295	0.00174	0.00137	0.7521	0.37402	0.00079	0.00047
DDX19B	-1.8162	0.00058	0.00205	0.60011	-0.1177	0.00225	0.00148
DDX52	-0.3178	0.00128	0.00159	1.31621	-0.4479	0.00324	0.0013
DENND1B	1.25126	0.00163	0.00068	1.03541	0.46133	0.00101	0.00049
DENND4A	0.72646	0.00395	0.00239	0.91959	-0.0647	0.00172	0.00091
DENND5B	2.6295	0.03695	0.00597	1.63655	0.25376	0.00661	0.00213
DHFR	0.35255	0.00407	0.00319	0.88712	-0.3425	0.00175	0.00095
DHH	3.92448	0.06479	0.00427	3.98052	-0.0117	0.07222	0.00457
DIS3	-0.0775	0.00151	0.00159	0.94801	0.04234	0.00102	0.00053
DKC1	1.76553	0.00116	0.00034	1.25126	-0.237	0.0007	0.00029
DLX4OS	0.63875	0.19935	0.12804	1.44155	0.1132	0.09478	0.0349
DUSP19	1.22325	0.00186	0.0008	1.2011	-0.0837	0.0013	0.00057
DZIP3	0.72291	0.00244	0.00148	0.86458	0.07606	0.00061	0.00034
EBAG9	1.75083	0.00325	0.00097	0.90478	-0.2502	0.00103	0.00055
EBP	0.9036	0.00128	0.00068	1.29248	0.46129	0.00204	0.00083
ECE2	2.78092	0.02894	0.00421	2.60461	-0.3333	0.04238	0.00697
ECHDC1	0.51711	0.00081	0.00057	1.21128	-0.0493	0.00071	0.00031
EFCAB14	1.12776	0.00895	0.0041	0.63931	0.11889	0.00135	0.00087
EFR3A	-0.0303	0.00267	0.00273	1.11979	0.1867	0.00141	0.00065
EHHADH	1.81409	0.0068	0.00193	1.15296	-0.292	0.00469	0.00211
EIF5A2	0.56352	0.0079	0.00535	0.81366	0.05569	0.0044	0.00251

ELMOD2	0.39336	0.00837	0.00637	0.73685	0.34049	0.00218	0.00131
EMC2	1.9036	0.00256	0.00068	0.93393	0.09752	0.0007	0.00036
ENPP4	3.33125	0.01604	0.00159	1.82958	1.59932	0.00613	0.00172
ENTPD8	0.86081	0.00558	0.00307	0.89178	0.24272	0.00704	0.00379
PB41L4AO	-2.144	0.0018	0.00796	0.77052	1.45408	0.00158	0.00093
EPS15	0.41788	0.00198	0.00148	0.93465	-0.1814	0.0008	0.00042
ERAP1	1.35119	0.00174	0.00068	0.81513	0.00208	0.00081	0.00046
ERC2	0.92754	0.02899	0.01524	0.64483	-0.0929	0.00318	0.00204
EXD2	0.15262	0.0043	0.00387	0.65292	0.35911	0.00196	0.00125
EXPH5	2.2404	0.0043	0.00091	1.04559	0.44731	0.00213	0.00103
FAM126B	1.98539	0.0018	0.00046	1.97733	1.0821	0.00131	0.00033
FAM13A	0.06914	0.00221	0.00211	0.65225	-0.4164	0.00153	0.00098
FAM20C	1.13131	0.03539	0.01615	0.8888	-0.4096	0.00798	0.00431
FAM229B	0.61633	0.0014	0.00091	0.58584	0.7289	0.00108	0.00072
FAM83F	1.95113	0.01848	0.00478	1.725	-0.2373	0.01871	0.00566
FAM8A1	1.49425	0.00465	0.00165	3.2536	0.56877	0.00468	0.00049
FAM92A	0.9036	0.00128	0.00068	0.94858	-0.4294	0.00063	0.00033
FASTKD2	0.7586	0.00616	0.00364	0.66992	0.74215	0.00124	0.00078
FCGR3	2.4907	0.00575	0.00102	1.18878	-0.1523	0.00246	0.00108
FGD4	0.72291	0.00244	0.00148	1.03767	0.33739	0.00158	0.00077
FGFBP1	0.25243	0.00244	0.00205	0.71049	-0.5281	0.0019	0.00116
FIGN	1.20718	0.02522	0.01092	1.2809	0.58358	0.00346	0.00142
FIGNL1	0.37552	0.00546	0.00421	0.78839	-0.048	0.00285	0.00165
FKTN	1.32615	0.00314	0.00125	0.80922	-0.4201	0.00142	0.00081
FLT4	0.80174	0.03817	0.0219	0.79929	-0.2203	0.01033	0.00594
FMR1	0.03136	0.00047	0.00046	1.02896	-0.1645	0.00023	0.00011
FOCAD	-0.3854	0.0007	0.00091	0.67776	0.13399	0.00105	0.00065
FOXD2OS	0.50364	0.04428	0.03123	1.03692	-0.0103	0.03377	0.01646
FYTTD1	-0.5913	0.00151	0.00228	0.59989	-0.4543	0.00103	0.00068
GAL3ST1	4.07876	0.08652	0.00512	2.52597	-0.5042	0.02943	0.00511
GAS2	0.9225	0.00151	0.0008	1.84857	1.25612	0.0011	0.0003
GATC	2.12104	0.00866	0.00199	1.73524	0.35625	0.00775	0.00233
GATM	0.5962	0.06862	0.04539	0.95519	0.48952	0.0274	0.01413
GC	2.04478	0.02394	0.0058	1.20477	0.15846	0.012	0.00521
GGH	-0.6615	0.00151	0.00239	0.74271	0.77294	0.00112	0.00067
GGPS1	1.38536	0.00267	0.00102	1.1298	-0.4915	0.00076	0.00035
GIMD1	1.10851	0.00442	0.00205	0.80294	-0.1245	0.00455	0.00261
GINS1	-0.2492	0.00163	0.00193	0.5854	0.17269	0.00151	0.00101
GKAP1	1.13094	0.01644	0.00751	0.67849	0.88533	0.0029	0.00181
GLCCI1	1.31345	0.00424	0.00171	1.08507	0.37002	0.00107	0.0005
GLMN	0.78251	0.00372	0.00216	0.93056	-0.4743	0.00218	0.00114
GM10033	0.74659	0.00267	0.00159	1.03056	-0.0057	0.00134	0.00066
GM11110	1.28437	0.01081	0.00444	0.92703	-0.5446	0.00942	0.00495
GM11992	0.45987	0.06165	0.04482	0.63593	0.22748	0.03249	0.02091
GM14326	0.54415	0.00116	0.0008	0.71476	-0.5311	0.00074	0.00045
GM15401	0.29551	0.00866	0.00705	0.66799	-0.3527	0.00984	0.00619
GM20939	0.53785	0.00314	0.00216	0.84458	0.66953	0.00151	0.00084
GM3336	0.40013	0.02289	0.01735	1.05684	-0.3629	0.01891	0.00909

GM7694	2.4059	0.00814	0.00154	2.45703	0.85818	0.00921	0.00168
GNG7	1.8533	0.03289	0.0091	1.31232	0.04403	0.01551	0.00624
GNPDA2	0.685	0.00494	0.00307	0.85917	-0.1701	0.00175	0.00097
GOLT1A	1.18419	0.0075	0.0033	1.26905	0.75172	0.00885	0.00367
GPA33	0.11356	0.00209	0.00193	0.95354	-0.3929	0.00525	0.00271
GPRIN3	1.14644	0.00302	0.00137	0.8586	0.4522	0.00142	0.00078
GRHL1	3.52272	0.01569	0.00137	1.78997	0.45262	0.00455	0.00132
GSPT1	1.28603	0.0043	0.00176	0.73331	-0.336	0.00152	0.00091
GTF3C3	0.95709	0.00552	0.00284	0.81485	-0.1875	0.00125	0.00071
GUCY1A2	0.73061	0.00151	0.00091	0.70702	0.16539	0.00061	0.00037
GUCY2C	0.96534	0.01755	0.00899	1.29992	0.1061	0.01908	0.00775
H2-K1	2.74137	0.07379	0.01104	2.702	-0.484	0.06094	0.00937
HARBI1	-0.4059	0.00198	0.00262	1.84556	0.05408	0.00641	0.00178
HARS2	1.15991	0.00203	0.00091	1.64892	-0.5221	0.00321	0.00102
HCCS	0.02987	0.00081	0.0008	1.35226	0.22871	0.00079	0.00031
HELLS	2.45145	0.00872	0.00159	2.15902	0.42665	0.00352	0.00079
HEPH	2.16522	0.00459	0.00102	2.96275	0.97586	0.00425	0.00054
HERC2	2.23383	0.00267	0.00057	1.26488	0.60363	0.0007	0.00029
HMGN5	1.03148	0.00023	0.00011	0.7676	1.46608	0.00039	0.00023
HNRNPA3	0.58308	0.00256	0.00171	0.70838	0.05824	0.0011	0.00067
HOMER1	0.19187	0.00221	0.00193	0.83388	-0.2286	0.00064	0.00036
HOOK1	1.29347	0.00418	0.00171	1.28603	-0.384	0.00091	0.00037
HPSE	1.88046	0.01005	0.00273	1.24959	-0.5133	0.00351	0.00148
HSD17B7	1.74119	0.01046	0.00313	1.81686	-0.3462	0.00422	0.0012
HTATIP2	2.19187	0.00442	0.00097	2.5016	0.21495	0.00286	0.0005
ICA1	0.54313	0.00564	0.00387	0.89466	0.25214	0.00184	0.00099
ICE2	0.07938	0.00174	0.00165	0.96151	-0.4528	0.00121	0.00062
IFIT2	0.99418	0.00453	0.00228	2.10354	0.19036	0.00795	0.00185
IFT81	-0.113	0.00221	0.00239	0.72362	-0.5837	0.0021	0.00127
IGSF9B	1.18837	0.01348	0.00592	0.98374	-0.0754	0.00324	0.00164
IL5RA	1.30209	0.00169	0.00068	0.81453	-0.3936	0.00179	0.00102
IL7	1.6885	0.00477	0.00148	0.78143	0.25066	0.00177	0.00103
ING3	1.7618	0.0054	0.00159	1.03101	-0.4266	0.00153	0.00075
IPO7	0.20186	0.00105	0.00091	0.73114	0.17778	0.00071	0.00043
IRF6	0.42833	0.05741	0.04266	0.91204	0.28096	0.02192	0.01165
ITGB3BP	-0.2916	0.00093	0.00114	0.98157	0.50821	0.00065	0.00033
ITIH2	0.37077	0.00471	0.00364	0.59907	0.40746	0.00712	0.0047
JRKL	1.6923	0.00221	0.00068	1.60933	2.14244	0.00142	0.00046
KBTBD3	2.17227	0.00308	0.00068	1.45342	0.39072	0.00106	0.00039
KBTBD8	0.80954	0.01017	0.0058	1.5182	-0.0855	0.00377	0.00132
KCNIP4	2.7408	0.00837	0.00125	2.76879	0.59562	0.00607	0.00089
KCNT2	4.03191	0.0093	0.00057	2.61358	1.24092	0.00198	0.00032
KIF18A	1.27902	0.00221	0.00091	0.86699	-0.0605	0.00086	0.00047
KIF24	2.23388	0.0075	0.00159	1.05936	-0.4597	0.00144	0.00069
KIF6	2.37526	0.00767	0.00148	1.12327	0.19267	0.00308	0.00142
KLHL11	1.31981	0.00511	0.00205	0.73354	-0.2764	0.00341	0.00205
KLHL13	2.84328	0.00081	0.00011	2.71671	-0.2023	0.00095	0.00015
KLHL15	0.73061	0.00151	0.00091	0.78606	-0.3127	0.00049	0.00028

KLHL28	1.83798	0.00163	0.00046	1.07466	-0.5351	0.0005	0.00024
KRT36	0.76736	0.00291	0.00171	0.89235	0.71103	0.00386	0.00208
KYAT3	1.17461	0.01348	0.00597	1.01602	-0.2414	0.0039	0.00193
L3MBTL3	-1.4476	0.00081	0.00222	0.59164	0.58061	0.00062	0.00041
LAD1	3.28951	0.10122	0.01035	3.3017	-0.2905	0.04336	0.0044
LATS1	0.02788	0.0007	0.00068	1.43164	-0.3251	0.00131	0.00048
LCP1	2.89075	0.02911	0.00393	1.38491	0.58988	0.00947	0.00363
LGALSL	1.06054	0.01424	0.00683	1.73333	0.16038	0.00629	0.00189
LGR4	1.30898	0.00564	0.00228	0.70141	-0.2234	0.00197	0.00121
LIN54	1.07938	0.00349	0.00165	1.22388	-0.3372	0.00166	0.00071
LIN7C	-0.9052	0.00134	0.0025	1.40371	0.47326	0.00198	0.00075
LMBRD2	0.58584	0.00273	0.00182	0.6849	-0.4081	0.00118	0.00073
LPL	1.57747	0.06723	0.02253	1.91874	1.16914	0.02392	0.00633
LRIF1	1.26189	0.00628	0.00262	1.46715	-0.3103	0.00262	0.00095
LRP11	0.71279	0.0165	0.01007	0.69899	-0.315	0.00488	0.003
LRRK2	3.33712	0.01034	0.00102	1.68382	0.49673	0.00185	0.00058
LYSMD2	0.41422	0.02464	0.01849	0.63244	0.1085	0.01002	0.00646
LZTFL1	1.21846	0.00569	0.00245	1.59386	0.73449	0.00399	0.00132
MAK16	0.7581	0.00558	0.0033	1.52874	-0.1698	0.00362	0.00125
MAP3K13	0.03081	0.00221	0.00216	1.1265	-0.2079	0.00381	0.00174
MAP9	2.08769	0.0104	0.00245	1.12581	0.33806	0.00264	0.00121
MARS2	0.56688	0.00674	0.00455	0.92656	-0.5673	0.00811	0.00426
MARVELD2	6.4469	0.02981	0.00034	2.67893	-0.5263	0.0077	0.0012
MARVELD3	0.75037	0.01598	0.0095	0.99839	0.14668	0.00753	0.00377
MBNL3	2.91688	0.00773	0.00102	1.81213	0.38286	0.00124	0.00035
MCM8	1.5854	0.00546	0.00182	1.60188	-0.1727	0.00392	0.00129
MDN1	-2.2926	0.00035	0.00171	0.59014	-0.128	0.00083	0.00055
MECR	-0.2392	0.01017	0.012	0.9723	-0.1764	0.00416	0.00212
MED14	-0.162	0.00081	0.00091	0.5854	0.18093	0.00038	0.00025
MEF2C	2.83271	0.00163	0.00023	1.78815	0.33812	0.00182	0.00053
MEGF9	1.07748	0.0036	0.00171	0.85306	0.02268	0.00106	0.00059
MEP1A	1.58965	0.00308	0.00102	0.75142	1.06548	0.00287	0.0017
MFSD4A	1.97775	0.03271	0.00831	2.09997	0.42715	0.01274	0.00297
MIS12	0.7	0.00407	0.0025	1.26227	0.82049	0.00243	0.00101
MKX	1.63499	0.10035	0.03231	1.66853	0.07393	0.01512	0.00476
MLH3	-0.5554	0.00047	0.00068	1.09841	-0.0545	0.00168	0.00078
MMP23	2.53071	0.02301	0.00398	2.35286	-0.1416	0.02107	0.00412
MMS22L	0.71049	0.00093	0.00057	0.58788	-0.3123	0.00051	0.00034
MOB1B	-0.1191	0.00424	0.00461	0.60719	-0.1635	0.00155	0.00102
MOGAT2	1.67656	0.03091	0.00967	0.8821	0.10753	0.00741	0.00402
MOSPD2	2.35963	0.00058	0.00011	0.64746	-0.1381	0.00036	0.00023
MPZL3	2.1603	0.0061	0.00137	1.37003	-0.0206	0.00388	0.0015
MRPL48	0.10517	0.00116	0.00108	1.01418	-0.4107	0.00193	0.00096
MRPL9	0.18267	0.00755	0.00666	0.99306	-0.214	0.00733	0.00368
MS4A8A	3.41517	0.01214	0.00114	2.46406	0.18575	0.01133	0.00205
MTIF2	0.61575	0.00209	0.00137	1.59922	-0.2338	0.00326	0.00108
MYCBP	1.81867	0.00843	0.00239	2.24507	-0.1308	0.00626	0.00132
MYO9A	1.47091	0.00442	0.00159	0.89113	-0.0403	0.00073	0.0004

MYZAP	2.61399	0.00209	0.00034	0.68929	-0.1508	0.00175	0.00109
NBEAL1	-0.1918	0.0014	0.00159	0.65785	0.70758	0.00063	0.0004
NCAM2	2.94148	0.00918	0.0012	2.01983	0.76156	0.00174	0.00043
NDUFAF4	1.03138	0.00209	0.00102	0.61596	0.32935	0.00136	0.00089
NECTIN3	1.46308	0.00314	0.00114	0.74333	0.54038	0.00121	0.00072
NFAT5	-0.3314	0.00081	0.00102	1.41684	-0.1473	0.00093	0.00035
NGEF	-0.1983	0.00169	0.00193	0.79184	0.41715	0.00331	0.00191
NIPA2	3.42824	0.00122	0.00011	1.43806	1.43248	0.00068	0.00025
NOD2	0.33188	0.00709	0.00563	0.66339	-0.214	0.00573	0.00362
NOL11	0.28388	0.0036	0.00296	0.76177	0.00893	0.00255	0.0015
NOL8	3.73591	0.00151	0.00011	1.29347	-0.3327	0.00097	0.00039
NOS1AP	4.164	0.03364	0.00188	2.7762	0.34405	0.00879	0.00128
NOSTRIN	2.54275	0.00331	0.00057	1.05538	-0.077	0.00306	0.00147
NOVA1	0.98099	0.02324	0.01178	1.2448	1.01481	0.00234	0.00099
NR0B2	0.71625	0.02243	0.01365	1.3181	0.91745	0.0239	0.00959
NRIP2	0.6406	0.00337	0.00216	0.7742	-0.4997	0.00566	0.00331
NRK	2.45619	0.00593	0.00108	0.75058	1.27275	0.00041	0.00025
NRXN1	0.69082	0.00174	0.00108	2.12637	0.53865	0.00494	0.00113
NSDHL	2.11791	0.00198	0.00046	0.9233	-0.3461	0.00045	0.00024
NSUN3	1.15555	0.00279	0.00125	1.31576	-0.4558	0.00169	0.00068
NT5E	3.03849	0.03178	0.00387	1.96572	-0.3886	0.00731	0.00187
NUDCD1	0.27866	0.00331	0.00273	1.13862	0.32689	0.00146	0.00066
NUDT12	1.2011	0.00314	0.00137	0.89086	0.30837	0.00182	0.00098
NUDT7	1.03138	0.00209	0.00102	0.7408	-0.1536	0.00134	0.0008
NUS1	-0.5544	0.00209	0.00307	0.95796	0.13356	0.00149	0.00077
NXNL2	2.17873	0.01493	0.0033	2.53605	0.05237	0.01616	0.00279
NXT2	0.47166	0.00442	0.00319	1.80779	0.4975	0.00259	0.00074
ONECUT1	0.74492	0.12812	0.07645	1.06279	0.01629	0.05027	0.02406
OPRD1	1.90997	0.04724	0.01257	0.75238	-0.091	0.01262	0.00749
ORC2	2.20071	0.00209	0.00046	1.04081	0.46845	0.00119	0.00058
ORC4	1.14389	0.00151	0.00068	0.87879	0.03817	0.00053	0.00029
OTUD4	0.16742	0.00192	0.00171	0.9291	0.1443	0.00113	0.00059
OVCA2	0.86417	0.01947	0.01069	0.66299	0.04625	0.01886	0.01191
PABPC4L	1.35259	0.00436	0.00171	1.49033	-0.5081	0.00401	0.00143
PAIP1	0.92753	0.00314	0.00165	0.8602	-0.3519	0.0007	0.00039
PAK6	0.5104	0.00616	0.00432	0.96845	-0.2394	0.01493	0.00763
PALB2	0.80736	0.00279	0.00159	1.36988	-0.1432	0.00117	0.00045
PAQR4	2.70204	0.08698	0.01337	3.6179	-0.2555	0.09908	0.00807
PAQR6	1.63196	0.02574	0.00831	1.23133	0.72625	0.02514	0.01071
PARD3B	3.97085	0.01249	0.0008	1.19734	0.90391	0.00158	0.00069
PCDH10	4.23596	0.03643	0.00193	2.40953	0.25135	0.00368	0.00069
PCDH11X	-0.4429	0.00209	0.00284	1.1052	0.44998	0.00031	0.00015
PCDH19	1.68742	0.03829	0.01189	1.86163	0.8514	0.00386	0.00106
PCDHB17	0.89235	0.00232	0.00125	1.03138	0.02861	0.00384	0.00188
PCDHB20	1.44553	0.00465	0.00171	1.5547	-0.3255	0.00472	0.00161
PCSK6	3.09151	0.04073	0.00478	1.40709	-0.2109	0.00385	0.00145
PCTP	0.7289	0.01348	0.00813	0.66068	-0.201	0.00528	0.00334
PDGFRL	1.3827	0.00564	0.00216	0.95374	0.17525	0.00473	0.00244

PDIA3	0.82252	0.00825	0.00467	1.68355	0.34181	0.0046	0.00143
PDPR	0.7221	0.00488	0.00296	1.18266	-0.0913	0.00207	0.00091
PFKFB1	-0.9701	0.00041	0.0008	0.66786	0.38493	0.00052	0.00033
PHF10	1.10457	0.00465	0.00216	0.67057	-0.4685	0.00257	0.00162
PHF14	1.20761	0.00302	0.00131	0.63063	0.19964	0.00097	0.00063
PHF20L1	0.27902	0.00221	0.00182	0.62878	-0.042	0.00114	0.00074
PHF7	-0.2498	0.00325	0.00387	0.80219	-0.3815	0.00686	0.00393
PHLPP2	1.35119	0.00174	0.00068	0.65131	-0.45	0.00151	0.00096
PHTF2	0.47091	0.00221	0.00159	0.58788	0.00502	0.00094	0.00063
PIGG	1.11141	0.00639	0.00296	1.68973	0.02604	0.00419	0.0013
PIK3AP1	2.79333	0.02406	0.00347	1.43487	-0.1648	0.00672	0.00248
PIK3C2B	2.52842	0.01772	0.00307	1.58954	-0.3244	0.0061	0.00203
PIK3C2G	2.11356	0.00418	0.00097	0.7437	0.27155	0.00124	0.00074
PILRA	0.55425	0.00535	0.00364	1.17273	-0.0966	0.0044	0.00195
PKIA	1.68834	0.01302	0.00404	2.22172	0.59381	0.00382	0.00082
PLA2G4C	3.61905	0.0007	5.67E-05	0.87032	-0.4743	0.00068	0.00037
PLCXD2	0.76781	0.00465	0.00273	1.10178	-0.1828	0.00269	0.00125
PLEKHF2	0.58308	0.00256	0.00171	0.69919	-0.5704	0.00114	0.0007
PMS1	1.17728	0.0018	0.0008	0.81572	0.39764	0.00109	0.00062
PNPT1	-0.7054	0.00105	0.00171	0.90124	-0.3704	0.00131	0.0007
POGK	2.60436	0.01522	0.0025	1.6078	1.01671	0.0077	0.00253
POLE	0.34108	0.0036	0.00284	0.69458	-0.4451	0.00288	0.00178
POU2F1	1.515	0.00325	0.00114	0.84537	0.48584	0.001	0.00056
PPIP5K1	1.92256	0.01273	0.00336	0.66889	-0.2469	0.00547	0.00344
PPIP5K2	0.94858	0.00198	0.00102	1.15911	0.14375	0.00089	0.0004
PPP1R12B	1.16163	0.00267	0.0012	0.78975	0.49143	0.00247	0.00143
PPP1R13B	1.51851	0.03585	0.01251	1.27072	-0.3673	0.00397	0.00165
PPWD1	0.515	0.00163	0.00114	0.81911	-0.4074	0.00112	0.00063
PREX2	3.55879	0.03486	0.00296	2.58957	0.63547	0.00549	0.00091
PRKAA2	1.01705	0.0061	0.00302	1.17278	-0.3066	0.00167	0.00074
PRMT3	1.73141	0.00302	0.00091	2.01382	0.67509	0.00148	0.00037
PRMT7	0.79493	0.00198	0.00114	0.91404	-0.566	0.00184	0.00097
PROM1	1.01202	0.05805	0.02878	0.62611	-0.2737	0.00802	0.0052
PRPF40A	0.94801	0.00395	0.00205	0.78326	-0.286	0.00096	0.00056
PRR15L	0.9851	0.02714	0.01371	0.95699	0.14284	0.02092	0.01077
PSMA4	1.47864	0.00523	0.00188	1.36654	-0.1656	0.00515	0.002
PTPN4	0.03033	0.00232	0.00228	1.14608	0.52066	0.00071	0.00032
PTPRD	1.69118	0.01139	0.00353	2.02125	0.54776	0.00197	0.00048
PYCR1	0.45684	0.03521	0.02565	1.23457	-0.4882	0.02691	0.01144
RABEP1	0.76472	0.0086	0.00506	0.72791	-0.2834	0.00161	0.00097
RAD54B	2.72991	0.00227	0.00034	0.6694	0.06471	0.00084	0.00053
RAET1D	0.86362	0.00331	0.00182	1.89618	1.42014	0.00436	0.00117
RANBP17	0.76714	0.00494	0.0029	1.08148	0.32198	0.00237	0.00112
RANBP6	-0.0928	0.00715	0.00762	1.28215	1.21201	0.00852	0.00351
RASEF	0.39375	0.00105	0.0008	1.18071	1.13825	0.00083	0.00037
RASGRP3	0.52548	0.0018	0.00125	0.68083	0.66716	0.00237	0.00148
RBBP8NL	0.76133	0.01755	0.01035	0.78717	-0.4981	0.01131	0.00656
RBBP9	0.34108	0.0036	0.00284	1.04447	0.29632	0.00418	0.00203

RDH11	-0.317	0.00256	0.00319	1.56054	-0.5513	0.00406	0.00137
RELN	-0.3194	0.00657	0.00819	0.63784	0.35327	0.0036	0.00231
RFC4	1.49033	0.00256	0.00091	2.23862	-0.3016	0.00491	0.00104
RFX7	0.20071	0.00209	0.00182	0.69365	0.00066	0.00051	0.00032
RGS11	2.595	0.04399	0.00728	1.01142	-0.5093	0.01543	0.00765
RHOBTB3	0.88099	0.00105	0.00057	1.01425	-0.0954	0.00084	0.00041
RICTOR	0.25302	0.00163	0.00137	0.65817	-0.1335	0.00117	0.00074
RIDA	0.31981	0.00256	0.00205	1.41258	0.01982	0.00227	0.00085
RNF123	0.04893	0.0093	0.00899	1.01889	-0.5641	0.00919	0.00453
RNF219	1.11791	0.00198	0.00091	1.76327	0.32065	0.00175	0.00051
RPF2	0.39375	0.00105	0.0008	1.09707	-0.23	0.00098	0.00046
RPL22L1	0.94998	0.00506	0.00262	1.76554	-0.5289	0.00727	0.00214
RPL39	0.76857	0.00174	0.00102	0.6146	-0.2028	0.00155	0.00101
RPP25	1.20185	0.06961	0.03026	1.14689	-0.213	0.08703	0.0393
RPS6KA5	0.59487	0.014	0.00927	1.02064	0.35731	0.00141	0.00069
RPS6KL1	1.48852	0.01261	0.00449	1.11394	-0.495	0.01241	0.00573
RPUSD2	0.11096	0.0043	0.00398	0.76802	-0.5096	0.00518	0.00304
RSPRY1	2.26148	0.00709	0.00148	1.46587	-0.3503	0.00186	0.00067
RTN2	1.08371	0.04184	0.01974	1.47689	0.15957	0.01045	0.00376
S100PBP	0.45508	0.00296	0.00216	0.6829	-0.1865	0.00138	0.00086
SAMD9L	0.26511	0.00232	0.00193	0.60258	-0.5274	0.00066	0.00044
SAXO2	2.19233	0.00546	0.0012	2.48558	2.21686	0.00737	0.00132
SC5D	-0.3607	0.00186	0.00239	0.94898	-0.4134	0.00176	0.00091
SCAI	1.26347	0.00546	0.00228	0.6702	-0.0397	0.00128	0.00081
SCAMP5	1.05612	0.03643	0.01752	1.1414	0.53309	0.01183	0.00536
SCEL	1.15648	0.00418	0.00188	1.62405	-0.3611	0.00179	0.00058
SEC14L2	1.72178	0.08105	0.02457	1.60246	0.10755	0.02451	0.00807
SEPSECS	1.24517	0.00674	0.00284	0.73745	0.05669	0.00167	0.001
SGPP2	2.52333	0.02551	0.00444	0.67973	0.64177	0.0039	0.00244
SHCBP1	0.29373	0.0007	0.00057	0.72797	0.47937	0.00086	0.00052
SHTN1	0.96346	0.01098	0.00563	0.75612	0.21005	0.0024	0.00142
SIMC1	0.62803	0.00686	0.00444	0.91733	-0.4057	0.00144	0.00076
SIS	1.9036	0.00128	0.00034	1.34255	0.20834	0.00127	0.0005
SLC10A2	0.478	0.00174	0.00125	1.15325	1.22298	0.00117	0.00053
SLC25A15	1.65242	0.00912	0.0029	0.67075	-0.3016	0.00363	0.00228
SLC25A23	2.0073	0.03202	0.00796	0.80081	-0.1749	0.0095	0.00545
SLC26A9	0.26511	0.00232	0.00193	1.44199	-0.3046	0.00682	0.00251
SLC27A6	1.03137	0.00302	0.00148	1.01873	-0.1838	0.00147	0.00072
SLC30A4	1.15555	0.00279	0.00125	0.9432	0.42949	0.0026	0.00135
SLC31A1	0.658	0.00198	0.00125	0.72006	-0.2633	0.00145	0.00088
SLC39A10	4.81718	0.0032	0.00011	1.71428	0.74853	0.00188	0.00057
SLCO5A1	0.95821	0.02365	0.01217	2.1256	0.11978	0.0098	0.00225
SLF1	-0.3854	0.00035	0.00046	1.22833	0.47144	0.00059	0.00025
SMARCA1	3.15464	0.00203	0.00023	1.65888	0.02828	0.00028	9.02E-05
SMCO4	0.78568	0.00628	0.00364	0.63675	0.24914	0.00186	0.0012
SMIM24	2.23206	0.02005	0.00427	2.48517	0.76563	0.0275	0.00491
SMN1	1.53747	0.00628	0.00216	0.60228	0.03516	0.00257	0.0017
SMOC1	0.80358	0.00477	0.00273	0.82833	-0.2884	0.00292	0.00164

SNHG3	0.03136	0.0014	0.00137	0.76857	0.38779	0.00298	0.00175
SNX14	0.03136	0.0014	0.00137	0.88166	-0.1611	0.00113	0.00061
SOWAHC	0.99711	0.0025	0.00125	2.6603	-0.2978	0.0024	0.00038
SOX11	1.44597	0.01116	0.0041	0.86019	-0.0653	0.00821	0.00452
SPACA6	0.16545	0.00262	0.00233	1.28856	0.79141	0.00525	0.00215
SPATS2L	0.2411	0.02911	0.02463	1.35856	0.87675	0.00706	0.00275
SPC25	0.37463	0.00192	0.00148	0.70879	0.06169	0.00181	0.00111
SPINT1	0.56976	0.11871	0.07998	0.60198	-0.0156	0.0572	0.03768
SPRED1	-0.0135	0.00372	0.00375	0.67741	-0.0249	0.00136	0.00085
SRL	0.72646	0.00395	0.00239	0.80883	0.13317	0.00338	0.00193
SSBP1	1.93444	0.00674	0.00176	2.26718	0.19798	0.00221	0.00046
SSH2	0.1298	0.00174	0.00159	0.63872	-0.1192	0.00144	0.00093
STK39	2.72672	0.02899	0.00438	0.85444	-0.2393	0.00224	0.00124
STRN3	0.69891	0.00314	0.00193	0.61078	-0.0804	0.00062	0.00041
STXBP3-PS	1.75379	0.00192	0.00057	2.41767	1.98457	0.00446	0.00083
SUCLA2	-0.4453	0.00267	0.00364	0.98019	-0.2316	0.00135	0.00068
SULT1B1	-0.4285	0.00093	0.00125	0.95125	0.36533	0.00149	0.00077
SULT1D1	1.93791	0.00174	0.00046	2.57273	1.27277	0.00155	0.00026
SVIP	1.32615	0.00314	0.00125	2.72991	1.56815	0.00154	0.00023
SYNGR3	1.45874	0.11429	0.04158	1.18488	-0.1764	0.08992	0.03955
SYTL5	#DIV/0!	0.0007	0	0.82203	0.34093	0.00028	0.00016
TACO1	1.14786	0.00895	0.00404	0.87129	0.31832	0.00676	0.0037
TAF1D	0.95604	0.00221	0.00114	0.98751	0.00114	0.00107	0.00054
TAF7	0.55818	0.00285	0.00193	0.68033	0.71838	0.00103	0.00064
TANC2	0.81017	0.00599	0.00341	1.01768	0.33984	0.00249	0.00123
TARSL2	0.04493	0.02359	0.02287	1.06559	-0.2365	0.00277	0.00132
TBC1D32	-0.2324	0.00058	0.00068	0.79928	0.27521	0.0006	0.00034
TBC1D4	-0.2257	0.00418	0.00489	0.7934	-0.006	0.00169	0.00098
TBPL1	0.76857	0.00174	0.00102	0.71661	-0.1478	0.00094	0.00057
TCAM1	1.05957	0.01162	0.00558	0.68218	0.11012	0.01213	0.00756
TCEA3	1.10371	0.05293	0.02463	0.60345	0.00652	0.01224	0.00806
TCF23	1.1489	0.0111	0.00501	1.74082	-0.5708	0.01107	0.00331
TDP2	1.49033	0.00128	0.00046	0.76819	-0.2757	0.00083	0.00049
TEFM	1.35295	0.00349	0.00137	1.00377	-0.4176	0.0035	0.00175
TENM1	#NUM!	0	0.00023	1.46482	0.64587	0.0006	0.00022
TFCP2L1	1.52117	0.01029	0.00358	0.88051	-0.1582	0.00294	0.0016
THSD4	0.22325	0.00186	0.00159	0.71964	-0.3856	0.00201	0.00122
TIGD2	0.515	0.00163	0.00114	0.86203	-0.2592	0.00176	0.00097
TIRAP	0.95919	0.00232	0.0012	1.04612	-0.0787	0.00204	0.00099
TM6SF2	2.37243	0.02975	0.00575	1.5708	0.43262	0.01859	0.00626
TMC3	1.10753	0.00784	0.00364	1.18214	0.22987	0.00487	0.00215
TMEM117	2.55	0.01366	0.00233	0.64158	0.2579	0.00172	0.0011
TMEM41B	0.40317	0.00256	0.00193	1.64254	-0.4671	0.00136	0.00044
TMEM62	1.2404	0.00215	0.00091	0.82294	0.1097	0.00271	0.00153
TMEM64	0.1329	0.00256	0.00233	0.85619	0.25484	0.0013	0.00072
TMEM67	1.16734	0.00256	0.00114	0.6157	0.32876	0.00142	0.00093
TMEM69	0.61575	0.00209	0.00137	2.34698	0.02867	0.00158	0.00031
TMEM74	3.28352	0.01662	0.00171	3.37177	2.18124	0.02331	0.00225

TMEM8	0.80122	0.10685	0.06132	1.05241	-0.5063	0.07977	0.03846
TMTC2	-0.7054	0.00105	0.00171	0.74746	-0.306	0.00068	0.00041
TMX4	-0.0656	0.00337	0.00353	0.67283	0.20828	0.00117	0.00074
TRIM68	1.88537	0.00546	0.00148	1.66566	-0.2389	0.00311	0.00098
TRMT10C	-0.4063	0.00378	0.00501	1.26511	-0.1081	0.00343	0.00143
TRNT1	1.20071	0.00209	0.00091	2.01157	1.31771	0.00206	0.00051
TSPAN12	1.25138	0.01517	0.00637	1.9318	-0.1143	0.00375	0.00098
TTC26	1.02987	0.00163	0.0008	1.30369	0.46213	0.00241	0.00098
TWISTNB	0.87464	0.00459	0.0025	0.96798	0.29866	0.00082	0.00042
TXLNG	-1.5536	0.00047	0.00137	0.87879	-0.576	0.0005	0.00027
U2SURP	1.6746	0.00291	0.00091	1.27563	-0.5111	0.00081	0.00033
UBR1	0.09996	0.00122	0.00114	1.07079	-0.4235	0.00207	0.00099
UCHL5	0.658	0.00198	0.00125	1.24212	0.8714	0.00102	0.00043
UFL1	0.70838	0.00186	0.00114	1.37525	1.04494	0.00066	0.00026
UGT2A3	1.14389	0.00151	0.00068	1.98473	1.52235	0.00177	0.00045
UGT2B35	2.02609	0.00093	0.00023	1.20187	1.57911	0.00051	0.00022
UGT2B36	-0.5605	0.00023	0.00034	1.70773	0.77019	0.00084	0.00026
UHMK1	1.61399	0.00209	0.00068	0.94123	-0.0303	0.00173	0.0009
ULBP1	-0.5829	0.00198	0.00296	0.78182	0.95623	0.00537	0.00313
UNC13B	3.83391	0.01947	0.00137	2.77326	-0.1163	0.00494	0.00072
UNC13C	-2.0558	0.00047	0.00193	0.62972	0.55172	0.00139	0.0009
UPRT	#NUM!	0	0.00011	1.51711	-0.0411	0.00033	0.00012
URB2	1.30209	0.00169	0.00068	0.7731	0.07619	0.00125	0.00073
USP16	2.02987	0.00325	0.0008	1.38304	-0.0193	0.00293	0.00112
USP45	-0.4538	0.00058	0.0008	0.67993	-0.4821	0.00078	0.00049
USP49	0.66797	0.00569	0.00358	1.54641	-0.3227	0.00569	0.00195
UTP14B	0.57495	0.00407	0.00273	1.64172	-0.2667	0.00207	0.00066
VIL1	1.62766	0.00703	0.00228	2.4481	-0.2037	0.01124	0.00206
VKORC1L1	0.01577	0.00552	0.00546	0.76504	-0.4655	0.00151	0.00089
VPS26B	0.46093	0.0036	0.00262	0.68282	-0.2478	0.0015	0.00093
VPS41	1.11791	0.00198	0.00091	0.77345	-0.2106	0.00097	0.00057
VPS50	1.3098	0.00198	0.0008	0.72391	-0.1353	0.00115	0.00069
VSIG1	0.02788	0.00035	0.00034	1.11852	-0.3917	0.00074	0.00034
WDR17	0.89891	0.00668	0.00358	1.09782	0.17146	0.00251	0.00117
WDR72	1.06661	0.00477	0.00228	1.71468	0.10711	0.00374	0.00114
XPNPEP3	0.57899	0.00221	0.00148	0.76773	0.01535	0.00182	0.00107
XRN2	0.64746	0.00267	0.00171	1.22134	-0.5716	0.00169	0.00072
YARS2	0.87773	0.00209	0.00114	1.3926	-0.2699	0.00188	0.00072
YDJC	2.54774	0.02162	0.0037	3.57826	0.35848	0.04314	0.00361
YES1	0.16742	0.00192	0.00171	0.71233	-0.2837	0.00106	0.00065
YIPF6	0.97457	0.00291	0.00148	0.73061	0.26099	0.00081	0.00049
ZBTB26	0.515	0.00163	0.00114	0.61596	0.02128	0.00086	0.00056
ZBTB41	1.31663	0.00227	0.00091	1.09707	0.28004	0.00076	0.00036
ZC4H2	2.25126	0.00163	0.00034	0.95603	-0.2061	0.00069	0.00035
ZCWPW1	0.33188	0.00709	0.00563	0.6345	-0.4444	0.00344	0.00221
ZDHHC23	1.97426	0.02324	0.00592	1.32317	-0.3533	0.0126	0.00504
ZFAND4	0.26511	0.00232	0.00193	0.96518	-0.4222	0.00181	0.00093
ZFP101	0.50059	0.00523	0.0037	1.17653	0.32378	0.00325	0.00144

ZFP13	1.10281	0.00599	0.00279	1.11755	-0.3673	0.00883	0.00407
ZFP2	-0.3128	0.00238	0.00296	0.79917	-0.0972	0.00184	0.00106
ZFP248	1.20071	0.00418	0.00182	1.15663	-0.3366	0.00221	0.00099
ZFP280C	0.03346	0.00058	0.00057	0.97795	0.2577	0.00036	0.00018
ZFP318	1.97531	0.01342	0.00341	1.24929	-0.1296	0.00467	0.00197
ZFP326	0.03096	0.00291	0.00284	1.08334	-0.2615	0.00123	0.00058
ZFP329	1.83798	0.00163	0.00046	1.58965	-0.1941	0.00123	0.00041
ZFP354A	1.56207	0.00302	0.00102	1.09826	-0.0201	0.00226	0.00106
ZFP420	2.39867	0.0018	0.00034	0.97921	0.05479	0.00167	0.00085
ZFP422	0.87361	0.00604	0.0033	0.73605	-0.5803	0.00525	0.00315
ZFP426	0.83798	0.00081	0.00046	1.10405	0.07755	0.00146	0.00068
ZFP454	1.10101	0.00488	0.00228	0.71125	0.57784	0.00257	0.00157
ZFP51	0.49641	0.00506	0.00358	0.82898	0.00285	0.00183	0.00103
ZFP54	0.73125	0.01133	0.00683	1.55283	0.41475	0.00387	0.00132
ZFP560	0.85467	0.00267	0.00148	0.82113	-0.0732	0.00072	0.00041
ZFP566	1.20094	0.00523	0.00228	1.39709	-0.4184	0.00437	0.00166
ZFP595	2.48505	0.00128	0.00023	1.24212	-0.2247	0.00102	0.00043
ZFP606	0.86732	0.00145	0.0008	0.70822	0.18037	0.00093	0.00057
ZFP617	1.39976	0.0036	0.00137	0.80865	0.44708	0.00213	0.00121
ZFP62	-0.0153	0.0036	0.00364	0.59165	0.00925	0.00272	0.0018
ZFP644	0.55818	0.00285	0.00193	0.58792	-0.0508	0.00113	0.00075
ZFP692	0.103	0.00477	0.00444	0.93289	0.26099	0.00924	0.00484
ZFP729B	3.12443	0.00099	0.00011	0.63537	-0.1417	0.00065	0.00042
ZFP738	0.61633	0.0014	0.00091	0.81939	-0.4664	0.00124	0.0007
ZFP763	1.73125	0.00755	0.00228	1.17498	-0.5498	0.00355	0.00157
ZFP770	1.41184	0.00575	0.00216	2.05318	-0.3056	0.00284	0.00068
ZFP788	2.58584	0.00273	0.00046	1.61497	0.8035	0.00078	0.00026
ZFP800	0.49197	0.01592	0.01132	0.92587	0.24635	0.00951	0.00501
ZFP81	1.9233	0.00604	0.00159	1.7856	0.55957	0.00187	0.00054
ZFP846	1.37688	0.00355	0.00137	0.60608	-0.0678	0.00191	0.00126
ZFP85	2.44464	0.00186	0.00034	2.4602	0.68099	0.00127	0.00023
ZFP850	0.41412	0.00174	0.00131	0.96303	0.27617	0.00099	0.00051
ZFP866	1.99057	0.00407	0.00102	1.00582	-0.5056	0.00292	0.00146
ZFP874A	1.56583	0.00337	0.00114	1.5157	0.77431	0.00262	0.00091
ZFP933	1.44699	0.0014	0.00051	1.0807	-0.3625	0.0008	0.00038
ZFP937	-0.0837	0.0014	0.00148	1.0307	0.40568	0.00122	0.0006
ZFP940	0.22325	0.00093	0.0008	0.93791	0.32699	0.00108	0.00056
ZFP946	1.49033	0.00128	0.00046	1.00325	0.40513	0.00055	0.00027
ZFP951	-0.485	0.00081	0.00114	0.82504	0.36482	0.00118	0.00067
ZFP953	1.29546	0.0014	0.00057	2.46821	0.20337	0.00213	0.00039
ZFP955B	0.37178	0.00221	0.00171	1.35226	-0.3387	0.00046	0.00018
ZFP959	0.96514	0.01499	0.00768	1.48129	-0.5297	0.00418	0.0015
ZFP960	2.14389	0.00151	0.00034	1.56793	0.17364	0.0004	0.00013
ZFP961	0.80979	0.00668	0.00381	0.63241	0.4566	0.00233	0.00151
ZFP964	1.60408	0.00709	0.00233	1.43304	-0.2167	0.00521	0.00193
ZFP971	0.25361	0.00122	0.00102	0.95603	-0.1854	0.00105	0.00054
ZKSCAN8	2.49101	0.0032	0.00057	1.46308	-0.4977	0.00105	0.00038
ZMYM5	-0.5524	0.00105	0.00154	0.78754	-0.1509	0.00162	0.00094

10002L01R	0.71049	0.00093	0.00057	0.1767	-0.5551	0.00079	0.0007
10002M06F	2.14389	0.00151	0.00034	0.51499	0.09234	0.00029	0.0002
32404H12R	0.79639	0.01185	0.00683	0.58131	-0.5567	0.01474	0.00985
)30440I19R	1.69121	0.0011	0.00034	-0.0774	0.49705	0.00088	0.00092
30107N01F	1.5547	0.00267	0.00091	#DIV/0!	4.07477	0.00269	0
AASDHPPT	2.14468	0.00302	0.00068	0.11471	0.28216	0.00043	0.00039
ABCA5	0.84597	0.00808	0.00449	0.3812	0.0639	0.00182	0.0014
ABCE1	0.93791	0.00174	0.00091	0.18285	-0.0446	0.00099	0.00087
ADAMTS9	2.05173	0.0283	0.00683	0.4369	0.33412	0.00199	0.00147
AFM	1.8351	0.01522	0.00427	0.54382	0.71074	0.00997	0.00684
AHCYL2	0.86081	0.00558	0.00307	0.58007	-0.4892	0.00209	0.0014
AKAP11	0.95604	0.00221	0.00114	-0.0459	-0.4437	0.00086	0.00089
AKR1C13	1.0296	0.0014	0.00068	-0.5547	0.17701	0.00029	0.00043
ALKBH8	0.83798	0.00081	0.00046	-0.1297	-0.5336	0.00047	0.00051
ANXA10	0.7676	0.00058	0.00034	0.04306	0.04941	0.00063	0.00061
AP4E1	1.38854	0.00477	0.00182	0.38813	-0.5232	0.00164	0.00125
AP4S1	0.73141	0.00302	0.00182	0.2937	-0.2733	0.0014	0.00114
APBA1	0.66085	0.02069	0.01308	0.31243	0.20529	0.00599	0.00482
APOOL	2.84328	0.00081	0.00011	-0.1208	-0.4297	0.00026	0.00028
AQR	1.5758	0.00628	0.00211	-0.0374	-0.3628	0.00117	0.0012
ARFGEF1	1.09479	0.00267	0.00125	-0.0268	-0.0845	0.0009	0.00092
ARID4B	1.38536	0.00267	0.00102	0.0685	-0.0899	0.0005	0.00047
ASB3	1.20186	0.00105	0.00046	0.38616	-0.1489	0.00082	0.00063
ASXL2	0.71049	0.00093	0.00057	-0.398	-0.4442	0.00045	0.0006
ATP11B	0.72023	0.00291	0.00176	-0.3691	-0.3372	0.00081	0.00105
ATP1B1	1.09776	0.01023	0.00478	-0.272	-0.4905	0.00197	0.00238
ATP8A1	0.61017	0.00755	0.00495	0.28589	-0.2457	0.00135	0.00111
BCHE	2.35119	0.00174	0.00034	0.26303	0.64932	0.00071	0.00059
BDP1	1.32099	0.00256	0.00102	0.06336	-0.2391	0.0009	0.00086
BIRC6	0.73809	0.00569	0.00341	0.26377	-0.5769	0.00108	0.0009
BMP8A	1.40569	0.033	0.01246	-0.0249	-0.3711	0.00899	0.00914
30018D20F	0.74659	0.00134	0.0008	0.36726	0.11496	0.00086	0.00066
CAB39L	1.16946	0.00128	0.00057	0.48135	-0.0827	0.00088	0.00063
CAR13	0.6746	0.00291	0.00182	0.4751	0.30648	0.00136	0.00098
CCDC127	1.14389	0.00151	0.00068	-0.0693	0.08868	0.00095	0.00099
CCDC15	0.89915	0.00244	0.00131	0.134	0.97784	0.00094	0.00085
CCDC50	0.61633	0.0014	0.00091	0.27444	-0.5149	0.00099	0.00082
CCDC88A	0.93225	0.00413	0.00216	0.39328	0.15808	0.00082	0.00063
CCNY	0.64425	0.00302	0.00193	-0.3655	-0.3882	0.00054	0.00069
CCR1	1.34905	0.00058	0.00023	0.48844	0.40684	0.00058	0.00042
CD274	2.51711	0.00325	0.00057	-0.3685	-0.1011	0.00154	0.00198
CDHR5	0.83312	0.02533	0.01422	0.19788	-0.545	0.01199	0.01046
CDK5RAP1	1.06	0.00581	0.00279	0.26224	-0.5655	0.00195	0.00163
CEP290	0.61514	0.00105	0.00068	0.15514	-0.2242	0.00062	0.00056
CEP295	1.54037	0.0043	0.00148	0.22521	-0.5626	0.00088	0.00075
CES2C	0.9036	0.00128	0.00068	0.44526	1.34889	0.00056	0.00041
CFAP36	0.59769	0.00465	0.00307	0.25046	-0.3563	0.00134	0.00113
CHN2	1.0164	0.00575	0.00284	0.22698	-0.4003	0.00225	0.00192

CLDN20	0.61596	0.00157	0.00102	-1.1432	2.45721	0.00211	0.00465
CLIC6	1.88846	0.04317	0.01166	0.29283	0.425	0.01836	0.01498
CLK4	0.89025	0.00569	0.00307	0.3443	0.49845	0.00153	0.00121
CMBL	1.0807	0.00337	0.00159	0.32032	-0.015	0.00252	0.00202
CNOT6L	1.25361	0.00244	0.00102	-0.0182	-0.5428	0.00066	0.00067
CNOT7	1.11913	0.00099	0.00046	0.2871	-0.023	0.00129	0.00105
COG5	0.61575	0.00209	0.00137	0.09846	0.12964	0.00075	0.0007
COL4A3BP	1.0887	0.00145	0.00068	-0.178	-0.0279	0.00064	0.00073
CRYBG3	1.10968	0.00221	0.00102	0.5168	-0.1179	0.00135	0.00095
CRYL1	1.87464	0.00918	0.0025	0.56733	-0.1398	0.00257	0.00174
CSRP2	1.09479	0.00267	0.00125	0.49033	-0.5567	0.00159	0.00113
CYB5R4	1.25327	0.00569	0.00239	0.10608	-0.0292	0.00098	0.00091
CYLD	0.91505	0.00558	0.00296	0.54935	-0.3707	0.00128	0.00088
CYP2J6	0.94858	0.00198	0.00102	0.49032	-0.0406	0.00082	0.00058
10WSU102	0.61483	0.00244	0.00159	0.15325	-0.4103	0.0015	0.00135
3ERTD751	0.83798	0.00244	0.00137	0.51079	-0.1686	0.00113	0.00079
30003M21F	0.8692	0.02109	0.01155	-0.5506	0.09889	0.01445	0.02116
DCT	2.58745	0.00581	0.00097	0.01194	0.87561	0.00338	0.00336
DEPDC1B	1.10207	0.00122	0.00057	0.19481	-0.002	0.00089	0.00077
DERL2	0.7	0.00407	0.0025	0.10622	-0.2323	0.00219	0.00204
DHCR7	1.29347	0.00418	0.00171	0.43267	0.06148	0.00255	0.00189
DHTKD1	0.63118	0.00529	0.00341	0.47932	0.08616	0.00338	0.00243
DISP2	1.11069	0.02801	0.01297	0.4472	0.5476	0.02905	0.02131
EIF2B3	0.6628	0.0036	0.00228	0.57758	-0.1507	0.00183	0.00123
EIF3E	0.81421	0.0036	0.00205	0.41117	-0.0094	0.00087	0.00066
ELOVL7	1.84668	0.00511	0.00142	0.57267	-0.3728	0.0008	0.00054
ENGASE	1.11549	0.03562	0.01644	0.30759	-0.2698	0.01823	0.01473
EOGT	0.61483	0.00244	0.00159	0.51903	0.40892	0.00266	0.00185
EPB41L4B	1.03909	0.05973	0.02907	-0.3791	-0.2504	0.01033	0.01344
EPHA7	1.96973	0.00401	0.00102	0.37152	1.01969	0.00061	0.00047
ERI2	0.83798	0.00081	0.00046	-0.7328	-0.2139	0.00034	0.00057
ERLEC1	1.35226	0.00116	0.00046	0.18796	-0.4033	0.00083	0.00073
ETFA	1.46561	0.00848	0.00307	0.49301	-0.0306	0.00119	0.00084
ETL4	0.75336	0.00767	0.00455	0.43575	-0.2781	0.00271	0.002
EXOC5	2.53119	0.00198	0.00034	-0.279	-0.2628	0.00045	0.00055
EZH2	0.71947	0.00169	0.00102	0.2544	-0.4644	0.00105	0.00088
F8	1.03148	0.00023	0.00011	0.01195	0.74256	0.00051	0.00051
FAM135A	1.00992	0.00389	0.00193	0.45464	0.02392	0.00104	0.00076
FANCB	2.35963	0.00058	0.00011	-0.037	-0.2048	0.00044	0.00045
FANCL	1.41651	0.00273	0.00102	0.44364	-0.2279	0.00101	0.00074
FANCM	1.4289	0.00337	0.00125	0.2389	-0.2107	0.00083	0.0007
FBXO11	0.68222	0.00256	0.00159	0.05583	-0.0958	0.00109	0.00105
FBXO48	0.82416	0.00302	0.00171	0.5382	0.51376	0.00277	0.00191
FMO5	1.12256	0.00471	0.00216	0.38896	-0.173	0.00557	0.00425
FRMD4B	2.4019	0.01743	0.0033	0.1549	-0.2175	0.00939	0.00843
GAN	0.6166	0.00227	0.00148	0.2282	-0.5605	0.00182	0.00155
GFM2	0.9225	0.00151	0.0008	0.41829	-0.5363	0.00154	0.00115
GFOD1	0.85467	0.00267	0.00148	-0.1636	0.09138	0.00109	0.00122

GGT7	1.19501	0.0939	0.04101	0.44484	-0.0301	0.03135	0.02303
GHR	0.78408	0.005	0.0029	-0.1023	0.49523	0.00092	0.00099
GJB1	3.62163	0.0014	0.00011	0.52831	0.68368	0.00181	0.00125
GM14410	1.03136	0.00186	0.00091	-0.1918	0.24626	0.00053	0.0006
GMFB	1.478	0.00349	0.00125	0.28236	-0.4862	0.00062	0.00051
GTPBP4	1.02987	0.00163	0.0008	0.36726	-0.2142	0.00071	0.00055
GUCY2G	0.72023	0.00291	0.00176	0.31851	0.4446	0.01621	0.013
HAO1	1.54979	0.0025	0.00085	0.4129	0.75122	0.00142	0.00107
HAT1	0.59266	0.0036	0.00239	0.16952	-0.2729	0.00138	0.00123
HEATR1	0.73685	0.00721	0.00432	0.32416	-0.1347	0.001	0.0008
HERC3	1.478	0.00349	0.00125	0.4712	-0.3392	0.00119	0.00086
HINFP	0.58977	0.00325	0.00216	0.10854	-0.5401	0.00199	0.00184
IFT74	1.29546	0.0014	0.00057	0.39765	-0.255	0.00061	0.00046
IKBKG	2.6746	0.00291	0.00046	0.55912	-0.1619	0.00086	0.00059
IMMP1L	1.49033	0.00256	0.00091	0.24488	0.38086	0.001	0.00084
IMPA1	0.72473	0.0032	0.00193	-0.1449	-0.0802	0.00152	0.00168
ISOC1	1.33068	0.00372	0.00148	0.49033	-0.1675	0.00229	0.00163
ITFG1	0.61514	0.00105	0.00068	0.12872	0.62778	0.00082	0.00075
ITSN2	0.6146	0.0007	0.00046	0.02039	-0.0018	0.0008	0.00079
KEG1	1.09996	0.00244	0.00114	0.19428	-0.297	0.00162	0.00142
KLHL1	1.53119	0.00198	0.00068	0.17017	0.28132	0.00032	0.00028
KMT2C	1.19187	0.00221	0.00097	0.36758	0.34616	0.00097	0.00075
KRR1	1.73061	0.00151	0.00046	0.41533	-0.3085	0.00104	0.00078
LCORL	0.73652	0.0036	0.00216	0.35157	-0.059	0.00074	0.00058
LNPEP	1.40561	0.00407	0.00154	0.34049	-0.0188	0.00075	0.00059
LRRC69	1.3098	0.00198	0.0008	0.37588	0.15129	0.00401	0.00309
LRRCC1	1.67915	0.00401	0.00125	0.52819	0.23365	0.00129	0.0009
LSM11	0.66786	0.00488	0.00307	0.24234	-0.0631	0.0023	0.00195
LYSMD3	1.61514	0.00105	0.00034	-0.8374	-0.5602	0.00057	0.00102
MAD2L1	1.03041	0.00511	0.0025	0.46472	0.79048	0.00296	0.00215
MALT1	0.95251	0.00418	0.00216	-0.1404	-0.4944	0.00087	0.00096
MAN1A	1.76553	0.00116	0.00034	0.42604	0.14833	0.0006	0.00044
MAP3K2	0.86649	0.00291	0.00159	-0.0918	-0.4078	0.00075	0.0008
MBLAC2	1.79704	0.00198	0.00057	0.47247	0.60427	0.00092	0.00066
MBTPS2	1.83271	0.00081	0.00023	-0.2742	0.23812	0.00023	0.00028
MCU	1.64534	0.00285	0.00091	0.07294	-0.4951	0.00085	0.00081
MIPEP	0.94975	0.01209	0.00626	0.38402	-0.1152	0.00161	0.00123
MIS18BP1	1.25126	0.00081	0.00034	0.00169	-0.4276	0.00043	0.00043
MOB3B	1.39867	0.0018	0.00068	0.35257	-0.0822	0.00109	0.00085
MOSPD1	1.03136	0.00093	0.00046	0.44495	-0.1443	0.00064	0.00047
MTMR1	1.29546	0.0014	0.00057	0.27187	-0.0064	0.0005	0.00041
MYCBP2	0.61596	0.00157	0.00102	0.29361	-0.0147	0.00054	0.00044
MYOM3	1.03081	0.00442	0.00216	0.13436	-0.3501	0.00306	0.00279
N4BP2L2	1.12347	0.00372	0.00171	0.49847	-0.2286	0.00173	0.00122
NCKAP1L	0.72237	0.00366	0.00222	-0.1846	0.31885	0.00559	0.00635
NCMAP	1.40194	0.0251	0.0095	0.07412	-0.5192	0.00737	0.007
NDFIP2	0.85467	0.00267	0.00148	0.56828	-0.0895	0.00124	0.00084
NEDD1	0.6628	0.0036	0.00228	0.08915	-0.4443	0.00093	0.00088

NID2	0.6969	0.00424	0.00262	0.32244	-0.1503	0.00293	0.00234
NKAIN4	0.77838	0.02859	0.01667	-0.4961	0.3883	0.01076	0.01517
NLRC4	0.84698	0.00471	0.00262	0.34936	-0.5663	0.00472	0.0037
NPAT	0.9225	0.00151	0.0008	0.16805	-0.4412	0.0006	0.00053
NPHP3	1.17332	0.01296	0.00575	0.53	-0.5163	0.00159	0.0011
NUP155	0.95604	0.00221	0.00114	0.24335	-0.373	0.00086	0.00072
NUP160	0.98473	0.0036	0.00182	0.55193	-0.3254	0.00144	0.00098
PAG1	1.44751	0.01737	0.00637	0.38757	-0.2556	0.00346	0.00264
PAPOLG	4.35756	0.00232	0.00011	0.15991	-0.525	0.00075	0.00067
PCNP	1.43554	0.00831	0.00307	-0.5055	-0.2616	0.00098	0.00139
PDZK1IP1	0.99407	0.03399	0.01707	0.46399	0.54251	0.03213	0.02329
PEX2	0.7408	0.00209	0.00125	0.24635	-0.1928	0.00078	0.00066
PGAP1	1.54415	0.00232	0.0008	0.52411	-0.4304	0.00104	0.00072
PICALM	0.89235	0.00232	0.00125	-0.3985	-0.3067	0.00044	0.00059
PIK3C2A	0.9036	0.00128	0.00068	-0.1231	-0.3953	0.00053	0.00057
PKHD1L1	3.03072	0.00372	0.00046	0.4335	0.67843	0.00076	0.00056
PLSCR4	1.18292	0.00116	0.00051	0.12092	-0.1866	0.00065	0.0006
POLR3G	1.79704	0.00198	0.00057	-0.004	-0.4346	0.00077	0.00077
POT1A	2.32685	0.00343	0.00068	0.12538	-0.0959	0.00092	0.00085
PPAT	0.72881	0.005	0.00302	0.03099	-0.3587	0.00102	0.001
PPM1L	0.60073	0.00552	0.00364	-0.5823	-0.1855	0.00164	0.00246
PPP1R14A	0.71205	0.02022	0.01234	0.41849	0.57737	0.01199	0.00897
PRDM2	1.35226	0.00116	0.00046	0.0036	-0.4168	0.00088	0.00087
PRKCE	1.03093	0.00558	0.00273	-0.1706	-0.0968	0.00191	0.00216
PTER	0.70822	0.00279	0.00171	0.58189	0.68526	0.0013	0.00087
PTPN13	1.42954	0.01011	0.00375	0.36361	-0.0565	0.00185	0.00143
PUM2	0.65222	0.00232	0.00148	-0.1119	0.12445	0.00047	0.00051
QSER1	0.658	0.00198	0.00125	0.1181	0.40967	0.00115	0.00106
RABL3	0.89235	0.00232	0.00125	0.15415	-0.4553	0.00131	0.00118
RAD51B	0.64425	0.00302	0.00193	0.22045	0.24276	0.00116	0.00099
RAD54L	1.41626	0.00198	0.00074	-0.2423	-0.5254	0.00125	0.00148
RALGAPA1	0.93074	0.00163	0.00085	0.15046	-0.1966	0.00062	0.00056
RASA1	2.61105	0.0014	0.00023	0.4369	-0.3683	0.00051	0.00038
RB1	0.69406	0.00221	0.00137	0.17657	0.20968	0.00083	0.00074
RBM25	1.73061	0.00151	0.00046	-0.4841	-0.4691	0.00034	0.00047
RBM41	1.34905	0.00058	0.00023	0.18248	-0.4714	0.00038	0.00034
RBPJ	1.60933	0.0007	0.00023	-0.1537	-0.0196	0.00133	0.00148
RC3H2	1.0296	0.0014	0.00068	-0.264	-0.2366	0.00064	0.00077
RCBTB1	1.09479	0.00267	0.00125	0.31496	-0.4354	0.00119	0.00096
RNPC3	0.68222	0.00256	0.00159	0.3065	-0.1941	0.00064	0.00052
RORB	0.8559	0.0036	0.00199	0.1096	0.32946	0.00105	0.00098
ROS1	0.71049	0.00093	0.00057	-0.6016	0.22976	0.00117	0.00177
RPE	0.88099	0.00105	0.00057	0.17474	1.15157	0.00077	0.00068
RPS6KA3	1.03136	0.00093	0.00046	0.00391	0.09092	0.00016	0.00016
RSBN1L	0.66864	0.00163	0.00102	0.44337	-0.0557	0.00122	0.00089
RSRC1	0.72979	0.00802	0.00484	-0.057	-0.0567	0.00085	0.00088
RTN4IP1	2.14389	0.00151	0.00034	0.26303	-0.2268	0.00112	0.00093
RWDD1	1.47091	0.00221	0.0008	0.31419	-0.0466	0.00042	0.00034

SASH1	0.95706	0.0011	0.00057	-0.1047	-0.2068	0.00082	0.00088
SBF2	0.81338	0.005	0.00284	0.18182	0.37864	0.00088	0.00078
SCFD1	0.83798	0.00081	0.00046	-0.2689	-0.3728	0.00044	0.00053
SCLT1	0.83798	0.00244	0.00137	-0.089	0.20982	0.0008	0.00085
SEC24A	1.0807	0.00337	0.00159	0.40204	-0.3819	0.00152	0.00115
SENP8	1.43229	0.0043	0.00159	0.49252	-0.2088	0.00132	0.00094
SERPIND1	1.5547	0.00267	0.00091	0.36502	-0.0519	0.00166	0.00129
SGK2	0.58689	0.00436	0.0029	0.29125	-0.5687	0.00459	0.00375
SH3BGRL	2.03666	0.00047	0.00011	-0.3	-0.1403	0.00018	0.00022
SHMT1	0.59068	0.00506	0.00336	0.36001	-0.0181	0.00305	0.00238
SKA1	0.61514	0.00105	0.00068	-0.7237	-0.3074	0.00089	0.00147
SLC22A18	0.85137	0.01098	0.00609	-0.3753	0.56696	0.0152	0.01972
SLC35D2	1.10207	0.00122	0.00057	-0.1703	-0.3032	0.00092	0.00103
SLC44A1	1.03139	0.00116	0.00057	0.22413	-0.1587	0.00095	0.00081
SMARCA5	0.97711	0.00302	0.00154	0.27759	-0.2758	0.00095	0.00079
SNX20	0.62265	0.00604	0.00393	0.47248	-0.009	0.00952	0.00686
SNX25	0.93203	0.01151	0.00603	0.39088	-0.1448	0.00176	0.00134
SORBS1	0.9294	0.00877	0.00461	0.44902	-0.156	0.00297	0.00218
SPOPL	0.93444	0.00674	0.00353	0.15784	-0.1973	0.0008	0.00072
SPTSSB	0.62712	0.00721	0.00467	-0.2339	0.3519	0.00275	0.00323
SRBD1	1.08964	0.00291	0.00137	0.24965	0.16943	0.0012	0.00101
STAG1	1.16163	0.00267	0.0012	0.28033	0.04817	0.00047	0.00039
STK3	0.67692	0.00418	0.00262	-0.0909	0.4151	0.00098	0.00104
SUCLG2	1.83692	0.00407	0.00114	0.49331	0.04845	0.00128	0.00091
SUGT1	1.0887	0.00145	0.00068	0.29022	0.19823	0.00074	0.0006
SUSD1	2.03072	0.00372	0.00091	0.40551	-0.068	0.00129	0.00097
SYNJ2BP	0.79493	0.00198	0.00114	-0.0922	0.19494	0.00077	0.00082
TAB3	1.35226	0.00116	0.00046	0.12412	-0.4816	0.00018	0.00017
TATDN1	0.83723	0.00285	0.00159	0.53418	-0.4329	0.00166	0.00114
TBC1D8B	3.35756	0.00116	0.00011	0.50693	-0.3316	0.00032	0.00023
TBL1X	0.94858	0.00198	0.00102	-0.2913	0.13518	0.00034	0.00041
TEAD4	0.9832	0.00697	0.00353	0.5629	-0.1704	0.00279	0.00189
TERT	2.57593	0.0061	0.00102	-0.0245	-0.225	0.00234	0.00238
TEX30	2.19659	0.00105	0.00023	0.5157	1.08116	0.00092	0.00065
TFRC	0.9117	0.00407	0.00216	0.54278	-0.4264	0.00127	0.00087
TGFA	1.2404	0.0043	0.00182	-0.0862	-0.5082	0.002	0.00212
TIMM21	0.63958	0.00709	0.00455	-0.5381	-0.3271	0.0019	0.00275
TLE1	1.32099	0.00256	0.00102	0.57134	-0.0032	0.00074	0.0005
TLN2	0.80822	0.0014	0.0008	-0.7395	0.35266	0.00181	0.00302
TM4SF20	0.70838	0.00186	0.00114	0.34482	-0.4456	0.00136	0.00107
TMCC1	0.76729	0.00232	0.00137	0.31108	-0.0648	0.00133	0.00107
TMEM161B	2.09671	0.00267	0.00063	0.19315	-0.4683	0.00067	0.00058
TMEM30A	0.95088	0.00308	0.00159	0.5157	-0.3167	0.0009	0.00063
TMEM97	0.89025	0.00569	0.00307	0.07128	-0.1219	0.00281	0.00268
TMLHE	0.70627	0.00047	0.00029	0.34304	0.62923	0.00015	0.00012
TMPO	1.39867	0.0018	0.00068	0.56851	0.03438	0.0013	0.00088
TMTC3	0.61514	0.00105	0.00068	0.38978	0.39398	0.00047	0.00036
TRPM2	1.48373	0.01273	0.00455	-1.578	-0.342	0.0052	0.01552

TSHZ2	0.91061	0.00535	0.00284	0.42577	-0.0807	0.0032	0.00238
TSPAN6	1.25126	0.00081	0.00034	#DIV/0!	-0.2344	0.00047	0
UGP2	0.79493	0.00198	0.00114	0.47811	0.29133	0.00104	0.00075
USP14	1.00325	0.00296	0.00148	-0.5782	-0.2892	0.00076	0.00114
USP24	0.89235	0.00232	0.00125	0.22456	0.11462	0.00089	0.00076
UTP20	2.51711	0.00325	0.00057	0.03703	-0.5779	0.00097	0.00094
VPS13B	1.24517	0.00337	0.00142	0.19053	0.07866	0.00104	0.00092
VWCE	2.51597	0.05206	0.0091	0.48783	0.2955	0.0519	0.03701
WASL	0.73141	0.00302	0.00182	0.34241	-0.3078	0.00119	0.00094
WDR20	1.53356	0.00395	0.00137	0.40105	-0.4665	0.00139	0.00106
WDR55	1.24517	0.00337	0.00142	0.35244	-0.5618	0.0025	0.00196
WDR73	0.9592	0.00343	0.00176	0.28127	-0.5509	0.00228	0.00188
WRN	1.31102	0.00099	0.0004	0.07561	0.11634	0.0009	0.00086
XRN1	2.38803	0.00238	0.00046	0.28248	-0.1788	0.00078	0.00064
YOD1	1.70328	0.00593	0.00182	0.27793	1.39366	0.0014	0.00115
YTHDC2	0.61584	0.00366	0.00239	0.45976	-0.5453	0.0008	0.00058
YTHDF3	1.4893	0.00447	0.00159	-0.1145	-0.4165	0.00068	0.00074
ZBTB32	2.67371	0.00726	0.00114	0.57808	0.24216	0.00431	0.00288
ZBTB37	0.64841	0.00384	0.00245	0.1003	0.39908	0.00114	0.00106
ZCCHC4	1.59738	0.00447	0.00148	-0.1716	-0.5368	0.00132	0.00148
ZFP109	1.13373	0.00674	0.00307	0.18982	-0.3536	0.00238	0.00209
ZFP169	0.7383	0.0018	0.00108	0.29032	-0.0279	0.00107	0.00087
ZFP442	1.09996	0.00244	0.00114	0.00284	0.43448	0.00073	0.00073
ZFP53	1.12347	0.00372	0.00171	0.47838	0.19692	0.00122	0.00087
ZFP661	0.98157	0.00674	0.00341	0.47223	-0.3204	0.00606	0.00437
ZFP667	0.94646	0.00384	0.00199	0.3731	0.1909	0.00116	0.0009
ZFP729A	1.22325	0.00186	0.0008	0.39399	-0.3436	0.00091	0.00069
ZFP748	1.32099	0.00256	0.00102	-0.2694	0.29891	0.00062	0.00075
ZFP809	0.6628	0.0036	0.00228	0.42537	-0.1344	0.0014	0.00104
ZFP932	1.46308	0.00314	0.00114	0.35197	-0.4805	0.00138	0.00108
ZMYM2	1.22325	0.00186	0.0008	-0.1141	-0.4969	0.00057	0.00061
ZRANB1	1.6746	0.00291	0.00091	0.22746	-0.2944	0.00059	0.00051
ZZEF1	0.83798	0.00325	0.00182	0.53752	-0.2111	0.00184	0.00127

GB_K36_KO	GB_K36_WT	Pvalue	logFC_KO/WT	KO_1	KO_2	KO_3	WT_1	WT_2	WT_3
0.00507	0.00466	5.70E-33	-2.060038	200	219	209.5	939	940	939.5
0.00365	0.00207	0.030237	-0.840666	65	55	60	104	127	115.5
0.00608	0.00675	0.001555	-1.218642	48	59	53.5	127	142	134.5
0.00621	0.00711	5.56E-151	-5.138349	48	45	46.5	1619	1905	1762
0.0018	0.00113	0.036786	-1.293971	14	28	21	55	57	56
0.00289	0.00166	0.027531	-1.332082	19	21	20	46	63	54.5
0.00208	0.00183	3.96E-08	-8.550856	0	0	0	75	73	74
0.00346	0.00315	0.005566	-1.64323	11	39	25	76	96	86
0.00578	0.00335	0.000411	-1.156778	89	67	78	194	177	185.5
0.00312	0.00198	0.008843	-5.685805	0	0	0	14	6	10
0.00636	0.00516	0.003966	-3.88629	3	0	1.5	31	15	23
0.0036	0.00442	3.25E-23	-0.971601	1187	1106	1147	2257	2579	2418
0.00758	0.0083	2.39E-20	-2.015654	136	107	121.5	490	564	527
0.00473	0.00607	2.28E-24	-1.202833	914	1003	958.5	2135	2634	2385
0.00414	0.0059	2.66E-17	-1.689224	170	157	163.5	552	580	566
0.00805	0.01132	2.99E-22	-4.808669	9	6	7.5	223	227	225
0.00762	0.00655	9.78E-07	-5.88848	2	0	1	53	74	63.5
0.00614	0.00618	7.56E-08	-8.429667	0	0	0	58	79	68.5
0.0018	0.0024	0.00038	-1.330959	50	62	56	161	142	151.5
0.00332	0.00355	0.003364	-0.5995	250	242	246	378	424	401
0.00228	0.00177	0.00032	-6.644881	0	0	0	14	26	20
0.00536	0.00531	2.31E-16	-1.034708	1057	757	907	1833	2145	1989
0.00099	0.00097	5.55E-05	-0.99729	155	130	142.5	281	330	305.5
0.00185	0.0015	2.69E-15	-1.693883	151	187	169	527	657	592
0.0042	0.00365	2.22E-13	-10.78076	0	0	0	296	403	349.5
0.00603	0.00672	1.01E-07	-8.368234	0	0	0	59	72	65.5
0.01313	0.0189	0.001617	-0.996349	85	87	86	168	202	185
0.00208	0.00267	3.76E-08	-1.313395	143	141	142	328	434	381
0.00573	0.00353	2.85E-05	-0.774179	383	275	329	565	639	602
0.00433	0.00557	0.007203	-2.050051	10	8	9	37	43	40
0.0082	0.00136	9.04E-09	-8.478766	0	1	0.5	215	166	190.5
0.00303	0.00252	5.32E-12	-4.913936	4	3	3.5	101	126	113.5
0.0023	0.00182	8.46E-10	-9.304336	0	0	0	97	155	126
0.0012	0.00103	4.36E-10	-1.238851	229	196	212.5	479	600	539.5
0.01627	0.01807	7.74E-05	-1.293439	81	63	72	169	210	189.5
0.00088	0.00086	0.001474	-4.529929	0	2	1	17	34	25.5
0.01987	0.02981	2.87E-06	-1.751338	49	39	44	158	159	158.5
0.00381	0.00452	2.25E-20	-1.049279	855	779	817	1820	1805	1813
0.00285	0.0031	2.05E-11	-0.929826	551	555	553	1027	1245	1136
0.00103	0.00097	6.06E-11	-1.908092	67	79	73	307	282	294.5
0.00452	0.0058	7.71E-80	-1.672444	1451	1177	1314	4261	4719	4490
0.00356	0.00296	5.46E-17	-1.117775	530	541	535.5	1215	1285	1250
0.00573	0.00713	3.74E-53	-3.402357	77	54	65.5	711	772	741.5
0.00175	0.00244	3.21E-13	-1.515474	173	213	193	542	652	597

0.00477	0.00657	2.86E-07	-0.682373	1031	732	881.5	1439	1585	1512
0.00364	0.00461	0.00014	-0.620041	406	385	395.5	621	686	653.5
0.00339	0.00203	0.032997	-2.005417	5	7	6	26	26	26
0.00405	0.00378	5.09E-20	-1.570291	303	226	264.5	796	886	841
0.00322	0.00439	6.34E-17	-1.119653	515	475	495	1105	1206	1156
0.00475	0.00454	1.71E-10	-7.061891	2	0	1	149	136	142.5
0.00596	0.00515	2.03E-35	-1.882829	336	263	299.5	1174	1189	1182
0.00393	0.00246	1.36E-09	-1.288923	192	174	183	501	456	478.5
0.01012	0.00725	8.56E-06	-0.727549	411	441	426	690	833	761.5
0.00618	0.00577	7.01E-07	-0.710734	589	512	550.5	981	948	964.5
0.00237	0.00317	0.009517	-3.999896	0	2	1	17	18	17.5
0.00515	0.00331	1.46E-05	-0.628244	526	492	509	819	871	845
0.00324	0.00276	3.12E-10	-1.833912	69	109	89	318	371	344.5
0.0033	0.00144	0.00195	-0.827605	173	124	148.5	301	260	280.5
0.00257	0.00347	2.09E-232	-5.574147	67	47	57	2718	3113	2916
0.00654	0.00383	0.000275	-0.732428	245	251	248	437	449	443
0.00529	0.00595	1.82E-22	-1.016635	1508	1398	1453	2740	3601	3171
0.00181	0.00216	0.000253	-0.616467	398	434	416	630	747	688.5
0.01172	0.01198	0.002498	-0.907937	94	119	106.5	205	227	216
0.00619	0.00851	2.45E-42	-1.338692	1038	933	985.5	2629	2718	2674
0.00399	0.00313	0.002116	-2.100736	12	10	11	51	50	50.5
0.00282	0.00131	2.00E-13	-6.094167	1	3	2	135	160	147.5
0.00661	0.0047	0.000591	-1.394693	58	35	46.5	122	139	130.5
0.01285	0.01694	2.86E-40	-4.693795	19	12	15.5	418	441	429.5
0.00332	0.00329	5.73E-12	-1.99357	78	58	68	275	305	290
0.00336	0.00339	3.88E-09	-0.885947	445	399	422	809	865	837
0.00745	0.00936	9.77E-76	-1.183623	4688	4185	4437	10056	11607	10832
0.01023	0.00813	0.000214	-6.746808	0	0	0	26	16	21
0.00745	0.00938	0.000988	-0.622844	330	307	318.5	553	497	525
0.00325	0.002	0.004004	-1.455749	59	18	38.5	115	107	111
0.00426	0.00357	0.012433	-1.491106	33	12	22.5	62	72	67
0.00856	0.00793	3.48E-15	-0.91068	863	899	881	1669	1901	1785
0.00887	0.00859	9.05E-17	-0.76663	2385	2590	2488	4471	4644	4558
0.00186	0.00163	0.000613	-3.326065	6	1	3.5	38	36	37
0.00385	0.0031	6.84E-17	-2.761704	34	41	37.5	243	307	275
0.0038	0.00365	0.001738	-0.611033	275	298	286.5	452	491	471.5
0.0027	0.00197	1.38E-11	-1.420738	167	191	179	501	532	516.5
0.0041	0.00416	0.000652	-2.2697	13	9	11	48	66	57
0.00352	0.00261	9.67E-08	-4.729944	0	5	2.5	65	80	72.5
0.0075	0.00923	7.97E-20	-0.894706	1372	1266	1319	2389	2891	2640
0.0045	0.00524	2.10E-07	-8.227067	0	0	0	51	68	59.5
0.00227	0.00281	1.72E-10	-1.113341	349	265	307	667	756	711.5
0.00431	0.00236	6.77E-13	-1.572468	162	206	184	621	557	589
0.00234	0.00205	1.54E-05	-0.85669	249	268	258.5	454	557	505.5
0.00121	0.00148	8.18E-07	-1.105689	168	203	185.5	412	450	431
0.00478	0.00502	1.58E-07	-0.890754	363	381	372	657	833	745
0.00242	0.00092	2.94E-11	-2.928561	119	205	162	1237	1453	1345
0.00493	0.00701	2.04E-34	-2.72601	111	73	92	604	699	651.5

0.00753	0.00636	0.042716	-2.419104	0	8	4	17	31	24
0.0033	0.0015	4.83E-06	-1.279745	153	88	120.5	289	335	312
0.00171	0.00223	1.05E-05	-0.631093	756	557	656.5	1063	1110	1087
0.00294	0.00272	6.22E-06	-7.529639	0	0	0	36	37	36.5
0.02742	0.02999	0.012533	-5.560502	0	0	0	5	14	9.5
0.00214	0.00257	1.88E-12	-10.39539	0	0	0	240	294	267
0.00144	0.00171	0.003997	-5.944977	0	0	0	16	8	12
0.00617	0.00578	1.65E-07	-1.215939	190	133	161.5	384	418	401
0.0069	0.0084	0.003942	-4.220354	1	1	1	20	20	20
0.01339	0.00837	0.000819	-0.672259	270	326	298	511	513	512
0.00249	0.00283	9.62E-05	-1.417521	49	65	57	160	169	164.5
0.0028	0.00405	6.40E-08	-0.703773	811	719	765	1171	1513	1342
0.00735	0.00709	3.07E-11	-2.50274	64	23	43.5	252	270	261
0.00414	0.00493	1.64E-08	-0.797457	524	493	508.5	928	970	949
0.00466	0.00604	4.91E-12	-1.199062	290	257	273.5	659	688	673.5
0.00972	0.00295	0.001878	-6.156297	0	0	0	16	12	14
0.00159	0.00161	2.19E-21	-0.988522	1086	935	1011	1999	2308	2154
0.0041	0.00372	1.25E-10	-0.887216	571	488	529.5	1032	1067	1050
0.0055	0.00328	4.48E-05	-4.767186	3	0	1.5	24	64	44
0.00194	0.0017	0.000956	-6.340613	0	0	0	16	16	16
0.00204	0.00147	3.55E-06	-7.675676	0	0	0	49	31	40
0.00166	0.00086	1.66E-06	-4.412551	4	1	2.5	47	67	57
0.00618	0.00819	4.01E-07	-8.099662	0	0	0	46	63	54.5
0.00624	0.00746	2.00E-09	-0.69253	923	819	871	1496	1522	1509
0.01017	0.0128	2.09E-06	-1.516281	72	62	67	188	224	206
0.00664	0.00723	3.91E-07	-1.231559	136	138	137	311	383	347
0.00159	0.0019	7.55E-12	-2.659561	43	23	33	253	188	220.5
0.00231	0.00179	1.34E-24	-1.327962	504	485	494.5	1280	1389	1335
0.00544	0.00591	4.87E-07	-0.990708	245	246	245.5	463	590	526.5
0.00693	0.00945	5.29E-25	-1.03752	1104	1038	1071	2190	2541	2366
0.004	0.00291	1.65E-06	-1.028104	363	214	288.5	554	703	628.5
0.00244	0.00255	6.79E-05	-0.983621	166	164	165	286	420	353
0.00611	0.00512	2.93E-10	-2.117499	51	44	47.5	192	252	222
0.00426	0.00541	6.00E-07	-0.662884	688	734	711	1191	1232	1212
0.01735	0.01749	0.001876	-0.8854	180	103	141.5	249	309	279
0.00641	0.00623	1.18E-20	-1.004175	951	967	959	1976	2165	2071
0.00564	0.00664	3.49E-24	-1.220753	913	1009	961	2145	2699	2422
0.0108	0.00999	4.85E-05	-7.084642	0	0	0	22	32	27
0.0071	0.00753	0.00071	-0.914124	131	112	121.5	238	253	245.5
0.00221	0.0021	2.93E-07	-1.107625	189	172	180.5	418	416	417
0.00425	0.00505	0.001119	-0.603322	311	291	301	467	516	491.5
0.00475	0.00345	1.89E-08	-0.651946	1041	969	1005	1738	1646	1692
0.00771	0.00971	0.038673	-0.585788	123	135	129	204	213	208.5
0.00214	0.00221	6.40E-23	-1.517199	318	324	321	928	1051	989.5
0.00418	0.00385	1.24E-07	-0.713979	652	580	616	982	1192	1087
0.00415	0.00364	3.33E-40	-1.295997	1074	1067	1071	2664	2995	2830
0.00469	0.00574	0.000281	-1.998191	16	22	19	92	71	81.5
0.00434	0.00418	7.87E-28	-2.441504	98	87	92.5	515	564	539.5

0.00466	0.00368	2.80E-06	-1.804657	36	77	56.5	221	208	214.5
0.00228	0.00213	0.000514	-0.662558	301	306	303.5	533	498	515.5
0.00765	0.00253	4.66E-05	-0.710389	346	312	329	542	615	578.5
0.01207	0.0102	7.73E-10	-5.433109	2	2	2	97	88	92.5
0.0141	0.00515	0.009027	-0.738152	139	112	125.5	235	212	223.5
0.00278	0.00315	4.06E-11	-0.731698	953	869	911	1525	1727	1626
0.00221	0.00221	6.36E-06	-0.768147	385	315	350	609	669	639
0.00267	0.00285	6.57E-08	-8.483575	0	0	0	51	92	71.5
0.00336	0.00262	0.000184	-0.872604	168	168	168	315	347	331
0.00331	0.00242	1.50E-15	-2.743164	34	33	33.5	221	262	241.5
0.00402	0.0019	0.000101	-1.251145	91	64	77.5	182	213	197.5
0.00333	0.00445	8.55E-15	-2.206113	61	73	67	342	323	332.5
0.00626	0.00831	8.77E-39	-8.018046	1	5	3	822	849	835.5
0.00248	0.00149	0.01554	-1.94764	7	10	8.5	33	38	35.5
0.00723	0.00852	4.71E-07	-2.840511	15	10	12.5	74	119	96.5
0.00553	0.00373	5.95E-22	-1.698922	310	226	268	968	887	927.5
0.00286	0.00385	1.40E-12	-1.395312	200	181	190.5	518	558	538
0.0049	0.00293	6.10E-06	-0.712592	482	410	446	798	766	782
0.00525	0.00583	0.005995	-2.395795	2	12	7	39	42	40.5
0.00485	0.00384	2.42E-16	-1.408938	274	238	256	710	748	729
0.00416	0.006	7.83E-06	-2.588145	18	9	13.5	70	104	87
0.0026	0.00174	1.56E-06	-7.823355	0	0	0	38	52	45
0.00689	0.00712	9.18E-08	-0.704605	701	755	728	1258	1296	1277
0.00224	0.00299	1.16E-07	-0.981816	263	269	266	539	592	565.5
0.00809	0.00943	0.000377	-6.585464	0	0	0	18	20	19
0.00134	0.0015	4.57E-18	-1.287653	572	429	500.5	1356	1250	1303
0.00269	0.00245	1.36E-15	-1.304948	385	288	336.5	833	948	890.5
0.00449	0.00452	0.029651	-1.325078	28	14	21	52	60	56
0.00511	0.00701	2.35E-36	-1.271756	1062	1097	1080	2725	2884	2805
0.00771	0.01093	2.20E-07	-8.216315	0	0	0	52	66	59
0.00286	0.0012	4.31E-14	-1.474986	219	197	208	651	586	618.5
0.01073	0.00838	3.03E-07	-0.857001	353	330	341.5	652	676	664
0.00454	0.00323	6.67E-39	-1.979019	340	263	301.5	1147	1405	1276
0.00248	0.00222	0.016533	-5.452012	0	0	0	12	5	8.5
0.00286	0.00167	4.66E-05	-1.191754	94	129	111.5	291	258	274.5
0.00515	0.00724	3.26E-08	-0.750977	623	673	648	1128	1222	1175
0.00359	0.00392	2.56E-05	-7.230177	0	0	0	33	26	29.5
0.00488	0.00433	0.000312	-0.644376	452	610	531	827	973	900
0.00364	0.00197	0.000188	-1.221652	113	62	87.5	200	235	217.5
0.0027	0.00209	0.000107	-1.127144	111	85	98	224	234	229
0.00508	0.00706	5.76E-08	-1.206095	188	164	176	460	408	434
0.00336	0.00337	0.019544	-1.599366	11	17	14	43	49	46
0.00616	0.00898	0.029211	-0.919356	54	46	50	108	94	101
0.00551	0.00471	0.03174	-4.361704	1	0	0.5	10	12	11
0.00161	0.00232	6.71E-14	-2.323946	56	45	50.5	273	268	270.5
0.00594	0.00758	0.016613	-3.00185	0	5	2.5	24	20	22
0.00352	0.00221	0.04759	-1.170298	25	20	22.5	46	63	54.5
0.01073	0.0138	0.01061	-5.597743	0	0	0	11	8	9.5

0.00566	0.00312	0.013612	-3.799141	22	1	11.5	147	185	166
0.00648	0.00629	0.00028	-1.408423	67	38	52.5	141	156	148.5
0.00198	0.00222	0.000221	-0.628184	368	371	369.5	577	653	615
0.01161	0.0069	0.000818	-3.883196	4	0	2	32	30	31
0.00462	0.00607	3.63E-10	-2.385627	32	35	33.5	157	222	189.5
0.00423	0.00309	6.11E-09	-1.0836	313	235	274	569	676	622.5
0.00454	0.00332	0.004408	-5.905933	0	0	0	8	16	12
0.00635	0.00802	3.47E-22	-0.699815	3251	2922	3087	5244	5514	5379
0.00336	0.00383	3.62E-05	-0.664377	443	381	412	696	703	699.5
0.00153	0.00137	5.04E-12	-2.659656	30	25	27.5	176	197	186.5
0.00519	0.00482	3.56E-08	-8.572433	0	0	0	67	84	75.5
0.01071	0.01498	0.033668	-1.716033	17	5	11	28	49	38.5
0.00756	0.00728	0.019927	-0.791954	136	70	103	169	211	190
0.00597	0.00857	8.43E-12	-1.112222	372	316	344	804	788	796
0.0044	0.00375	1.32E-08	-0.994249	317	367	342	692	779	735.5
0.00776	0.00578	1.81E-16	-1.318129	670	575	622.5	1892	1412	1652
0.00082	0.00042	3.50E-28	-8.253013	4	0	2	608	702	655
0.00327	0.00215	1.47E-12	-0.747994	1521	1232	1377	2227	2739	2483
0.00265	0.00096	9.38E-14	-1.75386	164	193	178.5	754	534	644
0.02416	0.0232	1.32E-16	-0.901998	915	884	899.5	1736	1878	1807
0.00251	0.00295	6.17E-17	-1.854907	138	116	127	441	547	494
0.00252	0.00329	5.12E-52	-2.806615	141	107	124	911	946	928.5
0.00544	0.00777	3.94E-05	-0.603319	602	480	541	829	933	881
0.00346	0.0044	4.45E-47	-2.523573	161	177	169	935	1164	1050
0.00578	0.00498	0.000377	-0.639384	378	296	337	542	582	562
0.00433	0.00364	2.88E-20	-1.410057	335	327	331	902	990	946
0.00345	0.00472	0.004622	-0.744594	138	175	156.5	281	285	283
0.00446	0.00391	0.007994	-0.601931	216	203	209.5	357	324	340.5
0.00473	0.00709	0.006514	-0.620108	269	185	227	337	410	373.5
0.00413	0.00435	3.41E-05	-1.769934	35	33	34	134	114	124
0.00883	0.0116	1.15E-298	-2.550953	2000	1887	1944	11319	13196	12258
0.00254	0.00213	2.48E-06	-2.756969	8	16	12	85	91	88
0.004	0.00537	0.00088	-0.662366	272	294	283	428	540	484
0.00611	0.0054	4.18E-93	-1.928963	1164	1083	1124	4699	4468	4584
0.0058	0.00478	0.000285	-1.810928	22	29	25.5	109	83	96
0.00185	0.0013	0.000197	-1.7912	20	46	33	133	115	124
0.00449	0.00338	1.82E-09	-3.968179	5	6	5.5	81	105	93
0.00317	0.00072	5.94E-06	-1.924135	30	33	31.5	130	127	128.5
0.00339	0.00258	0.012568	-1.813685	12	10	11	39	44	41.5
0.00369	0.00391	5.65E-09	-3.229039	8	14	11	122	100	111
0.00255	0.00169	0.016348	-5.431506	0	0	0	9	8	8.5
0.00241	0.00102	1.31E-09	-8.87317	1	0	0.5	213	293	253
0.00372	0.00388	6.91E-24	-2.043923	137	154	145.5	597	698	647.5
0.00269	0.0037	5.69E-07	-1.345363	136	91	113.5	284	333	308.5
0.00515	0.00451	0.029586	-2.707754	3	2	2.5	16	19	17.5
0.00968	0.01172	8.54E-07	-0.777686	423	371	397	698	763	730.5
0.001	0.00115	2.28E-121	-8.985616	6	9	7.5	3864	4309	4087
0.0014	0.00174	0.002278	-1.497898	28	29	28.5	74	100	87

0.00209	0.00303	0.005239	-1.234003	33	55	44	104	121	112.5
0.01215	0.00742	0.00815	-2.496465	3	7	5	34	27	30.5
0.00531	0.00628	2.87E-07	-1.233802	139	180	159.5	400	410	405
0.00359	0.0024	0.02775	-0.592673	180	128	154	219	279	249
0.00889	0.01087	2.10E-64	-2.067252	554	436	495	2229	2207	2218
0.00427	0.00535	3.79E-10	-0.920721	787	584	685.5	1187	1601	1394
0.00331	0.0022	0.014426	-5.490894	0	0	0	6	12	9
0.00743	0.00665	0.000603	-1.00503	100	152	126	268	280	274
0.00364	0.00425	2.21E-07	-1.007221	257	275	266	502	654	578
0.00537	0.00678	1.29E-06	-0.733451	533	466	499.5	785	1003	894
0.0058	0.00418	5.14E-14	-0.672575	1819	1798	1809	2908	3299	3104
0.00392	0.00521	1.82E-41	-3.141367	105	58	81.5	676	863	769.5
0.00469	0.00209	5.16E-19	-12.91073	0	0	0	1491	1552	1522
0.00348	0.00432	2.31E-17	-1.732893	178	181	179.5	687	591	639
0.00312	0.00388	9.19E-28	-2.405347	117	87	102	568	588	578
0.00383	0.00272	1.40E-14	-11.24757	0	0	0	445	518	481.5
0.00442	0.0041	0.003148	-1.552391	22	26	24	71	81	76
0.00384	0.00231	0.011811	-0.623089	162	182	172	277	294	285.5
0.00766	0.00862	1.60E-11	-0.785106	1141	1305	1223	2201	2345	2273
0.00669	0.00773	8.67E-28	-5.144231	13	3	8	284	320	302
0.00456	0.00361	0.005085	-1.258544	31	77	54	142	140	141
0.01124	0.01666	0.02393	-0.67578	110	118	114	205	186	195.5
0.00329	0.00474	9.30E-05	-3.042977	14	1	7.5	58	72	65
0.0032	0.00289	5.12E-07	-5.979189	2	0	1	60	75	67.5
0.00121	0.00093	1.44E-20	-8.065524	0	3	1.5	412	453	432.5
0.00524	0.0059	4.64E-06	-0.916414	260	208	234	458	488	473
0.00429	0.00469	1.51E-28	-1.457378	967	684	825.5	2120	2742	2431
0.00554	0.00626	0.000536	-0.677571	276	288	282	494	475	484.5
0.00215	0.0019	1.10E-18	-1.062381	775	654	714.5	1465	1741	1603
0.00197	0.00155	0.023813	-1.305476	18	28	23	62	61	61.5
0.00179	0.00177	0.000334	-2.185872	23	8	15.5	76	73	74.5
0.00597	0.00285	4.52E-10	-3.500559	9	9	9	105	114	109.5
0.00874	0.0065	1.15E-07	-1.209035	165	144	154.5	350	418	384
0.0111	0.00629	3.91E-06	-1.090607	155	141	148	324	353	338.5
0.00315	0.00299	1.09E-07	-8.352294	0	0	0	65	64	64.5
0.00438	0.00455	6.03E-08	-0.92402	380	308	344	632	770	701
0.01371	0.01513	0.021535	-5.325911	0	0	0	6	10	8
0.00245	0.00304	0.000182	-0.597461	500	405	452.5	669	801	735
0.00404	0.00453	4.00E-08	-1.215451	246	161	203.5	517	489	503
0.00689	0.0064	0.035709	-1.029571	29	45	37	76	88	82
0.00186	0.00205	8.04E-10	-1.006395	345	353	349	737	771	754
0.00375	0.00381	3.23E-09	-2.157248	42	35	38.5	181	187	184
0.00215	0.00286	0.000425	-0.752748	295	201	248	423	470	446.5
0.01099	0.01275	9.09E-13	-0.755955	1222	1005	1114	1954	2075	2015
0.00613	0.00539	5.80E-09	-8.919484	0	0	0	86	106	96
0.00465	0.00547	4.89E-05	-0.619988	530	609	569.5	888	1002	945
0.00298	0.00327	1.09E-05	-1.228676	100	103	101.5	258	253	255.5
0.00216	0.00223	3.15E-13	-1.310117	380	260	320	755	945	850

0.00446	0.00495	2.77E-05	-0.825805	285	224	254.5	454	513	483.5
0.00434	0.00265	1.11E-05	-0.780403	514	355	434.5	695	905	800
0.00224	0.00132	0.010267	-5.690211	0	0	0	3	18	10.5
0.00359	0.00286	0.000286	-0.928803	135	153	144	298	292	295
0.00385	0.00264	7.81E-09	-1.142303	217	207	212	469	538	503.5
0.00352	0.0039	1.21E-53	-1.787185	775	631	703	2301	2916	2609
0.00744	0.00557	0.002367	-0.644694	301	211	256	402	454	428
0.00259	0.00096	0.000645	-0.660683	373	270	321.5	555	529	542
0.00551	0.00639	0.023196	-4.508859	0	1	0.5	8	17	12.5
0.01055	0.01048	3.84E-12	-0.64326	1792	1693	1743	2658	3204	2931
0.00264	0.00332	3.83E-14	-1.382073	274	211	242.5	631	724	677.5
0.00492	0.00388	2.21E-07	-3.737303	6	4	5	66	77	71.5
0.0048	0.00506	2.02E-12	-4.625181	6	3	4.5	109	129	119
0.00191	0.00095	0.016599	-5.424628	0	0	0	8	9	8.5
0.00544	0.00288	0.000208	-6.738487	0	0	0	23	19	21
0.00675	0.00955	5.68E-06	-7.548489	0	0	0	36	38	37
0.00117	0.00048	3.98E-06	-4.359539	0	5	2.5	56	56	56
0.00267	0.00184	2.83E-05	-2.204271	16	20	18	86	93	89.5
0.00146	0.00185	8.66E-62	-2.36203	262	261	261.5	1344	1551	1448
0.00312	0.00427	0.000467	-1.090738	80	108	94	209	224	216.5
0.00324	0.00424	2.07E-15	-8.37762	2	0	1	346	367	356.5
0.00479	0.00382	1.23E-14	-1.048427	615	536	575.5	1314	1233	1274
0.00417	0.00337	3.16E-05	-1.245608	103	76	89.5	207	248	227.5
0.00257	0.00286	9.70E-05	-1.473922	63	40	51.5	151	154	152.5
0.00451	0.00412	1.30E-09	-0.735729	916	775	845.5	1529	1484	1507
0.00519	0.005	0.016599	-5.424628	0	0	0	8	9	8.5
0.001	0.00071	0.003113	-0.789506	131	172	151.5	276	290	283
0.00312	0.00308	0.000743	-6.444547	0	0	0	10	25	17.5
0.00371	0.00395	0.00032	-6.630504	0	0	0	21	18	19.5
0.0064	0.00463	1.30E-11	-1.005187	445	443	444	960	954	957
0.00443	0.00432	0.001642	-0.699523	244	242	243	468	376	422
0.00869	0.00786	1.97E-31	-0.884608	3469	2852	3161	5929	6583	6256
0.01017	0.00985	0.021696	-1.020089	37	104	70.5	145	169	157
0.00125	0.00178	6.63E-05	-7.007303	0	0	0	23	28	25.5
0.00309	0.00394	0.001111	-0.625153	472	411	441.5	834	616	725
0.00964	0.01138	4.29E-05	-0.850701	221	223	222	432	428	430
0.00422	0.00466	0.015094	-0.695783	156	104	130	211	239	225
0.01398	0.01669	2.12E-08	-1.457348	120	97	108.5	326	311	318.5
0.01448	0.00875	7.66E-11	-7.145033	0	2	1	159	145	152
0.00277	0.00148	0.000158	-6.805348	0	0	0	24	20	22
0.0021	0.00177	7.79E-19	-12.84605	0	0	0	1409	1502	1456
0.00067	0.00049	2.23E-11	-9.953585	0	0	0	202	189	195.5
0.00094	0.00052	0.000163	-6.794726	0	0	0	20	24	22
0.00639	0.00626	0.000479	-2.051722	22	11	16.5	77	68	72.5
0.00763	0.00956	0.00168	-4.512047	2	0	1	27	21	24
0.0042	0.00486	4.84E-22	-1.425666	370	332	351	955	1071	1013
0.00534	0.00614	0.010556	-0.719995	122	118	120	204	221	212.5
0.00478	0.00423	7.11E-05	-2.112512	22	15	18.5	83	88	85.5

0.01242	0.0098	5.39E-49	-1.189112	6983	7256	7120	18000	16858	17429
0.00361	0.00384	2.69E-09	-1.104324	260	231	245.5	533	601	567
0.00175	0.00134	0.006363	-0.91635	73	94	83.5	159	182	170.5
0.00722	0.00998	2.28E-08	-0.608619	1058	1011	1035	1665	1723	1694
0.00282	0.00245	8.76E-08	-0.706932	671	663	667	1064	1283	1174
0.00362	0.00373	3.08E-06	-0.660967	622	542	582	883	1097	990
0.00354	0.00461	0.006518	-0.950169	66	84	75	152	161	156.5
0.0037	0.00505	0.00201	-0.646601	311	221	266	404	488	446
0.00359	0.00358	5.93E-10	-1.404616	157	133	145	413	409	411
0.00636	0.00625	0.000238	-0.739419	259	229	244	400	476	438
0.00768	0.00861	5.45E-07	-2.251303	24	24	24	115	131	123
0.00821	0.01028	8.03E-08	-1.812215	63	45	54	191	215	203
0.00305	0.00253	1.39E-05	-5.432701	0	2	1	41	53	47
0.01028	0.01099	5.84E-12	-1.089233	399	325	362	790	861	825.5
0.00371	0.00246	4.18E-23	-2.401514	92	71	81.5	450	472	461
0.00259	0.0036	0.015644	-2.91431	6	0	3	26	21	23.5
0.00464	0.00527	0.000826	-1.104401	93	65	79	163	201	182
0.00399	0.00592	1.33E-07	-1.053056	222	209	215.5	448	514	481
0.00325	0.00247	0.020596	-0.671537	117	115	116	183	215	199
0.00386	0.005	1.30E-25	-1.971302	328	201	264.5	1160	1043	1102
0.00546	0.0027	1.43E-39	-4.53501	19	15	17	389	458	423.5
0.00854	0.01163	1.62E-27	-1.047346	1780	1503	1642	3236	4061	3649
0.00384	0.00274	2.12E-07	-0.692874	655	629	642	1012	1224	1118
0.00597	0.00709	0.00048	-0.658338	358	271	314.5	489	575	532
0.0038	0.00344	3.57E-38	-1.44096	768	707	737.5	1949	2362	2156
0.00536	0.00381	1.61E-06	-0.633078	663	631	647	997	1163	1080
0.00414	0.00534	4.21E-08	-1.324159	161	118	139.5	338	411	374.5
0.00263	0.00348	0.000159	-1.086809	132	88	110	227	273	250
0.00319	0.00205	1.78E-25	-4.567815	6	17	11.5	247	347	297
0.00199	0.00246	0.000233	-1.904201	21	21	21	82	87	84.5
0.00387	0.00242	1.08E-07	-0.69071	665	599	632	1057	1133	1095
0.00589	0.00872	7.88E-12	-0.890961	762	590	676	1288	1396	1342
0.00475	0.00574	6.82E-05	-6.397791	1	0	0.5	48	42	45
0.00314	0.00383	1.34E-11	-0.600441	2128	1820	1974	3187	3224	3206
0.01408	0.01275	2.44E-05	-7.237737	0	0	0	25	35	30
0.00663	0.00743	2.67E-07	-0.645993	1330	1654	1492	2419	2629	2524
0.00267	0.00186	3.01E-11	-1.398002	171	180	175.5	501	493	497
0.0016	0.0011	0.000596	-3.908824	0	4	2	30	36	33
0.01349	0.01893	1.45E-05	-0.757359	344	312	328	593	596	594.5
0.00451	0.00549	4.30E-05	-1.035991	143	209	176	365	418	391.5
0.0038	0.00364	0.00016	-0.905013	183	141	162	306	344	325
0.00247	0.00092	0.000334	-4.264439	3	22	12.5	249	285	267
0.00341	0.00273	2.90E-13	-2.33769	43	53	48	245	279	262
0.0037	0.0016	0.00162	-1.438999	28	49	38.5	118	108	113
0.00245	0.00111	1.40E-12	-10.44472	0	0	0	267	284	275.5
0.00495	0.00312	8.68E-10	-2.001492	49	65	57	215	280	247.5
0.00789	0.01114	0.000291	-6.653752	0	0	0	17	23	20
0.00716	0.00583	5.23E-07	-0.82722	398	450	424	809	811	810

0.00364	0.00533	9.97E-46	-2.003007	318	325	321.5	1284	1493	1389
0.00367	0.00287	1.78E-14	-6.531632	12	2	7	626	739	682.5
0.00844	0.0104	2.44E-30	-1.759093	335	282	308.5	1143	1091	1117
0.00267	0.00267	1.19E-36	-1.611695	482	444	463	1406	1638	1522
0.01065	0.01516	0.000188	-1.026954	115	107	111	233	253	243
0.00249	0.00266	0.000308	-0.713258	256	272	264	439	494	466.5
0.00643	0.00705	1.59E-12	-0.924641	787	612	699.5	1293	1556	1425
0.00542	0.00535	8.69E-20	-1.410177	341	376	358.5	991	1062	1027
0.00839	0.01241	7.76E-06	-0.7058	421	401	411	679	763	721
0.0027	0.00216	4.39E-07	-1.033315	233	192	212.5	428	506	467
0.00463	0.00543	2.31E-11	-1.396084	201	264	232.5	686	633	659.5
0.00319	0.00461	5.26E-11	-1.541679	161	220	190.5	662	531	596.5
0.00612	0.00705	1.23E-14	-1.250795	344	389	366.5	936	942	939
0.00588	0.00682	0.000155	-6.841054	0	0	0	14	32	23
0.00269	0.0021	2.75E-08	-1.565813	130	75	102.5	305	342	323.5
0.00787	0.01109	0.000427	-4.783158	0	2	1	28	32	30
0.00807	0.01148	0.000172	-1.129023	111	86	98.5	250	209	229.5
0.00241	0.00307	0.000631	-0.718464	280	205	242.5	403	451	427
0.00611	0.00547	1.36E-07	-1.410891	112	94	103	299	287	293
0.00398	0.00453	0.009877	-0.779268	103	129	116	184	248	216
0.00306	0.00441	1.48E-13	-1.303629	264	266	265	706	699	702.5
0.0019	0.00041	0.011083	-3.975882	0	2	1	22	12	17
0.00526	0.00701	3.02E-50	-1.494774	992	1003	997.5	2792	3267	3030
0.0042	0.00432	0.00012	-1.14727	88	94	91	210	224	217
0.00566	0.00391	6.75E-10	-3.385603	11	9	10	98	127	112.5
0.00235	0.00301	1.20E-25	-4.305666	9	18	13.5	255	324	289.5
0.00838	0.00778	1.64E-15	-4.563058	5	7	6	141	165	153
0.00436	0.0042	0.001359	-0.885358	151	107	129	225	286	255.5
0.00476	0.00305	6.11E-16	-7.257764	0	3	1.5	200	298	249
0.00391	0.0028	2.11E-29	-1.873291	274	316	295	1214	1109	1162
0.00545	0.00471	2.21E-09	-9.099204	0	0	0	103	114	108.5
0.00402	0.00532	0.000851	-0.759661	204	178	191	308	388	348
0.00163	0.00141	6.28E-13	-10.58874	0	0	0	305	303	304
0.00336	0.00144	5.26E-07	-8.045542	0	0	0	56	48	52
0.00571	0.00704	3.15E-06	-0.926917	257	215	236	439	525	482
0.00987	0.01115	6.50E-06	-0.616052	752	774	763	1098	1429	1264
0.00967	0.01195	1.41E-38	-2.36516	190	143	166.5	821	1021	921
0.00205	0.00233	3.00E-12	-10.31596	0	0	0	220	286	253
0.00637	0.00473	5.14E-10	-0.714509	1449	1104	1277	2273	2199	2236
0.0029	0.00348	7.16E-35	-1.576107	477	438	457.5	1372	1561	1467
0.00384	0.00229	4.89E-05	-0.591213	560	479	519.5	792	888	840
0.00353	0.00324	2.79E-07	-8.175396	0	0	0	47	68	57.5
0.00189	0.00136	0.000196	-1.101141	129	85	107	212	280	246
0.0008	0.00079	6.40E-08	-1.091667	214	229	221.5	511	504	507.5
0.00426	0.00358	0.003412	-1.588292	24	21	22.5	60	86	73
0.01429	0.0084	0.00033	-5.956812	0	1	0.5	32	35	33.5
0.00522	0.00509	0.002812	-2.904745	4	5	4.5	55	16	35.5
0.00395	0.00483	4.62E-09	-0.697225	888	749	818.5	1330	1519	1425

0.01396	0.01067	0.01008	-1.086932	42	71	56.5	146	113	129.5
0.00326	0.00365	8.01E-07	-0.824328	440	332	386	699	764	731.5
0.00535	0.00657	0.002446	-0.705257	207	163	185	311	335	323
0.00128	0.00134	5.58E-10	-9.036299	0	1	0.5	276	288	282
0.01645	0.0095	0.000439	-0.790562	199	254	226.5	418	428	423
0.00493	0.00268	2.22E-10	-9.540153	0	0	0	123	173	148
0.0064	0.00613	0.001203	-0.671954	241	256	248.5	432	419	425.5
0.00865	0.00874	4.77E-09	-8.955324	0	0	0	98	98	98
0.00361	0.00367	1.96E-32	-1.553978	690	510	600	1902	1859	1881
0.00743	0.00677	0.008769	-0.61097	248	172	210	320	366	343
0.00342	0.00298	4.26E-32	-1.691907	386	446	416	1381	1519	1450
0.00425	0.00462	1.06E-12	-0.898028	811	698	754.5	1333	1693	1513
0.00396	0.00468	5.41E-54	-3.140806	84	85	84.5	755	849	802
0.00304	0.00321	6.81E-16	-1.215999	463	375	419	1061	1020	1041
0.00679	0.00172	0.028841	-1.664757	5	22	13.5	47	47	47
0.00316	0.0037	7.22E-15	-1.163476	455	522	488.5	1175	1182	1179
0.00243	0.00189	5.43E-07	-3.912939	5	3	4	61	68	64.5
0.00273	0.00113	2.28E-05	-7.24945	0	0	0	31	29	30
0.0026	0.00088	0.001504	-0.855395	149	112	130.5	234	272	253
0.01215	0.01373	2.90E-05	-3.299295	1	11	6	60	70	65
0.00124	0.00098	0.040351	-0.664502	137	86	111.5	155	224	189.5
0.01419	0.01138	0.000349	-0.708674	270	233	251.5	422	460	441
0.01405	0.01404	3.67E-22	-1.540525	319	349	334	1061	1028	1045
0.00485	0.00295	2.31E-05	-1.302758	84	76	80	189	236	212.5
0.00464	0.00367	0.000507	-0.589862	656	461	558.5	766	1038	902
0.00436	0.00513	0.004428	-0.71488	160	197	178.5	278	357	317.5
0.00222	0.00183	0.020538	-0.652394	129	147	138	198	272	235
0.00287	0.00288	0.00027	-1.104065	137	78	107.5	237	255	246
0.00201	0.00223	0.001594	-0.632474	382	254	318	509	543	526
0.01196	0.01108	1.14E-07	-4.458363	5	18	11.5	223	336	279.5
0.00526	0.00524	0.001654	-1.5015	24	43	33.5	100	106	103
0.01316	0.01955	4.34E-11	-9.8419	0	0	0	150	215	182.5
0.00278	0.00336	0.000149	-0.773825	342	238	290	550	506	528
0.00763	0.01019	0.000165	-0.876384	174	165	169.5	306	364	335
0.00078	0.0005	0.033546	-4.328526	0	1	0.5	8	14	11
0.00552	0.00616	1.94E-10	-9.560835	0	0	0	128	172	150
0.00357	0.00467	2.86E-93	-2.4127	442	429	435.5	2247	2751	2499
0.00671	0.00803	5.57E-09	-1.315186	155	198	176.5	445	505	475
0.00648	0.00684	4.81E-05	-0.836181	278	263	270.5	425	619	522
0.01588	0.01176	1.30E-14	-3.165839	14	33	23.5	226	232	229
0.0047	0.00401	0.025368	-5.266887	0	0	0	10	5	7.5
0.00423	0.00354	3.20E-05	-2.321166	15	15	15	80	81	80.5
0.00369	0.0051	1.43E-23	-1.769663	207	216	211.5	738	815	776.5
0.00469	0.00435	2.05E-14	-1.950039	96	80	88	353	376	364.5
0.00201	0.00169	1.46E-05	-1.076152	144	202	173	389	400	394.5
0.0037	0.00295	0.005198	-0.847084	97	103	100	179	209	194
0.00196	0.00192	7.12E-08	-1.929674	44	49	46.5	204	176	190
0.00864	0.0019	0.000321	-6.633492	0	0	0	22	17	19.5

0.01195	0.01698	1.30E-15	-2.824608	31	30	30.5	216	249	232.5
0.00216	0.00267	7.32E-14	-2.331762	50	50	50	240	303	271.5
0.00353	0.00305	4.44E-13	-1.127061	424	341	382.5	886	902	894
0.00436	0.00514	3.44E-10	-2.765272	31	14	22.5	150	176	163
0.0116	0.0125	6.25E-15	-1.452847	244	232	238	737	658	697.5
0.01306	0.00524	4.54E-17	-0.852714	1251	1287	1269	2402	2529	2466
0.00299	0.00324	0.004806	-1.481655	26	23	24.5	69	78	73.5
0.00523	0.0038	0.000949	-0.651269	270	249	259.5	427	448	437.5
0.00303	0.00246	5.51E-07	-1.012487	245	195	220	455	496	475.5
0.00264	0.00394	0.000862	-0.960142	110	103	106.5	229	215	222
0.00301	0.00429	9.49E-33	-1.212811	1040	941	990.5	2454	2469	2462
0.005	0.00671	2.57E-37	-1.687112	485	386	435.5	1440	1565	1503
0.00415	0.00227	8.93E-05	-0.745283	308	348	328	616	566	591
0.00269	0.0013	0.002227	-0.683677	225	284	254.5	383	504	443.5
0.00183	0.00064	0.00238	-6.084634	0	0	0	11	16	13.5
0.00238	0.0008	2.56E-07	-8.189505	0	0	0	49	67	58
0.00262	0.00154	0.001181	-6.350733	0	0	0	7	26	16.5
0.00593	0.00605	0.002615	-0.76749	177	130	153.5	268	291	279.5
0.00393	0.00202	0.001057	-1.025858	125	75	100	196	239	217.5
0.00279	0.00302	1.06E-62	-3.267324	91	81	86	853	925	889
0.0023	0.00157	0.001448	-2.154205	8	15	11.5	55	56	55.5
0.00149	0.00154	6.25E-05	-2.109576	32	12	22	99	102	100.5
0.00612	0.0058	5.96E-10	-0.909156	598	471	534.5	957	1199	1078
0.00828	0.00839	1.23E-07	-0.721857	638	699	668.5	1124	1254	1189
0.00317	0.00442	7.25E-13	-1.276172	311	235	273	683	732	707.5
0.00702	0.00878	4.92E-11	-1.200074	313	232	272.5	626	715	670.5
0.00714	0.00859	9.57E-05	-0.941193	152	153	152.5	303	327	315
0.00938	0.0108	1.35E-113	-5.750351	24	23	23.5	1263	1458	1361
0.00475	0.00656	2.82E-16	-0.949597	887	944	915.5	1770	2044	1907
0.00414	0.00491	4.76E-06	-0.609169	994	723	858.5	1378	1419	1399
0.00251	0.00291	1.63E-07	-0.641609	802	826	814	1312	1422	1367
0.00362	0.00398	6.20E-13	-0.848062	774	742	758	1388	1547	1468
0.00167	0.00219	1.70E-70	-2.808824	168	184	176	1247	1411	1329
0.00281	0.00249	0.007842	-5.713287	0	0	0	7	14	10.5
0.00313	0.00291	8.77E-05	-2.367359	28	6	17	85	100	92.5
0.00699	0.00691	2.88E-06	-0.677696	525	474	499.5	808	909	858.5
0.00425	0.00632	2.35E-21	-0.764701	2447	2441	2444	4275	4661	4468
0.00728	0.00878	9.03E-11	-1.01201	433	502	467.5	1005	1027	1016
0.01675	0.01307	8.57E-05	-1.300319	78	61	69.5	184	182	183
0.00344	0.00419	1.59E-08	-1.088401	304	425	364.5	827	850	838.5
0.00151	0.00126	0.010851	-0.646459	153	165	159	248	289	268.5
0.00664	0.00654	0.001069	-0.95296	116	92	104	211	220	215.5
0.00301	0.00248	1.04E-05	-0.738199	464	346	405	699	746	722.5
0.00127	0.00146	0.004232	-1.095529	66	48	57	132	128	130
0.00495	0.00673	3.97E-08	-1.039677	250	232	241	504	561	532.5
0.00531	0.00679	0.033088	-1.84287	4	13	8.5	29	38	33.5
0.00367	0.00492	0.020505	-0.711394	101	102	101.5	167	191	179
0.00672	0.00537	2.45E-15	-3.107228	26	19	22.5	215	199	207

0.00738	0.00953	2.37E-07	-1.642236	65	62	63.5	205	221	213
0.00635	0.00679	1.87E-06	-1.526411	65	70	67.5	211	207	209
0.00439	0.00555	1.29E-05	-1.339915	121	68	94.5	249	260	254.5
0.00208	0.00174	8.59E-13	-1.475068	169	189	179	510	563	536.5
0.00478	0.00523	6.81E-15	-1.376904	267	316	291.5	758	878	818
0.00416	0.00499	7.25E-05	-0.635661	423	388	405.5	653	700	676.5
0.00258	0.00295	1.35E-05	-1.675331	40	48	44	136	168	152
0.00467	0.00473	0.002887	-1.651941	14	38	26	82	97	89.5
0.00341	0.00328	0.008181	-1.250213	32	36	34	88	86	87
0.00289	0.00432	2.02E-95	-3.174611	154	165	159.5	1448	1655	1552
0.00488	0.00462	1.95E-16	-1.854088	122	113	117.5	433	480	456.5
0.00492	0.0033	0.000162	-2.788467	8	6	7	45	59	52
0.004	0.00399	0.000478	-1.271804	57	58	57.5	153	145	149
0.00542	0.00406	0.004457	-1.563358	30	19	24.5	93	60	76.5
0.00185	0.00195	0.004248	-1.135961	52	48	50	114	122	118
0.00708	0.00946	0.048846	-0.807027	63	49	56	111	98	104.5
0.0032	0.00373	0.002705	-1.952939	21	8	14.5	57	62	59.5
0.00522	0.00461	0.030587	-0.835106	64	58	61	121	112	116.5
0.00787	0.00577	4.66E-10	-1.710535	157	87	122	466	379	422.5
0.00524	0.00521	5.50E-09	-1.079922	367	262	314.5	734	683	708.5
0.00471	0.00487	3.82E-18	-1.206574	572	464	518	1146	1423	1285
0.01346	0.01123	1.72E-06	-0.982774	254	192	223	444	500	472
0.00256	0.00282	7.97E-05	-2.503299	18	7	12.5	89	60	74.5
0.00197	0.00272	0.012153	-1.454131	30	16	23	78	55	66.5
0.0061	0.00893	5.79E-05	-1.449007	60	48	54	151	165	158
0.0033	0.00407	7.07E-36	-2.205267	174	168	171	792	905	848.5
0.00344	0.00197	0.001617	-1.265867	48	58	53	158	115	136.5
0.00383	0.00323	4.27E-17	-1.892419	153	105	129	489	535	512
0.00339	0.0023	0.000178	-2.011974	52	11	31.5	149	116	132.5
0.00301	0.00316	0.003661	-0.993035	68	96	82	162	192	177
0.00214	0.00133	0.00601	-3.281095	5	0	2.5	24	27	25.5
0.00415	0.00343	3.41E-05	-1.4985	81	43	62	188	184	186
0.00964	0.01369	0.000113	-0.598452	458	427	442.5	686	754	720
0.00334	0.00196	1.32E-06	-2.991626	10	9	9.5	93	68	80.5
0.00255	0.00328	7.43E-05	-1.308226	64	96	80	210	219	214.5
0.00321	0.00243	0.000708	-1.386205	37	65	51	147	142	144.5
0.00456	0.00363	0.005431	-0.88902	91	84	87.5	156	193	174.5
0.00304	0.00229	1.81E-10	-2.128083	47	47	47	223	218	220.5
0.00371	0.00288	2.77E-08	-2.139762	33	42	37.5	193	162	177.5
0.00356	0.00309	2.16E-05	-3.84934	3	3	3	44	49	46.5
0.00181	0.00228	0.002498	-1.011647	81	166	123.5	263	280	271.5
0.00379	0.00547	0.009953	-1.284457	28	42	35	110	73	91.5
0.00103	0.00091	0.027759	-2.012926	6	7	6.5	34	22	28
0.00473	0.00344	9.29E-08	-1.296602	138	192	165	402	477	439.5
0.00687	0.00799	0.000417	-2.044916	19	13	16	70	71	70.5
0.00269	0.00306	1.59E-08	-2.074731	38	46	42	204	176	190
0.00201	0.00284	1.30E-13	-1.936436	87	80	83.5	336	350	343
0.00503	0.00558	2.81E-05	-0.797644	270	292	281	511	541	526

0.00471	0.00692	0.003015	-0.732911	180	147	163.5	260	324	292
0.00074	0.00069	1.38E-08	-1.453803	167	101	134	395	386	390.5
0.00526	0.00773	0.023037	-1.35051	25	17	21	48	67	57.5
0.00291	0.00206	0.048569	-3.446203	2	0	1	10	13	11.5
0.00916	0.00054	0.031792	-5.156291	0	0	0	8	6	7
0.00304	0.0025	2.00E-05	-1.070001	146	125	135.5	309	300	304.5
0.00454	0.00434	1.26E-34	-1.810404	317	280	298.5	1060	1189	1125
0.00565	0.00583	4.91E-55	-1.581425	1742	2050	1896	5857	6397	6127
0.00409	0.00325	1.51E-08	-8.736305	0	0	0	77	92	84.5
0.00279	0.0017	1.38E-05	-7.372852	0	0	0	38	27	32.5
0.00458	0.00643	8.32E-33	-1.286895	890	754	822	2004	2303	2154
0.00311	0.00423	1.09E-21	-1.116769	711	695	703	1569	1710	1640
0.00255	0.00225	0.001738	-0.635248	294	394	344	560	596	578
0.00224	0.00324	1.20E-08	-1.17926	246	184	215	536	503	519.5
0.00262	0.00253	1.35E-09	-8.857586	0	1	0.5	224	276	250
0.00531	0.00764	2.11E-07	-0.930563	309	348	328.5	614	739	676.5
0.00374	0.00453	0.012067	-1.078868	48	41	44.5	105	96	100.5
0.00451	0.00391	0.009099	-2.029699	3	21	12	54	54	54
0.00107	0.00144	7.99E-15	-2.737689	41	26	33.5	236	241	238.5
0.00508	0.00653	4.82E-13	-0.681354	2018	2069	2044	3194	3878	3536
0.00558	0.00592	2.97E-30	-0.984976	1872	1589	1731	3444	3909	3677
0.00261	0.00278	1.87E-09	-0.992465	343	314	328.5	672	732	702
0.00312	0.00346	4.51E-07	-1.073786	209	171	190	403	455	429
0.00302	0.00411	7.46E-22	-1.464463	410	381	395.5	1006	1349	1178
0.00396	0.005	5.29E-62	-1.825148	621	547	584	2119	2324	2222
0.00705	0.0099	8.25E-44	-1.729484	796	563	679.5	2351	2462	2407
0.00365	0.00433	3.93E-34	-8.176812	3	2	2.5	726	829	777.5
0.00221	0.00141	4.45E-13	-10.64822	0	0	0	313	321	317
0.00347	0.0041	1.06E-11	-1.454571	382	202	292	769	936	852.5
0.00491	0.00732	3.29E-20	-0.97357	2212	1696	1954	3587	4655	4121
0.00653	0.00844	0.041562	-2.812476	1	3	2	10	21	15.5
0.00379	0.0035	5.36E-16	-1.750449	135	141	138	486	513	499.5
0.002	0.00212	1.45E-09	-0.844015	527	511	519	977	1025	1001
0.00646	0.00523	5.68E-61	-1.915202	514	488	501	1988	2070	2029
0.00398	0.00374	1.18E-07	-0.644159	784	752	768	1256	1323	1290
0.00225	0.00114	0.000603	-1.844266	24	17	20.5	69	89	79
0.00319	0.00455	3.26E-91	-1.631264	1772	1544	1658	5091	5948	5520
0.0032	0.00287	1.90E-10	-1.012463	387	429	408	868	905	886.5
0.00257	0.00337	7.41E-48	-1.515333	832	717	774.5	2285	2465	2375
0.00285	0.00215	0.045273	-2.595075	6	0	3	16	22	19
0.0065	0.00697	0.030505	-0.769786	120	62	91	155	175	165
0.00767	0.01118	1.49E-37	-5.402957	8	10	9	418	399	408.5
0.00418	0.00619	0.001153	-0.910503	202	108	155	291	329	310
0.00291	0.00339	4.35E-15	-2.11615	74	118	96	414	490	452
0.00357	0.00527	1.39E-11	-1.383471	253	382	317.5	814	987	900.5
0.00146	0.00057	0.00018	-6.784005	0	0	0	16	28	22
0.00453	0.0058	0.000394	-0.757646	224	245	234.5	441	411	426
0.00575	0.00759	3.78E-90	-2.261206	517	432	474.5	2314	2567	2441

0.01473	0.00268	0.020266	-5.347899	0	0	0	9	7	8
0.00719	0.00536	2.28E-13	-10.54043	0	1	0.5	798	800	799
0.00687	0.00486	2.64E-05	-0.697615	439	532	485.5	813	888	850.5
0.00945	0.00955	1.02E-07	-0.761548	518	529	523.5	908	1003	955.5
0.00273	0.00398	5.31E-41	-1.708887	465	407	436	1482	1576	1529
0.00639	0.0065	6.61E-16	-0.91662	918	965	941.5	1799	2032	1916
0.0027	0.00247	2.36E-05	-1.017267	158	213	185.5	371	443	407
0.00279	0.00285	5.54E-07	-0.803911	414	451	432.5	809	816	812.5
0.00428	0.00464	0.000368	-0.718865	321	231	276	445	528	486.5
0.00328	0.00361	6.05E-09	-1.999529	51	42	46.5	188	211	199.5
0.00536	0.00789	3.48E-65	-2.293088	333	342	337.5	1798	1754	1776
0.00238	0.00243	1.28E-14	-1.118642	432	397	414.5	933	1000	966.5
0.00356	0.0046	1.38E-06	-0.603613	798	840	819	1268	1414	1341
0.00216	0.00222	5.29E-06	-1.012989	189	244	216.5	473	470	471.5
0.00765	0.01016	1.00E-14	-0.918553	834	875	854.5	1605	1879	1742
0.00181	0.00203	7.70E-09	-1.743527	73	63	68	242	246	244
0.00904	0.00844	5.01E-11	-4.319545	7	3	5	105	108	106.5
0.00351	0.00191	0.03174	-5.164588	0	0	0	9	5	7
0.0044	0.00441	0.000835	-0.650448	281	251	266	423	474	448.5
0.00836	0.00982	0.001674	-1.269743	49	48	48.5	139	111	125
0.00753	0.00722	1.21E-35	-1.377399	787	675	731	1900	2179	2040
0.00573	0.0054	0.003568	-1.051524	74	56	65	131	158	144.5
0.00925	0.00633	0.003445	-4.99746	7	0	3.5	116	115	115.5
0.00404	0.00449	0.000467	-0.983126	122	206	164	334	371	352.5
0.00334	0.00336	1.05E-20	-1.03414	1863	2233	2048	4530	4515	4523
0.00256	0.00332	3.98E-18	-2.15261	111	78	94.5	464	432	448
0.01737	0.02094	0.043209	-4.208975	0	10	5	94	114	104
0.00743	0.0056	0.000792	-0.59968	359	353	356	512	652	582
0.00387	0.0046	0.029412	-2.861138	5	0	2.5	18	20	19
0.00157	0.00077	7.54E-28	-3.157159	37	46	41.5	385	413	399
0.00327	0.00379	4.13E-18	-1.920164	147	109	128	473	566	519.5
0.00449	0.00594	3.41E-16	-1.099089	669	582	625.5	1503	1362	1433
0.00299	0.00305	0.000126	-1.215252	525	750	637.5	1510	1704	1607
0.00383	0.00464	1.08E-06	-0.586656	1125	894	1010	1464	1791	1628
0.00156	0.00188	9.06E-09	-0.848422	491	433	462	888	895	891.5
0.0046	0.00635	1.28E-29	-1.075768	1212	1085	1149	2503	2694	2599
0.00095	0.00057	0.027053	-2.964646	2	2	2	23	10	16.5
0.00267	0.00262	7.19E-21	-1.594555	245	242	243.5	742	841	791.5
0.00123	0.00142	2.73E-05	-1.381308	63	84	73.5	211	202	206.5
0.00223	0.00261	0.015578	-0.728297	101	125	113	199	205	202
0.00348	0.00403	2.45E-07	-1.067675	226	189	207.5	419	516	467.5
0.00539	0.00576	6.16E-13	-0.797744	1041	918	979.5	1836	1812	1824
0.00297	0.00208	0.039368	-1.42225	8	29	18.5	51	58	54.5
0.00454	0.00512	4.49E-09	-2.201443	47	29	38	169	205	187
0.00485	0.00563	3.59E-16	-1.714774	168	135	151.5	485	583	534
0.00567	0.00837	0.040937	-0.807076	103	48	75.5	125	156	140.5
0.00436	0.00632	1.90E-06	-0.840502	501	332	416.5	775	816	795.5
0.00433	0.00407	4.75E-05	-1.186341	213	96	154.5	360	385	372.5

0.00642	0.00655	0.000174	-2.796399	5	9	7	55	50	52.5
0.00299	0.00212	3.72E-08	-1.330509	139	120	129.5	321	379	350
0.00245	0.00153	3.12E-10	-9.465722	0	0	0	130	150	140
0.00258	0.00217	0.039544	-1.121283	23	34	28.5	56	79	67.5
0.00483	0.00676	4.55E-15	-1.040064	703	703	703	1629	1470	1550
0.00451	0.00523	2.56E-09	-0.683767	991	1044	1018	1648	1876	1762
0.01215	0.00892	0.00914	-1.583254	15	21	18	52	65	58.5
0.00388	0.0023	2.58E-10	-2.893424	17	18	17.5	132	148	140
0.00521	0.0063	7.63E-07	-0.691302	1063	1061	1062	2040	1627	1834
0.00366	0.00402	9.79E-19	-0.952905	1036	1025	1031	1955	2345	2150
0.00393	0.00498	1.46E-13	-1.044077	508	427	467.5	997	1070	1034
0.00835	0.01215	2.83E-08	-0.980976	316	293	304.5	660	628	644
0.00194	0.00231	0.001394	-0.858648	125	139	132	260	255	257.5
0.00393	0.00439	2.14E-09	-0.690692	1107	879	993	1684	1746	1715
0.00365	0.0028	6.56E-10	-1.446	186	140	163	522	423	472.5
0.00876	0.00926	2.36E-25	-0.887537	1874	1641	1758	3406	3567	3487
0.00419	0.00471	1.90E-63	-2.608725	194	183	188.5	1206	1263	1235
0.00421	0.00273	1.81E-07	-0.632357	1036	1179	1108	1777	1927	1852
0.00368	0.00368	7.38E-06	-0.609219	735	824	779.5	1177	1392	1285
0.0026	0.00319	0.013216	-5.529929	0	0	0	12	6	9
0.00201	0.00165	3.01E-12	-0.893568	974	707	840.5	1585	1755	1670
0.00342	0.00269	4.93E-08	-0.975064	395	311	353	631	862	746.5
0.00574	0.00711	2.34E-08	-0.724924	813	931	872	1466	1646	1556
0.0027	0.00281	2.59E-14	-2.314173	60	46	53	279	285	282
0.00393	0.00398	4.82E-26	-1.782073	299	219	259	878	1031	954.5
0.00186	0.00167	0.006078	-5.789374	0	0	0	9	13	11
0.00363	0.00309	3.21E-10	-1.380181	169	220	194.5	556	536	546
0.00693	0.00724	0.001608	-0.73183	239	180	209.5	315	433	374
0.0035	0.00516	2.40E-12	-1.77528	143	87	115	411	428	419.5
0.00819	0.00474	3.87E-27	-1.305983	863	932	897.5	2472	2295	2384
0.00291	0.0041	3.13E-24	-2.198405	119	106	112.5	503	608	555.5
0.00237	0.00214	6.54E-20	-1.603859	281	213	247	739	871	805
0.00575	0.00762	3.41E-114	-1.979007	1040	964	1002	4125	4357	4241
0.00342	0.00225	0.044021	-1.266529	12	37	24.5	53	77	65
0.00188	0.0016	0.000211	-2.174023	184	70	127	600	607	603.5
0.00435	0.00613	2.18E-21	-0.954911	1302	1066	1184	2339	2582	2461
0.0037	0.00401	3.50E-18	-1.500949	267	230	248.5	750	757	753.5
0.00229	0.00307	9.01E-11	-1.575323	130	105	117.5	342	410	376
0.00269	0.00285	2.53E-09	-1.585623	146	86	116	369	372	370.5
0.00175	0.00193	0.00441	-0.840079	113	101	107	213	197	205
0.00136	0.00136	0.002422	-0.712636	266	179	222.5	332	450	391
0.00276	0.00279	2.85E-15	-0.98453	1073	877	975	1766	2387	2077
0.0077	0.00981	3.94E-07	-2.115205	28	32	30	136	144	140
0.00562	0.00659	4.10E-25	-1.333594	565	590	577.5	1432	1708	1570
0.00364	0.00292	0.015133	-5.484258	0	0	0	5	13	9
0.00457	0.00655	3.35E-05	-3.116783	4	8	6	52	61	56.5
0.00401	0.00426	2.33E-06	-0.777306	418	490	454	824	854	839
0.00247	0.00335	5.97E-11	-0.947794	483	439	461	877	1036	956.5

0.00397	0.00441	4.09E-17	-12.21798	0	0	0	871	1016	943.5
0.00712	0.00544	8.03E-07	-7.982361	0	0	0	60	39	49.5
0.00658	0.00975	1.81E-25	-7.250793	5	0	2.5	370	447	408.5
0.00315	0.00428	1.53E-09	-0.913383	551	513	532	913	1250	1082
0.00274	0.00392	0.000308	-1.179933	79	77	78	158	224	191
0.00731	0.00946	4.76E-20	-0.783906	2122	1827	1975	3374	3930	3652
0.00756	0.00948	1.53E-14	-0.876176	1331	1529	1430	2587	3089	2838
0.0045	0.00537	1.63E-25	-2.789224	57	58	57.5	374	484	429
0.00373	0.00537	6.70E-07	-1.103719	217	157	187	431	427	429
0.00899	0.01078	2.98E-15	-0.920981	938	825	881.5	1830	1743	1787
0.00416	0.00286	1.16E-07	-8.338789	0	0	0	62	66	64
0.00352	0.00402	4.45E-08	-0.793977	562	637	599.5	1041	1204	1123
0.01493	0.02011	8.88E-120	-1.629035	3004	2531	2768	8674	9694	9184
0.0032	0.00396	9.72E-20	-0.863397	1559	1622	1591	2984	3248	3116
0.00314	0.00413	2.41E-42	-1.686293	705	837	771	2649	2701	2675
0.00266	0.00166	8.42E-27	-3.776819	16	34	25	320	426	373
0.00281	0.00319	7.38E-08	-1.2101	243	154	198.5	491	487	489
0.0033	0.00446	1.08E-21	-2.048147	123	123	123	503	593	548
0.00381	0.00407	5.50E-05	-0.717374	321	329	325	555	595	575
0.0044	0.00564	9.03E-10	-0.592975	1387	1278	1333	2081	2236	2159
0.00476	0.00542	1.37E-41	-1.948059	317	275	296	1165	1287	1226
0.00699	0.00468	0.00245	-4.411156	2	0	1	22	23	22.5
0.00419	0.00559	7.04E-09	-0.684675	821	797	809	1345	1451	1398
0.00532	0.00569	3.66E-06	-1.029574	183	162	172.5	362	394	378
0.00345	0.00214	1.82E-09	-1.010409	341	297	319	675	703	689
0.00581	0.00604	2.24E-07	-0.658844	1899	1266	1583	2416	2927	2672
0.00335	0.00307	8.40E-42	-1.239349	1598	1685	1642	3947	4406	4177
0.00583	0.00439	1.45E-24	-1.14621	836	708	772	1745	1921	1833
0.0037	0.00507	0.000178	-0.781576	256	219	237.5	381	498	439.5
0.00288	0.00243	0.014042	-0.917057	60	72	66	117	153	135
0.00607	0.00874	5.82E-06	-0.666057	552	490	521	798	981	889.5
0.00193	0.00222	9.73E-09	-0.980721	316	321	318.5	633	721	677
0.00208	0.00269	1.05E-19	-1.562233	247	266	256.5	803	827	815
0.00252	0.00218	1.25E-07	-1.273822	132	135	133.5	330	365	347.5
0.00424	0.00588	3.18E-26	-0.896114	2225	1880	2053	4044	4138	4091
0.00123	0.0017	2.43E-08	-1.502222	125	84	104.5	297	336	316.5
0.00735	0.00745	1.07E-25	-0.741073	3705	3301	3503	5848	6737	6293
0.00443	0.00522	4.38E-09	-1.0217	342	287	314.5	698	668	683
0.0043	0.00581	4.04E-13	-0.867882	891	983	937	1742	1947	1845
0.00413	0.00473	0.000572	-1.022186	165	91	128	291	260	275.5
0.00324	0.00258	0.014356	-1.65647	38	6	22	83	61	72
0.0022	0.00188	0.002905	-6.027495	0	0	0	10	16	13
0.00463	0.00208	0.006908	-0.770466	121	136	128.5	198	277	237.5
0.00166	0.00156	6.41E-11	-0.800269	764	756	760	1416	1427	1422
0.00593	0.00616	5.70E-08	-0.945262	330	291	310.5	583	703	643
0.00295	0.00306	1.03E-07	-0.693238	865	678	771.5	1213	1464	1339
0.00428	0.00501	2.55E-05	-1.163302	112	110	111	228	309	268.5
0.00351	0.00363	1.07E-05	-0.612257	684	737	710.5	1190	1145	1168

0.00305	0.00352	4.89E-07	-0.691371	581	542	561.5	914	1036	975
0.00288	0.00222	1.65E-08	-0.754125	843	664	753.5	1206	1524	1365
0.00323	0.00419	1.14E-07	-0.825951	452	480	466	799	985	892
0.0031	0.00268	9.72E-09	-1.715468	70	118	94	325	345	335
0.00695	0.00906	6.42E-20	-1.300093	573	424	498.5	1215	1415	1315
0.00246	0.00284	0.004416	-1.006801	79	68	73.5	126	193	159.5
0.00826	0.00856	0.001962	-6.139594	0	0	0	12	16	14
0.00535	0.00793	0.001558	-1.141303	84	52	68	165	154	159.5
0.00121	0.00134	0.001213	-0.634358	393	579	486	778	859	818.5
0.00887	0.00898	3.28E-21	-0.997813	1069	1110	1090	2199	2490	2345
0.00483	0.00598	0.000191	-0.678719	333	310	321.5	565	538	551.5
0.00692	0.00467	0.001151	-2.892862	7	3	5	50	28	39
0.00392	0.00484	0.007598	-0.680236	154	145	149.5	245	270	257.5
0.00333	0.00372	5.73E-23	-0.903977	1508	1325	1417	2776	2909	2843
0.0052	0.0063	1.26E-49	-1.436161	1436	1578	1507	4271	4516	4394
0.00739	0.00744	7.82E-08	-8.417862	0	0	0	68	67	67.5
0.00533	0.0059	1.36E-08	-0.825119	612	467	539.5	993	1052	1023
0.0062	0.00691	0.000863	-0.707981	314	212	263	421	498	459.5
0.00298	0.00342	0.000249	-0.972532	122	158	140	284	310	297
0.00382	0.00299	0.022373	-1.675702	6	22	14	49	49	49
0.00443	0.00394	5.25E-09	-0.776913	602	597	599.5	1058	1152	1105
0.00183	0.00177	6.35E-29	-1.129353	1002	958	980	2220	2388	2304
0.00386	0.0029	1.62E-05	-0.806258	323	413	368	672	719	695.5
0.00443	0.00428	1.43E-43	-1.236028	1649	1652	1651	3836	4545	4191
0.00556	0.00484	1.15E-09	-0.900691	515	443	479	977	937	957
0.00446	0.00468	2.45E-14	-1.186844	357	363	360	831	934	882.5
0.00195	0.0017	7.05E-09	-1.354634	144	185	164.5	463	444	453.5
0.0009	0.00126	1.54E-16	-1.705995	167	140	153.5	529	544	536.5
0.00546	0.00737	0.009925	-0.648849	187	143	165	251	304	277.5
0.00156	0.00196	1.64E-14	-1.411993	239	219	229	664	642	653
0.0021	0.00191	2.42E-12	-0.587937	2579	2578	2579	3852	4501	4177
0.00854	0.00961	2.40E-05	-0.863351	229	215	222	403	466	434.5
0.00581	0.00679	1.71E-10	-1.530612	119	139	129	397	406	401.5
0.00443	0.0021	0.000324	-0.735748	268	282	275	526	456	491
0.00744	0.01	1.06E-147	-1.974216	1516	1379	1448	5884	6329	6107
0.00586	0.00833	7.17E-17	-0.976092	781	679	730	1485	1596	1541
0.00459	0.00576	3.67E-21	-1.841016	192	266	229	858	917	887.5
0.00359	0.00359	2.01E-07	-0.761607	539	449	494	834	964	899
0.00399	0.00312	0.000973	-0.740055	237	176	206.5	341	398	369.5
0.00658	0.00896	3.89E-05	-7.44438	8	0	4	698	741	719.5
0.00421	0.0044	0.000155	-0.673582	346	307	326.5	511	609	560
0.00216	0.00299	1.36E-11	-1.832417	88	89	88.5	363	312	337.5
0.00109	0.00135	2.75E-08	-0.78678	667	618	642.5	1257	1115	1186
0.00957	0.01041	1.38E-22	-1.062761	1053	1143	1098	2376	2567	2472
0.00113	0.00073	2.10E-06	-1.166493	137	137	137	337	323	330
0.00708	0.00691	4.32E-18	-0.606155	3577	3221	3399	5305	5807	5556
0.00278	0.00211	9.47E-11	-0.966453	555	418	486.5	993	1040	1017
0.00408	0.00517	0.000133	-6.851414	0	0	0	18	28	23

0.00537	0.00568	3.28E-25	-1.684139	305	241	273	873	1009	941
0.00098	0.00115	1.12E-07	-8.396819	0	0	0	44	91	67.5
0.00513	0.00419	2.49E-09	-0.764007	881	924	902.5	1716	1574	1645
0.00512	0.00626	3.78E-92	-2.076054	749	783	766	3419	3528	3474
0.00426	0.00393	3.53E-16	-0.999323	1083	880	981.5	1811	2411	2111
0.00435	0.00649	6.12E-17	-0.784822	1615	1567	1591	2694	3212	2953
0.00318	0.00301	2.72E-36	-1.583482	538	497	517.5	1501	1839	1670
0.00772	0.00629	0.004594	-3.442288	3	1	2	25	21	23
0.00429	0.00531	4.41E-25	-0.944571	1483	1300	1392	2706	3046	2876
0.00478	0.00661	0.000321	-0.594904	430	362	396	599	685	642
0.0096	0.01417	1.77E-10	-0.745526	930	813	871.5	1574	1556	1565
0.0085	0.01245	0.008964	-0.601071	358	213	285.5	482	437	459.5
0.00386	0.00356	3.76E-06	-0.672251	511	481	496	811	888	849.5
0.00364	0.00412	9.62E-07	-0.736245	465	452	458.5	769	875	822
0.0051	0.00194	4.74E-11	-2.124298	48	55	51.5	244	239	241.5
0.002	0.00293	1.69E-90	-3.814893	75	78	76.5	1069	1250	1160
0.00509	0.00679	6.68E-59	-1.563727	1208	1024	1116	3559	3503	3531
0.00651	0.00551	0.014885	-4.468228	8	0	4	76	108	92
0.00488	0.0037	0.002909	-0.854284	114	106	110	196	232	214
0.00553	0.00803	5.24E-06	-0.679904	515	436	475.5	782	852	817
0.00242	0.0031	0.019205	-1.019554	46	49	47.5	80	129	104.5
0.00457	0.00466	0.003248	-0.689727	200	163	181.5	307	320	313.5
0.00281	0.00208	1.33E-05	-1.438726	60	85	72.5	209	216	212.5
0.00683	0.00596	5.38E-05	-0.973314	195	162	178.5	414	333	373.5
0.00797	0.00995	0.001406	-1.157806	96	50	73	158	189	173.5
0.00333	0.00292	0.016853	-1.241023	28	28	28	73	69	71
0.00294	0.00374	2.10E-07	-3.309589	15	3	9	98	90	94
0.00377	0.00307	5.78E-07	-1.905231	53	32	42.5	171	168	169.5
0.00496	0.00544	0.001238	-1.110161	66	95	80.5	161	217	189
0.00251	0.0035	0.000286	-1.143979	93	71	82	186	202	194
0.00297	0.00418	3.45E-14	-1.078656	477	423	450	946	1097	1022
0.00252	0.00309	0.000102	-0.756881	270	316	293	507	562	534.5
0.00696	0.00806	0.00031	-0.695811	341	268	304.5	462	598	530

Supplemental table S7

name	PR_K27me_LogFC	PR_K27me_KO	PR_K27me_WT	GB_K27me_LogFC	GB_K36_LogFC_KO/	GB_K27me_KO	GB_K27me_WT	GB_K36_KO
)0109H08I	-1.132	0.0024	0.0053	-0.24	-1.651	0.0051	0.006	0.0309
)0110K17I	-0.108	0.0046	0.0049	-0.678	-1.833	0.0041	0.0065	0.0011
)0002D01I	0.103	0.0048	0.0044	-0.383	-2.589	0.0066	0.0086	0.0057
10528A11I	0.2392	0.006	0.0051	-0.015	-1.415	0.0123	0.0124	0.0023
10021J22I	0.3142	0.0033	0.0026	0.1091	-0.866	0.0023	0.0021	0.0064
33406O09I	-0.723	0.003	0.0049	-0.97	-2.547	0.0017	0.0033	0.0038
30417C22I	0.1865	0.0153	0.0135	0.5671	-1.02	0.0141	0.0095	0.005
30188P03I	-0.632	0.0014	0.0022	-0.555	-1.134	0.0006	0.0009	0.0035
30020M07I	0.0017	0.0029	0.0029	0.3755	-0.725	0.0028	0.0022	0.0061
30012L18I	0.1298	0.0017	0.0016	-0.024	-1.012	0.0012	0.0013	0.0039
AACS	0.4575	0.0048	0.0035	-0.129	-1.228	0.003	0.0033	0.0132
AARSD1	0.1729	0.0037	0.0033	0.2577	-0.891	0.0052	0.0043	0.0103
ABCB1A	-2.333	0.0004	0.002	-0.562	-2.128	0.0028	0.0041	0.0081
ABCB1B	0.0223	0.01	0.0098	0.0662	-0.832	0.0071	0.0068	0.0166
ABCD3	-0.632	0.0021	0.0032	0.0135	-1.096	0.0015	0.0015	0.0043
ABHD11OS	-0.842	0.0045	0.008	-1.669	-1.441	0.0027	0.0086	0.009
ABHD2	0.2147	0.0029	0.0025	-0.29	-1.144	0.0014	0.0018	0.0087
ABHD6	-0.522	0.0017	0.0025	-0.423	-1.594	0.0015	0.0021	0.0047
ABI3	-0.517	0.0038	0.0054	-0.316	-1.084	0.0058	0.0072	0.0118
ACAA1B	0.3787	0.0049	0.0038	-0.429	-1.652	0.004	0.0054	0.0062
ACSF2	0.384	0.004	0.0031	-0.201	-0.89	0.0023	0.0027	0.0076
ACSL4	-1.555	0.0003	0.001	0.4804	-0.976	0.0005	0.0004	0.0018
ACY3	-0.591	0.0099	0.015	0.286	-2.294	0.0075	0.0062	0.0034
ADAT2	-0.138	0.0009	0.001	0.0304	-0.668	0.0015	0.0014	0.0027
ADGRL2	-1.969	0.0005	0.002	-0.867	-1.539	0.0009	0.0016	0.0036
ADH7	-0.377	0.003	0.0039	0.2792	-1.142	0.0039	0.0032	0.0028
ADIPOR2	-0.94	0.0028	0.0055	0.3276	-0.648	0.0032	0.0025	0.0054
AFF4	-0.314	0.0015	0.0019	0.0969	-1.028	0.0012	0.0011	0.0085
AFG3L2	-0.533	0.0027	0.0039	-0.191	-1.546	0.0018	0.0021	0.0051
AGL	0.2764	0.003	0.0024	-0.62	-1.062	0.0012	0.0019	0.0049
AGO2	-0.969	0.0027	0.0052	-0.517	-0.816	0.0016	0.0024	0.012
AKAP9	-1.189	0.0044	0.0101	-0.885	-1.347	0.0037	0.0068	0.0154
ALDOC	0.0309	0.0042	0.0042	0.2289	-1.06	0.0052	0.0044	0.014
ALG5	0.476	0.0037	0.0027	-0.153	-1.625	0.0025	0.0028	0.0052
ALS2CL	0.1085	0.0055	0.0051	-0.464	-0.885	0.0034	0.0047	0.0151
AMMECR1I	-0.612	0.0024	0.0036	0.0439	-0.963	0.0016	0.0016	0.0065
ANKHD1	-0.135	0.0038	0.0042	-0.21	-1.513	0.0007	0.0008	0.0026
ANKIB1	-1.047	0.0031	0.0065	-0.825	-1.288	0.0028	0.005	0.0124
ANKRD13C	-0.612	0.0019	0.0028	0.0161	-0.769	0.0011	0.0011	0.0039
ANKRD50	-0.02	0.0049	0.0049	-0.345	-1.12	0.0017	0.0022	0.012
ANP32E	-0.048	0.0062	0.0064	-0.251	-1.362	0.0025	0.003	0.0081
ANXA13	-0.809	0.0011	0.0019	-0.44	-2.282	0.0015	0.0021	0.0072
ANXA3	0.031	0.0029	0.0028	-0.216	-0.842	0.0014	0.0016	0.011
ANXA4	0.2404	0.0043	0.0036	-0.392	-2.141	0.0018	0.0023	0.008

AP1G1	-0.271	0.0025	0.003	0.4988	-0.622	0.0009	0.0007	0.0034
APLP2	-0.108	0.0023	0.0025	0.1897	-1.168	0.0013	0.0011	0.0034
APOB	-0.554	0.0014	0.002	-0.145	-0.702	0.0012	0.0013	0.0039
AQP5	-0.311	0.037	0.0459	-0.126	-0.991	0.0448	0.0489	0.0104
AREG	-0.518	0.0015	0.0022	-0.053	-1.553	0.0013	0.0014	0.0149
RHGAP11	-0.136	0.0066	0.0073	0.3347	-0.86	0.0029	0.0023	0.0069
ARHGAP12	-1.506	0.0012	0.0033	-0.792	-1.153	0.0006	0.001	0.0033
ARHGAP18	0.447	0.0014	0.001	0.2091	-0.831	0.0008	0.0007	0.0028
ARHGAP27	0.015	0.0106	0.0105	-0.217	-0.768	0.0093	0.0108	0.0146
ARHGAP33	0.4439	0.0152	0.0111	-0.409	-0.777	0.0084	0.0111	0.0066
ARHGAP40	-0.314	0.0037	0.0046	-0.304	-0.627	0.0057	0.0071	0.0066
ARL14	-0.528	0.0022	0.0032	-0.232	-0.999	0.0029	0.0034	0.0081
ARL4A	-0.291	0.0019	0.0023	0.4032	-1.261	0.0016	0.0012	0.0028
ARL5B	0.3523	0.0023	0.0018	0.0764	-1.428	0.0008	0.0008	0.0036
ARMC1	-1.046	0.001	0.0022	-0.016	-1.025	0.0017	0.0017	0.0034
ARMCX5	0.0314	0.0019	0.0018	-0.258	-1.709	0.0014	0.0016	0.0014
ARNT	-0.132	0.0024	0.0027	-0.003	-0.905	0.0016	0.0016	0.007
ASAH1	0.515	0.0016	0.0011	-0.628	-0.916	0.001	0.0015	0.0067
ASAP2	-0.025	0.0015	0.0015	-0.318	-0.984	0.001	0.0012	0.0063
ASH1L	-0.077	0.0015	0.0016	-0.398	-0.998	0.0011	0.0014	0.0074
ATP1A1	-1.158	0.0191	0.0427	-0.557	-1.469	0.0026	0.0039	0.0099
ATP1B3	-0.806	0.0016	0.0028	-0.056	-1.194	0.0007	0.0007	0.0027
ATP5A1	-0.597	0.0013	0.0019	0.1599	-1.154	0.0027	0.0024	0.006
ATP6V0A1	0.533	0.002	0.0014	-0.212	-1.534	0.0029	0.0034	0.0133
AZI2	0.4999	0.0042	0.003	-0.011	-1.578	0.0012	0.0012	0.0023
B3GALNT1	-1.337	0.0172	0.0435	-1.703	-0.851	0.0069	0.0224	0.0032
B4GALT5	0.4359	0.014	0.0104	-0.229	-0.897	0.0026	0.003	0.0058
B4GALT6	-0.876	0.0009	0.0017	-0.929	-1.83	0.0007	0.0014	0.0037
BC031181	-0.244	0.0036	0.0043	-0.113	-1.209	0.0032	0.0035	0.0037
BCAR3	-1.056	0.0009	0.0019	-0.453	-1.097	0.0017	0.0023	0.0089
BEND3	-0.198	0.0017	0.0019	-0.026	-0.707	0.0012	0.0012	0.0044
BIN1	-0.607	0.0021	0.0032	-0.47	-1.482	0.0018	0.0024	0.0068
BMPR1A	-0.242	0.0014	0.0017	-0.552	-0.697	0.0007	0.001	0.0036
BRD8	-0.417	0.0013	0.0017	-0.048	-1.752	0.0012	0.0012	0.004
BTF3L4	-0.417	0.0013	0.0017	0.3643	-0.603	0.0011	0.0009	0.0033
BTG2	0.513	0.0047	0.0033	0.2284	-1.79	0.0038	0.0033	0.0112
BVHT	-0.984	0.0028	0.0055	-0.554	-1.816	0.0055	0.0081	0.0036
C2CD3	0.0306	0.0015	0.0015	0.2862	-0.681	0.0012	0.001	0.0043
C2CD4C	0.3908	0.0245	0.0187	-0.123	-0.826	0.0195	0.0212	0.0094
30027H18	-0.249	0.0016	0.0019	-0.246	-3.17	0.0014	0.0016	0.0017
C77080	0.3071	0.0037	0.003	-0.709	-0.88	0.0019	0.0031	0.0103
CAMK2D	-0.998	0.0015	0.003	-0.432	-0.999	0.0009	0.0012	0.0049
CAPN7	0.0042	0.0063	0.0063	0.3763	-0.608	0.0023	0.0017	0.004
CAPZA1	-0.438	0.0023	0.0031	-0.015	-1.031	0.0013	0.0013	0.0061
CAPZA2	-0.292	0.0009	0.0011	0.4456	-0.683	0.0008	0.0006	0.0045
CAR8	-0.708	0.0007	0.0011	-0.133	-1.106	0.0007	0.0008	0.0045
CCAR1	0.2651	0.0023	0.0019	-0.762	-0.588	0.0006	0.001	0.0047
CCDC25	0.5613	0.0015	0.001	-0.226	-0.726	0.001	0.0011	0.0045

CCDC47	-0.152	0.0043	0.0048	-0.643	-0.669	0.0017	0.0027	0.0136
CCDC66	-0.095	0.0013	0.0014	-0.682	-0.961	0.0008	0.0013	0.0045
CCDC68	0.2233	0.0009	0.0008	-0.446	-0.597	0.0009	0.0012	0.0035
CCDC88C	0.3902	0.0045	0.0034	0.5626	-1.116	0.0023	0.0016	0.0053
CCNC	0.0301	0.0025	0.0024	0.4461	-0.682	0.001	0.0007	0.0017
CD24A	-0.729	0.0015	0.0025	-0.778	-1.748	0.0008	0.0014	0.0053
CD82	-0.503	0.0066	0.0094	-1.048	-1.19	0.0029	0.006	0.0095
CDC14A	-0.385	0.0033	0.0043	-0.199	-0.837	0.0014	0.0017	0.0044
CDC23	-0.541	0.0023	0.0033	-1.11	-0.812	0.0008	0.0017	0.0062
CDC25C	-0.554	0.0019	0.0027	-0.439	-1.574	0.0012	0.0017	0.0031
CDK6	-0.977	0.0056	0.011	-0.992	-1.446	0.0035	0.0071	0.0253
CELA1	-0.255	0.0061	0.0073	-1.128	-0.614	0.0044	0.0097	0.0052
CELF1	0.2943	0.0035	0.0028	0.2894	-0.622	0.0012	0.001	0.0046
CELF4	-0.569	0.0734	0.109	-0.804	-0.835	0.0058	0.0101	0.0045
CELF5	-0.028	0.0307	0.0313	-0.205	-0.941	0.0586	0.0676	0.0072
CELSR2	-0.355	0.0538	0.0688	-0.393	-0.881	0.0233	0.0305	0.0112
CEP120	-0.913	0.0015	0.0028	-0.049	-1.395	0.0013	0.0013	0.0044
CEP192	0.2707	0.0015	0.0013	-0.354	-1.271	0.0007	0.0009	0.0042
CEP57	-0.361	0.0019	0.0024	-0.095	-0.953	0.0008	0.0008	0.0037
CEP76	-0.622	0.0008	0.0013	-0.878	-1.018	0.0007	0.0013	0.0036
CEP95	-0.478	0.003	0.0042	0.3202	-0.909	0.0023	0.0019	0.0101
CHKA	-0.047	0.0021	0.0022	-0.349	-2.225	0.0014	0.0017	0.0101
CISD2	-0.265	0.0026	0.0031	-0.779	-1.244	0.0015	0.0026	0.0054
CLCC1	-1.171	0.0017	0.0039	0.1136	-1.057	0.0023	0.0021	0.0067
CLCN6	0.1306	0.0035	0.0032	0.1136	-0.964	0.0016	0.0015	0.0055
CLDN12	-1.344	0.005	0.0127	-1.199	-2.338	0.005	0.0114	0.0323
CLIC4	-0.351	0.0038	0.0049	0.2441	-1.043	0.0016	0.0013	0.0047
CLPX	-0.233	0.0017	0.002	0.1319	-1.003	0.0013	0.0012	0.0043
CLTC	0.533	0.002	0.0014	0.0917	-1.162	0.0009	0.0009	0.0068
CMTM4	-0.47	0.0024	0.0033	-0.293	-0.94	0.0013	0.0015	0.0045
CMTM6	0.1729	0.0037	0.0033	0.3107	-0.661	0.002	0.0016	0.0044
CNKSR1	0.3008	0.0048	0.0039	-0.139	-1.112	0.004	0.0044	0.0056
CNOT1	-0.327	0.0023	0.0028	0.2762	-0.823	0.0009	0.0007	0.004
CNOT6	0.0308	0.0033	0.0032	0.3319	-0.681	0.001	0.0008	0.0052
COG6	-0.205	0.0046	0.0053	0.0793	-0.668	0.0024	0.0023	0.0056
COPB1	0.0314	0.0014	0.0014	-0.287	-0.587	0.0007	0.0008	0.0039
CORO2A	0.5006	0.0052	0.0037	-0.447	-2.007	0.0017	0.0023	0.005
COX5A	0.3458	0.0033	0.0026	0.536	-0.774	0.0014	0.0009	0.0048
CRAMP1L	0.1954	0.0038	0.0033	0.3675	-0.761	0.0022	0.0017	0.0085
CRLS1	0.2234	0.0037	0.0032	0.0167	-0.791	0.0018	0.0017	0.0051
CROT	-1.22	0.0063	0.0146	-0.487	-0.951	0.003	0.0043	0.0165
CSDE1	-0.933	0.0035	0.0068	-1.212	-0.656	0.0009	0.002	0.0096
CSNK1A1	-1.68	0.0013	0.0041	-1.665	-1.414	0.0005	0.0015	0.0037
CSNK1G3	-1.971	0.0006	0.0025	-0.551	-1.142	0.0005	0.0008	0.0024
CSNK2A1	-1.032	0.0026	0.0053	-0.594	-0.779	0.0009	0.0014	0.0039
CTDP1	-0.408	0.008	0.0106	-0.434	-1.293	0.0023	0.0031	0.0058
CTDSPL2	0.2836	0.0065	0.0053	0.1431	-0.691	0.0009	0.0008	0.0037
CTH	-0.922	0.0053	0.0101	-0.333	-1.103	0.0016	0.002	0.0032

CTPS	0.1508	0.0029	0.0026	-0.171	-0.941	0.0009	0.001	0.0046
CUL1	-0.97	0.0015	0.003	-0.074	-0.587	0.001	0.001	0.0043
CUL2	-0.03	0.0013	0.0014	-0.095	-0.823	0.0007	0.0008	0.0023
CUL5	-0.318	0.0013	0.0016	0.1436	-0.778	0.0007	0.0006	0.0022
CXXC1	-0.75	0.0019	0.0031	-0.895	-1.456	0.002	0.0037	0.0091
CXXC4	-0.206	0.0033	0.0038	-0.315	-0.904	0.0015	0.0018	0.0017
CYB5A	0.5041	0.0029	0.002	-0.678	-1.777	0.001	0.0016	0.0046
CYP51	-0.392	0.0111	0.0146	-0.03	-2.458	0.006	0.0062	0.0157
CYSTM1	-1.317	0.0013	0.0032	-0.871	-2.207	0.0015	0.0027	0.0077
30028M14I	-0.432	0.0298	0.0402	0.151	-1.07	0.0355	0.032	0.0079
DAP3	-0.64	0.0028	0.0044	-0.427	-1.285	0.0021	0.0029	0.0083
DBF4	-1.177	0.0032	0.0072	-1.068	-1.806	0.002	0.0042	0.0106
DDX1	-2.284	0.0001	0.0006	-0.607	-0.959	0.0004	0.0006	0.0027
DDX20	-1.124	0.0062	0.0134	-0.262	-1.112	0.0034	0.0041	0.0062
DENND2C	-0.686	0.0016	0.0026	-0.05	-1.067	0.0026	0.0027	0.0047
DENND6A	-0.121	0.0021	0.0023	-0.483	-1.191	0.0008	0.0011	0.0039
DEPDC1A	-0.078	0.0008	0.0008	-1.006	-1.15	0.0004	0.0008	0.0015
DFFB	0.1237	0.0056	0.0051	-0.516	-1.064	0.0029	0.0042	0.0043
DHCR24	0.2296	0.0036	0.0031	0.2653	-1.145	0.002	0.0017	0.0066
DHX40	-0.456	0.0012	0.0016	0.2828	-0.762	0.0012	0.001	0.0047
DIO3	-0.593	0.1131	0.1706	-0.456	-0.694	0.1568	0.215	0.0039
DMTF1	-0.199	0.0077	0.0089	-0.52	-1.379	0.0026	0.0037	0.0085
DMXL1	-1.697	0.0009	0.003	-0.506	-0.692	0.0004	0.0006	0.0023
DNTTIP2	-0.305	0.0024	0.003	0.159	-0.856	0.003	0.0027	0.009
DPH5	0.1684	0.0026	0.0023	-0.036	-1.395	0.0009	0.0009	0.0023
DPM1	-1.232	0.0017	0.0041	-0.02	-0.862	0.0023	0.0023	0.0075
DPP8	0.1374	0.0024	0.0022	-0.108	-0.968	0.0011	0.0012	0.0032
DRAM2	-1.033	0.0013	0.0026	-0.433	-1.299	0.0007	0.001	0.0034
DSG2	-0.476	0.0044	0.0061	-0.203	-0.66	0.0017	0.0019	0.003
DSTN	-0.534	0.0028	0.004	-0.906	-1.625	0.0012	0.0022	0.0075
DUSP11	0.0017	0.0029	0.0029	-0.706	-0.943	0.0015	0.0025	0.0057
DUSP6	-0.192	0.0014	0.0016	-0.646	-0.817	0.0008	0.0013	0.0238
DYM	-0.242	0.0042	0.0049	-0.516	-0.767	0.0013	0.0018	0.0042
DYRK2	0.0306	0.0015	0.0015	-0.357	-0.779	0.0009	0.0012	0.0042
ECHDC2	-1.829	0.0031	0.0111	-2.434	-0.622	0.0013	0.0068	0.0037
ECT2	-0.455	0.0029	0.004	-0.356	-0.657	0.0008	0.001	0.0085
EDN2	-0.884	0.0071	0.0131	-0.844	-1.343	0.0054	0.0098	0.0057
EED	-0.259	0.0021	0.0025	-1.232	-0.645	0.0004	0.0009	0.0043
EEF1AKMT	0.4464	0.0019	0.0014	0.2053	-0.706	0.0018	0.0016	0.0041
EEPD1	0.3065	0.0027	0.0022	0.4826	-0.634	0.0013	0.0009	0.0031
EGR1	-0.574	0.0097	0.0144	-0.516	-2.506	0.0095	0.0136	0.029
EHF	-0.385	0.0016	0.002	0.24	-0.691	0.0019	0.0016	0.0075
EIF1A	0.2234	0.0023	0.002	0.3089	-0.722	0.0016	0.0013	0.0087
EIF2A	-0.251	0.003	0.0035	-0.03	-0.691	0.0013	0.0013	0.0044
ELAC1	-0.412	0.0015	0.0019	-1.483	-1.388	0.001	0.0028	0.0037
ELOVL6	-0.724	0.0028	0.0046	-0.21	-1.044	0.0013	0.0015	0.0048
ELP2	0.2839	0.0036	0.003	-0.371	-1.045	0.0014	0.0018	0.0056
EML4	-1.322	0.001	0.0026	0.0079	-0.968	0.0012	0.0012	0.005

EPPK1	-0.111	0.0079	0.0085	-0.326	-1.209	0.0037	0.0047	0.0127
EPS8L3	-0.645	0.0045	0.007	-0.69	-3.244	0.0035	0.0056	0.0085
ERCC6	0.2269	0.0032	0.0027	-0.1	-0.652	0.0013	0.0014	0.0051
EREG	-0.51	0.0013	0.0018	0.1545	-0.702	0.0011	0.001	0.0044
ERO1L	-1.554	0.0013	0.0038	-0.933	-3.782	0.0008	0.0015	0.0026
ERRFI1	-1.455	0.0012	0.0032	-0.72	-0.796	0.001	0.0017	0.0391
ESCO1	-1.419	0.0015	0.004	-0.475	-0.789	0.0005	0.0007	0.0031
ETF1	-0.872	0.0013	0.0024	-0.868	-1.106	0.0009	0.0016	0.0082
ETFDH	-0.076	0.006	0.0064	-0.184	-1.466	0.0015	0.0017	0.0041
ETV3	0.1288	0.008	0.0073	-0.095	-0.904	0.0026	0.0028	0.0107
EXOSC8	0.476	0.0037	0.0027	0.1068	-0.895	0.002	0.0019	0.0104
EYA2	-0.656	0.0201	0.0317	-0.828	-1.378	0.003	0.0053	0.0066
F3	-0.355	0.0028	0.0036	-0.282	-1.434	0.0019	0.0023	0.0196
FAM102B	0.1626	0.0027	0.0024	-0.175	-0.634	0.002	0.0022	0.008
FAM114A2	-0.706	0.0014	0.0023	-0.103	-0.93	0.0018	0.002	0.0051
FAM120A	0.3358	0.0024	0.0019	0.3516	-0.877	0.0014	0.0011	0.0051
FAM136A	-0.349	0.0048	0.0061	0.3592	-1.232	0.0041	0.0032	0.0117
FAM13B	-0.22	0.0022	0.0025	-0.89	-1.045	0.0007	0.0013	0.0048
FAM168A	-0.97	0.0008	0.0016	-0.682	-1.076	0.0005	0.0008	0.0019
FAM210A	-1.258	0.001	0.0025	-0.891	-1.016	0.0007	0.0012	0.0039
FAM43A	-0.65	0.0045	0.0071	-0.303	-1.785	0.0057	0.007	0.0084
FAM53C	-0.688	0.0018	0.0029	-0.625	-1.611	0.0015	0.0024	0.0071
FAM83G	0.1183	0.0059	0.0055	0.0563	-1.22	0.0047	0.0045	0.0143
FAM91A1	0.308	0.0023	0.0019	0.5184	-0.946	0.002	0.0014	0.0096
FAR1	0.3718	0.0022	0.0017	-0.602	-1.079	0.0005	0.0007	0.0028
FASN	-0.152	0.0082	0.0091	-0.157	-1.419	0.0078	0.0087	0.0383
FBXL3	0.3718	0.0022	0.0017	-0.607	-1.445	0.0006	0.001	0.0036
FCHSD2	-0.844	0.0017	0.0031	-0.75	-1.058	0.0004	0.0006	0.0015
FDFT1	-0.472	0.0028	0.0039	-0.655	-1.008	0.0012	0.0019	0.006
FECH	0.279	0.0022	0.0018	-0.286	-1.094	0.001	0.0013	0.0046
FGF1	-3.978	0.0017	0.0275	-2.564	-1.129	0.0013	0.0078	0.0052
FHDC1	-2.286	0.003	0.0147	-1.329	-0.693	0.0025	0.0062	0.0074
FOXA2	0.1844	0.0058	0.0051	-0.106	-0.707	0.0051	0.0055	0.0165
FOXQ1	-0.806	0.0016	0.0028	-0.485	-2.961	0.0017	0.0024	0.0067
FPGT	-0.506	0.0023	0.0033	0.3746	-1.222	0.0014	0.001	0.0035
FUBP1	-1.166	0.0028	0.0063	-1.03	-1.517	0.0009	0.0019	0.0109
FXR1	-0.148	0.0031	0.0034	-0.377	-0.793	0.0007	0.001	0.0035
G6PC3	0.253	0.0033	0.0027	-0.297	-1.282	0.0041	0.005	0.0123
GABPB2	-0.053	0.002	0.002	-0.271	-1.182	0.0019	0.0023	0.0073
GALNT1	-1.583	0.0009	0.0028	-0.57	-0.706	0.0008	0.0012	0.0035
GARS	-1.033	0.0013	0.0026	-0.113	-0.732	0.002	0.0022	0.0075
GATAD2B	0.5171	0.0008	0.0006	0.2935	-0.976	0.0015	0.0012	0.0061
GDPD3	-0.872	0.0027	0.0049	-0.806	-1.569	0.0011	0.0018	0.0058
GFM1	-0.2	0.003	0.0035	-0.311	-0.876	0.0015	0.0018	0.0052
GFPT1	-1.116	0.0016	0.0035	0.1173	-1.132	0.0016	0.0015	0.0077
GLIPR1	-0.108	0.0012	0.0013	-0.385	-1.29	0.001	0.0013	0.0049
GM14023	-0.259	0.0021	0.0025	-0.082	-0.79	0.002	0.0022	0.0051
GM14305	-1.056	0.0005	0.001	-1.331	-1.872	0.0002	0.0005	0.0008

GM14317	0.4164	0.0047	0.0035	0.559	-0.712	0.0028	0.0019	0.0069
GM15638	0.4903	0.0013	0.0009	-0.286	-0.6	0.0015	0.0018	0.005
GM2237	-0.062	0.0017	0.0018	-0.25	-1.725	0.0007	0.0008	0.0006
GM3604	0.286	0.0022	0.0018	0.3098	-0.624	0.0008	0.0006	0.0016
GM5148	-0.384	0.0017	0.0023	-1.281	-0.735	0.0009	0.0022	0.0077
GM8773	-0.332	0.032	0.0402	-0.193	-2.796	0.0458	0.0524	0.0055
GMPS	0.3422	0.0021	0.0017	0.3225	-0.74	0.001	0.0008	0.0048
GOLM1	-0.907	0.0014	0.0026	-0.963	-1.23	0.0009	0.0018	0.0076
GOLPH3	-0.506	0.0023	0.0033	-0.16	-0.721	0.0012	0.0013	0.004
GOLPH3L	0.5547	0.0027	0.0018	-0.332	-1.37	0.0011	0.0014	0.0069
GPBP1	-0.969	0.0031	0.006	0.0822	-0.654	0.0006	0.0006	0.0032
GPD1L	0.4999	0.0042	0.003	0.2259	-0.599	0.0025	0.0021	0.0041
GPR68	0.142	0.0219	0.0199	-0.799	-1.075	0.0135	0.0235	0.0032
GPRC5A	0.445	0.0028	0.002	-0.119	-1.358	0.002	0.0022	0.0208
GRAMD3	-1.383	0.001	0.0027	-0.715	-0.723	0.0009	0.0015	0.0032
GRPEL2	-0.012	0.0038	0.0039	0.1603	-2.309	0.0026	0.0024	0.0035
GSS	-0.027	0.0099	0.0101	0.2567	-1.107	0.0039	0.0033	0.008
GSTA1	0.2707	0.0015	0.0013	-0.258	-1.343	0.0005	0.0006	0.0019
GSTA4	-0.484	0.0016	0.0023	0.3826	-1.134	0.0015	0.0011	0.0069
GSTM3	-1.241	0.0213	0.0504	-1.554	-0.592	0.0077	0.0226	0.0088
GSTM5	-0.257	0.005	0.006	-0.266	-0.877	0.007	0.0084	0.0109
GTF2E2	-0.467	0.002	0.0027	0.1682	-1.391	0.0012	0.0011	0.0046
GTPBP10	-0.954	0.0053	0.0102	-0.832	-1.629	0.0033	0.0059	0.0141
HADH	-0.422	0.0022	0.003	-0.077	-1.396	0.0041	0.0043	0.0059
HAL	0.5823	0.0161	0.0108	0.0659	-0.872	0.0109	0.0104	0.0024
HBEGF	-0.554	0.0019	0.0027	-0.645	-1.545	0.002	0.0031	0.0455
HIPK1	-1.583	0.002	0.0059	-0.857	-1.085	0.0014	0.0026	0.0062
HMGCR	0.4449	0.0016	0.0012	-0.283	-1.641	0.0012	0.0014	0.0038
HMGCS1	0.1041	0.0023	0.0022	-0.706	-1.91	0.0006	0.001	0.0032
HMGXB3	-0.179	0.0037	0.0042	-0.673	-1.284	0.0016	0.0025	0.0064
HNF4A	0.5379	0.0031	0.0022	0.0246	-2.312	0.0025	0.0025	0.005
HNRNPH3	-0.357	0.0015	0.0019	-1.599	-0.894	0.0004	0.0013	0.0037
HOXA13	-0.284	0.1536	0.187	-0.513	-1.474	0.1585	0.2262	0.0043
HPRT	-2.434	0.0002	0.0013	-0.033	-1.078	0.0005	0.0005	0.002
HSPA4L	-1.006	0.0023	0.0047	-0.023	-1.032	0.001	0.0011	0.0053
HSPA9	-2.155	0.0006	0.0028	-1.278	-1.334	0.0005	0.0011	0.0119
IARS	0.5061	0.0015	0.001	-0.014	-1.097	0.0009	0.0009	0.0058
IBTK	-0.573	0.0015	0.0022	0.1062	-1.002	0.0007	0.0006	0.0033
ICE1	-0.024	0.0015	0.0015	-0.204	-0.874	0.0008	0.001	0.0044
ID1	-0.472	0.0042	0.0058	-0.037	-2.122	0.0063	0.0065	0.0416
IER2	-0.274	0.0057	0.0069	-0.872	-1.749	0.0071	0.013	0.0281
IFI27L2A	-1.147	0.0035	0.0079	-1.643	-0.63	0.0022	0.007	0.004
IMPA2	-0.173	0.0038	0.0043	-0.984	-1.541	0.0018	0.0035	0.0059
INSIG1	0.4117	0.0073	0.0055	-0.236	-2.42	0.0031	0.0036	0.0104
ISG20L2	-0.326	0.0062	0.0078	-0.861	-0.737	0.0042	0.0076	0.0134
ITGA2	0.1673	0.0013	0.0011	-0.291	-2.215	0.0007	0.0009	0.0044
ITGA3	0.3262	0.0063	0.005	-0.332	-1.277	0.0032	0.004	0.0235
ITGB3	-1.259	0.0026	0.0063	-0.317	-0.992	0.0024	0.003	0.0124

ITPK1	-0.062	0.0017	0.0018	-0.141	-1.019	0.0016	0.0017	0.006
IVL	-0.023	0.0016	0.0016	-0.662	-0.834	0.0016	0.0025	0.0028
IWS1	-0.647	0.0017	0.0027	0.3023	-0.844	0.001	0.0008	0.0045
JADE1	-0.597	0.0013	0.0019	-0.837	-0.788	0.0017	0.003	0.0068
JAG1	-0.844	0.0017	0.0031	-0.029	-1.171	0.0014	0.0014	0.0109
KAT6A	-0.577	0.0024	0.0036	-0.181	-0.692	0.0017	0.002	0.0064
KDM1B	0.2131	0.0049	0.0042	0.3527	-0.853	0.0017	0.0013	0.0034
KDM3B	-0.349	0.0023	0.003	-0.766	-1.346	0.0009	0.0015	0.0046
KDM5A	-0.361	0.0019	0.0024	-0.243	-0.844	0.0008	0.001	0.0056
KIF5B	-0.233	0.0012	0.0014	-0.671	-1.013	0.0005	0.0008	0.0035
KLF11	0.0308	0.0064	0.0063	0.2585	-0.864	0.0027	0.0023	0.0079
KLF3	-1.82	0.001	0.0037	-0.458	-1.829	0.0016	0.0022	0.0133
KLF5	-1.492	0.0005	0.0013	-0.216	-1.92	0.001	0.0012	0.0089
KLHDC10	-0.667	0.0026	0.0042	-0.15	-0.688	0.0014	0.0016	0.0046
KLHL9	-0.498	0.003	0.0043	-0.675	-1.027	0.0031	0.005	0.0055
KNTC1	0.0304	0.0013	0.0013	0.5809	-0.739	0.0018	0.0012	0.0076
KRIT1	-1.021	0.0031	0.0064	-0.61	-1.229	0.0024	0.0037	0.0106
KRT19	-0.005	0.0046	0.0047	-0.184	-1.782	0.0038	0.0043	0.0489
KRT20	0.3879	0.0042	0.0032	0.5275	-2.725	0.0054	0.0037	0.0078
KRT23	0.3617	0.0045	0.0035	-0.279	-1.952	0.0028	0.0034	0.0063
L2HGDH	0.3636	0.002	0.0015	0.2559	-1.184	0.0009	0.0008	0.003
LARP7	-1.586	0.0017	0.0052	-0.511	-1.489	0.0017	0.0024	0.0038
LARS	-0.661	0.0015	0.0024	-0.621	-0.849	0.0012	0.0018	0.0055
LBH	-1.221	0.0024	0.0057	-0.554	-1.267	0.0015	0.0023	0.0071
LDLR	-0.272	0.0017	0.0021	-0.861	-1.916	0.0013	0.0023	0.0109
LDLRAP1	0.4112	0.0048	0.0036	-0.567	-1.931	0.0018	0.0027	0.0065
LENG8	-0.701	0.0029	0.0047	-1.337	-1.742	0.0019	0.0049	0.0152
LGALS2	-0.39	0.0034	0.0045	-0.018	-1.807	0.0043	0.0044	0.0107
LGALS3BP	0.0304	0.0049	0.0048	-0.119	-1.49	0.0055	0.006	0.0229
LGALS4	-0.076	0.003	0.0032	0.2463	-2.457	0.0032	0.0027	0.006
LGALS9	-0.728	0.003	0.005	-0.38	-0.647	0.0024	0.0031	0.0095
LMNB1	-0.372	0.0033	0.0042	-1.01	-0.941	0.0011	0.0021	0.0072
LONRF3	-1.61	0.0015	0.0044	-0.292	-1.215	0.0004	0.0005	0.0019
LPIN1	0.4396	0.0137	0.0101	0.0253	-1.094	0.0049	0.0048	0.0046
LPIN2	0.188	0.0034	0.003	-0.333	-0.869	0.0015	0.0019	0.0065
LRFN3	-0.216	0.015	0.0174	0.3103	-0.732	0.0117	0.0095	0.0061
LRP8	-0.981	0.007	0.0138	-0.604	-0.85	0.0019	0.003	0.0064
LSS	0.3773	0.0052	0.004	0.3826	-1.401	0.0032	0.0024	0.0082
LUC7L3	-0.467	0.002	0.0027	0.2023	-0.59	0.0013	0.0011	0.0064
MAB21L3	-0.139	0.0042	0.0046	0.1032	-1.067	0.008	0.0074	0.0044
MALAT1	-0.084	0.0014	0.0015	0.2943	-1.1	0.0015	0.0012	0.0376
MAP3K1	-0.555	0.0007	0.001	-0.359	-1.512	0.001	0.0012	0.0058
MAPK15	0.565	0.0349	0.0236	0.4852	-0.955	0.1136	0.0811	0.0088
MAPK6	0.2175	0.0024	0.002	-0.071	-0.922	0.0012	0.0013	0.0061
MATR3	-0.554	0.0014	0.002	-1.092	-0.951	0.0004	0.0008	0.0043
MBD1	0.5571	0.0021	0.0014	-0.673	-1.6	0.0013	0.0021	0.0063
MBD2	-0.97	0.0013	0.0025	-0.777	-1.218	0.0008	0.0013	0.0046
MBNL2	-0.069	0.0016	0.0017	-0.211	-0.772	0.001	0.0011	0.0054

MBP	0.4111	0.0033	0.0024	-0.839	-1.045	0.0023	0.0042	0.0052
MCL1	-1.055	0.0019	0.004	-0.277	-0.826	0.0016	0.002	0.0268
ME2	-1.235	0.0027	0.0064	-0.863	-1.114	0.0009	0.0017	0.004
MEIS2	-0.309	0.0028	0.0035	0.2131	-0.81	0.0014	0.0012	0.0038
METTL7A1	-0.51	0.0026	0.0036	0.3731	-1.038	0.0033	0.0025	0.0056
MEX3C	-0.612	0.0019	0.0028	-0.622	-1.093	0.0005	0.0008	0.0023
MFN1	-0.46	0.0024	0.0034	0.3975	-0.711	0.0021	0.0016	0.0048
MFNG	-0.597	0.0078	0.0118	-1.405	-0.586	0.0093	0.0246	0.0106
MFSD1	0.2333	0.0071	0.006	0.0307	-0.759	0.0023	0.0022	0.0046
MFSD14A	0.2715	0.003	0.0025	0.0306	-1.383	0.0016	0.0015	0.0058
MFSD8	-1.027	0.0014	0.0028	0.1902	-1.102	0.0016	0.0014	0.0043
MGAT3	-0.714	0.0022	0.0035	-0.323	-1.081	0.0018	0.0023	0.01
MIER1	-0.591	0.0015	0.0023	-0.118	-0.831	0.0006	0.0007	0.0039
MINK1	0.0983	0.0026	0.0024	-0.01	-1.089	0.0026	0.0026	0.0131
MISP	-0.484	0.0016	0.0023	-0.126	-1.388	0.0026	0.0028	0.0048
MLLT6	0.3675	0.0084	0.0065	0.0661	-0.969	0.0072	0.0068	0.0189
MPHOSPH6	-0.683	0.0029	0.0047	0.1973	-1.053	0.0016	0.0014	0.0051
MPPE1	-0.455	0.0023	0.0032	0.3699	-1.666	0.0025	0.0019	0.0035
MRPL47	-0.407	0.0028	0.0037	-0.241	-1.087	0.0022	0.0026	0.0082
MRPS15	-0.265	0.0026	0.0031	-0.541	-1.963	0.0016	0.0023	0.0033
MRPS30	0.0304	0.0013	0.0013	0.5002	-1.627	0.0009	0.0006	0.003
MSLN	0.3437	0.0092	0.0073	-0.094	-0.73	0.006	0.0065	0.014
MTERF1B	-1.032	0.0039	0.008	0.0308	-1.328	0.0025	0.0025	0.0068
MTF2	-0.384	0.0017	0.0023	-0.027	-0.655	0.001	0.001	0.0048
MTFR1	0.4089	0.0045	0.0034	0.4457	-0.985	0.0019	0.0014	0.0057
MTHFD2	-0.146	0.004	0.0044	-0.054	-1.173	0.0029	0.003	0.0083
MTMR11	0.3933	0.0037	0.0028	0.265	-0.905	0.0029	0.0024	0.0085
MTPAP	-0.024	0.003	0.0031	0.0307	-0.739	0.001	0.001	0.0032
MYBL2	0.4382	0.004	0.003	-0.254	-1.176	0.0024	0.0029	0.0089
MYNN	-0.755	0.0034	0.0057	-0.466	-1.062	0.0018	0.0024	0.0078
MYO6	0.2715	0.003	0.0025	0.0132	-1.179	0.0008	0.0008	0.0026
MYO7B	-0.656	0.0024	0.0038	-0.811	-0.952	0.0025	0.0044	0.0061
MYOZ3	-1.57	0.0105	0.0311	-1.182	-1.051	0.0143	0.0325	0.0032
NBEAL2	0.3332	0.0043	0.0034	-0.438	-1.373	0.0024	0.0033	0.0105
NBN	-0.069	0.0016	0.0017	0.1343	-0.85	0.001	0.0009	0.0031
NCAPG2	0.5215	0.003	0.0021	0.3184	-0.635	0.001	0.0008	0.0043
NCOA3	-1.206	0.0008	0.0019	-0.056	-0.7	0.0016	0.0017	0.0058
NDST1	-0.267	0.002	0.0024	-0.458	-0.908	0.0024	0.0034	0.0082
NDUFA9	0.1464	0.003	0.0027	-0.647	-0.654	0.0011	0.0017	0.0061
NDUFAF1	-1.979	0.0022	0.0087	-1.776	-0.588	0.0022	0.0074	0.0067
NDUFB5	-0.325	0.0029	0.0036	-0.128	-0.775	0.0017	0.0018	0.0069
NEDD4L	-0.463	0.0016	0.0022	-0.875	-0.837	0.0009	0.0017	0.0038
NEK4	-0.301	0.0036	0.0044	0.5516	-0.631	0.0027	0.0018	0.0037
NES	-1.035	0.0824	0.1689	-1.229	-0.906	0.0625	0.1464	0.0116
NEU1	0.3994	0.0059	0.0045	0.134	-2.429	0.005	0.0046	0.0062
NEURL3	0.1278	0.0027	0.0024	0.031	-1.494	0.0021	0.0021	0.009
NFATC1	-2.417	0.0357	0.1905	-1.846	-0.84	0.0037	0.0131	0.0072
NGLY1	-0.291	0.0016	0.002	-0.233	-1.189	0.0008	0.0009	0.0021

NIPAL1	0.3477	0.0041	0.0032	0.2022	-2.329	0.002	0.0017	0.007
NKAP	-0.842	0.001	0.0019	0.0682	-0.723	0.0011	0.0011	0.002
NPY4R	-1.491	0.0041	0.0114	-1.649	-0.944	0.0023	0.0071	0.0043
NQO1	0.4289	0.0017	0.0013	0.2383	-0.813	0.0023	0.0019	0.0028
NR1D2	-0.782	0.0043	0.0074	-0.715	-0.713	0.001	0.0016	0.005
NRN1	0.3781	0.0016	0.0013	-0.777	-0.894	0.0006	0.0011	0.0054
NSMAF	0.3938	0.001	0.0008	0.1639	-0.76	0.0008	0.0007	0.0033
OAS1C	-1.37	0.0023	0.0059	-0.979	-0.692	0.0044	0.0087	0.0045
OASL1	-0.384	0.0045	0.0059	-0.364	-1.229	0.0029	0.0038	0.012
ODF2L	-1.233	0.0012	0.0027	-0.253	-0.776	0.0012	0.0015	0.0051
ONECUT2	-0.586	0.0017	0.0026	-1.481	-2.517	0.0006	0.0018	0.0069
ORC3	-0.969	0.0005	0.0009	0.5027	-1.162	0.0006	0.0004	0.0029
OSGIN1	-1.064	0.0026	0.0053	0.1456	-1.917	0.0075	0.0068	0.0112
OTUD7B	0.4461	0.0026	0.0019	-0.631	-1.001	0.0015	0.0023	0.0069
OXSM	-0.249	0.0016	0.0019	0.1556	-1.202	0.0015	0.0013	0.004
P2RY1	-0.693	0.0037	0.0059	-0.364	-0.96	0.003	0.0039	0.0036
P2RY2	-0.648	0.0087	0.0137	0.0979	-0.818	0.0064	0.006	0.0051
P2RY6	-1.388	0.0064	0.0167	-1.706	-1.36	0.0083	0.027	0.0033
PADI2	0.5336	0.004	0.0027	0.1262	-0.795	0.002	0.0018	0.0052
PAFAH1B1	-0.554	0.0019	0.0027	-0.012	-0.864	0.0009	0.0009	0.0043
PAIP2	0.3098	0.002	0.0016	0.0563	-1.568	0.0011	0.001	0.0042
PAK4	-0.882	0.002	0.0036	-0.287	-1.114	0.0027	0.0033	0.0083
PAQR8	-0.969	0.0038	0.0074	-0.711	-2.402	0.0018	0.003	0.0037
PBRM1	-0.062	0.0035	0.0036	-0.117	-1.27	0.0008	0.0008	0.0035
PCDH1	0.0852	0.0063	0.0059	-0.619	-1.181	0.0027	0.0042	0.0077
PCDHB16	0.5396	0.0043	0.003	0.1626	-0.709	0.003	0.0027	0.0016
PCDHB22	-0.064	0.0026	0.0027	-0.471	-0.972	0.0027	0.0038	0.0045
PCLO	-1.438	0.0033	0.0088	-1.915	-1.795	0.0008	0.0032	0.0026
PCYOX1L	-0.449	0.0044	0.006	-0.5	-1.015	0.0038	0.0053	0.006
PDE4DIP	-0.345	0.0534	0.0678	0.3515	-0.753	0.0049	0.0038	0.0056
PDE6A	-0.818	0.0012	0.002	-1.075	-1.037	0.0013	0.0028	0.0038
PDIK1L	0.2301	0.0054	0.0046	0.2531	-1.083	0.0029	0.0024	0.0031
PDLIM5	-0.926	0.002	0.0038	-0.196	-0.728	0.0011	0.0013	0.0043
PDP2	-0.014	0.0037	0.0037	0.2452	-0.761	0.0027	0.0023	0.0039
PDXK	-0.895	0.0035	0.0065	0.0221	-0.69	0.0042	0.0041	0.0089
PEAR1	-0.724	0.0113	0.0187	0.0459	-1.793	0.0117	0.0114	0.0141
PEX12	0.5385	0.0037	0.0026	0.3009	-0.794	0.0037	0.003	0.0076
PFDN1	-0.348	0.0012	0.0015	-0.796	-1.341	0.0009	0.0015	0.0073
PFDN6	-0.449	0.0022	0.003	-1.554	-0.779	0.0019	0.0057	0.01
PGRMC1	0.5007	0.0021	0.0015	0.3038	-0.785	0.0013	0.001	0.0034
PHAX	0.3529	0.0017	0.0014	-0.576	-0.885	0.0012	0.0018	0.0054
PHC3	-0.183	0.0029	0.0033	-0.094	-0.838	0.0013	0.0014	0.0056
PHF6	0.279	0.0022	0.0018	0.2269	-0.843	0.0004	0.0004	0.0012
PHYKPL	0.1397	0.0064	0.0058	-0.259	-0.816	0.0031	0.0036	0.0214
PIK3C3	0.1829	0.0012	0.001	-0.366	-0.794	0.0009	0.0012	0.0038
PIK3CA	-0.97	0.0012	0.0023	-0.217	-0.937	0.0012	0.0014	0.0052
PIK3R1	-1.02	0.0016	0.0033	0.1865	-0.717	0.001	0.0009	0.0029
PIR	0.5441	0.0012	0.0008	0.1508	-1.055	0.0003	0.0003	0.0013

PKD2L2	-0.145	0.0184	0.0204	-0.667	-1.015	0.0075	0.012	0.0041
PLA2G12A	-0.573	0.0059	0.0088	-0.517	-0.724	0.0029	0.0042	0.0058
PLAT	-0.216	0.0025	0.0029	0.4264	-1.059	0.0028	0.0021	0.0193
PLAUR	0.5048	0.0044	0.0031	0.5498	-1.136	0.0033	0.0023	0.031
PLEK2	-0.526	0.002	0.0028	0.1603	-1.279	0.002	0.0018	0.0048
PLSCR1	0.2513	0.0008	0.0007	-1.265	-2.023	0.0005	0.0012	0.0092
PMAIP1	-0.417	0.0013	0.0017	-0.867	-3.902	0.0011	0.002	0.0025
PMVK	0.253	0.0098	0.0082	-0.124	-1.39	0.0053	0.0058	0.0058
POLR1B	-0.622	0.002	0.0031	-0.475	-0.587	0.0019	0.0026	0.0085
POLR3F	0.2623	0.0196	0.0163	0.5066	-0.656	0.003	0.0021	0.0059
POLR3K	-0.135	0.0038	0.0042	0.2352	-1.378	0.0029	0.0024	0.0035
POP5	-0.222	0.0055	0.0064	0.1038	-0.772	0.0055	0.0051	0.0082
PORCN	-1.845	0.0003	0.0013	0.2527	-1.76	0.001	0.0008	0.0025
PPARG	-0.844	0.0014	0.0025	-0.426	-1.738	0.001	0.0013	0.0049
PPID	-0.902	0.0013	0.0024	-0.838	-0.818	0.0013	0.0023	0.0066
PPP1CB	-0.026	0.0029	0.003	-0.643	-0.85	0.0008	0.0013	0.0052
PPP1R3B	0.0115	0.0086	0.0085	-0.332	-1.4	0.0026	0.0032	0.0049
PPP3CB	0.1398	0.0024	0.0022	-0.279	-0.818	0.0005	0.0006	0.0026
PPTC7	0.3376	0.0027	0.0022	-0.17	-1.085	0.002	0.0022	0.0071
PRDX1	-1.733	0.0006	0.0019	-0.121	-1.77	0.0009	0.0009	0.0115
PRKACB	-0.265	0.0026	0.0031	-0.419	-0.585	0.0012	0.0016	0.0043
PROSER1	0.0079	0.0037	0.0036	0.0061	-1.058	0.0023	0.0022	0.0062
PRPF38B	-0.448	0.0016	0.0022	-0.669	-1.233	0.0006	0.001	0.009
PRSS35	-0.077	0.0015	0.0016	-0.03	-0.632	0.0018	0.0018	0.0037
PSIP1	0.321	0.0013	0.001	0.5782	-0.905	0.0009	0.0006	0.0039
PSMA3	-0.777	0.0009	0.0016	-0.575	-0.699	0.0008	0.0011	0.0044
PSMA5	-0.005	0.007	0.0071	0.5786	-0.788	0.0046	0.0031	0.0081
PSMC6	-1.396	0.0009	0.0024	-1.361	-1.003	0.0004	0.0011	0.005
PSME3	0.3204	0.0051	0.0041	-0.169	-0.91	0.0035	0.004	0.0129
PSMG2	-0.622	0.0008	0.0013	-1.706	-1.495	0.0006	0.0019	0.005
PTP4A2	-0.544	0.0026	0.0038	-0.743	-1.136	0.0007	0.0012	0.0055
PTPN2	-0.841	0.0027	0.0049	-0.861	-1.216	0.0008	0.0014	0.0031
PUS10	0.3529	0.0017	0.0014	0.3177	-0.665	0.0014	0.0011	0.0045
R3HDML	-0.555	0.0029	0.0043	-0.691	-1.099	0.0019	0.0031	0.0066
RAB6A	0.2155	0.0015	0.0013	-0.655	-1.052	0.0005	0.0007	0.0017
RALGAPA2	0.5454	0.0046	0.0032	-0.083	-1.377	0.0015	0.0016	0.0052
RALGAPB	-0.661	0.0063	0.01	0.0042	-1.163	0.0013	0.0013	0.0035
RAP1GDS1	-0.998	0.0015	0.003	-0.376	-0.768	0.0008	0.001	0.0039
RAPGEF2	-1.069	0.0008	0.0017	-0.477	-1.073	0.0011	0.0016	0.004
RASA2	-0.111	0.0034	0.0036	0.2677	-0.694	0.0009	0.0007	0.0038
RASIP1	0.1565	0.0056	0.005	-0.244	-0.845	0.0024	0.0029	0.0054
RASSF6	-0.826	0.0019	0.0033	0.0468	-1.031	0.0017	0.0016	0.0071
RBBP4	-0.863	0.0024	0.0044	-0.195	-0.711	0.0008	0.0009	0.0057
RBFA	-0.844	0.0014	0.0025	-2.198	-1.015	0.0008	0.0036	0.0049
RBM15	-0.696	0.0064	0.0104	-1.512	-0.886	0.0017	0.0047	0.0095
RBM22	0.1626	0.0027	0.0024	0.4276	-1.305	0.003	0.0023	0.006
RBM27	-0.192	0.0028	0.0032	-1.064	-0.666	0.0005	0.0011	0.0036
RBM39	-0.431	0.0026	0.0035	-0.682	-1.16	0.0008	0.0012	0.0153

RBM47	-0.844	0.0014	0.0025	-0.25	-0.786	0.0013	0.0015	0.0073
RCOR1	-0.793	0.0015	0.0026	0.1958	-0.725	0.001	0.0009	0.0047
REEP5	0.0582	0.0062	0.0059	-1.048	-1.543	0.0013	0.0027	0.0052
REL	0.1183	0.003	0.0027	0.0634	-0.621	0.0009	0.0009	0.004
RFFL	0.0309	0.0024	0.0024	0.4622	-0.653	0.0021	0.0015	0.0055
RHPN2	0.4934	0.003	0.0021	-0.353	-1.83	0.0012	0.0016	0.0041
RIOK3	-1.194	0.0007	0.0016	0.2373	-1.191	0.0011	0.0009	0.0045
RLF	0.3052	0.0087	0.0071	0.3571	-0.902	0.0008	0.0006	0.0027
RLIM	-0.969	0.0005	0.0009	-0.97	-0.723	0.0002	0.0004	0.0029
RMI1	-0.217	0.0019	0.0022	-0.866	-1.173	0.0011	0.0021	0.0058
RNF103	-0.233	0.0029	0.0034	0.1784	-1.15	0.0015	0.0013	0.0056
RNF13	-0.862	0.0033	0.0059	-0.054	-0.658	0.001	0.0011	0.0034
RNF138	-0.899	0.0043	0.008	-0.698	-0.698	0.0009	0.0015	0.0031
RNF139	-0.108	0.0023	0.0025	0.1153	-0.874	0.0014	0.0013	0.0056
RNF14	-0.884	0.002	0.0038	-1.02	-1.283	0.0009	0.0019	0.0052
RNF19A	-0.578	0.0046	0.0069	-0.382	-0.634	0.0011	0.0015	0.0047
RNF213	-0.104	0.0071	0.0077	0.4876	-0.93	0.0051	0.0036	0.013
RPAP1	-0.126	0.0041	0.0044	0.3627	-0.786	0.0069	0.0054	0.0098
RPF1	-0.233	0.0023	0.0027	-0.473	-1.195	0.0015	0.0022	0.009
RPL17	-0.9	0.0031	0.0059	-0.168	-2.384	0.0041	0.0046	0.005
RPP14	-0.31	0.0017	0.0022	-0.225	-1.302	0.0019	0.0022	0.0069
RPP21	0.2354	0.007	0.006	-0.021	-1.077	0.0088	0.0089	0.0109
RPRD2	-0.484	0.0024	0.0034	-0.424	-0.907	0.0013	0.0017	0.0077
RPS6KA1	0.2938	0.0038	0.0031	-0.378	-1.868	0.002	0.0027	0.0079
RPS6KB1	-0.204	0.002	0.0023	-0.182	-0.648	0.001	0.0011	0.0066
RSBN1	-1.384	0.0012	0.0032	-0.221	-0.736	0.0011	0.0013	0.0059
RUNDC3B	-0.187	0.0096	0.011	-0.789	-1.393	0.0021	0.0036	0.007
SAP130	0.3922	0.022	0.0168	-0.279	-0.953	0.0011	0.0013	0.005
SASS6	0.3938	0.001	0.0008	0.0979	-0.961	0.0013	0.0012	0.004
SBNO1	-0.317	0.0026	0.0032	-0.262	-0.644	0.0011	0.0013	0.0082
SCAF11	-0.464	0.0026	0.0035	-0.787	-0.765	0.0005	0.0009	0.0054
SCARB2	0.2045	0.0056	0.0049	0.5438	-0.965	0.0023	0.0016	0.0044
SDAD1	0.4697	0.0046	0.0034	-0.253	-0.963	0.002	0.0023	0.0081
SDCBP	0.0306	0.0015	0.0015	-0.436	-1.646	0.0006	0.0009	0.0037
SEC11C	-1.241	0.0014	0.0033	-0.129	-0.782	0.0016	0.0017	0.0055
SEC14L1	-0.167	0.004	0.0044	-0.379	-0.853	0.0031	0.004	0.0147
SEH1L	-0.77	0.0018	0.0031	-0.804	-1.426	0.001	0.0018	0.0048
SELENBP1	0.3667	0.0089	0.0069	0.5728	-1.773	0.0079	0.0053	0.0079
SEMA3E	-1.222	0.0027	0.0064	-1.652	-2.315	0.001	0.0031	0.0038
SEMA6B	0.3584	0.1235	0.0964	0.5003	-1.305	0.0414	0.0292	0.0102
SENP6	-0.162	0.0008	0.0009	0.1194	-0.643	0.0007	0.0006	0.0034
SERINC3	-0.667	0.0022	0.0034	-0.825	-1.342	0.0013	0.0022	0.0056
SERPINB1A	-0.291	0.0014	0.0017	-1.19	-1.133	0.0007	0.0016	0.003
SERPINE1	0.2296	0.0036	0.0031	-0.002	-2.57	0.0028	0.0028	0.0108
SETD1B	0.0308	0.0022	0.0022	-0.049	-1.378	0.0032	0.0034	0.0091
SGMS2	-0.798	0.001	0.0018	-1.554	-1.371	0.0009	0.0025	0.0158
SH3BGRL2	-0.292	0.0009	0.0011	0.2042	-0.623	0.0014	0.0012	0.005
SH3D19	-1.179	0.0019	0.0042	-0.508	-0.983	0.0011	0.0016	0.0049

SH3GLB1	-0.128	0.0025	0.0027	-0.982	-1.087	0.0011	0.0021	0.0083
SH3RF1	-0.047	0.0021	0.0022	0.0076	-0.644	0.0013	0.0013	0.006
SHPRH	-0.97	0.0012	0.0023	-0.206	-0.885	0.0006	0.0007	0.0023
SIAH2	-0.277	0.0061	0.0074	-0.189	-1.123	0.0034	0.0039	0.0049
SIGLEC15	-0.284	0.0026	0.0032	-1.544	-1.65	0.0022	0.0064	0.0037
SKIL	-0.7	0.0024	0.0039	-0.049	-0.586	0.0013	0.0014	0.0076
SKP1A	-1.892	0.0011	0.0041	-0.18	-0.644	0.0012	0.0014	0.0057
SLC12A2	-0.729	0.0015	0.0025	-0.901	-1.462	0.0007	0.0012	0.0083
SLC1A5	-0.414	0.0035	0.0047	-0.508	-1.294	0.002	0.0028	0.0064
SLC20A1	-0.415	0.0034	0.0045	-0.943	-1.064	0.0015	0.0029	0.0165
SLC25A24	-0.258	0.001	0.0013	-0.651	-0.76	0.0009	0.0014	0.0077
SLC25A40	-1.28	0.003	0.0073	-0.828	-1.382	0.002	0.0035	0.0087
SLC26A2	-0.528	0.0021	0.003	-0.702	-3.543	0.0015	0.0025	0.0062
SLC35A4	-0.989	0.0021	0.0042	-1.032	-0.81	0.0025	0.0052	0.0139
SLC39A6	0.2839	0.0036	0.003	-0.696	-1.139	0.0013	0.0021	0.0045
SLC4A7	-0.669	0.0009	0.0015	-0.107	-0.944	0.0006	0.0006	0.0035
SLC4A9	-0.276	0.0032	0.0039	0.519	-1.159	0.0048	0.0034	0.0043
SLC52A3	0.3481	0.0185	0.0146	0.3829	-1.002	0.0125	0.0096	0.0053
SLC6A6	-1.116	0.0049	0.0106	-0.53	-0.608	0.0024	0.0034	0.0142
SLC7A1	-0.367	0.0048	0.0061	-0.539	-1.162	0.0018	0.0027	0.0079
SLC7A11	-0.233	0.0012	0.0014	-0.337	-1.358	0.001	0.0012	0.005
SLMAP	-1.845	0.0007	0.0025	-0.698	-1.566	0.0008	0.0013	0.0038
SMAD4	-0.777	0.0016	0.0028	-1.019	-1.129	0.0006	0.0013	0.0027
SMARCC1	0.4623	0.0016	0.0011	-0.228	-0.618	0.001	0.0012	0.0046
SMC4	-0.79	0.0017	0.003	-0.691	-0.78	0.0009	0.0014	0.0086
SMG1	-0.172	0.0023	0.0026	-0.157	-0.776	0.0006	0.0007	0.0038
SMG5	-0.546	0.0034	0.005	0.2066	-1.375	0.004	0.0034	0.0118
SMIM1	0.0152	0.0054	0.0053	-0.498	-1.5	0.0038	0.0054	0.0056
SMIM3	-0.554	0.0019	0.0027	-0.57	-1.289	0.0015	0.0023	0.0053
SNCG	-1.345	0.004	0.0102	-1.186	-1.541	0.0029	0.0067	0.005
SNRNP40	-1.069	0.0008	0.0017	0.3453	-0.589	0.0012	0.0009	0.0056
SNX16	-0.069	0.0024	0.0026	-0.373	-0.88	0.001	0.0013	0.004
SNX27	-0.002	0.0026	0.0026	-0.078	-0.757	0.0013	0.0013	0.0045
SNX5	-0.879	0.0024	0.0044	0.1413	-0.925	0.0016	0.0014	0.009
SNX6	0.321	0.0013	0.001	-0.232	-0.76	0.0006	0.0007	0.004
SP1	-0.028	0.007	0.0071	-0.236	-0.981	0.0015	0.0017	0.0081
SPATA24	0.4891	0.0098	0.007	0.5695	-1.699	0.0175	0.0118	0.0038
SPIRE1	0.4743	0.002	0.0014	-0.187	-0.715	0.0012	0.0013	0.0034
SPP1	-0.729	0.0015	0.0025	-0.309	-1.29	0.0009	0.0011	0.0093
SPPL2A	0.3673	0.0028	0.0022	0.1745	-0.693	0.0015	0.0013	0.0045
SPRR1A	0.2019	0.001	0.0009	-0.749	-2.644	0.0013	0.0021	0.003
SPRY1	-0.586	0.0009	0.0013	0.1673	-2.041	0.0016	0.0015	0.0131
SPRY4	-1.31	0.0017	0.0043	-1.5	-1.236	0.0008	0.0024	0.0115
SQLE	-0.076	0.003	0.0032	0.0305	-0.933	0.0018	0.0018	0.0088
SREBF2	0.3787	0.0081	0.0063	-0.137	-1.688	0.0031	0.0034	0.0087
SRFBP1	-1.056	0.0009	0.0019	-0.882	-0.943	0.0005	0.0008	0.0028
SRI	-0.006	0.0045	0.0046	-0.101	-1.692	0.0047	0.0051	0.018
SRP19	-0.595	0.0041	0.0061	-0.627	-2.238	0.0017	0.0026	0.0021

SRRM2	-0.336	0.0022	0.0028	-1.02	-0.822	0.0009	0.0018	0.0187
SRSF10	0.0948	0.0013	0.0013	-0.681	-1.116	0.0003	0.0004	0.0036
SS18	-0.743	0.0018	0.003	-1.141	-0.824	0.0005	0.0011	0.004
SSB	-0.331	0.0008	0.001	-0.629	-0.681	0.0006	0.001	0.004
ST3GAL6	-0.648	0.0012	0.0018	0.0447	-1.29	0.0013	0.0013	0.01
STAG2	-0.555	0.0005	0.0007	0.0612	-0.591	0.0003	0.0003	0.0016
STARD4	-1.631	0.001	0.0032	-0.733	-1.647	0.0008	0.0013	0.0032
STC2	0.1902	0.0078	0.0068	0.1606	-1.005	0.0038	0.0034	0.0081
STEAP1	-0.744	0.0295	0.0494	-1.354	-1.768	0.0035	0.009	0.0121
STEAP2	-0.909	0.0057	0.0107	-1.04	-1.535	0.0041	0.0084	0.022
STK4	0.0308	0.0035	0.0035	-0.039	-0.656	0.0018	0.0019	0.0054
STT3B	-0.121	0.001	0.0011	0.1919	-0.786	0.0006	0.0006	0.0034
SULT1C2	-0.192	0.0014	0.0016	0.3008	-0.976	0.0018	0.0014	0.004
SUOX	0.5336	0.004	0.0027	0.3376	-1.374	0.0041	0.0032	0.0105
SUZ12	-0.597	0.0013	0.0019	0.1118	-1.055	0.0009	0.0009	0.005
TAF13	-1.12	0.004	0.0086	-0.751	-0.96	0.0022	0.0036	0.0044
TAF9	-0.484	0.0016	0.0023	-2.293	-1.226	0.0004	0.0018	0.0043
TARDBP	-0.445	0.0013	0.0018	-0.41	-0.817	0.0007	0.0009	0.0106
TBC1D31	-0.969	0.0014	0.0027	-0.157	-1.056	0.0021	0.0023	0.0061
TCOF1	-1.402	0.0035	0.0092	-1.159	-1.238	0.0016	0.0036	0.0084
TET2	-0.648	0.0012	0.0018	-0.591	-0.971	0.001	0.0015	0.0061
TET3	-0.686	0.0067	0.0108	-0.483	-0.879	0.0023	0.0032	0.0094
TFAM	-0.484	0.0053	0.0074	0.0661	-1.883	0.0011	0.0011	0.0019
TGOLN1	-1.627	0.0015	0.0047	-0.192	-1.34	0.0029	0.0033	0.0074
THUMPD3	-0.192	0.0035	0.004	0.3786	-0.943	0.002	0.0015	0.0076
TIPARP	0.4095	0.003	0.0023	0.1235	-1.099	0.0008	0.0008	0.0068
TIPIN	0.0853	0.0016	0.0015	0.3405	-0.787	0.0013	0.001	0.0037
TM4SF4	0.5685	0.0026	0.0018	-0.337	-2.308	0.0013	0.0017	0.0219
TM9SF2	-0.86	0.0028	0.0051	0.3483	-0.837	0.0011	0.0009	0.0032
TMCO6	-1.303	0.0013	0.0033	-0.993	-1.573	0.0014	0.0028	0.0072
TMED7	0.1424	0.0023	0.0021	-0.217	-1.189	0.001	0.0011	0.005
TMEM14A	-1.286	0.0034	0.0084	-0.431	-1.257	0.0027	0.0037	0.0044
TMEM167E	-0.371	0.0062	0.008	0.2419	-2.491	0.0085	0.0072	0.0036
TMEM184C	0.1018	0.006	0.0056	-0.038	-1.353	0.0015	0.0015	0.0028
TMEM243	-0.686	0.0057	0.0092	-0.324	-0.638	0.0062	0.0077	0.0157
TMEM253	-0.616	0.0013	0.002	-1.258	-0.929	0.0011	0.0026	0.0105
TMEM29	0.4446	0.0005	0.0003	0.2719	-1.099	0.0004	0.0004	0.0009
TMEM37	-0.213	0.012	0.0139	-0.46	-0.874	0.0071	0.0098	0.0094
TMEM51	-0.126	0.003	0.0033	-0.031	-0.84	0.0015	0.0015	0.0046
TMEM79	-0.431	0.004	0.0054	0.2719	-1.392	0.0047	0.0039	0.0136
TNFAIP8	-1.191	0.0014	0.0032	-0.533	-0.858	0.001	0.0014	0.0036
TNFRSF10E	-0.016	0.0017	0.0018	0.409	-1.621	0.0019	0.0014	0.0056
TNFRSF21	0.2296	0.0036	0.0031	-0.335	-1.698	0.0014	0.0017	0.0052
TOB1	0.1129	0.0042	0.0039	-0.876	-2.385	0.0021	0.0038	0.0098
TOP1MT	-0.473	0.0256	0.0355	-0.078	-0.688	0.0053	0.0056	0.0094
TPD52	-0.512	0.0027	0.0038	-0.32	-1.233	0.0021	0.0026	0.0056
TRAPPC8	-0.464	0.0026	0.0035	-0.773	-0.965	0.0007	0.0012	0.0029
TRF	-0.418	0.0135	0.0181	-0.143	-1.238	0.0081	0.009	0.0035

TRIM25	-0.232	0.0035	0.0041	-0.999	-1.589	0.0016	0.0032	0.0105
TRIM33	-1.968	0.001	0.0041	-0.42	-0.851	0.0008	0.0011	0.0045
TRMT10A	-0.801	0.0019	0.0032	-0.786	-0.786	0.0019	0.0032	0.0066
TRMT13	-0.602	0.0012	0.0018	0.1222	-0.623	0.0013	0.0012	0.0055
TRMT2B	0.2707	0.0015	0.0013	-0.079	-0.862	0.0005	0.0005	0.002
TRP53INP1	-0.526	0.002	0.0028	0.3708	-0.662	0.0019	0.0015	0.0037
TSC22D2	-0.597	0.0026	0.0039	-0.429	-0.767	0.0007	0.001	0.004
TTC14	-0.114	0.006	0.0065	0.2719	-0.591	0.0019	0.0015	0.004
TTF2	-1.211	0.0019	0.0044	-1.022	-1.01	0.0015	0.0031	0.007
TTLL7	-0.841	0.0027	0.0049	-0.084	-0.608	0.0017	0.0018	0.0046
UBA3	-0.809	0.0011	0.0019	-0.187	-0.91	0.0008	0.0009	0.0037
UBE2D2A	-1.372	0.0016	0.0042	-1.192	-1.053	0.0009	0.0021	0.0042
UBE2D3	-0.72	0.0026	0.0042	-0.706	-0.934	0.0008	0.0014	0.0088
UBN2	-0.454	0.0017	0.0024	0.5268	-0.701	0.0013	0.0009	0.0041
UGDH	0.1398	0.0024	0.0022	-0.932	-2.2	0.0012	0.0023	0.0114
UNC5B	-0.254	0.0019	0.0022	-0.903	-1.305	0.0015	0.0028	0.0078
UPK3BL	-0.429	0.0051	0.0069	-0.049	-0.809	0.0052	0.0054	0.0062
URGCP	-0.384	0.0026	0.0034	-0.049	-0.867	0.0021	0.0022	0.0075
USP10	-0.223	0.003	0.0035	-0.347	-0.82	0.0015	0.0019	0.0056
USP33	-2.167	0.0007	0.0031	-0.326	-1.054	0.0015	0.0019	0.0075
USP53	-0.699	0.0026	0.0042	-0.324	-1.187	0.0012	0.0015	0.0046
VCAM1	-0.192	0.0014	0.0016	-0.384	-1.394	0.001	0.0012	0.0034
VMA21	-0.383	0.001	0.0014	-0.622	-0.635	0.0006	0.0009	0.002
VMP1	-0.062	0.0017	0.0018	-0.518	-0.704	0.0011	0.0015	0.0106
VPS13D	-0.233	0.0012	0.0014	0.422	-0.812	0.0011	0.0008	0.0051
VPS26A	-0.597	0.0013	0.0019	-0.802	-0.906	0.0007	0.0012	0.0035
WAC	-1.212	0.0006	0.0015	-0.93	-0.669	0.0004	0.0008	0.0036
WAPL	-1.325	0.002	0.0049	-0.817	-1.136	0.0004	0.0007	0.0021
WDFY3	-0.002	0.0026	0.0026	-0.061	-0.732	0.0012	0.0012	0.0054
WWC1	-0.233	0.0017	0.002	-0.023	-0.777	0.0017	0.0017	0.007
XPOT	-0.362	0.0028	0.0036	-0.606	-0.972	0.001	0.0015	0.0065
YAE1D1	-0.454	0.0006	0.0008	-1.331	-1.112	0.0004	0.0009	0.0029
YIPF5	-0.145	0.0036	0.004	0.5075	-1.473	0.0017	0.0012	0.0024
ZBTB7C	-1.471	0.031	0.086	-2.788	-0.724	0.0027	0.0185	0.004
ZC3H15	0.4179	0.002	0.0015	0.2889	-0.959	0.0011	0.0009	0.004
ZDHHC9	-0.498	0.001	0.0015	0.2234	-1.258	0.0005	0.0005	0.0022
ZFP11	-0.908	0.0035	0.0065	-0.203	-0.616	0.0026	0.003	0.0074
ZFP236	-2.179	0.0009	0.0042	-0.621	-1.023	0.0011	0.0017	0.0049
ZFP260	0.447	0.0014	0.001	0.1179	-0.685	0.0009	0.0009	0.0045
ZFP280B	0.3718	0.0022	0.0017	-0.174	-1.031	0.0015	0.0017	0.0042
ZFP334	-0.645	0.0042	0.0065	-0.961	-0.799	0.0028	0.0055	0.0062
ZFP36	-0.802	0.0024	0.0042	-0.788	-0.84	0.0024	0.0042	0.016
ZFP59	-0.729	0.0015	0.0025	-0.185	-1.765	0.0013	0.0015	0.0027
ZFP672	-1.51	0.0013	0.0036	0.5003	-0.769	0.0037	0.0026	0.007
ZFP697	0.0878	0.003	0.0028	0.2421	-1.09	0.0023	0.002	0.0043
ZFP706	-0.121	0.0021	0.0023	-0.344	-0.645	0.0009	0.0012	0.0109
ZFP87	0.3636	0.002	0.0015	0.0728	-0.912	0.0012	0.0011	0.003
ZFP871	-1.107	0.0023	0.005	0.4925	-1.066	0.0021	0.0015	0.0054

ZFPM1	0.4747	0.0088	0.0063	0.0101	-1.398	0.0035	0.0035	0.01
ZGRF1	-1.586	0.0017	0.0052	-0.109	-0.837	0.0017	0.0018	0.0039
ZIC3	0.4325	0.0359	0.0266	-0.105	-4.142	0.0218	0.0235	0.0001
ZRANB2	-0.232	0.0055	0.0065	-0.444	-1.096	0.0023	0.0032	0.0065
ZSCAN12	-0.302	0.0016	0.0019	0.457	-0.946	0.0016	0.0012	0.0022
ZZZ3	-0.106	0.0035	0.0038	-0.279	-0.884	0.0012	0.0015	0.0065
)0066M21I	#DIV/0!	0.002	0	4.844	-1.282	0.0025	######	0.0022
AMOT	#DIV/0!	0.0003	0	0.4709	-0.921	0.0003	0.0002	0.001
GM14288	#DIV/0!	0.0001	0	2.0351	-0.977	######	######	######
RNF128	#NUM!	0	0.0005	1.5166	-1.377	0.0004	0.0002	0.0011
SERINC1	#DIV/0!	0.0012	0	1.0296	-0.62	0.0005	0.0002	0.0026
SRP54B	#NUM!	0	0.0001	0.8782	-0.747	######	######	0.0003
UTY	#DIV/0!	0	0	-3.967	-7.128	######	0.0002	######

GB_K36_WT	Pvalue	logFC_KO/WT	KO_1	KO_2	KO_3	WT_1	WT_2	WT_3
0.0971	0.000967	-1.4	49	33	41	108	123	115.5
0.0039	0.000188	-6.78	0	0	0	27	16	21.5
0.0343	6.47E-08	-0.7	781	656	718.5	1273	1231	1252
0.006	0.01911	-1.49	47	8	27.5	70	92	81
0.0117	4.21E-08	-1.18	179	190	184.5	453	445	449
0.022	0.034551	-1.27	16	32	24	73	52	62.5
0.0102	7.49E-14	-4.28	19	43	31	623	688	655.5
0.0076	0.000939	-0.88	128	171	149.5	285	310	297.5
0.01	3.74E-10	-2.49	24	35	29.5	163	197	180
0.0079	0.00117	-4.6	2	0	1	29	22	25.5
0.031	3.88E-28	-0.9	2427	2059	2243	4423	4544	4484
0.019	1.69E-08	-0.68	882	857	869.5	1516	1472	1494
0.0355	3.62E-266	-2.43	4320	3852	4086	21153	26376	23765
0.0295	5.18E-18	-0.63	3493	3365	3429	5338	6099	5719
0.0092	1.40E-43	-1.46	840	718	779	2206	2387	2297
0.0245	0.011076	-5.58	0	0	0	8	11	9.5
0.0192	1.82E-21	-1.09	1122	990	1056	2081	2747	2414
0.0143	1.43E-08	-1.04	261	271	266	554	628	591
0.0251	3.21E-10	-0.85	696	555	625.5	1200	1208	1204
0.0196	1.57E-06	-1.39	91	80	85.5	238	244	241
0.0141	2.19E-12	-0.68	1527	1336	1432	2460	2465	2463
0.0036	1.70E-100	-1.98	907	905	906	3679	3985	3832
0.0164	9.17E-15	-2.49	39	58	48.5	264	328	296
0.0043	0.010974	-0.88	71	78	74.5	147	149	148
0.0104	9.91E-56	-1.52	1241	1069	1155	3199	3912	3556
0.0062	1.33E-63	-4.73	29	20	24.5	655	740	697.5
0.0084	8.88E-29	-0.95	1890	1631	1761	3418	3861	3640
0.0172	3.32E-15	-0.66	3930	3286	3608	5446	6821	6134
0.015	2.91E-37	-1.1	1801	1817	1809	4031	4286	4159
0.0103	2.23E-14	-0.84	1227	983	1105	1938	2309	2124
0.0211	9.31E-08	-0.72	1563	1071	1317	1996	2656	2326
0.039	4.72E-22	-0.9	3389	3006	3198	5469	7377	6423
0.0292	6.44E-62	-1.68	802	758	780	2601	2752	2677
0.016	8.17E-10	-0.88	533	594	563.5	1079	1159	1119
0.0278	2.41E-11	-0.64	1650	1356	1503	2401	2636	2519
0.0126	1.65E-21	-1.1	723	678	700.5	1545	1689	1617
0.0075	7.90E-75	-2.11	717	753	735	2972	3889	3431
0.0302	1.57E-26	-0.85	3910	3094	3502	6375	7145	6760
0.0066	5.25E-13	-0.96	734	556	645	1280	1410	1345
0.026	1.60E-17	-0.69	3408	2736	3072	5031	5581	5306
0.0209	2.37E-40	-0.92	3948	3721	3835	7225	8419	7822
0.035	1.03E-175	-3.18	432	510	471	4241	4964	4603
0.0197	5.92E-135	-1.58	6716	6814	6765	20862	22645	21754
0.0351	1.26E-193	-1.92	3907	3869	3888	15310	16260	15785

0.0052	8.90E-44	-1.32	1132	1088	1110	2943	3029	2986
0.0077	1.56E-21	-0.68	3686	3185	3436	5583	6227	5905
0.0064	6.28E-215	-2.75	1628	1329	1479	9401	12031	10716
0.0207	7.11E-101	-2.25	594	586	590	3008	3039	3024
0.0438	7.43E-31	-2.38	114	114	114	607	666	636.5
0.0125	2.11E-12	-0.59	2172	1966	2069	3110	3586	3348
0.0073	2.22E-72	-2.27	435	346	390.5	1993	2028	2011
0.005	5.00E-42	-1.45	938	988	963	2595	3084	2840
0.0248	2.21E-06	-0.66	912	651	781.5	1205	1448	1327
0.0112	0.01718	-1.65	14	12	13	39	49	44
0.0102	0.041533	-4.06	0	8	4	88	60	74
0.0162	8.68E-63	-2.86	143	178	160.5	1220	1297	1259
0.0066	4.15E-12	-1.54	141	145	143	435	457	446
0.0098	1.98E-17	-1.43	305	245	275	743	850	796.5
0.0068	8.10E-21	-1.12	763	689	726	1721	1662	1692
0.0046	0.014353	-1.12	57	30	43.5	104	96	100
0.0131	8.06E-11	-0.63	1464	1327	1396	2138	2512	2325
0.0127	8.93E-32	-0.9	3427	3298	3363	6748	6679	6714
0.0125	6.57E-24	-1.2	938	713	825.5	1879	2184	2032
0.0147	1.23E-44	-1.3	2089	1798	1944	4492	5776	5134
0.0275	2.10E-65	-0.97	13975	11883	12929	26165	28029	27097
0.0062	2.74E-21	-1	1301	1330	1316	2883	2753	2818
0.0134	5.31E-64	-0.91	17081	15059	16070	31718	33085	32402
0.0385	8.00E-45	-1.07	2770	2329	2550	5464	5985	5725
0.0067	1.01E-15	-1.08	543	479	511	1129	1182	1156
0.0058	0.012858	-3	4	1	2.5	23	19	21
0.0108	2.80E-06	-0.92	338	232	285	528	629	578.5
0.013	1.48E-147	-2.17	1314	1093	1204	5407	6199	5803
0.0085	8.21E-12	-0.96	560	625	592.5	1207	1269	1238
0.019	3.13E-16	-0.91	922	794	858	1664	1788	1726
0.0072	1.31E-14	-0.95	737	786	761.5	1478	1704	1591
0.0189	8.50E-21	-0.86	1607	1626	1617	3036	3269	3153
0.0058	5.49E-09	-0.71	983	1004	993.5	1799	1681	1740
0.0136	6.48E-41	-1.16	1885	1946	1916	4335	4868	4602
0.005	1.49E-06	-1.21	151	235	193	506	460	483
0.0386	0.0001	-1.06	158	101	129.5	263	315	289
0.0126	6.46E-06	-4.05	5	1	3	48	58	53
0.0069	6.26E-09	-0.76	678	628	653	1072	1318	1195
0.0166	2.49E-11	-5.74	2	2	2	92	140	116
0.0151	2.21E-14	-11.2	0	0	0	419	494	456.5
0.019	0.002181	-0.82	189	133	161	335	268	301.5
0.0097	1.16E-62	-1.43	1438	1269	1354	3781	4025	3903
0.0062	1.56E-06	-0.75	444	441	442.5	803	793	798
0.0125	7.73E-50	-1.29	2051	1629	1840	4629	5012	4821
0.0072	2.44E-23	-1.07	1470	1111	1291	2872	2912	2892
0.0098	4.30E-62	-1.52	1316	1357	1337	4054	4218	4136
0.007	2.00E-14	-0.72	1665	1417	1541	2515	2955	2735
0.0075	1.27E-11	-1.14	306	322	314	712	779	745.5

0.0216	6.19E-22	-0.69	3160	2891	3026	5019	5461	5240
0.0088	9.61E-09	-1	307	331	319	650	721	685.5
0.0053	8.80E-07	-1.43	101	196	148.5	401	472	436.5
0.0115	1.19E-16	-1.29	475	344	409.5	994	1143	1069
0.0028	1.04E-06	-1.49	71	90	80.5	247	241	244
0.0178	4.73E-27	-0.89	2164	2115	2140	4167	4330	4249
0.0216	6.55E-40	-2.25	284	186	235	1123	1259	1191
0.0078	1.10E-07	-1.15	240	164	202	459	497	478
0.0108	2.51E-26	-1.26	789	859	824	1950	2298	2124
0.0094	4.27E-15	-1.2	373	349	361	845	932	888.5
0.0688	3.45E-58	-1.1	8318	7136	7727	15975	19530	17753
0.008	2.05E-09	-8.77	1	0	0.5	227	242	234.5
0.0071	3.12E-27	-0.79	3742	3342	3542	6023	7167	6595
0.008	1.21E-23	-1.83	234	180	207	707	875	791
0.0138	0.000501	-2.92	12	1	6.5	49	54	51.5
0.0206	2.09E-09	-1.79	72	68	70	226	297	261.5
0.0115	4.29E-11	-0.86	621	549	585	1110	1174	1142
0.0101	6.06E-54	-1.51	1367	1266	1317	3540	4524	4032
0.0072	1.52E-05	-0.76	415	511	463	745	953	849
0.0073	8.80E-26	-1.96	193	201	197	708	948	828
0.0189	0.002139	-0.6	356	259	307.5	489	506	497.5
0.0473	1.30E-64	-1.65	1237	970	1104	3504	3918	3711
0.0127	2.18E-09	-0.85	597	685	641	1167	1322	1245
0.014	1.23E-08	-0.6	1128	1104	1116	1776	1872	1824
0.0108	0.00033	-0.63	381	317	349	545	610	577.5
0.1635	3.09E-179	-1.84	2943	2651	2797	10412	11111	10762
0.0097	4.07E-05	-0.88	289	192	240.5	433	517	475
0.0086	3.08E-07	-0.75	487	458	472.5	804	907	855.5
0.0152	3.70E-92	-1.19	6645	6270	6458	14953	16838	15896
0.0086	5.91E-18	-0.82	1922	1556	1739	3010	3604	3307
0.007	9.20E-12	-0.87	897	682	789.5	1532	1550	1541
0.0122	0.047265	-1.16	23	24	23.5	44	70	57
0.0071	3.06E-41	-1.23	2358	2246	2302	5062	6621	5842
0.0083	3.60E-45	-1.25	1892	1825	1859	4814	4644	4729
0.0089	3.43E-11	-0.87	625	543	584	1095	1193	1144
0.0059	1.34E-18	-1.09	1255	1575	1415	3038	3472	3255
0.0202	5.36E-43	-1.51	752	775	763.5	2195	2475	2335
0.0081	0.000607	-0.6	398	396	397	675	612	643.5
0.0143	0.000184	-0.59	543	421	482	735	814	774.5
0.0088	7.51E-14	-1.45	206	210	208	623	595	609
0.0319	1.06E-53	-1.21	2054	1911	1983	4595	5277	4936
0.0151	1.81E-102	-1.6	3911	4054	3983	12815	13070	12943
0.0099	1.44E-84	-1.41	3276	2943	3110	8898	8823	8861
0.0053	5.27E-63	-1.77	704	617	660.5	2301	2536	2419
0.0068	2.24E-11	-0.6	1909	1634	1772	2798	2950	2874
0.0141	1.64E-10	-0.73	914	806	860	1440	1618	1529
0.006	5.01E-30	-1.57	414	356	385	1174	1286	1230
0.0069	9.46E-65	-2.83	173	133	153	1134	1201	1168

0.0088	4.27E-09	-0.63	1219	1006	1113	1777	1914	1846
0.0064	3.36E-11	-0.68	2373	2736	2555	4073	4781	4427
0.004	2.11E-73	-2.22	391	384	387.5	1893	1996	1945
0.0038	6.69E-09	-0.89	430	421	425.5	850	844	847
0.0251	1.82E-37	-1.19	1417	1188	1303	3093	3260	3177
0.0032	9.15E-06	-1.07	173	139	156	305	398	351.5
0.0158	9.59E-41	-1.27	1964	2185	2075	5284	5473	5379
0.0864	0	-3.23	4003	3806	3905	37766	41059	39413
0.0357	9.42E-150	-2.16	1630	1320	1475	7002	7145	7074
0.0167	0.001889	-6.16	0	0	0	17	11	14
0.0203	2.52E-18	-0.72	2062	1915	1989	3344	3678	3511
0.037	1.37E-90	-1.3	4435	4204	4320	10655	12243	11449
0.0053	1.08E-10	-0.59	2384	2575	2480	3811	4266	4039
0.0134	3.47E-19	-1.13	601	527	564	1270	1386	1328
0.0099	1.05E-24	-2.24	113	101	107	527	554	540.5
0.0088	6.50E-10	-1.07	307	274	290.5	645	664	654.5
0.0033	4.32E-06	-1.26	132	111	121.5	350	269	309.5
0.0091	0.005964	-1.06	78	46	62	120	157	138.5
0.0145	8.11E-17	-0.64	3149	2670	2910	4583	5131	4857
0.008	2.20E-10	-0.65	1312	1123	1218	2020	2078	2049
0.0062	0.00226	-3.22	3	3	3	40	19	29.5
0.0222	3.29E-38	-1.38	824	758	791	2044	2391	2218
0.0037	1.03E-87	-3.02	217	158	187.5	1498	1763	1631
0.0163	1.35E-40	-1.17	1673	1690	1682	3977	4159	4068
0.0059	0.000374	-0.75	222	262	242	417	464	440.5
0.0136	0.041266	-3.51	2	0	1	12	12	12
0.0062	6.11E-17	-0.94	944	790	867	1659	1922	1791
0.0083	6.70E-66	-2.69	192	231	211.5	1401	1534	1468
0.0048	7.10E-51	-3.85	37	43	40	567	681	624
0.023	4.16E-68	-1.18	3883	3816	3850	8955	9783	9369
0.011	2.87E-22	-0.84	1945	1795	1870	3292	3909	3601
0.0419	8.46E-21	-0.65	3690	3306	3498	5626	6132	5879
0.0072	1.06E-17	-1.11	564	559	561.5	1232	1371	1302
0.0072	9.31E-19	-1.42	408	305	356.5	914	1133	1024
0.0058	3.81E-06	-0.74	436	387	411.5	667	810	738.5
0.0133	9.49E-45	-1.04	3476	3007	3242	6547	7757	7152
0.0144	0.000303	-6	1	0	0.5	27	42	34.5
0.0068	2.56E-07	-0.83	407	361	384	727	734	730.5
0.0068	0.001243	-0.61	338	268	303	476	514	495
0.0049	4.54E-13	-2.07	104	57	80.5	345	374	359.5
0.1646	1.16E-07	-1.22	148	173	160.5	386	422	404
0.0121	0.001539	-3.94	2	2	2	6	62	34
0.0143	1.25E-75	-1.45	1711	1567	1639	4660	4985	4823
0.0071	2.12E-35	-1.35	1331	1560	1446	3725	4245	3985
0.0098	5.90E-06	-1.26	109	95	102	269	253	261
0.0099	1.40E-106	-2.39	826	967	896.5	4614	5543	5079
0.0117	1.97E-43	-1.21	1987	2087	2037	4943	5169	5056
0.0098	8.20E-44	-1.12	1848	1726	1787	3951	4416	4184

0.0294	4.39E-16	-2.37	70	48	59	293	361	327
0.0801	4.02E-53	-2.37	224	242	233	1187	1413	1300
0.0081	6.88E-06	-0.96	215	260	237.5	438	564	501
0.0071	7.38E-09	-8.52	1	0	0.5	218	174	196
0.0353	6.89E-21	-1.15	634	574	604	1379	1491	1435
0.0679	0.013798	-1.41	26	28	27	105	47	76
0.0054	2.00E-22	-2.37	142	84	113	675	562	618.5
0.0177	5.41E-61	-1.1	3454	3100	3277	7196	7910	7553
0.0113	1.91E-14	-0.79	1174	1149	1162	2117	2186	2152
0.0201	2.10E-08	-0.75	645	540	592.5	1013	1131	1072
0.0193	1.22E-16	-0.92	903	879	891	1681	1948	1815
0.0171	1.25E-27	-1.42	624	478	551	1481	1673	1577
0.053	2.03E-25	-0.91	1848	1877	1863	3581	3970	3776
0.0124	5.05E-12	-0.94	615	668	641.5	1203	1462	1333
0.0098	7.18E-07	-0.6	851	761	806	1207	1420	1314
0.0094	8.85E-05	-0.67	6466	4637	5552	9070	9789	9430
0.0275	1.69E-21	-1.32	506	578	542	1375	1541	1458
0.01	6.91E-37	-1.4	905	751	828	2126	2585	2356
0.004	2.79E-20	-1.27	605	465	535	1256	1508	1382
0.0078	1.81E-51	-1.52	995	838	916.5	2611	3052	2832
0.029	1.32E-10	-1.12	388	277	332.5	719	829	774
0.0217	1.63E-09	-0.75	1030	773	901.5	1502	1757	1630
0.0333	1.76E-09	-0.77	705	598	651.5	1166	1222	1194
0.0185	4.78E-24	-0.72	4213	3579	3896	6433	7361	6897
0.0059	2.36E-61	-1.95	952	721	836.5	3613	3278	3446
0.1024	4.22E-55	-1.03	9980	8378	9179	18374	21977	20176
0.0097	7.53E-10	-0.83	669	748	708.5	1348	1369	1359
0.0032	3.06E-08	-1.31	173	131	152	424	380	402
0.012	2.20E-60	-1.43	1292	1195	1244	3409	3814	3612
0.0099	6.28E-29	-1.21	832	766	799	1853	2124	1989
0.0114	1.11E-161	-4.58	70	98	84	2009	2330	2170
0.0119	5.07E-05	-0.76	312	297	304.5	484	625	554.5
0.027	8.81E-15	-0.88	924	772	848	1634	1721	1678
0.052	4.78E-33	-1.41	658	571	614.5	1732	1776	1754
0.0081	2.24E-06	-1.12	172	256	214	473	533	503
0.0311	6.65E-32	-0.95	1955	1775	1865	3692	4059	3876
0.0061	3.94E-40	-1.01	2444	2152	2298	4698	5209	4954
0.03	2.42E-10	-0.86	565	511	538	1025	1072	1049
0.0165	8.01E-47	-1.32	1469	1426	1448	3520	4262	3891
0.0057	6.93E-58	-1.21	2343	2273	2308	5436	6069	5753
0.0125	6.94E-46	-0.95	4645	4560	4603	9283	9858	9571
0.0121	3.49E-26	-1	1275	1140	1208	2514	2678	2596
0.0174	0.037098	-0.75	70	74	72	128	133	130.5
0.0096	1.78E-27	-0.92	1813	1774	1794	3494	3825	3660
0.0169	7.42E-14	-0.74	2351	1758	2055	3443	3917	3680
0.0119	0.00058	-0.75	219	284	251.5	435	481	458
0.0088	0.000783	-4.65	0	2	1	24	31	27.5
0.0029	3.43E-06	-2.21	19	26	22.5	105	120	112.5

0.0113	0.014271	-2.37	3	7	5	31	25	28
0.0076	0.015709	-2.81	5	1	3	25	19	22
0.0019	0.005767	-5.81	0	0	0	13	9	11
0.0025	0.023415	-3.11	4	0	2	18	18	18
0.0128	0.005933	-0.91	76	88	82	154	180	167
0.0385	1.82E-05	-2.07	23	22	22.5	95	108	101.5
0.008	2.01E-42	-1.33	1275	1341	1308	3496	3588	3542
0.0178	2.63E-56	-1.06	4231	4041	4136	8638	9983	9311
0.0066	6.36E-11	-0.67	1593	1666	1630	2824	2756	2790
0.0177	2.16E-45	-1.47	839	795	817	2257	2612	2435
0.0051	2.15E-23	-1.09	908	869	888.5	2022	2030	2026
0.0062	4.08E-14	-1.23	327	332	329.5	833	828	830.5
0.0068	0.000322	-2.42	7	14	10.5	54	68	61
0.0532	1.31E-32	-0.8	3807	3427	3617	6555	7000	6778
0.0052	1.17E-12	-1.68	126	105	115.5	378	418	398
0.0172	1.48E-29	-1.64	352	371	361.5	1174	1243	1209
0.0172	4.03E-19	-1.05	852	726	789	1763	1731	1747
0.0048	3.42E-179	-3.93	155	132	143.5	2244	2459	2352
0.015	5.60E-175	-1.92	2778	2628	2703	10793	11096	10945
0.0133	0.032084	-5.17	0	0	0	10	4	7
0.0201	4.98E-06	-0.71	608	774	691	1195	1246	1221
0.0119	1.56E-10	-1.22	265	348	306.5	738	801	769.5
0.0437	9.88E-39	-1.43	873	933	903	2585	2639	2612
0.0155	9.73E-15	-0.63	2349	2261	2305	3752	3922	3837
0.0044	1.72E-08	-8.71	0	0	0	76	90	83
0.1327	1.24E-16	-0.93	1029	1107	1068	2147	2221	2184
0.0132	4.46E-09	-0.71	781	762	771.5	1262	1462	1362
0.0119	1.90E-147	-2.14	1347	1119	1233	5483	6180	5832
0.0121	1.32E-217	-2.61	1000	847	923.5	5722	6414	6068
0.0157	1.37E-14	-0.85	1135	899	1017	1887	2049	1968
0.0248	0	-3.78	534	480	507	7019	7944	7482
0.0069	0.000779	-0.94	188	127	157.5	243	409	326
0.0118	1.09E-07	-8.36	0	0	0	57	73	65
0.0043	3.18E-48	-1.49	906	854	880	2631	2660	2646
0.0108	4.88E-165	-2.08	1979	1641	1810	7678	8754	8216
0.03	5.35E-34	-2.02	7074	8110	7592	32085	34196	33141
0.0124	1.16E-28	-0.89	2562	2135	2349	4414	4954	4684
0.0066	2.28E-34	-1.3	859	845	852	2101	2421	2261
0.008	4.51E-12	-0.77	1033	856	944.5	1631	1831	1731
0.1811	5.23E-08	-0.62	1815	1334	1575	2621	2546	2584
0.0946	4.65E-07	-0.7	847	610	728.5	1228	1303	1266
0.0062	0.043158	-4.26	1	0	0.5	4	17	10.5
0.0172	1.08E-13	-1.29	277	318	297.5	760	814	787
0.0555	1.59E-258	-2.11	3400	3236	3318	14848	15948	15398
0.0223	1.53E-20	-0.77	1937	1786	1862	3252	3584	3418
0.0206	2.42E-78	-2.31	698	537	617.5	2806	3762	3284
0.0569	2.21E-26	-0.63	19547	16129	17838	28434	30876	29655
0.0246	6.07E-08	-0.64	1155	879	1017	1629	1771	1700

0.0122	2.79E-24	-1.24	702	569	635.5	1527	1692	1610
0.005	8.54E-41	-5.02	11	14	12.5	438	433	435.5
0.0081	3.82E-69	-1.61	1202	1155	1179	3538	4192	3865
0.0118	1.15E-11	-0.8	980	827	903.5	1513	1873	1693
0.0245	2.84E-14	-0.88	1547	1114	1331	2596	2630	2613
0.0103	4.51E-07	-0.61	1742	1384	1563	2147	2978	2563
0.0061	6.17E-05	-1.14	205	100	152.5	361	352	356.5
0.0118	1.73E-31	-1.17	1285	1109	1197	2604	3206	2905
0.0101	0.000305	-1.02	138	109	123.5	215	325	270
0.0072	7.28E-75	-1.51	1869	1834	1852	5190	6158	5674
0.0144	0.001288	-0.69	220	203	211.5	346	389	367.5
0.0474	6.39E-09	-0.59	2260	1713	1987	3170	3211	3191
0.0338	9.15E-44	-1.5	750	664	707	2025	2259	2142
0.0074	3.78E-26	-1.04	1896	1439	1668	3456	3895	3676
0.0111	4.12E-10	-0.7	946	842	894	1515	1605	1560
0.0127	1.20E-17	-0.88	1345	1100	1223	2222	2621	2422
0.025	2.38E-26	-1.1	1445	1115	1280	2885	2982	2934
0.1682	1.13E-51	-0.89	21591	20846	21219	41904	42295	42100
0.0514	5.05E-104	-2.78	379	274	326.5	2312	2480	2396
0.0243	6.84E-64	-1.98	593	672	632.5	2544	2843	2694
0.0067	2.67E-11	-1.35	209	226	217.5	511	687	599
0.0106	2.34E-16	-0.71	1828	1721	1775	2940	3291	3116
0.0099	7.42E-56	-1.19	2644	2597	2621	6274	6541	6408
0.0171	1.45E-16	-0.81	1304	1268	1286	2360	2472	2416
0.0409	3.23E-44	-1.16	2523	2028	2276	5102	5814	5458
0.0247	3.05E-08	-0.68	799	702	750.5	1195	1389	1292
0.0507	5.22E-11	-0.84	715	632	673.5	1168	1432	1300
0.0375	0	-6.79	87	69	78	9081	9453	9267
0.0643	1.76E-43	-0.73	16376	14385	15381	26220	28450	27335
0.033	0	-6.39	126	148	137	11673	13012	12343
0.0149	1.60E-57	-1.31	1628	1544	1586	4034	4439	4237
0.0138	3.94E-19	-0.65	3108	2799	2954	4751	5192	4972
0.0045	1.39E-18	-1.51	274	274	274	874	805	839.5
0.0097	2.10E-33	-1.85	423	295	359	1258	1506	1382
0.0119	1.49E-31	-1.05	1511	1326	1419	2947	3358	3153
0.0102	1.63E-10	-9.29	0	1	0.5	342	328	335
0.0116	2.66E-61	-1.82	713	631	672	2290	2805	2548
0.0216	2.98E-165	-2.77	614	486	550	3775	4285	4030
0.0097	8.45E-08	-0.69	1092	1342	1217	1985	2273	2129
0.0093	5.19E-10	-9.05	1	0	0.5	289	281	285
0.0806	1.89E-39	-1.27	1652	1515	1584	3596	4648	4122
0.0166	1.34E-09	-0.91	734	497	615.5	1186	1289	1238
0.017	0.048642	-2.36	6	1	3.5	16	22	19
0.0116	8.11E-16	-0.63	3003	2577	2790	4305	4959	4632
0.0084	3.43E-83	-1.86	1151	1256	1204	4560	4831	4696
0.019	4.20E-16	-0.85	1091	959	1025	1853	2117	1985
0.0107	4.03E-32	-1.01	1822	1701	1762	3508	4126	3817
0.0093	2.27E-31	-1.59	814	544	679	2004	2381	2193

0.0107	1.29E-09	-0.8	676	628	652	1092	1352	1222
0.0475	1.80E-26	-0.8	2867	2538	2703	4950	5151	5051
0.0086	6.94E-55	-1.47	1130	985	1058	2928	3381	3155
0.0067	6.47E-22	-1.25	525	509	517	1268	1368	1318
0.0115	1.36E-20	-1.38	423	338	380.5	1042	1081	1062
0.0049	2.78E-102	-2.06	771	698	734.5	3144	3422	3283
0.0079	9.06E-30	-1.25	819	733	776	1962	1996	1979
0.0159	0.028821	-5.22	0	0	0	4	11	7.5
0.0079	2.42E-24	-0.94	1430	1240	1335	2626	2884	2755
0.0153	1.24E-51	-1.54	979	809	894	2597	2976	2787
0.0092	0.013063	-0.94	82	50	66	127	143	135
0.0212	5.06E-23	-0.62	6137	5360	5749	8953	10033	9493
0.0069	1.61E-28	-1.42	535	513	524	1517	1495	1506
0.0278	0.023088	-1.07	72	43	57.5	70	192	131
0.0127	3.10E-14	-1.73	125	119	122	424	445	434.5
0.037	1.27E-12	-0.63	2085	1747	1916	3086	3253	3170
0.0106	2.14E-09	-1.03	307	332	319.5	673	735	704
0.0112	4.29E-17	-1.5	273	220	246.5	758	731	744.5
0.0174	6.03E-07	-0.97	240	230	235	464	528	496
0.0129	1.24E-05	-0.62	622	643	632.5	1056	1026	1041
0.0093	6.78E-07	-0.73	527	507	517	928	906	917
0.0232	1.88E-30	-0.8	3843	3734	3789	6818	7331	7075
0.0171	0.027476	-0.65	122	107	114.5	199	186	192.5
0.0075	6.68E-16	-1.02	674	567	620.5	1266	1432	1349
0.0113	3.79E-31	-1.1	1614	1312	1463	3325	3389	3357
0.0187	2.99E-26	-1.08	1226	992	1109	2441	2594	2518
0.016	1.91E-23	-0.87	1749	1568	1659	3214	3305	3260
0.0054	5.49E-31	-1.71	317	300	308.5	1048	1126	1087
0.0201	8.66E-16	-0.74	1535	1508	1522	2631	2851	2741
0.0164	2.13E-06	-0.78	559	388	473.5	814	925	869.5
0.0059	4.23E-49	-1.45	1060	907	983.5	2670	3112	2891
0.0118	6.82E-112	-2.21	688	681	684.5	3224	3596	3410
0.0066	0.016599	-5.42	0	0	0	8	9	8.5
0.0271	4.93E-48	-1.83	727	520	623.5	2181	2570	2376
0.0055	6.47E-08	-0.96	298	326	312	631	676	653.5
0.0068	5.31E-12	-0.8	1154	925	1040	1737	2161	1949
0.0095	1.27E-15	-0.94	796	697	746.5	1430	1647	1539
0.0153	4.04E-33	-1.04	2026	1654	1840	3865	4229	4047
0.0095	1.44E-17	-0.92	1090	1154	1122	2231	2340	2286
0.01	4.05E-09	-1.04	308	265	286.5	624	637	630.5
0.0119	4.15E-08	-0.74	767	885	826	1485	1487	1486
0.0069	9.22E-12	-0.8	1071	870	970.5	1626	1998	1812
0.0058	0.000399	-0.78	195	218	206.5	366	400	383
0.0217	0	-2.85	1373	1163	1268	9124	10464	9794
0.0333	0.001307	-0.81	159	144	151.5	293	275	284
0.0254	3.10E-17	-0.87	1100	1087	1094	2040	2256	2148
0.0129	8.63E-09	-0.81	640	495	567.5	1012	1124	1068
0.0048	7.14E-08	-1.05	260	212	236	511	532	521.5

0.0353	7.25E-35	-2.04	314	428	371	1480	1825	1653
0.0034	0.001505	-0.62	284	269	276.5	416	497	456.5
0.0082	0.00061	-1.86	25	16	20.5	66	94	80
0.0049	2.69E-07	-8.17	0	0	0	57	57	57
0.0081	1.21E-12	-0.95	577	497	537	1057	1176	1117
0.0101	4.62E-185	-2.44	930	833	881.5	4893	5405	5149
0.0056	9.65E-09	-0.9	440	510	475	946	971	958.5
0.0072	5.34E-05	-4.38	4	0	2	27	62	44.5
0.0282	1.43E-08	-0.95	349	310	329.5	660	702	681
0.0087	3.16E-18	-1.2	547	586	566.5	1254	1548	1401
0.0394	0	-3.3	1119	949	1034	10541	11344	10943
0.0064	2.33E-19	-1.38	356	396	376	1008	1103	1056
0.0424	4.29E-19	-1.35	654	432	543	1464	1491	1478
0.0139	9.56E-37	-0.96	3423	2807	3115	6187	6786	6487
0.0092	0.001557	-0.77	168	155	161.5	266	328	297
0.007	1.81E-19	-1.88	138	141	139.5	517	591	554
0.0089	0.000339	-1.91	23	17	20	75	86	80.5
0.0086	1.67E-05	-7.32	0	0	0	31	32	31.5
0.009	5.19E-12	-3.19	13	18	15.5	143	162	152.5
0.0078	1.18E-19	-0.75	2436	2473	2455	4269	4610	4440
0.0125	1.66E-53	-1.36	1595	1612	1604	4364	4465	4415
0.018	1.99E-10	-0.65	1199	1095	1147	1879	1985	1932
0.0195	3.66E-18	-1.19	498	427	462.5	1073	1198	1136
0.0085	1.16E-09	-0.75	1374	1109	1242	1891	2602	2247
0.0174	4.17E-07	-0.75	775	537	656	1118	1238	1178
0.0027	1.09E-06	-3.84	4	4	4	59	64	61.5
0.0087	0.012841	-2.67	4	3	3.5	18	30	24
0.009	0.000332	-4.46	4	31	17.5	364	496	430
0.0121	1.26E-06	-1.1	186	163	174.5	425	376	400.5
0.0095	1.27E-29	-1.03	1399	1230	1315	2750	3025	2888
0.0077	6.99E-10	-3.2	16	9	12.5	105	142	123.5
0.0067	0.000206	-1.1	92	125	108.5	227	276	251.5
0.0071	1.39E-44	-1.42	983	1000	991.5	2651	3078	2865
0.0065	1.04E-46	-2.21	244	223	233.5	1147	1163	1155
0.0143	2.37E-45	-1.64	589	524	556.5	1798	1922	1860
0.0487	1.70E-85	-1.71	1215	1056	1136	3832	4132	3982
0.0131	1.09E-05	-1.23	126	88	107	255	280	267.5
0.0184	1.39E-43	-1.19	1525	1395	1460	3359	3825	3592
0.0172	5.85E-05	-5.2	1	1	1	26	54	40
0.0058	4.02E-26	-1.19	761	734	747.5	1801	1871	1836
0.0099	4.09E-09	-0.72	817	877	847	1433	1582	1508
0.01	2.57E-12	-0.94	661	567	614	1123	1411	1267
0.0022	4.43E-25	-1.88	193	225	209	800	854	827
0.0377	6.67E-05	-0.95	168	147	157.5	297	357	327
0.0066	1.68E-12	-0.85	905	717	811	1487	1645	1566
0.0099	9.50E-46	-1.31	1382	1344	1363	3315	3945	3630
0.0048	0.000338	-0.63	373	318	345.5	563	580	571.5
0.0027	6.99E-05	-0.66	425	379	402	700	661	680.5

0.0083	0.03174	-5.16	0	0	0	9	5	7
0.0095	0.000307	-0.59	443	487	465	766	743	754.5
0.0403	2.26E-105	-1.28	7185	6211	6698	16510	18324	17417
0.0682	2.04E-13	-0.6	2212	2007	2110	3318	3548	3433
0.0116	5.43E-21	-1.55	322	250	286	827	971	899
0.0373	1.64E-24	-0.86	2361	2410	2386	4591	4740	4666
0.0372	3.54E-38	-2.77	88	91	89.5	603	716	659.5
0.0151	4.35E-58	-1.91	558	493	525.5	2139	2082	2111
0.0128	3.36E-12	-0.69	1317	1300	1309	2212	2331	2272
0.0093	2.38E-06	-0.69	696	516	606	960	1139	1050
0.0091	1.49E-07	-0.87	361	364	362.5	706	717	711.5
0.0139	0.000243	-0.76	256	216	236	432	421	426.5
0.0085	1.36E-07	-0.83	567	421	494	964	915	939.5
0.0163	1.93E-66	-1.61	1009	948	978.5	3021	3391	3206
0.0116	2.31E-54	-1.93	770	950	860	3561	3504	3533
0.0094	4.89E-12	-0.64	1918	1815	1867	3176	3075	3126
0.0129	0.000106	-0.74	296	350	323	540	622	581
0.0046	1.12E-09	-0.85	605	602	603.5	1202	1128	1165
0.0151	2.60E-12	-1.05	462	388	425	861	1026	943.5
0.0393	1.76E-10	-1.23	7060	8330	7695	19325	19612	19469
0.0065	2.62E-10	-0.62	1871	1475	1673	2586	2938	2762
0.0129	1.59E-05	-0.62	587	538	562.5	835	1022	928.5
0.0212	2.13E-17	-0.76	1633	1533	1583	2709	3045	2877
0.0058	2.88E-12	-10.3	0	0	0	237	268	252.5
0.0073	7.55E-19	-0.84	1936	2108	2022	3725	4096	3911
0.0072	2.20E-08	-0.76	617	604	610.5	1121	1099	1110
0.0139	5.61E-31	-1.09	1878	2001	1940	4440	4437	4439
0.0101	2.84E-21	-0.87	1491	1387	1439	2780	2868	2824
0.0243	5.62E-19	-0.69	2877	2872	2875	4771	5228	5000
0.0141	1.29E-11	-0.97	473	443	458	900	1032	966
0.012	4.02E-14	-0.59	2474	2310	2392	3738	4020	3879
0.0073	2.25E-26	-1.16	826	778	802	1863	1979	1921
0.0072	6.21E-13	-1.33	271	328	299.5	714	912	813
0.0142	3.88E-15	-2.5	44	43	43.5	265	265	265
0.0035	8.25E-74	-2.29	396	337	366.5	1765	2090	1928
0.0134	2.45E-65	-1.68	1728	1396	1562	4697	6094	5396
0.0078	2.78E-38	-1.17	1790	1569	1680	3641	4480	4061
0.0066	1.95E-26	-1.01	1307	1136	1222	2521	2750	2636
0.0085	1.17E-42	-1.53	868	798	833	2269	2909	2589
0.0061	9.95E-19	-1.27	457	519	488	1192	1357	1275
0.0096	3.65E-09	-2	56	43	49.5	217	205	211
0.0144	2.20E-33	-1.69	369	333	351	1138	1304	1221
0.0094	6.91E-18	-1	1088	1231	1160	2505	2497	2501
0.0099	2.45E-10	-0.78	813	729	771	1452	1393	1423
0.0176	1.99E-16	-1.08	599	504	551.5	1174	1324	1249
0.0149	4.04E-13	-0.74	1293	1080	1187	2010	2249	2130
0.0057	2.10E-39	-1.6	536	550	543	1694	1847	1771
0.0343	2.64E-17	-0.66	2487	2233	2360	3894	4129	4012

0.0126	5.13E-28	-1.46	783	542	662.5	1836	2069	1953
0.0077	2.20E-07	-0.6	961	847	904	1348	1607	1478
0.0152	3.69E-29	-1.11	1182	1177	1180	2721	2764	2743
0.0062	4.15E-08	-1.8	56	58	57	190	238	214
0.0086	5.37E-17	-1.39	300	265	282.5	731	861	796
0.0146	1.66E-14	-1.11	437	448	442.5	985	1071	1028
0.0102	1.76E-17	-0.93	935	813	874	1743	1839	1791
0.0051	4.43E-07	-0.87	452	345	398.5	676	885	780.5
0.0048	8.26E-26	-1.07	1018	953	985.5	2179	2274	2227
0.0131	0.007611	-0.84	89	124	106.5	211	201	206
0.0125	3.88E-22	-1.31	565	467	516	1392	1343	1368
0.0054	2.21E-32	-1.3	774	704	739	1901	2004	1953
0.0051	1.24E-05	-1.6	66	50	58	222	152	187
0.0103	4.29E-07	-1.14	276	168	222	542	501	521.5
0.0128	1.02E-14	-0.78	1162	1073	1118	1944	2188	2066
0.0072	1.23E-06	-0.61	747	682	714.5	1108	1229	1169
0.0247	4.34E-09	-0.61	2937	2312	2625	3688	4935	4312
0.0169	1.21E-09	-0.62	1230	1083	1157	1819	2002	1911
0.0206	2.51E-15	-1.26	576	624	600	1727	1355	1541
0.0259	1.67E-09	-0.59	1522	1367	1445	2322	2331	2327
0.0169	3.61E-05	-0.63	472	428	450	700	801	750.5
0.0231	0.00061	-0.6	352	373	362.5	572	611	591.5
0.0144	2.92E-11	-0.74	924	858	891	1519	1680	1600
0.0288	7.10E-59	-1.59	877	824	850.5	2617	2896	2757
0.0103	6.41E-10	-0.7	1078	1041	1060	1649	2075	1862
0.0098	3.60E-06	-2.59	11	18	14.5	108	80	94
0.0185	4.11E-169	-3.69	218	163	190.5	2616	2646	2631
0.0098	6.17E-15	-0.77	1427	1277	1352	2249	2711	2480
0.0077	3.50E-10	-1.29	189	198	193.5	464	554	509
0.0129	5.91E-26	-0.85	2432	2252	2342	4154	4909	4532
0.0092	2.54E-20	-0.67	4332	3688	4010	6325	7380	6853
0.0086	3.10E-45	-1.43	936	948	942	2577	2894	2736
0.0158	1.54E-15	-0.83	1374	1114	1244	2183	2555	2369
0.0114	3.61E-20	-0.73	2693	2629	2661	4658	4809	4734
0.0094	2.32E-08	-0.8	512	462	487	877	943	910
0.0266	3.88E-25	-0.75	4214	3481	3848	6629	7280	6955
0.0129	1.29E-65	-1.53	1246	1082	1164	3395	3819	3607
0.0271	2.93E-203	-3.55	252	231	241.5	2881	3192	3037
0.0189	2.75E-286	-2.6	1868	1585	1727	10396	12026	11211
0.0253	2.20E-08	-1.02	327	251	289	619	633	626
0.0052	2.18E-40	-1.25	1449	1181	1315	3250	3441	3346
0.0141	4.73E-19	-0.91	1367	1435	1401	2806	2844	2825
0.0066	9.64E-19	-2.66	43	80	61.5	383	466	424.5
0.0639	2.02E-12	-1.2	303	348	325.5	762	856	809
0.0236	2.77E-06	-0.77	430	374	402	650	825	737.5
0.0409	2.41E-152	-1.78	6477	5221	5849	20105	23069	21587
0.0077	2.21E-07	-0.59	973	939	956	1510	1571	1541
0.0097	2.39E-74	-1.33	2655	2354	2505	6276	7247	6762

0.0177	8.60E-39	-1.15	2900	3220	3060	6955	7667	7311
0.0093	2.75E-24	-1.14	780	733	756.5	1675	1921	1798
0.0042	1.86E-07	-0.93	432	305	368.5	669	835	752
0.0106	2.16E-06	-0.99	350	215	282.5	590	602	596
0.0117	4.05E-07	-6.02	1	1	1	50	91	70.5
0.0114	1.36E-13	-0.82	1536	1134	1335	2426	2614	2520
0.0089	1.25E-32	-1.04	2355	2429	2392	5294	5254	5274
0.023	9.20E-144	-1.72	5334	4416	4875	16992	17364	17178
0.0156	5.95E-31	-1.24	832	803	817.5	1983	2163	2073
0.0345	3.81E-276	-2.19	3097	2910	3004	13964	15601	14783
0.0131	0.028999	-1	338	333	335.5	470	990	730
0.0227	2.04E-35	-2.07	283	199	241	1066	1103	1085
0.0726	2.08E-102	-5.08	318	215	266.5	8449	10892	9671
0.0243	1.16E-14	-0.66	2214	2052	2133	3620	3611	3616
0.01	5.90E-18	-1	1214	909	1062	2265	2277	2271
0.0067	4.34E-32	-1.41	606	587	596.5	1582	1834	1708
0.0097	4.30E-16	-6.56	4	0	2	192	211	201.5
0.0106	1.59E-08	-2.26	28	37	32.5	142	197	169.5
0.0216	1.20E-54	-0.95	7447	6404	6926	13478	15332	14405
0.0176	5.26E-65	-1.53	1880	1507	1694	4774	5733	5254
0.0129	3.27E-131	-2.11	1873	1427	1650	7217	8070	7644
0.0113	1.43E-122	-2.04	1337	1099	1218	4982	5788	5385
0.006	5.04E-53	-1.25	1721	1522	1622	3979	4324	4152
0.007	3.02E-16	-0.6	3257	2954	3106	4715	5425	5070
0.0147	6.36E-76	-2.06	947	951	949	4527	3959	4243
0.0064	6.80E-54	-1.76	1032	793	912.5	2908	3729	3319
0.0305	8.91E-45	-0.95	3847	3377	3612	7231	7715	7473
0.0159	6.57E-36	-4.34	22	14	18	381	400	390.5
0.013	6.26E-28	-1.47	449	403	426	1236	1302	1269
0.0145	0.000712	-6.42	0	0	0	15	19	17
0.0085	0.009584	-2.27	9	11	10	89	12	50.5
0.0075	4.31E-09	-2.19	32	70	51	255	250	252.5
0.0076	2.82E-46	-1.23	1989	1678	1834	4217	5050	4634
0.0171	2.65E-43	-1.03	3287	3251	3269	7113	7256	7185
0.0067	8.60E-10	-0.79	827	929	878	1485	1807	1646
0.016	1.65E-46	-1.37	1197	1242	1220	3243	3548	3396
0.0123	0.003699	-0.87	111	91	101	191	204	197.5
0.0056	1.70E-90	-2.63	322	257	289.5	1841	2002	1922
0.0228	4.10E-57	-0.95	70523	71983	71253	1E+05	2E+05	1E+05
0.0072	8.41E-60	-2.02	597	722	659.5	2795	2982	2889
0.0187	1.94E-183	-3.84	239	161	200	3004	3110	3057
0.0538	2.29E-06	-0.65	749	578	663.5	1103	1127	1115
0.0271	1.07E-45	-2.06	287	265	276	1193	1277	1235
0.0168	8.59E-118	-1.71	1905	1685	1795	6006	6641	6324
0.0281	1.34E-55	-1.18	5369	4216	4793	10948	12252	11600
0.0054	1.07E-05	-0.75	385	349	367	590	735	662.5
0.0582	6.56E-35	-0.79	4547	4168	4358	7822	8385	8104
0.01	2.61E-22	-1.22	600	628	614	1498	1586	1542

0.033	8.79E-32	-0.79	10052	8696	9374	15645	19118	17382
0.0078	7.92E-21	-0.89	1374	1189	1282	2450	2643	2547
0.0072	1.45E-52	-1.5	955	875	915	2585	2983	2784
0.0065	1.60E-19	-1.23	1058	1094	1076	3017	2381	2699
0.0244	3.19E-163	-2.45	883	918	900.5	5114	5469	5292
0.0024	2.99E-58	-1.82	657	647	652	2509	2430	2470
0.0099	6.92E-193	-2.51	1448	1125	1287	7388	8301	7845
0.0163	4.24E-12	-0.85	782	669	725.5	1295	1506	1401
0.0411	2.55E-47	-1.24	1847	1907	1877	4575	4953	4764
0.0637	1.02E-36	-0.76	9983	8996	9490	15852	18597	17225
0.0085	3.67E-15	-0.79	1206	1065	1136	1993	2225	2109
0.0058	3.96E-24	-0.78	2533	2313	2423	4196	4722	4459
0.0078	8.67E-17	-12.1	0	0	0	868	862	865
0.0273	4.72E-24	-1.16	860	697	778.5	1790	1930	1860
0.0105	3.14E-35	-1.21	1418	1127	1273	3030	3265	3148
0.0086	8.64E-08	-1.12	190	209	199.5	457	479	468
0.0102	3.76E-20	-1.31	455	379	417	1081	1132	1107
0.0187	1.72E-16	-0.87	1016	911	963.5	1796	1984	1890
0.0126	3.20E-06	-0.59	817	846	831.5	1230	1461	1346
0.0199	4.68E-30	-0.77	3807	3491	3649	6316	7073	6695
0.012	6.31E-11	-0.98	564	431	497.5	948	1153	1051
0.0173	3.62E-09	-0.88	1026	656	841	1506	1802	1654
0.0071	4.52E-13	-1.08	480	564	522	1159	1222	1191
0.0188	1.81E-101	-1.66	1857	1835	1846	5920	6646	6283
0.0146	4.06E-11	-0.99	450	379	414.5	854	914	884
0.0145	1.73E-18	-1.2	491	520	505.5	1219	1288	1254
0.0064	9.04E-21	-1.31	482	515	498.5	1339	1313	1326
0.1086	2.32E-183	-1.89	3725	3126	3426	13090	14121	13606
0.0056	1.02E-18	-1.06	870	838	854	1989	1831	1910
0.0215	6.29E-11	-1.3	243	182	212.5	525	593	559
0.0113	1.73E-48	-1.31	1528	1490	1509	4000	4010	4005
0.0105	0.026022	-1.51	21	10	15.5	43	51	47
0.0201	4.67E-11	-0.89	674	775	724.5	1353	1551	1452
0.0071	3.30E-19	-1.27	467	401	434	1029	1213	1121
0.0245	2.35E-10	-0.91	485	479	482	943	999	971
0.0199	2.71E-05	-5.32	0	2	1	37	50	43.5
0.0019	0.000582	-0.93	118	142	130	268	267	267.5
0.0173	0.001962	-6.14	0	0	0	12	16	14
0.0081	1.49E-09	-1.1	347	257	302	707	676	691.5
0.0358	0.018262	-0.64	160	124	142	240	232	236
0.0065	3.56E-08	-0.82	517	457	487	944	898	921
0.0171	8.60E-22	-1.08	914	756	835	1757	2037	1897
0.017	1.02E-61	-2.1	537	395	466	2059	2205	2132
0.0512	6.82E-21	-1.05	927	758	842.5	1775	1974	1875
0.0151	0.000529	-0.63	332	323	327.5	546	539	542.5
0.0131	9.07E-85	-1.59	1497	1433	1465	4496	5003	4750
0.0057	3.25E-99	-2.47	419	354	386.5	2222	2381	2302
0.0083	0	-4.34	364	319	341.5	7268	7542	7405

0.0316	3.97E-21	-0.91	1262	1168	1215	2304	2589	2447
0.0082	1.22E-45	-1.4	1008	886	947	2504	2865	2685
0.0114	2.00E-05	-0.73	542	366	454	759	852	805.5
0.0085	4.20E-07	-1.29	129	177	153	345	471	408
0.0036	5.12E-05	-0.82	235	240	237.5	426	474	450
0.0058	4.29E-08	-1.53	105	79	92	285	281	283
0.0069	2.97E-18	-1.15	612	684	648	1530	1572	1551
0.0061	2.75E-47	-2.4	269	189	229	1332	1247	1290
0.0142	2.93E-08	-0.81	581	464	522.5	898	1065	981.5
0.007	7.22E-42	-1.65	749	560	654.5	2185	2215	2200
0.007	8.84E-37	-1.53	847	853	850	2787	2464	2626
0.0086	2.72E-26	-1.16	1178	961	1070	2600	2492	2546
0.0168	8.75E-28	-0.74	3901	3619	3760	6536	6947	6742
0.0066	1.61E-27	-1.69	359	274	316.5	1005	1188	1097
0.0524	2.03E-297	-2.02	6541	5831	6186	26175	27826	27001
0.0194	1.95E-18	-0.65	4809	3894	4352	6995	7630	7313
0.0109	0.022753	-0.91	50	73	61.5	127	122	124.5
0.0136	1.79E-09	-0.64	1243	1041	1142	1888	1931	1910
0.0098	2.30E-16	-0.74	1783	1554	1669	2767	3217	2992
0.0156	6.94E-29	-1.16	956	938	947	2127	2437	2282
0.0104	7.49E-33	-1.96	239	259	249	944	1142	1043
0.009	2.53E-13	-0.81	1755	2083	1919	3535	3719	3627
0.0031	5.38E-05	-0.83	227	219	223	409	442	425.5
0.0172	5.09E-20	-0.75	2456	2467	2462	4351	4569	4460
0.009	1.12E-15	-1.04	904	941	922.5	1728	2382	2055
0.0066	8.84E-16	-0.77	1378	1257	1318	2378	2453	2416
0.0058	3.62E-85	-2.12	803	913	858	3701	4370	4036
0.0046	9.22E-38	-1.31	897	827	862	2212	2392	2302
0.009	1.86E-09	-0.61	1825	1508	1667	2431	3054	2743
0.012	7.74E-55	-1.14	2629	2350	2490	5528	6251	5890
0.0127	9.47E-19	-0.77	1925	1644	1785	3067	3475	3271
0.0063	0.000177	-0.79	227	272	249.5	438	491	464.5
0.0065	1.10E-15	-0.95	818	709	763.5	1572	1582	1577
0.0066	8.73E-09	-8.84	0	0	0	91	90	90.5
0.0077	9.92E-10	-0.63	1925	2170	2048	3189	3654	3422
0.0053	0.000345	-0.6	444	378	411	590	752	671
0.0114	0.004408	-0.97	80	92	86	141	225	183
0.0099	2.84E-13	-1.13	748	487	617.5	1300	1595	1448
0.0073	9.32E-17	-1.26	376	344	360	913	938	925.5
0.0085	1.16E-13	-1.05	517	425	471	985	1113	1049
0.0108	9.35E-16	-11.7	0	0	0	608	714	661
0.0287	1.19E-08	-1.26	182	145	163.5	415	425	420
0.0091	0.001742	-1.21	51	55	53	115	150	132.5
0.0118	0.002021	-0.82	182	119	150.5	268	298	283
0.0092	7.18E-09	-1.13	306	216	261	549	677	613
0.017	1.64E-23	-0.87	2346	2049	2198	4394	4233	4314
0.0056	0.00161	-1.05	78	71	74.5	163	168	165.5
0.0112	1.13E-47	-1.77	729	559	644	2105	2626	2366

0.0263	5.86E-75	-2.4	326	279	302.5	1639	1786	1713
0.007	8.65E-07	-1.06	211	180	195.5	384	491	437.5
0.0021	3.91E-11	-9.85	0	0	0	175	189	182
0.0138	1.67E-53	-1.49	968	883	925.5	2656	2914	2785
0.0042	0.010501	-0.62	171	182	176.5	276	308	292
0.012	4.30E-32	-0.85	3240	2950	3095	5569	6425	5997
0.0054	0.000279	-1	118	118	118	262	243	252.5
0.0019	1.90E-16	-1.7	199	140	169.5	570	607	588.5
######	0.000322	-3.33	1	7	4	52	35	43.5
0.003	1.73E-50	-1.83	466	449	457.5	1664	1823	1744
0.004	7.41E-14	-0.66	2001	1968	1985	3116	3635	3376
0.0006	0.004051	-1.57	26	20	23	82	63	72.5
0.002	1.95E-12	-10.4	0	0	0	312	226	269

Supplemental table S8

ID	sequence
Setd2-RT-F	TAAGGGCTGCTAAGGATCTTCC
Setd2-RT-R	GTGGCATCTATTATCTCGTCATTTT
Cxcl1-RT-F	TGAGCTGCGCTGTCAGTGCCT
Cxcl1-RT-R	TGGCTATGACTTCGGTTTGGGT
Cxcl2-RT-F	CACCAACCACCAGGCTACAGG
Cxcl2-RT-R	GGCTTCAGGGTCAAGGCAAACT
Cxcl3-RT-F	CAGTGCCTGAACACCCTACCAA
Cxcl3-RT-R	GACTTGCCGCTCTTCAGTATCTTC
Cxcl5-RT-F	TCCAGCTCGCCATTCATGC
Cxcl5-RT-R	TTGCGGCTATGACTGAGGAAG
Cxcr2-RT-F	ATGCCCTCTATTCTGCCAGAT
Cxcr2-RT-R	GTGCTCCGGTTGTATAAGATGAC
Csf1-RT-F	ATGAGCAGGAGTATTGCCAAGG
Csf1-RT-R	TCCATTCCCAATCATGTGGCTA
Csf2-RT-F	GGCCTTGGAAGCATGTAGAGG
Csf2-RT-R	GGAGAACTCGTTAGAGACGACTT
Csf2ra-RT-F	CTGCTCTTCTCCACGCTACTG
Csf2ra-RT-R	GAGACTCGCCGGTGTATCC
Csf2rb-RT-F	AAAAACAGCCAGTGTCCTGTG
Csf2rb-RT-R	GATGCTGACGTTCTTGGGAAG
Csf2rb2-RT-F	TCCAGCCAGATCGTGACCT
Csf2rb2-RT-R	AATCCCCAAGAGATACACTCCA
Cxadr-RT-F	TCTTCTGCTGTCACAGGAAACG
Cxadr-RT-R	CGGTCTTGTAAGCGTACTTGAA
Cxadr-ChIP-1F	CCCTGGTACCTGGTATGGGA
Cxadr-ChIP-1R	GCAGAGGTGGGGTTGCTAAA
Cxadr-ChIP-2F	GTCCCACCATCCTTGACACA
Cxadr-ChIP-2R	CCGTGATGAGCGACCTTGTA
